# Effectiveness of digital health interventions for perinatal depression: a systematic review and meta-analysis

**DOI:** 10.1093/oodh/oqae026

**Published:** 2024-08-03

**Authors:** Ifunanya Stellamaris Anyanwu, Judy Jenkins

**Affiliations:** Department of Health Informatics, Faculty of Medicine, Health, and Life Sciences, Swansea University, Swansea, SA2 8PP, United Kingdom; Department of Health Informatics, Faculty of Medicine, Health, and Life Sciences, Swansea University, Swansea, SA2 8PP, United Kingdom

**Keywords:** digital health, E-health, M-health, telemedicine, teletherapy, perinatal depression, postpartum depression, perinatal depression, antepartum depression

## Abstract

Pregnant women and new mothers within 1 year after delivery are at a high risk of depression, yet many do not get the help they need due to wide reasons heralding stigma, access, cost, time, and shortage of human resources. Hence, compelling the exploration of alternate and potentially cost-effective means of delivering care, including the leverage of digital tools. This review aimed to evaluate the effectiveness of digital health interventions in reducing depressive symptoms among perinatal women. Literatures were sought from seven academic databases alongside the references of previous reviews. Included studies were all quantitative study types involving the use of digital health interventions for perinatal women not more than 1-year post-delivery. Standardized mean difference and standard error were used to perform random-effect model meta-analysis. Sensitivity and subgroup analyses were performed to determine certainty and modifiers of the findings, respectively. Forty-eight studies were included in this review with 28 studies used for meta-analyses. Numerous digital channels were identified; however, none specified the use of a digital health theory in its development. The digital health interventions showed a small positive significant effect over the controls (standardized mean difference = 0.29, *P* = 0.003, *I*^2^ = 34%), and this was significantly influenced by intervention delivery and facilitation modes, time of initiation of the intervention, and period covered by the intervention. Although digital health interventions may hold some potential for perinatal depression, scaling the interventions may be challenging sequel to overlooked influences from the interactions within the human–computer–society complex.

## Lay Summary

The study ‘Effectiveness of digital health interventions for perinatal depression: A systematic review and meta-analysis’ explores the potential of digital tools in managing perinatal depression (PND). PND affects many women during pregnancy and after childbirth, but traditional treatments face challenges like stigma and access barriers. Digital health interventions (DHIs) offer an alternative by providing accessible, personalized support. Although this review confirms that treatment delivery (and/or delivery of prevention schemes) through DHIs can effectively reduce PND symptoms, this review however, found that most DHIs lack foundational theoretical frameworks, potentially limiting their scalability. Notably, DHIs delivered via video conferencing are becoming more popular, indicating a shift towards more user-friendly platforms. Additionally, DHIs for PND were commoner in high-income countries than other country groups, indicating a shift and possible influence of economic might in supporting digital infrastructure. This review highlights the need for future research to integrate digital health theories into DHI design, ensuring interventions are both effective and accessible for women experiencing perinatal depression.

## INTRODUCTION

Perinatal depression (PND) is the occurrence of depression anytime during pregnancy and/or within the first 12 months after childbirth [1]. It is estimated to occur in one out of every ten perinatal women and has dire short- and long-term consequences for the sufferer, the newborn, the family, and society [2, 3]. Several groups of evidenced-based protocols are available for its management including pharmacotherapy, psychotherapy, supportive therapy, educational therapies, physical/sensory therapies and somatic therapies [4]. Nevertheless, while pharmacotherapy is championed as the first-line treatment option in some guidelines, non-medication options are often explored first in PND due to patients’ preferences [5, 6] and concerns about breastfeeding [7]. Common psychotherapy options include cognitive behavioural therapy, interpersonal psychotherapy, problem-solving therapy, mindfulness and behavioural activation while physical/sensory therapies include acupuncture, exercise, yoga, light and music therapy [4, 8]. Despite these wide treatment options, women do not often get the help they need due to various reasons including stigma, unsupportive partners, managing childcare during appointments, cost of care, and the inconvenience of having to leave their homes or fit an extra treatment appointment into their already busy schedules [5–7]. These necessitate the exploration of alternative means of delivering care, including using digital tools.

Digital health interventions (DHIs) encompass the use of digital technologies to address the needs of health and health systems [9]. It might help PND management by enabling accessibility, personalization, and overcoming distance and human resource limitations [10]. However, it is not devoid of flaws with limitations including questionable quality of components and design [11, 12], users’ and providers’ acceptance [10, 13, 14], and non-foundational theoretical designing basis [15]. Nonetheless, previous contemporary reviews [16–22] demonstrated some levels of DHIs’ effectiveness in the reduction of PND symptoms. However, many of them are not representative of the comprehensive perinatal window with most addressing only postpartum depression [17, 19, 22–24]. Yet, studies suggest that these are interconnected as most postpartum depressive episodes actually start in the antepartum period [25, 26]. Furthermore, some reviews addressed only a fraction of DHIs thereby limiting the comprehensiveness of the evidence [16, 21–24, 27–29]. Additionally, most of the reviews were limited by their inclusion of poor-quality studies [20–22, 27, 28, 30, 31] and in some studies, a bias toward a particular measurement scale [19, 22], and others focused on the comorbidity of depression and anxiety [16–18, 20, 21, 27–29, 31–34]. Hence, this review seeks to evaluate the effectiveness of DHIs as options or adjuncts to routine methods of managing PND, using a more comprehensive representation of the perinatal population, DHIs, and measurement scales. Sequentially, the guiding research question was ‘what is the effect of DHIs on depressive symptoms among perinatal women?’ and the specific objectives include to identify and describe the various DHIs that have been quantitatively evaluated for preventing or managing perinatal depression, to identify the theories supporting the development of these DHIs, to evaluate the effectiveness of the DHIs, and to identify sociodemographic and methodological differences that may influence the effectiveness of the DHIs.

## METHOD

This review was reported following the Preferred Reporting Items for Systematic Reviews and Meta-Analyses (PRISMA) statement [35] and the protocol was registered at the International Prospective Register of Systematic Reviews (Registration ID: CRD42024518661).

### Measured outcomes

The primary outcome was the changes in depressive symptoms as measured by any validated measurement scale.

### Eligibility criteria

The eligibility criteria were predominantly defined based on the population, intervention, comparison, outcome and study type (PICOS) strategy alongside other relevant factors (Table 1).

**Table 1:** Eligibility criteria for this review

**Factor**	**Inclusion**	**Exclusion**	**Rationale**
Population	Studies that involved pregnant women or postpartum women up to 12 months post-birth. No restriction was placed on age, ethnic background, socioeconomic status, and level of educational attainment.	Studies on non-pregnant population or among women who are beyond 12 months post-birth. Studies mandating the inclusion of partners or family members of perinatal women. Studies on male partners of perinatal women.	The inclusion of only pregnant of 12-months postpartum women was to align with the definition of perinatal depression as depression occurring anytime in pregnancy or within the first 12 months after birth [1]. The exclusion of partners and family members was because of the assumed confounding effect that they would have on the study population. Studies on male partners were beyond the scope of this review.
Intervention	Interventions for preventing or managing depression, implemented wholly or partially via a digital health platform. Digital health platforms including but not limited to telehealth, telemedicine, electronic health records, social media, websites, mobile applications, video conferences, wearable devices, artificial intelligence, machine learning, mobile health, blockchain, and internet of things [9].	Non-DHIs. Interventions for monitoring, screening for or detecting depression in perinatal population. Non-validated intervention for perinatal depression.	To align with the aims and objectives of the review. Studies on monitoring, screening, and detection of perinatal depression were beyond the scope of this review. The primary focus of this review is on DHIs and not necessarily on treatment options for perinatal depression, hence, only interventions containing validated and existing evidence-based management options were considered as components to be included in the review.
Comparator	Placebo, waiting-list control, an active treatment or treatment as usual implemented via a non-digital platform.	Active control interventions implemented via a digital platform.	To align with the aims and objectives of the review.
Outcome	Studies reporting changes in perinatal, antepartum, or postpartum depression either as a primary or secondary outcome.	Studies not reporting changes in depressive symptoms in perinatal population.	To align with the aims and objectives of the review.
Study Design	Primary research of the quantitative study design type, including individual or cluster randomized and non-randomized controlled trials, pre- and post- single-arm studies, case–control, cohort longitudinal studies, quasi-experimental. Mixed-method study designs with a quantitative component of the type already stipulated in the previous paragraph.	Qualitative study designs. Secondary research including systematic and scoping reviews. Case reports and case series.	Primary quantitative studies represent summative evaluations of interventions and are essential for assessing interventions for routine use [36]. While case reports and case series may report quantitative outcomes, they were considered inappropriate for this review because they are not essentially population-based studies, and the aim of this review is population-oriented [37, 38].
Publication year	Studies published within the past five years.	Studies published on or before the year, 2018	The initial decision of this review was to include papers published within the past 10 years; however, due to the vast amount of evidence returned during the search and screening and sequel to the available time to complete this review, this was further reduced to only include papers published within the past 5 years. Limitation of papers to include only contemporary evidence was necessary because digital health interventions seem to have a high rate of obsolescence [36] due to the high growth rate of available technologies and their capabilities [9]. Hence, to capture relevant findings that are applicable to current times and trend, it was essential to limit the publication year of the studies included in the review.
Language of publication	English language	Non-English language	To align with the researcher’s lingual capability alongside with the language of the master’s program in Swansea University.
Scientific evidence type	Full text articles of published empirical studies, dissertations, and conference papers. Pilot and feasibility studies. Good study quality as screened during methodological assessment.	Protocols, Books, magazines, continuing education units (CEUs), editorials, letters, and meeting abstracts. Economic evaluations. Poor study quality on methodological assessment.	Only empirical studies were considered relevant for this review because the objectives of this systematic review rely on data from empirical studies. Economic evaluations were beyond this review’s scope. Protocols were excluded because they do not contain study data yet [39]. Dissertations and conference papers were considered in order to account for publication bias. Nonetheless, to be amenable to methodological appraisal, the full-text of all included studies had to be retrievable [39], hence, only full-texts were allowed. Furthermore, to ensure that only studies of good quality are used for the review, only studies with good study quality will be included in the review.
Geographical setting	No limitations		No restriction was set for these factors in order to allow for the collation of all relevant papers without extreme limitations.
Setting or context of intervention			
Measurement tools used			
Meta-analysis conditions	To be eligible for inclusion into the meta-analysis, a study had to be a randomized controlled trial (RCT), reporting continuous outcomes of at least two time-points measurements of depressive symptoms including baseline measurement. It also had to report the *P*-value of effect changes to enable the calculation of data to be used for the meta-analysis [40].		

### Searching and selection of studies

The search strategy comprised keywords generated using the PICOS paradigm and further expanded from pilot search alongside extracted keywords from previous similar reviews and protocols [16, 19, 20, 23, 27, 28, 31, 32, 34, 41, 42]. The final generated keywords per paradigm are presented in Table 2 below. These were further refined with Medical Subject Headings and tailored to fit the orientation of each database employed during the search. The final search sequence per database is presented in Supplementary file.

**Table 2:** Keywords used for the search strategy

**Paradigm**	**Keyword**
Population	(pregnan^*^ or perinatal OR ‘peri-natal’ OR prepartum OR ‘pre-partum’ OR antenatal OR ‘ante-natal’ OR birth^*^ OR childbirth OR ‘child-birth’ OR ‘child birth’ OR postnatal OR ‘post-natal’ OR postpartum OR ‘post-partum’ OR peripartum OR ‘peri-partum’ OR antepartum OR ‘ante-partum’ OR prenatal OR ‘pre-natal’ OR matern^*^ OR “after birth” OR puerper^*^ OR obstetric^*^ OR mother^*^ OR mums OR mum OR moms OR mom OR prepartal OR ‘pre-partal’ OR childbearing)
	AND
Intervention (Comparison keyword not necessary for search)	(digital OR ‘digital health’ OR telehealth OR phone^*^ OR ‘cell-phone’ OR cellphone OR ‘cell phone’ OR telephone OR ‘mobile-phone’ OR smartphone^*^ OR ‘smart-phone’ OR telemedic^*^ OR telecare OR telesupport OR ‘tele-health’ OR ‘tele-medicine’ OR ‘tele-care’ OR ‘tele-support’ OR wearable^*^ OR mobile OR mhealth OR m-health OR web^*^ OR ‘web-based’ OR ‘web-guided’ OR ‘web-supported’ OR ‘web-delivered’ OR ‘web-assisted’ OR ‘web-aided’ OR ‘web-facilitated’ OR ‘web portal’ OR app OR apps OR application^*^ OR ‘mobile apps’ OR ‘mobile app’ OR ‘app-based’ OR ‘web-app’ OR ‘web-apps’ OR ‘social media’ OR facebook OR twitter OR ehealth OR ‘e-health’ OR internet OR ‘internet-based’ OR ‘internet-guided’ OR ‘internet-supported’ OR ‘internet-delivered’ OR ‘internet-assisted’ OR ‘internet-aided’ OR ‘internet-facilitated’ OR online OR ‘on-line’ OR ‘online-based’ OR ‘online-guided’ OR ‘online-supported’ OR ‘online-delivered’ OR ‘online-assisted’ OR ‘online-aided’ OR ‘online-facilitated’ OR computer^*^ OR ‘computer-based’ OR ‘computer-guided’ OR ‘computer-supported’ OR ‘computer-delivered’ OR ‘computer-assisted’ OR ‘computer-aided’ OR ‘computer-facilitated’ OR SMS OR ‘text-based’ OR ‘text based’ OR ‘self-help’ OR ‘self help’ OR ‘self-guided’ OR ‘self guided’ OR ‘self-directed’ OR ‘self directed’ OR ‘self direct’ OR ‘self-direct’ OR telecommunication^*^ OR ‘e-therapy’ OR ‘e-mental’ OR teletherapy OR telemental OR technolog^*^ OR ‘technology-assisted’ OR virtual OR cyber OR cyberpsychology OR ‘cyber psychology’ OR cybertherapy OR iCBT OR cCBT OR eCBT or mCBT OR electronic^*^ OR software OR telepsychiatry OR ‘instant messaging’ OR ‘e-learning’)
	AND
Outcome	(mental^*^ OR depress^*^ OR ‘well-being’ OR ‘well being’ OR wellbeing OR "baby blues" OR distress^*^ OR stress^*^ OR psychological^*^ OR psychiatric)
	AND
Study Type	(trial^*^ OR cohort OR prospective OR retrospective OR ‘case–control’ OR ‘case control’ OR ‘quasi-experimental’ OR ‘quasi experimental’ OR intervention OR ‘user experience’ OR ‘user activity’ OR ‘user log’ OR longitudinal OR efficacy OR effectiveness OR evaluat^*^ OR comparative OR ‘before and after’ OR ‘pre-post’) NOT (systematic OR review OR scoping OR protocol OR qualitative OR "focus group" OR "thematic analysis" OR "meta-analysis" OR predict^*^)

Papers were sought between 8 and 27 January 2024, from seven databases including Medline, Embase, Business Source, APA PsychInfo, the Cumulative Index to Nursing and Allied Health Literature (CINAHL), Web of science, and Cochrane Central Register of Controlled Trials (CENTRAL). Limiters (for year and language of publication) were applied to the databases to reflect the eligibility criteria and to facilitate easier process of the screening.

The search results and screened titles from the databases were all exported and imported into Mendeley®, a reference management system. Additionally, the references of previous similar systematic reviews and potentially eligible papers were screened for other potentially relevant papers. Papers from this network search were combined with the papers from databases, in Mendeley®, and duplicates were removed.

The abstract screening was sequentially conducted in Mendeley®, and the full texts of the screened abstracts sought afterwards. The screening of the retrieved full texts (based on the eligibility criteria) and the documentation of the entire screening process was performed with Microsoft Excel®. The second limitation of publication date eligibility (excluding papers published between 2014 and 2018) was conducted during this stage. Other missed duplicates were identified during the abstract and full-text screening and discarded. Such duplicates included papers published more than once with different titles, or when an intervention is identified to have undergone both a pilot trial and full-powered trial, by similar authors and among similar populations, then the pilot component was discarded. Papers considered eligible from the full-text screening were then appraised for methodological quality and classified as relevant for either systematic review alone or for both systematic review and meta-analysis. The final decision for all screened full-text papers and the detailed documented step-by-step screening are presented in the supplementary file.

### Critical appraisal

Critical appraisal was performed using a validated template—Mixed Methods Appraisal Tool (MMAT) version 2018 [43]. The MMAT was used because of its versatility in appraising multiple study designs and can only be used for empirical studies. It comprises two initial questions for screening papers to ensure they are empirical studies and five separate sections of five questions per section used for screening different study types including qualitative, randomized clinical trials (RCTs), non-randomized, quantitative descriptive, and mixed-methods studies [43]. Each question is answered with either ‘yes’ for clear conformity or ‘no’ for overt non-conformity, or ‘can’t tell’ for when there is not enough information to judge.

Sequel to the eligibility criteria of this review, the first two questions of the MMAT were excluded from the assessment in this review since only empirical studies would have been included from the eligibility screening stage of the review. Furthermore, due to the study types included in the eligibility criteria, only the RCTs and non-randomized sections were employed in this review. The mixed-methods section was excluded because the interest of this review was focused only on the quantitative component of the study and not the mixed-method as a whole; hence, the quantitative component of each mixed-method study design was appraised with the respective quantitative appraising component.

One of the appraising questions in both employed sections assessed the completeness of outcome data, and this was measured at a tolerable attrition rate of 20% based on suggestions from previous evidence [44]. Although this tolerable attrition rate was used, it was not considered tangible enough to exclude studies based on only this flaw. Furthermore, for the RCTs, due to the nature of the interventions, double blinding was not realistic for most of the studies; hence, the judgement on the blinding of the outcome assessors was tolerated. Additionally, when the study groups are not comparable and the randomization method was optimal, the study is still included. Nonetheless, when the randomization of a claimed RCT study is not optimal or stipulated, it is flagged as a significant flaw enough to exclude the paper. When a study, whether RCT or not, has so many components with ‘can’t tell’ answers, it is equally excluded because of inadequate information provided by the study. All flaws, whether tolerated or not, were stipulated in the comment section of the appraisal table report. Additionally, to support the judgements made about each appraised paper, the published protocol and/or trial registry, when available, were equally examined for necessary supporting information.

### Assessment of risk of bias

An assessment of risk of bias was further performed for studies used for meta-analyses. This was essential to assess the RCTs using the regular tool for assessing RCTs in meta-analyses – Cochrane’s Risk of Bias assessment tool for randomized trials, version 2.0 (RoB 2.0) [45]. RoB 2.0 contains five domains that assess bias due to randomization process, deviances from planned interventions, missing outcome data, measurement of the outcome, and selection of the reported result, respectively. Judgments about each paper were made following the guidelines for the tool and using the tool’s macro-enabled Microsoft Excel Template [46]. Based on the guideline, each domain and the overall bias were judged as either low risk, having some concerns, or having a high risk. Additionally, as in the critical appraisal section, to support the judgements made about each appraised paper, the published protocol and/or trial registry, when available, were equally examined for necessary supporting information.

### Data extraction

Data extraction was performed using Microsoft Excel® and based on a template adapted from both Cochrane’s guide [47] and the template for intervention description and replication checklist and guide [48]. The use of the latter template was essential for extracting data that will be relevant in addressing the first objective of this study. The extracted data included sufficient details to describe the study, its participants, the intervention, measurement tools, and results.

### Data synthesis

Quantitative content analysis was used to integrate and summarize descriptive findings, and these were presented as tables and dashboards, generated using Tableau® software.

Meta-analysis was used for pooling the extracted quantitative data. The primary outcome in this review is often measured as a continuous outcome with diverse measuring scales [49–51], and when a continuous outcome is measurable with diverse scales, the standardized mean difference (SMD) is used as effect measure of choice for performing meta-analysis in order to correct for the bias introduced by the scale diversity [40]. However, SMD is not routinely reported in studies, hence, necessitating the need to calculate it using a formula that combines regularly reported data (Equation 1):

Equation 1: Formula for calculating SMD. 


$$ SMD=\frac{mean\ difference}{standard\ deviation} $$

The meta-analysis, including the generation of forest plots and statistical outputs, was conducted using Cochrane’s Review Manager version 5.4.1, with a *P* value of ≤0.05 indicating statistical significance. For the pooled SMD, an effect size of 0.2–0.5 was considered small, 0.6–0.8, medium and > 0.8, large [40]. Regarding the interpretation of the forest plot, results to the right were interpreted as favouring DHIs because the investigated outcome (reduced depressive symptoms) was desirable, as against adverse outcomes where results to the left favour intervention [52]. Furthermore, because SMD relies on mean difference and different studies calculate and interpret mean difference differently, with some subtracting the baseline from the post-intervention score, and other papers doing the opposite, this review therefore resorted to calculating mean differences using the distinct data reported in each paper (Equations 2–4) [40] and the mean difference used in calculating the SMD corresponds to the between-within-group mean difference (BWGD).

Equation 2: Formula for within-group difference. 


$$ Within\ group\ mean\ difference= post\ intervention\ score- baseline\ score $$

Equation 3: Formula for between-group difference. 


$$ Between\ group\ difference= Mean\ of\ DHI\ group- Mean\ of\ control\ group $$

Equation 4: Formula for BWGD 


\begin{align*} Between\ within\ group\ mean\ difference= Within\ group\ mean\ difference\\ of\ DHI\ group- Within\ group\ mean\ difference\ of\ control\ group \end{align*}

.

To assess the certainty of the findings from the meta-analysis, sensitivity analysis was conducted by excluding some studies to explore if they influenced the pooled result [39, 40]. The exclusions included pilot studies, studies with total sample size < 100, and studies reporting depression with scales that are not Edinburgh Postnatal Depression Scale (EPDS). A post-hoc decision was also made to test the influence of studies with wide confidence intervals (considered an outlier) and the influence of studies with hybrid intervention platforms. The testing of pilot studies and studies with few sample sizes was based on the scientific claim of the possible over-estimation of effect sizes in such study types [53] while the decision of the measurement scale was because EPDS is the commonest depression measurement tool used for perinatal depression [49].

Given the diversity of interventions and measuring scales used for measuring PND, heterogeneity was anticipated and planned for. It was assessed using a combination of the Inconsistency index (I^2^) and the Cochran’s-Q statistic (Chi^2^) and its *P*-value. An I^2^ statistic of 0%–25% was considered unimportant, 26–50%, low, 51%–75%, moderate, and ≥ 76%, high [54]. When heterogeneity is present, further subgroup analyses were performed to explore the source [39, 40]; regardless, this subgroup analysis aligned to the addressing of the fourth objective of this review. Similarly, to accommodate the anticipated heterogeneity, the random-effect meta-analytic model was used as against the fixed-effect model [39].

When post-intervention measurement scales were administered more than once, the immediate post-intervention score was used for meta-analysis. Nonetheless, in a tabular presentation of both between-group and within-group mean differences, the most distant depression scores were also evaluated.

Additionally, when multiple reports arose from concurrent measurement of depression with multiple measurement scales, then, the EPDS scale, if used as one of the multiples, was used as the primary scale for meta-analysis, because it is the commonest scale for assessing perinatal depression [49]. If EPDS was not used, the scale reported as the primary outcome or reported with continuous measures was adopted.

For studies with more than two arms including the DHI arm, the group receiving regular treatment was used as the control group because the primary intent of this review was to test the effectiveness of the DHI as an alternative or adjunct to routine treatment.

When missing data was met, attempts were made to compute them using validated formulae in Cochrane handbook [40]. Missing data was only finally declared when there were insufficient values to compute the data. Common missing data were standard error and standard deviation which were both essential for meta-analysis and calculating SMD, respectively. The formulas used in the computation are stipulated in Equations 5 and 6 below:

Equation 5: Formula for standard error. 


$$ Standard\ error=\frac{between\ within\ group\ mean\ difference}{t\ statistic} $$

**Where,**


$$ t\ statistic= tinv\left(p\ value, degree\ of\ freedom\right) $$

and


$$ degree\ of\ freedom=\left({sample\ size}_{DHI}-{sample\ size}_{control}\right)-2 $$

Equation 6: Formula for standard deviation. 


$$ Standard\ deviation=\frac{standard\ error}{\sqrt{\left(\frac{1}{{sample\ size}_{DHI}}+\frac{1}{{sample\ size}_{control}}\right)}} $$

The t-statistic represented an estimation of the t-test that would be generated from a paired-samples t-test for estimating differences within groups over time [40] and the formula was generated using an Excel formula (Equation 5). Due to the dependence of the t-statistic on *P*-value, *P*-values became necessary data for conducting the meta-analysis. Nonetheless, as stated by Cochrane’s guideline [40], when the exact value of p-value was not stated, its nearest estimate was used as follows, *P* < 0.001 represented as 0.001, and *P* < 0.05 as 0.05. When *P*-value was simply expressed as >0.05, the study was removed because the *P*-value estimation would have been unreliable due to the wide range of what interprets as *P* > 0.05 [40].

Sequel to the earlier decision to calculate all the mean differences anew and in order to maintain uniformity of estimations, all the calculations was performed anew for all studies using the formulas, and these were done using Microsoft Excel®.

## RESULTS

### Results of the search and screening process

**Figure 1:**
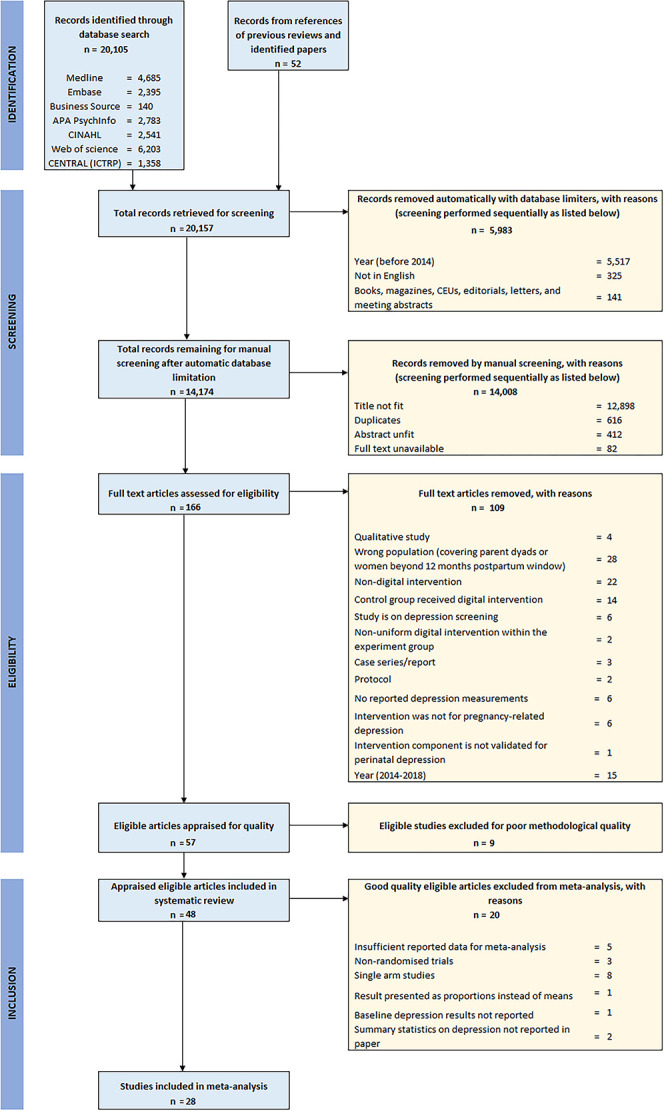
PRISMA flow of the search and selection strategy.

The process of the whole search and screening is summarily reported using the PRISMA flow chart in Fig. 1 below.

The details of the critical appraisal are presented in Table 3 below. Nine studies [55–63] (four RCTs and five non-RCTs) were excluded from the review at this stage due to concerns about the study quality which was mostly contributed by the vague, shallow or absent descriptions of many essential components of the respective studies (Table 3). As planned, none of the included RCTs had any concern due to randomization; nonetheless, many of the papers had issues related to blinding of the outcome assessors mostly because of insufficient information about the blinding of the assessors, which in some cases, were the study participants. Overall, the most noticed concern was a high attrition rate among the studies.

**Table 3:** Methodological appraisal report of eligible papers screened for possible inclusion

**First author and year** ^**a**^	**Study type**	**2.1**	**2.2**	**2.3** ^**b**^	**2.4**	**2.5**	**3.1**	**3.2**	**3.3** ^**b**^	**3.4**	**3.5**	**Decision**	**Comments**
**RCTs**													
Abujilban 2023 [64]	RCT	Y	Y	Y	C	Y						Include	No information was stated about the blinding or not, of the outcome assessor.
Arakawa 2023 [65]	RCT	Y	Y	Y	Y	Y						Include	
Asadzadeh 2020 [66]	RCT	Y	Y	Y	Y	Y						Include	
Boyd 2019 [67]	RCT	Y	Y	Y	C	Y						Include	Also, no information was stated about the blinding or not, of the outcome assessor.
Carona 2023 [68]	RCT	Y	Y	N	C	Y						Include	>20% of enrolled participants were lost to follow-up. No information was stated about the blinding or not, of the outcome assessor.
Chan 2019 [69]	RCT	Y	Y	Y	Y	Y						Include	
Coo 2023 [70]	Mixed-methods; RCT	Y	Y	Y	N	Y						Include	Participants were the outcome assessors and were not blinded to their intervention group, however, there is no suggestion that this non-blinding influences the outcomes measured.
Danaher 2023 [71]	RCT	Y	Y	Y	C	Y						Include	No information was stated about the blinding or not, of the outcome assessor.
Dennis 2020 [72]	RCT	Y	Y	Y	Y	Y						Include	
Gammer 2020 [73]	RCT	Y	Y	Y	C	Y						Include	Assessors were the participants themselves and it was not stated whether the participants knew if they were in the intervention group or not.
Gomà 2023 [55]	RCT	N	N	Y	Y	Y						Exclude	A non-random method was used for allocation sequence (based on date of birth of children) and there were some significant differences between the two groups (education and history of mental illness) further highlighting some problems with the randomisation. For a study claimed to be an RCT, this was considered a significant flaw and thus excluded.
Haga 2019 [74]	RCT	Y	Y	Y	C	Y						Include	Assessors were the participants themselves and it was not stated whether the participants knew if they were in the intervention group or not.
Hassdenteufel 2023 [75]	RCT	Y	Y	N	C	Y						Include	>20% of enrolled participants were lost to follow-up. No information was stated about the blinding or not, of the outcome assessor.
Heller 2020 [76]	RCT	Y	Y	Y	N	Y						Include	Assessors were the participants themselves and it was stated that they knew if they were in the intervention group or not.
Huang 2021 [77]	RCT	Y	Y	Y	Y	Y						Include	
Huh 2023 [78]	RCT	Y	Y	N	Y	Y						Include	>20% attrition rate
Hulsbosch 2023 [79]	RCT	Y	Y	Y	N	Y						Include	Assessors were not blinded.
Jannati 2020 [80]	RCT	Y	Y	Y	N	Y						Include	Assessors were not blinded.
Jiao 2019 [81]	RCT	Y	Y	Y	Y	Y						Include	
Liu 2022 [56]	RCT	C	Y	C	C	C						Exclude	Authors made claim about doing certain things without giving exact terms on how that was then, hence, the records: cannot tell
Loughnan, Butler 2019 [82]	RCT	Y	Y	Y	C	Y						Include	Assessors were the participants themselves and it was not stated whether the participants knew if they were in the intervention group or not.
Loughnan, Sie 2019 [83]	RCT	Y	Y	Y	C	Y						Include	Assessors were the participants themselves and it was not stated whether the participants knew if they were in the intervention group or not.
Merza 2023 [84]	RCT	Y	Y	Y	Y	Y						Include	
Milgrom 2021 [85]	RCT	Y	Y	Y	Y	Y						Include	
Monteiro 2020 [86]	RCT	Y	N	Y	C	Y						Include	Although randomization was performed, some characteristics were significantly different at baseline, between the two groups, but does not seem to suggest a problem with the randomization. And the participants themselves were the assessors of the outcomes and it was not stated if the participants knew whether they were in the intervention or control group.
Naja 2023 [87]	RCT	Y	Y	N	C	Y						Include	The participants themselves were the assessors of the outcomes and it was not stated if the participants knew whether they were in the intervention or control group.
Nishi 2022 [88]	RCT	Y	Y	Y	C	Y						Include	The participants themselves were the assessors of the outcomes and it was not stated if the participants knew whether they were in the intervention or control group.
Qin 2022 [89]	RCT	Y	Y	Y	C	Y						Include	The participants themselves were the assessors of the outcomes and it was not stated if the participants knew whether they were in the intervention or control group.
Sawyer 2019 [90]	RCT	Y	N	Y	N	Y						Include	Some significant differences were noted in the baseline characteristics of both groups. The assessors of the outcomes could not be blinded.
Seo 2022 [91]	RCT	Y	Y	N	Y	Y						Include	>20% attrition rate
Shariatpanahi 2023 [57]	RCT	Y	C	C	C	C						Exclude	Enough information was not provided to ascertain "comparable baseline characteristics" and other bias.
Shorey 2019 [92]	RCT	Y	Y	Y	Y	Y						Include	
Suchan 2022 [93]	RCT	Y	Y	Y	C	Y						Include	Cannot tell who the exact outcome assessors were
Suharwardy 2023 [94]	RCT	Y	Y	Y	C	Y						Include	The participants themselves were the assessors of the outcomes and it was not stated if the participants knew whether they were in the intervention or control group.
Van 2021 [95]	RCT	Y	Y	N	Y	Y						Include	Attrition rate > 20%
Vigod 2021 [96]	RCT	Y	Y	Y	N	Y						Include	The participants themselves were the assessors of the outcomes and they were not blinded to their place in the intervention or control group.
Yang, Jia 2019 [97]	RCT	Y	Y	Y	Y	Y						Include	
Yang, Vigod 2019 [63]	RCT	Y	Y	C	N	Y						Exclude	The number of completers in both arms was not specified in the paper, hence, the paper appears to be intentionally hiding some data. Also, participants self-reported outcomes and they were not blinded.
Zhang 2023 [98]	RCT	Y	Y	Y	Y	Y						Include	
***Non-RCTs***													
Albanese 2022 [61]	Pre-post single group test						Y	Y	Y	C	C	Exclude	The intervention was not clearly described, hence reviewing it was incomprehensive.
Anis 2021 [62]	Case–control						Y	Y	Y	C	C	Exclude	Many parts of the intervention were not clearly explained to be able to determine the full components and quality of the paper.
Avalos 2020 [99]	Mixed-methods; single-arm trial						Y	Y	N	Y	Y	Include	>20% of enrolled participants were lost to follow-up.
Brewington-Brown 2020 [58]	Mixed-methods; single-arm trial						Y	Y	C	Y	Y	Exclude	Exact number of participants pre- and post-intervention were not reported and even the sample size of the reported participants was so small (6 only), hence, questioning the reliability of the paper's quality, especially being an unpublished thesis.
Bryant 2023 [100]	Mixed-methods; single-arm trial						Y	Y	Y	Y	Y	Include	
Buultjens 2023 [101]	Quasi-experimental; double arm						Y	Y	N	Y	Y	Include	The proportion of participants lost to follow-up in the intervention arm was >20%.
DeMairo 2023 [102]	Retrospective cohort						Y	Y	Y	Y	Y	Include	
Gemmill 2022 [103]	Pre-post single group test						Y	Y	C	Y	Y	Include	Missing data were acknowledged at different time points, but the actual population per time point was not stated.
Goetz 2020 [104]	Pre-post single group test						Y	Y	N	C	Y	Include	Attrition rate > 20%
Gong 2021 [105]	Quasi-experimental; double arm						Y	Y	Y	Y	Y	Include	
Kubo 2021 [106]	Pre-post single group test						Y	Y	Y	Y	Y	Include	
Kuipers 2024 [60]	Retrospective cohort^c^						Y	Y	Y	C	C	Exclude	The intervention was not clearly described, hence reviewing it was incomprehensive.
Latendresse 2023 [107]	Pre-post single group test						Y	Y	Y	Y	Y	Include	
Lee 2021 [108]	Quasi-experimental; single arm						Y	Y	Y	Y	Y	Include	
Miles 2023 [109]	Pre-post single group test						Y	Y	N	Y	Y	Include	>20% attrition rate
Shahsavan 2021 [110]	Quasi-experimental; double arm						Y	Y	Y	Y	Y	Include	
Wu 2019 [111]	Quasi-experimental; double arm						Y	Y	Y	Y	Y	Include	
Yang 2023 [59]	Quasi-experimental; double arm^d^						Y	C	C	C	Y	Exclude	There appears to be selective reporting noted in the paper, hence questioning the reliability of some deducted components.

a: Second author added when there are multiple by the same first author within a similar year; b: Withdrawal/dropout tolerance rate used was ≤20%; c: Study was recorded as a nested case–control but actually a retrospective cohort study, as depicted by the described methods; d: Study was recorded as RCT but actually a quasi-experimental study as depicted by their methods and participants allocation flow; Y = Yes; N = No; C = Cannot tell; RCT = Randomized Controlled Trial; 2.1. Is randomization appropriately performed?; 2.2. Are the groups comparable at baseline?; 2.3. Are there complete outcome data?; 2.4. Are outcome assessors blinded to the intervention provided?; 2.5 Did the participants adhere to the assigned intervention?; 3.1. Are the participants representative of the target population?; 3.2. Are measurements appropriate regarding both the outcome and intervention (or exposure)?; 3.3. Are there complete outcome data?; 3.4. Are the confounders accounted for in the design and analysis?; 3.5. During the study period, is the intervention administered (or exposure occurred) as intended?

**Figure 2:**
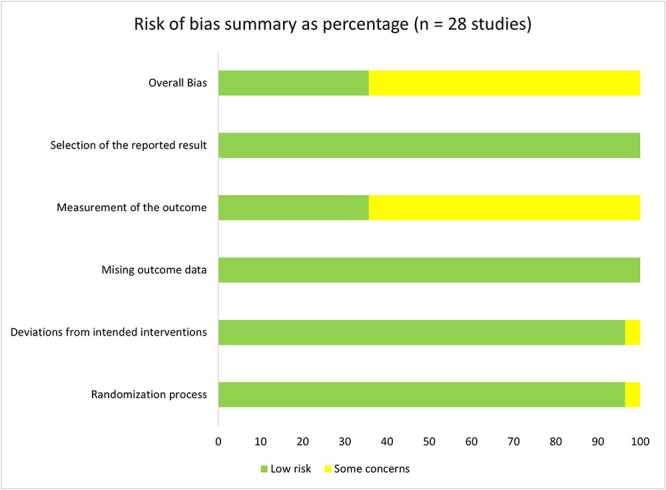
Risk of bias summary of papers included in the meta-analysis.

**Figure 3:**
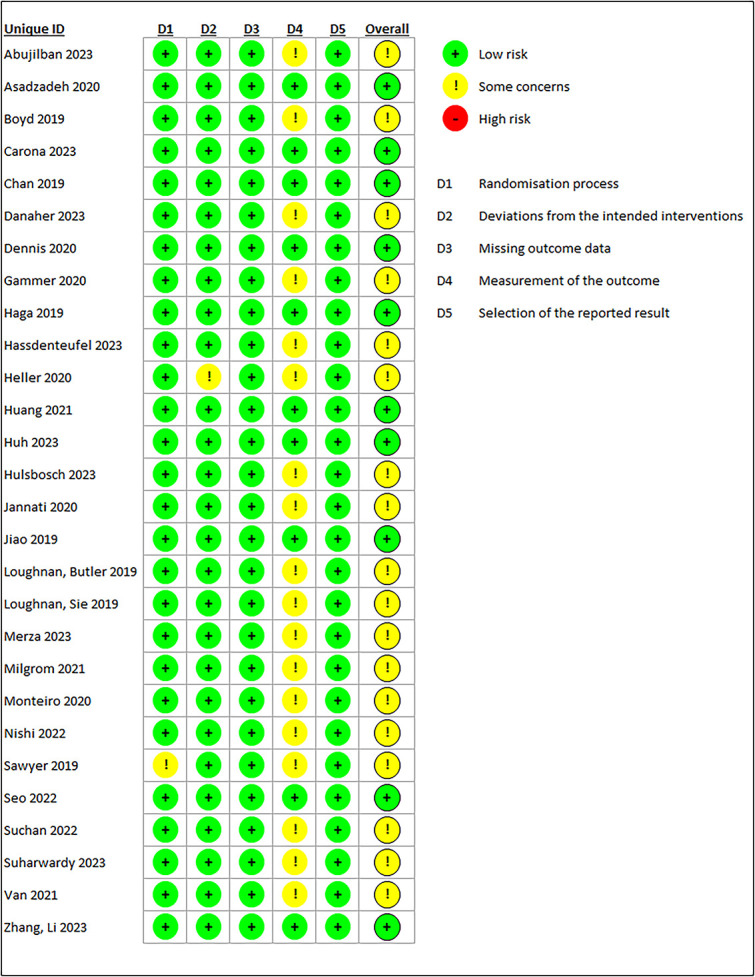
Detailed risk of bias outcome for each paper in the meta-analysis.

The outputs from the risk of bias assessment are presented in Figs 2 and 3 below and the populated report presented in the supplementary file. As seen from Figs 2 and 3, none of the studies included in the meta-analyses had a high risk of bias, possibly because the potential papers that would have contributed to this, have been excluded during the MMAT assessment phase. Furthermore, nearly two-thirds of the studies (64.3%) showed some concerns on the bias from measurement of the outcomes, and this is mostly because of the blinding of the outcome assessors concerns registered in the MMAT assessment above. Only two studies showed some concerns in other domains including bias from randomization process [90] and deviations from the intended interventions [76]. The randomization concern was due to many significant differences between the experiment groups despite appropriate randomization process [90], while the bias from ‘the deviation of intervention from the intended plan’ was contributed by the change in the duration of the intervention from the planned 6 weeks published in the protocol [112] to a minimum completion time of 5 weeks [76].

### Characteristics summary of included studies

**Figure 4:**
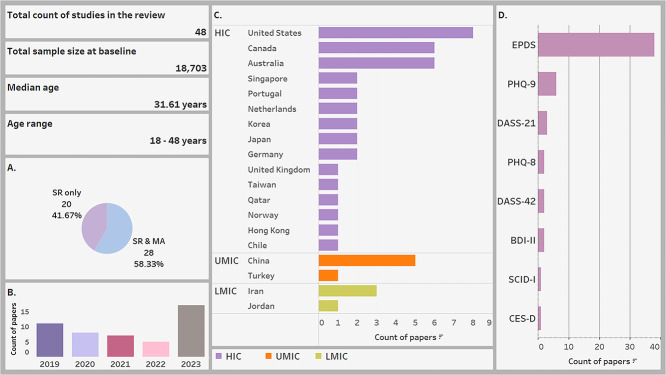
Characteristics of the studies included in this systematic review (a: Role of papers in the review; B: Year of publication; C: Economic region and country of the studies; D: Depression measurement tools; EPDS = Edinburgh postnatal depression scale; PHQ = patient health questionnaire; DASS = depression, anxiety, and stress symptom severity scale; BDI = Beck depression inventory-II; SCID-I = structured clinical interview for DSM-IV; CES-D = Center for Epidemiological Studies Depression scale; SR = systematic review; MA = meta-analysis; HIC = high income countries; UMIC = upper middle income countries; LMIC = low middle income countries)

This review included 48 studies [64–111] which evaluated 18 703 participants. The detailed extracted data are presented in the Supplementary file. Figure 4 below summarizes the characteristics of the included studies showing that most studies were published in 2023 (35.4%) [64, 65, 68, 71, 75, 78, 79, 84, 87, 94, 98, 100–102, 107, 109] (Fig. 4B) and in higher-income countries (HICs) (79.2%) [65, 67–76, 78, 79, 81–88, 90–96, 99–104, 106–108, 111] (Fig. 4C). More than four-fifths (83.3%) [64–66, 68–70, 72, 74–84, 86–96, 98, 100–102, 104, 105, 107, 108, 111] used EPDS as a measurement scale for depressive symptoms while other scales each had less than 25% representative [67, 71–73, 76, 82, 83, 85, 92–94, 97, 99, 103, 106, 109, 110] (Fig. 4D).

### The digital health interventions for managing perinatal depression

**Figure 5:**
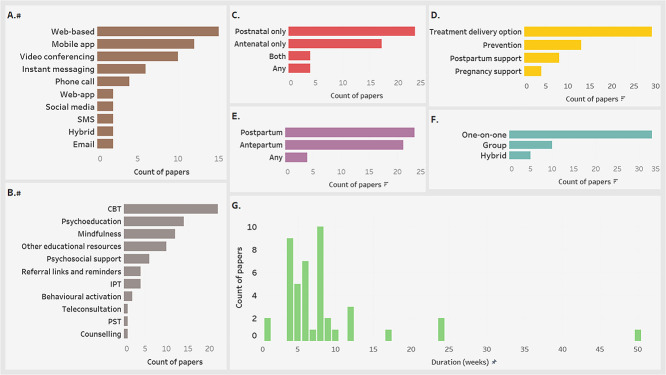
Summary characteristics of the interventions (#: Multiple platforms or components can be applied in one intervention; a: Intervention platforms used; B: Intervention components; C: Period covered intervention; D: Intervention purpose; E: Time of initiation of intervention; F: Intervention delivery mode; G: Duration of the intervention in weeks; CBT = cognitive behavioural therapy; IPT = interpersonal therapy; PST = problem solving therapy)

The studies employed a wide range of digital channels including websites [68, 73, 74, 76, 77, 79, 81–83, 85, 86, 93, 96, 103, 106], dedicated mobile-apps [69, 75, 80, 88, 90, 91, 94, 99, 103, 104, 106, 110], video conferencing [65, 78, 84, 87, 95, 101, 102, 107–109], instant messaging [65, 70, 89, 92, 97, 98], phone calls [64, 66, 72, 92], web-apps [71, 100], social media [67, 111], short messaging services (SMS) [65, 105], and emails [74, 92]; with websites, mobile-apps, and video conferencing being the top-three platforms (Fig. 5-A). Hybrid platforms included interventions that had some physical components within the intervention [66, 101]. Some of the interventions were deployed with multiple digital platforms [65, 74, 92, 103, 106] while others used only one platform [64, 66–73, 75–91, 93–102, 104, 105, 107–111]. Likewise, the intervention component featured various forms of evidence-based management of depression (Fig. 5-B) with many interventions using a mix of the components [67, 70, 77, 82, 83, 85, 88, 90, 91, 94, 100–102, 104, 105, 107, 111], and others maintaining just a single component [64, 66, 68, 69, 71–73, 75, 76, 79–81, 84, 86, 87, 93, 95–99, 108–110].

**Figure 6:**
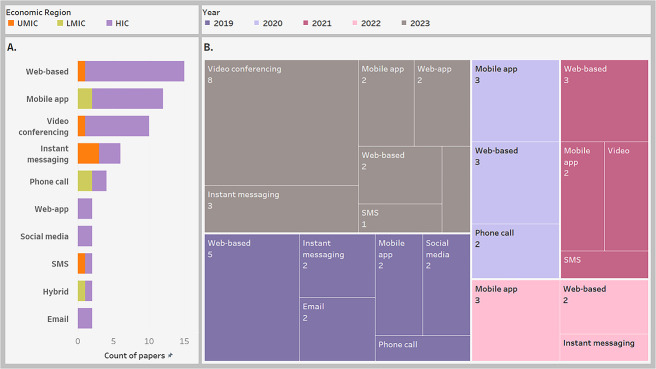
Intervention platforms by economic region and year of publication, multiple possibilities apply as one intervention can have more than one platform (a: Intervention platform by economic region; B: Intervention platform by year of publication; UMIC = upper-middle-income countries; LMIC = lower-middle-income countries; HIC: Higher-income countries)

Although some interventions were initiated during pregnancy [64, 69, 75, 76, 79, 82, 83, 87, 88, 97, 98, 101, 104, 105, 108–111], others were initiated during the postpartum period [66–68, 70, 72, 73, 77, 78, 80–86, 89–91, 93–96, 99], and others, non-specific [71, 100, 102, 107] (Fig. 5-E). Similarly, the periods covered by the intervention varied from the pregnancy period [64, 69, 76, 79, 83, 87, 88, 97, 98, 104, 105, 108–111] to the postpartum period [66–68, 70, 72, 73, 77, 78, 80–82, 84–86, 89–91, 93–96, 99], or both [65, 74, 75, 101], or even non-specific [71, 100, 102, 107] (Fig. 5-C). The intervention purposes were mostly for the direct treatment and prevention of depression [64, 65, 67, 70–76, 78–80, 82–91, 93–99, 103–110] while others were for a secondary influence on depression via offering either pregnancy support or postpartum support [66, 73, 77, 81, 90, 92, 100–102, 109, 111] (Fig. 5-D). Regarding the duration of the interventions, while most of the interventions lasted between 4 and 8 weeks, one intervention was as short as one-day only [95] while another one was as long as 11.5 months [74] (Fig. 5-G).

Some patterns of intervention platforms with respect to delivery and facilitation modes, economic region, and year of publication, were noted. Mobile-apps, phone calls, SMS, websites, emails, and instant messaging were predominantly executed as one-on-one interventions while social media and video conferencing were predominantly group-based (Table 4). Furthermore, the group-based interventions were mostly therapist-facilitated, while the one-on-one interventions were either purely self-guided or a hybrid of self-guidance components and therapist-facilitated components (Table 4).

**Table 4:** Distribution of the various intervention platforms by delivery and facilitation modes

**Platform**	**Delivery mode**			**Facilitation mode**			
	**Group**	**Hybrid**	**One-on-one**	**Hybrid**	**Peer-facilitated**	**Self**	**Therapist-facilitated**
Email			2		1	1	
Hybrid		1	1				2
Instant messaging	1	1	4		1	2	3
Mobile app		1	11	4		8	
Phone call			4		1		3
SMS			2				2
Social media	2				1		1
Video conferencing	6	2	2		1		9
Web-app			2			2	
Web-based	1	1	13	8		6	1

Although the study was dominated by HICs, the few from upper-middle-income countries (UMICs) and low-middle-income countries (LMICs) were mostly used as dedicated mobile-apps, instant messaging, and phone calls (Fig. 6-A). Regarding the year, video conferencing seemed to have a dramatic increase from 2021 to 2023 while web-based interventions decreased over the years (Fig. 6).

### Theories supporting the development of digital health interventions

None of the studies specified any digital health theory considered during the designing or execution of the interventions.

### Effectiveness of digital health interventions for perinatal depression

Table 5 shows the various between-group and within-group mean differences (calculated using equations 2–4) for all the studies that reported depressive symptoms as continuous outcomes. Comments were added for some remarkable observations against the DHI groups.

**Table 5:** Group and time differences in depressive symptom scores

**First author and year** ^**a**^	**Measurement tool**	**Group & Difference**	**Baseline**	**Post-intervention measurement (t2)**	**Most distant follow-up measurement (t3)**	**Within group mean difference 1 (t2 - t1)**	**Within group mean difference 2 (t3 - t1)**	**Within group mean difference 3 (t3 - t2)**	**Comments**
***Double-arm studies***									
Abujilban 2023 [64]	EPDS	Intervention	19.34	8.88		−10.46			
		Control	17.82	16.42		−1.4			
		B/w group difference	1.52	−7.54		−9.06			
Asadzadeh 2020 [66]	EPDS	Intervention	7.52	3.43	1.25	−4.09	−6.27	−2.18	
		Control	7.3	7	4	−0.3	−3.3	−3	
		B/w group difference	0.22	−3.57	−2.75	−3.79	−2.97	−3	
Boyd 2019 [67]	BDI-II	Intervention	29.5	20.2		−9.3			
		Control	23.4	23.3		−0.1			
		B/w group difference	6.1	−3.1		−9.2			
Carona 2023 [68]	EPDS	Intervention	10.99	8.75		−2.24			
		Control	11.73	10.78		−0.95			
		B/w group difference	−0.74	−2.03		−1.29			
Chan 2019 [69]	EPDS	Intervention	7.3	5.3		−2			
		Control	7.2	5.9		−1.3			
		B/w group difference	0.1	−0.6		−0.7			
Coo 2023 [70]	EPDS	Intervention	8.2	8.84		0.64			Control group had better improvement than the DHI group, over time
		Control	8.67	8.63		−0.04			
		B/w group difference	−0.47	0.21		0.68			
Danaher 2023 [71]	PHQ-9	Intervention	10.68	5.78		−4.9			
		Control	10.24	7.48		−2.76			
		B/w group difference	0.44	−1.7		−2.14			
DeMairo 2023 [102]	EPDS	Intervention	18.9	6.86		−12.04			
		Control	17.43	8.57		−8.86			
		B/w group difference	1.47	−1.71		−3.18			
Dennis 2020 [72]	EPDS	Intervention	17.57	7.27	6.79	−10.3	−10.78	−0.48	Although both groups improved, at post-intervention, DHI group was better but lost this at follow-up. Post-intervention = 12 weeks Follow-up = 36 weeks
		Control	17.47	12.4	9.77	−5.07	−7.7	−2.63	
		B/w group difference	0.1	−5.13	−2.98	−5.23	−3.08	2.15	
Gammer 2020 [73]	DASS-21-depression	Intervention	4.77	3.11	2.84	−1.66	−1.93	−0.27	Although both groups improved, at post-intervention, DHI group was better but lost this at follow-up. Post-intervention = 6 weeks Follow-up = 12 weeks
		Control	4.55	4.09	3.49	−0.46	−1.06	−0.6	
		B/w group difference	0.22	−0.98	−0.65	−1.2	−0.87	0.33	
Gong 2021 [105]	EPDS	Intervention	6.2	3.9		−2.3			
		Control	6	5.2		−0.8			
		B/w group difference	0.2	−1.3		−1.5			
Haga 2019 [74]	EPDS	Intervention	6.5	4		−2.5			
		Control	6.2	4.4		−1.8			
		B/w group difference	0.3	−0.4		−0.7			
Hassdenteufel 2023 [75]	EPDS	Intervention	11.8	9.3	7.8	−2.5	−4	−1.5	
		Control	11.7	10.8	9.8	−0.9	−1.9	−1	
		B/w group difference	0.1	−1.5	−2	−1.6	−2.1	−0.5	
Heller 2020 [76]	CES-D	Intervention	28.8	19.5	13.8	−9.3	−15	−5.7	Based on EPDS scale, control group was better at post-intervention but not the same at follow-up. The pattern was also almost the same for the CES-D measurement scale showing some reliability. Post-intervention = 10 weeks Follow-up = 6 weeks postpartum
		Control	27.9	18.6	16.8	−9.3	−11.1	−1.8	
		B/w group difference	0.9	0.9	−3	0.00	−3.9	−3.9	
	EPDS	Intervention	14.3	9.5	8	−4.8	−6.3	−1.5	
		Control	14	8.9	8.7	−5.1	−5.3	−0.2	
		B/w group difference	0.3	0.6	−0.7	0.3	−1	−1.3	
Huang 2021 [77]	EPDS	Intervention	5.05	6.11	5.78	1.06	0.73	−0.33	Compared to baseline, the depressive symptoms worsened in both groups at both post-intervention and follow-up. Also, the change from post-intervention to follow-up time seem to be better in control group than intervention group. Post-intervention = 12 weeks Follow-up = 24 weeks
		Control	5.25	9.22	8.28	3.97	3.03	−17.5	
		B/w group difference	−0.2	−3.11	−2.5	−2.91	−2.3	0.61	
Huh 2023 [78]	EPDS	Intervention	14.6	10.8	10.4	−3.8	−4.2	−0.4	Although both groups improved, at post-intervention, DHI group was better but lost this at follow-up. Post-intervention = 9 weeks Follow-up = 6 months
		Control	14.92	12.86	11.13	−2.06	−3.79	−1.73	
		B/w group difference	−0.32	−2.06	−0.73	−1.74	−0.41	1.33	
Hulsbosch 2023 [79]	EPDS	Intervention	9.5	8.6	8	−0.9	−1.5	−0.6	At post-intervention, control group showed slightly better improvement than intervention group.
		Control	9.2	8.1		−1.1			
		B/w group difference	0.3	0.5		0.2			
Jannati 2020 [80]	EPDS	Intervention	17.42	8.18		−9.24			
		Control	17.39	15.05		−2.34			
		B/w group difference	0.03	−6.87		−6.9			
Jiao 2019 [81]	EPDS	Intervention	7.4	4.73	4.34	−2.67	−3.06	−0.39	At both pst-intervention and follow-up, the control group showed better improvement than intervention group. Post-intervention = 4 weeks Follow-up = 6 months
		Control	9	5.14	5.06	−3.86	−3.94	−0.08	
		B/w group difference	−1.6	−0.41	−0.72	1.19	0.88	−0.31	
Loughnan, Butler 2019 [82]	PHQ-9	Intervention	11.81	6.11	6.32	−5.7	−5.49	0.21	Although both groups improved, at post-intervention, DHI group was better but lost this at follow-up. Post-intervention = 7 weeks Follow-up = 11 weeks
		Control	12.26	10.44	9.52	−1.82	−2.74	−0.92	
		B/w group difference	−0.45	−4.33	−3.2	−3.88	−2.75	1.13	
	EPDS	Intervention	14.91	8.82	8.01	−6.09	−6.9	−0.81	
		Control	15.04	13.34	12.13	−1.7	−2.91	−1.21	
		B/w group difference	−0.13	−4.52	−4.12	−4.39	−3.99	0.4	
Loughnan, Sie 2019 [83]	PHQ-9	Intervention	11.69	7.67	6.75	−4.02	−4.94	−0.92	
		Control	11.05	8.99	8.25	−2.06	−2.8	−0.74	
		B/w group difference	0.64	−1.32	−1.5	−1.96	−2.14	−0.18	
	EPDS	Intervention	13.41	10.01	8.98	−3.4	−4.43	−1.03	At post-intervention, control group showed slightly better improvement than intervention group.
		Control	14.5	10.97	10.62	−3.53	−3.88	−0.35	
		B/w group difference	−1.09	−0.96	−1.64	0.13	−0.55	−0.68	
Merza 2023 [84]	EPDS	Intervention	16.19	10.2		−5.99			
		Control	15.79	13.96		−1.83			
		B/w group difference	0.4	−3.76		−4.16			
Milgrom 2021 [85]	BDI-II	Intervention	28.1	11.63	8.7	−16.47	−19.4	−2.93	
		Control	29.97	18.85	17.41	−11.12	−12.56	−1.44	
		B/w group difference	−1.87	−7.22	−8.71	−5.35	−6.84	−1.49	
Monteiro 2020 [86]	EPDS	Intervention	6.38	5.26		−1.12			
		Control	6.72	6.19		−0.53			
		B/w group difference	−0.34	−0.93		−0.59			
Naja 2023 [87]	EPDS	Intervention	8.7	8.3		−0.4			At post-intervention, control group showed better improvement than intervention group.
		Control	11	9.4		−1.6			
		B/w group difference	−2.3	−1.1		1.2			
Nishi 2022 [88]	EPDS	Intervention	5.26	5.55	5.21	0.29	−0.05	−0.34	Both groups showed worsening symptoms post-intervention, buit the DHI group seem to have recovered some extent at follow-up. Post-intervention = 32 weeks gestation Follow-up = 3 months postpartum
		Control	5.07	5.41	5.16	0.34	0.09	−0.25	
		B/w group difference	0.19	0.14	0.05	−0.05	−0.14	−0.09	
Qin 2022 [89]	EPDS	Intervention	4.58	2.71		−1.87			
		Control	5.42	4.55		−0.87			
		B/w group difference	−0.84	−1.84		−1			
Sawyer 2019 [90]	EPDS	Intervention	8.8	7.9	8.6	−0.9	−0.2	0.7	Intervention group showed some worsening depressive symptoms at follow-up and compared to the controls, this was tangible. Post-intervention = 6 months Follow-up = 10 months
		Control	9.5	8.7	7	−0.8	−2.5	−1.7	
		B/w group difference	−0.7	−0.8	1.6	−0.1	2.3	2.4	
Seo 2022 [91]	EPDS	Intervention	13.95	10.7	9.84	−3.25	−4.11	−0.86	Although both groups improved, at post-intervention, DHI group was better but lost this at follow-up. Post-intervention = 8 weeks Follow-up = 5 months
		Control	15.03	13.03	11.47	−2	−3.56	−1.56	
		B/w group difference	−1.08	−2.33	−1.63	−1.25	−0.55	0.7	
Shahsavan 2021 [110]	DASS-42-depression	Intervention	11.43	10.4		−1.03			At post-intervention, control group showed better improvement than intervention group.
		Control	15.53	11.53		−4			
		B/w group difference	−4.1	−1.13		2.97			
Suchan 2022 [93]	DASS-21-depression	Intervention	24.14	13.03	9.91	−11.11	−14.23	−3.12	
		Control	26.25	20.24		−6.01			
		B/w group difference	−2.11	−7.21		−5.1			
	EPDS	Intervention	14.47	9.54	7.86	−4.93	−6.61	−1.68	
		Control	15.44	12.35		−3.09			
		B/w group difference	−0.97	−2.81		−1.84			
Suharwardy 2023 [94]	PHQ-9	Intervention	4.41	3.09		−1.32			
		Control	3.36	3.23		−0.13			
		B/w group difference	1.05	−0.14		−1.19			
	EPDS	Intervention	5.51	4.88		−0.63			At post-intervention, control group showed slightly better improvement than intervention group.
		Control	5.37	4.61		−0.76			
		B/w group difference	0.14	0.27		0.13			
Van 2021 [95]	EPDS	Intervention	16.47	11.65		−4.82			
		Control	15.92	14.04		−1.88			
		B/w group difference	0.55	−2.39		−2.94			
Vigod 2021 [96]	EPDS	Intervention	14.5	11.3		−3.2			
		Control	15	12		−3			
		B/w group difference	−0.5	−0.7		−0.2			
Yang 2019 [97]	PHQ-9	Intervention	5.98	3.58		−2.4			Depressive symptoms in control group worsened
		Control	5.72	6.26		0.54			
		B/w group difference	0.26	−2.68		−2.94			
Zhang 2023 [98]	EPDS	Intervention	8.91	5.21	5.54	−3.7	−3.37	0.33	Both DHI and control group had slightly worsening symptoms at follow-up. Post-intervention = 8 weeks Follow-up = 6 months postpartum
		Control	9.43	7.86	8.45	−1.57	−0.98	0.59	
		B/w group difference	−0.52	−2.65	−2.91	−2.13	−2.39	−0.26	
***Single-arm studies***									
Avalos 2020 [99]	PHQ-8	Intervention cohort	15.2	11.4		−3.8			
Bryant 2023 [100]	EPDS	Intervention cohort	8.94	8.7		−0.24			
Gemmill 2022 [103]	DASS-42-depression	Intervention cohort	14.43	6.17		−8.26			
Goetz 2020 [104]	EPDS	Intervention cohort	8.41	8.62		0.21			The cohort showed slightly worsening depressive symptoms at post-intervention
Kubo 2021 [106]	PHQ-8	Intervention cohort	13.5	7.5		−6			
Latendresse 2023 [107]	EPDS	Intervention cohort	14	9.5	9.9	−4.5	−4.1	0.4	The cohort showed slightly worsening depressive symptoms at follow-up
Lee 2021 [108]	EPDS	Intervention cohort	14.38	6.3		−8.08			
Miles 2023 [109]	DASS-42-depression	Intervention cohort	7	5.78		−1.22			

a: Second author added when there are multiple papers by the same first author within a similar year; BDI-II: Beck Depression Inventory-II; CES-D = Center for Epidemiological Studies Depression scale; DASS-21 = 7-item Depression, anxiety, and stress symptom severity scale; DASS-42 = 14-item Depression, anxiety, and stress symptom severity scale; DHI = - Digital Health Intervention; EPDS = Edinburgh Postpartum Depression Scale; PHQ = Patient Health Questionnaire

Table 6 shows the computed data used in conducting the meta-analysis; Equations 1 and 5–8 were used in computing the data.

**Table 6:** Computed data used for meta-analysis

**First author and year** ^**a**^	**Scale**	**Ne**	**Nc**	**Ntot**	***P*-value**	**Mean diff.**	**t statistic**	**SE**	**SD**	**SMD**	**RoB**
Abujilban 2023 [64]	EPDS	50	50	100	0.001	−9.06	3.392588114	−2.670527543	−13.35263771	0.6785176	Some concerns
Asadzadeh 2020 [66]	EPDS	44	43	87	0.0001	−3.79	4.083297521	−0.928171406	−4.328417305	0.8756087	Low
Boyd 2019 [67]	BDI-II	8	12	20	0.001	−9.2	3.921645825	−2.345953819	−5.139727302	1.7899782	Some concerns
Carona 2023 [68]	EPDS	542	511	1053	0.001	−1.29	3.299808029	−0.390931833	−6.340112193	0.2034664	Low
Chan 2019 [69]	EPDS	330	330	660	0.049	−0.7	1.972244811	−0.354925512	−4.559100755	0.1535391	Low
Danaher 2023 [71]	PHQ-9	96	95	191	0.003	−2.14	3.00670235	−0.711743216	−4.918176064	0.4351207	Some concerns
Dennis 2020 [72]	EPDS	104	100	204	0.001	−5.23	3.339340666	−1.566177435	−11.18259377	0.4676911	Low
Gammer 2020 [73]	DASS-21	54	80	134	0.33	−1.2	0.977722657	−1.227341917	−6.968752509	0.1721972	Some concerns
Haga 2019 [74]	EPDS	528	589	1117	0.008	−0.7	2.656854863	−0.263469416	−4.396207992	0.1592281	Low
Hassdenteufel 2023 [75]	EPDS	230	230	460	0.086	−1.6	1.720593656	−0.9299116	−9.972190944	0.1604462	Some concerns
Heller 2020 [76]	EPDS	79	80	159	0.1	0.3	1.654617035	0.181310837	1.143098725	0.2624445	Some concerns
Huang 2021 [77]	EPDS	18	18	36	0.007	−2.91	2.870641288	−1.013710773	−3.04113232	0.9568804	Low
Huh 2023 [78]	EPDS	71	65	136	0.04	−1.74	2.073933212	−0.838985552	−4.887321179	0.3560233	Low
Hulsbosch 2023 [79]	EPDS	110	110	220	0.86	0.2	0.176582855	1.132612786	8.399681233	0.0238104	Some concerns
Jannati 2020 [80]	EPDS	38	37	75	0.001	−6.9	3.42885415	−2.012334062	−8.712887511	0.7919303	Some concerns
Jiao 2019 [81]	EPDS	68	68	136	0.857	1.19	0.180541807	6.591271121	38.43338483	0.0309627	Low
Loughnan, Butler 2019 [82]	EPDS	65	55	120	0.001	−4.39	3.374891682	−1.300782488	−7.099897545	0.6183188	Some concerns
Loughnan, Sie 2019 [83]	EPDS	36	41	77	0.84	0.13	0.202595239	0.641673518	2.809389355	0.0462734	Some concerns
Merza 2023 [84]	EPDS	77	67	144	0.001	−4.16	3.360360751	−1.237962323	−7.409841956	0.5614155	Some concerns
Milgrom 2021 [85]	BDI-II	39	38	77	0.01	−5.35	2.642983067	−2.024227876	−8.880514762	0.6024426	Some concerns
Monteiro 2020 [86]	EPDS	104	145	249	0.194	−0.59	1.302378789	−0.453017206	−3.52545921	0.1673541	Some concerns
Nishi 2022 [88]	EPDS	2509	2508	5017	0.608	−0.05	0.512962709	−0.097472972	−3.45204347	0.0144842	Some concerns
Sawyer 2019 [90]	EPDS	70	61	131	0.001	−0.1	3.367546457	−0.02969521	−0.169537078	0.5898415	Some concerns
Seo 2022 [91]	EPDS	37	36	73	0.278	−1.25	1.093202969	−1.143429021	−4.88427258	0.2559235	Low
Suchan 2022 [93]	EPDS	25	29	54	0.13	−1.84	1.538458372	−1.196002462	−4.382321111	0.4198688	Some concerns
Suharwardy 2023 [94]	EPDS	84	68	152	0.87	0.13	0.163938814	0.792978774	4.861092307	0.026743	Some concerns
Van 2021 [95]	EPDS	161	201	362	0.001	−2.94	3.317756549	−0.886140968	−8.378369253	0.3509036	Some concerns
Zhang 2023 [98]	EPDS	80	80	160	0.001	−2.13	3.353167742	−0.635220235	−4.017485515	0.5301824	Low

a: Second author added when there are multiple papers by the same first author within a similar year; BDI-II: Beck Depression Inventory-II; DASS-21 = 7-item Depression, anxiety, and stress symptom severity scale; EPDS = Edinburgh Postpartum Depression Scale; Ne = Sample size of intervention group (Mediterranean diet group); Nc = Sample size of control group; Ntot = Total sample size; Mean diff. = Between-within mean difference (mean difference between the two test groups over time); PHQ-9 = Patient Health Questionnaire-9; SE = standard error; SD = standard deviation; SMD = standardized mean difference; RoB = Risk of bias.

**Figure 7:**
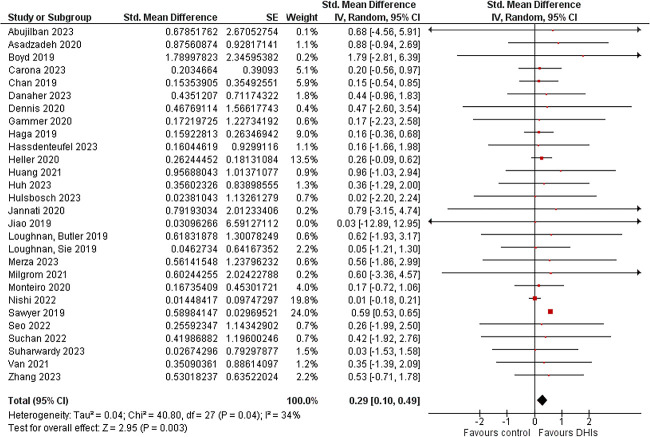
Forest plot of the meta-analysis comparing the effectiveness of DHIs against controls in reducing depressive symptoms.

The pooled data from the 28 studies used for meta-analyses [64, 66–68, 71–86, 88, 90, 91, 93–95, 98] comprised 5567 participants in the DHI group and 5747 control participants. The forest plot (Fig. 7) showed a relatively homogenous pattern with a significant small-sized random-effect SMD in favour of the DHIs against the controls (SMD = 0.29; 95% confidence interval (CI) = 0.10–0.49, *P* = 0.003). Furthermore, the I^2^ statistic only showed a low level of heterogeneity.

As planned, sensitivity analysis was conducted to confirm the validity of the meta-analytic outcome in the situation of some modifications, and the findings presented in Table 7 showed no changes in the outcome of the meta-analysis.

**Table 7:** Findings from sensitivity analysis

**Condition**	**Ne**	**Nc**	**Pooled SMD (95% CI)**	**Heterogeneity** **I**^**2**^**, Chi**^**2**^ **(*P*-value)**	**Pooled effect (*P*-value)**
All studies for MA	5657	5747	0.29 (0.10, 0.49)	34%, 40.80 (0.04)^*^	2.95 (0.003)^*^
Pilot studies [67, 77, 86, 93, 94], excluded	5418	5475	0.29 (0.07, 0.51)	44%, 39.31 (0.01)^*^	2.58 (0.010)^*^
Studies with total sample size less than 100 [66,67,77,80,83,85,91,93], excluded	5412	5413	0.27 (0.04, 0.50)	52%, 39.55 (0.004)^*^	2.33 (0.020)^*^
Intervention with wide SMD CI [81], excluded	5589	5679	0.29 (0.09, 0.49)	36%, 40.80 (0.03)^*^	2.88 (0.004)^*^
Studies reporting depression measurements with non-EPDS scales [67, 71, 73, 85], excluded	5460	5522	0.28 (0.07, 0.50)	43%, 40.41 (0.01)^*^	2.63 (0.009)^*^
Studies with hybrid intervention platforms [66], excluded	5613	5704	0.29 (0.09, 0.49)	36%, 40.66 (0.03)^*^	2.80 (0.005)^*^

^*^= significant finding; Ne = sample size of intervention group; Nc = sample size of control group; CI = confidence interval; I2 = heterogeneity index; SMD = Standardised mean difference; EPDS = Edinburgh Postnatal Depression Scale; MA = Meta-analysis

### Sociodemographic and methodological differences influencing the effectiveness of the DHIs

Subgroup analyses were conducted to determine possible differences based on specified categories. The findings are presented in Table 8 below and the forest plots are presented in the Supplementary file. Subgroups with only one representative study in the group were excluded as follows: for intervention platform, instant messaging [98], social media [67], hybrid [66], and email [74] were excluded while web-app was recategorized as web-based platform; peer facilitation [84] was excluded from facilitation mode group, and ‘any’ was excluded from both timing of intervention [71] and period covered by intervention [71].

**Table 8:** Findings from subgroup analysis

**Category**	**Subgroup**	**Number of studies**	**Ne**	**Nc**	**Group's pooled SMD (95% CI)**	**Group's heterogeneity** **I**^**2**^**, Chi**^**2**^ **(*P*-value)**	**Group's pooled effect (*P*-value)**	**Subgroup differences X**^**2**^ **(*P*-value)**
Economic region	HIC	23	5427	5519	0.27 (0.05, 0.48)	46%, 40.45 (0.010)	2.38 (0.02)^*^	0.97 (0.61)
	UMIC	2	98	98	0.65 (−0.40, 1.71)	0%, 0.13 (0.72)	1.21 (0.23)	
	LIC	3	132	130	0.84 (−0.73, 2.42)	0%, 0.01 (1.00)	1.05 (0.29)	
Intervention platform	Mobile application	7	3298	3270	0.26 (−0.15, 0.68)	82%, 33.72 (<0.00001)^*^^*^	1.23 (0.22)	0.09 (0.99)
	Phone call	2	154	150	0.52 (−2.13, 3.17)	0%, 0.00 (0.95)	0.39 (0.70)	
	Video conferencing	3	309	333	0.39 (−0.68, 1.47)	0%, 0.02 (0.99)	0.72 (0.47)	
	Web based	12	1236	1270	0.26 (−0.02, 0.53)	0%, 0.88 (1.00)	1.83 (0.07)	
Intervention delivery mode	Individual	22	5202	5273	0.11 (−0.03, 0.26)	0%, 4.35 (1.00)	1.56 (0.12)	36.09 (<0.00001)^*^
	Group	4	317	345	0.47 (−0.58, 1.51)	0%, 0.36 (0.95)	0.88 (0.38)	
	Hybrid	2	138	129	0.59 (0.53, 0.65)	0%, 0.01 (0.93)	19.86 (<0.00001)^*^	
Intervention facilitation mode	Self	13	4105	4167	0.07 (−0.10, 0.23)	0%, 2.40 (1.00)	0.79 (0.43)	32.70 (<0.00001)^*^
	Therapist	6	438	471	0.56 (−0.35, 1.48)	0%, 0.51 (0.99)	1.21 (0.23)	
	Mixed	8	1037	1042	0.58 (0.52, 0.63)	0%, 5.19 (0.64)	19.80 (<0.00001)^*^	
Time of intervention initiation	Antepartum	9	3952	1718	0.09 (−0.06, 0.24)	0%, 2.15 (0.98)	1.15 (0.25)	35.44 (<0.00001)^*^
	Postpartum	18	1609	1634	0.58 (0.53, 0.64)	0%, 3.20 (1.00)	19.88 (<0.00001)^*^	
Period covered by intervention	Antepartum	7	3194	3199	0.08 (−0.08, 0.24)	0%, 2.06 (0.91)	1.00 (0.32)	35.52 (<0.00001)^*^
	Postpartum	18	1609	1634	0.58 (0.53, 0.64)	0%, 3.20 (1.00)	19.88 (<0.00001)^*^	
	Both	2	758	819	0.16 (−0.34, 0.66)	0%, 0.00 (1.00)	0.63 (0.53)	
Duration of intervention	1–4 weeks	5	162	161	0.84 (−0.86, 2.54)	0%, 0.02 (0.99)	0.97 (0.33)	3.24 (0.20)
	5–12 weeks	20	4369	4364	0.11 (−0.04, 0.26)	0%, 4.30 (1.00)	1.39 (0.16)	
	>12 weeks	3	928	9890	0.41 (0.08, 0.74)	51% 4.10 (0.13)	2.43 (0.01)	

^*^ = significant finding; Ne = sample size of experiment group; Nc = sample size of control group; CI = confidence interval; I^2^ = heterogeneity index; X^2^ = Chi-square; HIC = High-Income country; UMIC = Upper-Middle-Income Country; LMIC = Lower-Middle-Income Country

Table 8 above showed that intervention delivery mode, intervention facilitation mode, time of initiating the intervention, and period covered by the intervention, all significantly influenced the effectiveness of DHIs in perinatal depression (*P* < 0.00001) in favour of the DHIs against the controls, with the subgroups contributing to these differences, respectively, being hybrid delivery mode, mixed facilitation mode, initiating intervention in postpartum period, and intervention covering only the postpartum period. Nonetheless, the significant effect contributed by the hybrid subgroup in intervention delivery mode is not certain because there are few studies in the subgroup (n = 2) with the significance lost when the subgroup is excluded (*P* = 0.51); however, this was not noticed for the ‘period covered by intervention’ group since the *P*-value was retained when the subgroup with only two studies (‘both’) was excluded.

For the economic region, although no significance was noted between the subgroups, this is uncertain because of the non-uniform distribution of studies among the groups with the HIC subgroup having >10 000 participants and the other subgroups having < 300 participants per subgroup. Similarly, the non-significance difference in the intervention platform group and duration of the intervention had some subgroups with few representative studies, however, these were inconsequential since the pooled subgroup effect remained insignificant (*P* > 0.05) when the small groups were removed both sequentially and collectively.

## DISCUSSION

This review used good-quality empirical quantitative studies to evaluate the effectiveness of DHIs in reducing PND symptoms. The review found a significant small positive effect of DHIs over controls (including treatment as usual and waiting list) and this finding is dependable since sensitivity analysis showed that the outcome was not influenced by pilot studies, over-estimation of effects from small sample size, diverse measurement scales, wide confidence interval, and hybrid intervention platforms (digital and physical components). This effectiveness finding is similar to previous reviews [16–22] and an addition to the evidence; it also indicates that DHIs can be used as adjuncts or alternatives to traditional methods for preventing or managing perinatal depression.

The age range of the participants (18–48 years) represent the typical age of fertility of women (15–49 years) [113], however, no study with adolescent representatives was included, hence limiting the context and interpretations of these findings to only adult populations, as seen in other previous reviews [16–24, 27–34, 41]. Additionally, it is well known that sociodemographic characteristics influence the acceptance and utilization of digital tools [9], hence, it is essential that studies include a wide array of sociodemographic profiles of participants involved in the evaluation of DHIs.

Similar to other reviews [16, 23, 29], most studies that contribute to this review were from HICs, closely followed by the UMICs; this pattern may be explained by countries’ economic capacity and political willingness to support digital infrastructure [114, 115]. Likewise, the publication year pattern showed a gradual decline from 2019 to 2022 and a sudden spike in 2023, which may have been influenced by the COVID-19 pandemic on both the volume of research activities in the respective years and the adoption of digital health services [116].

The array of digital tools employed in this review is wide and covers most mainstream digital platforms particularly websites, mobile apps, video conferencing, instant messaging, and phone calls. Although this finding is similar to other studies [19, 31, 34], a particular trend that is established in this review is the declining implementation of website interventions and the increasing choice of video conferencing as the year advances. This drifting away from website DHIs may be explained by poor adherence rates recorded in previous website-based interventions [18, 21, 27], while the increase of video conferencing may be due to its ease of use by the patients [117, 118]. Additionally, the increasing use of video conferences may also be a result of preference of group interventions over one-on-one interventions as this was the predominant delivery modes for video conferencing in this review compared to websites which are mostly one-on-one. However, subgroup meta-analysis between individual and group delivery modes was not significant. Nonetheless, this may not be completely dependable as the ratio of papers in both groups was highly imbalanced as 11:2, respectively.

In contrast to other reviews [19, 23], the duration of the intervention did not show any significant influence on the outcome of the effects. Nonetheless, this may not be the complete situation because the post-intervention measures used for meta-analysis in this review were the immediate post-intervention measurements and none of the follow-ups. While there is no demonstrated difference in the remission rates herein, in the long run, the duration of intervention may be protective against relapse of symptoms [19].

The DHIs in this review were designed to serve various purposes including the direct treatment and prevention of depression and an indirect influence on depression via the offering of pregnancy and/or postpartum support. Particularly, the intervention facilitation mode, timing of initiation, and period covered by the intervention, all influenced the outcome of the intervention. While this is not readily comparable to other studies because they were focused on only antepartum or postpartum depression, the findings, which were mostly in favour of postpartum initiation and coverage may be due to the wider research attention given to postpartum over antepartum occurrences [25, 26].

Predictably, none of the studies, both in their full publications and available protocols, stipulated the use of any digital-health-related theory for designing the interventions, despite stating the use of various psychological theories for the intervention components. This negligence of DHI theories may contribute to the high attrition and poor adherence rates noted in previous similar reviews [21]. This negligence may also hinder the scalability of these interventions because the outcomes of DHIs are highly dependent on the interplays of complex interactions between the technical system, the users, and the social context, often referred to as human-computer-interaction or socio-technological frameworks [9, 119–122].

### Strengths and limitations

While this review adhered to best practice, it is not without limitations. Firstly, while studies of good quality were used for the review, most interpretations of effectiveness in the discussion were from only the meta-analyses and not all the 48 papers in the reviews. Furthermore, due to limited time and scope of the review, further meta-analyses explorations could not be performed; these include analysis with single-arm studies, non-randomized dual arm studies, and analysis of various follow-up and measurement time points. Lastly, while the aim of reducing this review to only 5 years was for manageability and containment of obsolescence in DHIs, vital information may have been lost in potentially eligible studies that are not yet so old.

## CONCLUSION

DHIs may help bridge the barriers in managing PND by offering digital tools as alternative delivery channels of management. However, contemporary interventions do not seem to consider relevant digital health theories in their designs thereby raising concerns about acceptability and scalability. While existing reviews are solely focused on either antepartum or postpartum depression, this review offers a more comprehensive perspective by offering insight on PND. Future research in this direction will help to understand the pattern of response to DHIs, given that both antepartum and postpartum depressions are related.

## Supplementary Material

Supplementary_file_oqae026

## Data Availability

The data underlying this article are available in the article and in its online supplementary material.
