# Supplementary material for: Effectiveness of digital health interventions for perinatal depression: a systematic review and meta-analysis
Source: Oxf Open Digit Health. 2024 Aug 3;2:oqae026. doi: 10.1093/oodh/oqae026 (PMC11998592; doi:10.1093/oodh/oqae026)

**Effectiveness of digital health interventions for perinatal depression: A systematic review and meta-analysis**

Dr Ifunanya Stellamaris Anyanwu^1^, Judy Jenkins^1^

^1^Department of Health Informatics, Faculty of Medicine, Health, and Life Sciences, Swansea University, Swansea, Wales, United Kingdom

**Corresponding author:** Ifunanya Stellamaris Anyanwu, [2235284@swansea.ac.uk](mailto:2235284@swansea.ac.uk), Data Science Building, Faculty of Medicine, Health, and Life Sciences, Swansea University, Singleton Park, Swansea, SA2 8PP, Wales, United Kingdom

# DETAILED SEARCH STRATEGY FOR EACH DATABASE

## MEDLINE – 24^th^ January 2024

| **ID** | **DETAILS** | **NO.** | **Comment** |
| --- | --- | --- | --- |
| S1 | TI ( pregnan* or perinatal OR “peri-natal” OR prepartum OR “pre-partum” OR antenatal OR “ante-natal” OR birth* OR childbirth OR “child-birth” OR “child birth” OR postnatal OR “post-natal” OR postpartum OR “post-partum” OR peripartum OR “peri-partum” OR antepartum OR “ante-partum” OR prenatal OR “pre-natal” OR matern* OR "after birth" OR puerper* OR obstetric* OR mother* OR mums OR mum OR moms OR mom OR prepartal OR “pre-partal” OR childbearing ) OR AB ( pregnan* or perinatal OR “peri-natal” OR prepartum OR “pre-partum” OR antenatal OR “ante-natal” OR birth* OR childbirth OR “child-birth” OR “child birth” OR postnatal OR “post-natal” OR postpartum OR “post-partum” OR peripartum OR “peri-partum” OR antepartum OR “ante-partum” OR prenatal OR “pre-natal” OR matern* OR "after birth" OR puerper* OR obstetric* OR mother* OR mums OR mum OR moms OR mom OR prepartal OR “pre-partal” OR childbearing ) | 1,382,507 | Title and abstract search of pregnancy keywords |
| S2 | TI ( mental* OR depress* OR “well-being” OR “well being” OR wellbeing OR "baby blues" OR distress* OR stress* OR psychological* OR psychiatric ) OR AB ( mental* OR depress* OR “well-being” OR “well being” OR wellbeing OR "baby blues" OR distress* OR stress* OR psychological* OR psychiatric ) | 2,401,549 | Title and abstract search of depression keywords |
| S3 | S1 AND S2 | 151,156 | Combining previous search results for perinatal depression |
| S4 | (MH "Depression, Postpartum") | 7,683 | MeSH search of postpartum depression |
| S5 | S3 OR S4 | 151,741 | Combining MeSH search with Title and abstract searches |
| S6 | TI ( digital OR “digital health” OR telehealth OR phone* OR “cell-phone” OR cellphone OR “cell phone” OR telephone OR “mobile-phone” OR smartphone* OR “smart-phone” OR telemedic* OR telecare OR telesupport OR “tele-health” OR “tele-medicine” OR “tele-care” OR “tele-support” OR wearable* OR mobile OR mhealth OR m-health OR web* OR “web-based” OR “web-guided” OR “web-supported” OR “web-delivered” OR “web-assisted” OR “web-aided” OR “web-facilitated” OR “web portal” OR app OR apps OR application* OR “mobile apps” OR “mobile app” OR “app-based” OR “web-app” OR “web-apps” OR “social media” OR facebook OR twitter OR ehealth OR “e-health” OR internet OR “internet-based” OR “internet-guided” OR “internet-supported” OR “internet-delivered” OR “internet-assisted” OR “internet-aided” OR “internet-facilitated” OR online OR “on-line” OR “online-based” OR “online-guided” OR “online-supported” OR “online-delivered” OR “online-assisted” OR “online-aided” OR “online-facilitated” OR computer* OR “computer-based” OR “computer-guided” OR “computer-supported” OR “computer-delivered” OR “computer-assisted” OR “computer-aided” OR “computer-facilitated or SMS OR “text-based” OR “text based” OR “self-help” OR “self help” OR “self-guided” OR “self guided” OR “self-directed” OR “self directed” OR “self direct” OR “self-direct” OR telecommunication* OR “e-therapy” OR “e-mental” OR teletherapy OR telemental OR technolog* OR “technology-assisted” OR virtual OR cyber OR cyberpsychology OR “cyber psychology” OR cybertherapy OR iCBT OR cCBT OR eCBT or mCBT OR electronic* OR software OR telepsychiatry OR “instant messaging” OR “e-learning” ) OR AB ( digital OR “digital health” OR telehealth OR phone* OR “cell-phone” OR cellphone OR “cell phone” OR telephone OR “mobile-phone” OR smartphone* OR “smart-phone” OR telemedic* OR telecare OR telesupport OR “tele-health” OR “tele-medicine” OR “tele-care” OR “tele-support” OR wearable* OR mobile OR mhealth OR m-health OR web* OR “web-based” OR “web-guided” OR “web-supported” OR “web-delivered” OR “web-assisted” OR “web-aided” OR “web-facilitated” OR “web portal” OR app OR apps OR application* OR “mobile apps” OR “mobile app” OR “app-based” OR “web-app” OR “web-apps” OR “social media” OR facebook OR twitter OR ehealth OR “e-health” OR internet OR “internet-based” OR “internet-guided” OR “internet-supported” OR “internet-delivered” OR “internet-assisted” OR “internet-aided” OR “internet-facilitated” OR online OR “on-line” OR “online-based” OR “online-guided” OR “online-supported” OR “online-delivered” OR “online-assisted” OR “online-aided” OR “online-facilitated” OR computer* OR “computer-based” OR “computer-guided” OR “computer-supported” OR “computer-delivered” OR “computer-assisted” OR “computer-aided” OR “computer-facilitated or SMS OR “text-based” OR “text based” OR “self-help” OR “self help” OR “self-guided” OR “self guided” OR “self-directed” OR “self directed” OR “self direct” OR “self-direct” OR telecommunication* OR “e-therapy” OR “e-mental” OR teletherapy OR telemental OR technolog* OR “technology-assisted” OR virtual OR cyber OR cyberpsychology OR “cyber psychology” OR cybertherapy OR iCBT OR cCBT OR eCBT or mCBT OR electronic* OR software OR telepsychiatry OR “instant messaging” OR “e-learning” ) | 2,933,195 | Title and abstract search of digital health keywords |
| S7 | (MH "Digital Health") OR (MH "Mental Health Teletherapy") OR (MH "Distance Counseling") | 134 | MeSH search of digital health related keywords |
| S8 | S6 OR S7 | 2,933,217 | Combining digital health keywords |
| S9 | TI ( trial* OR cohort OR prospective OR retrospective OR “case-control” OR “case control” OR “quasi-experimental” OR “quasi experimental” OR intervention OR “user experience” OR “user activity” OR “user log” OR longitudinal OR efficacy OR effectiveness OR evaluat* OR comparative OR “before and after” OR “pre-post” ) NOT TI ( systematic OR review OR scoping OR protocol OR qualitative OR "focus group" OR "thematic analysis" OR "meta-analysis" OR predict* ) | 1,970,833 | Title search of desired study types while excluding unwanted study types reflected in titles |
| S10 | AB ( trial* OR cohort OR prospective OR retrospective OR “case-control” OR “case control” OR “quasi-experimental” OR “quasi experimental” OR intervention OR “user experience” OR “user activity” OR “user log” OR longitudinal OR efficacy OR effectiveness OR evaluat* OR comparative OR “before and after” OR “pre-post” ) NOT TI ( systematic OR review OR scoping OR protocol OR qualitative OR "focus group" OR "thematic analysis" OR "meta-analysis" OR predict* ) | 7,293,821 | Abstract search of desired study types while excluding unwanted study types reflected in titles |
| S11 | S9 OR S10 | 7,964,818 | Combining search of desired study types |
| S12 | S5 AND S8 AND S11 | 4,685 | Combining all searches |
| S13 | S5 AND S8 AND S11  Limiters - Publication Date: 20140101-20241231 | 3,342 | Year limited to between 2014 to current |
| S14 | S5 AND S8 AND S11  Limiters - Publication Date: 20140101-20241231; Narrow by Language: - english | 3,271 | Language limited to English only |
|  | Title screened | 360 |  |

## BUSINESS SOURCE COMPLETE – 25^th^ January 2024

| **ID** | **DETAILS** | **NO.** | **COMMENT** |
| --- | --- | --- | --- |
| S1 | TI ( pregnan* or perinatal OR “peri-natal” OR prepartum OR “pre-partum” OR antenatal OR “ante-natal” OR birth* OR childbirth OR “child-birth” OR “child birth” OR postnatal OR “post-natal” OR postpartum OR “post-partum” OR peripartum OR “peri-partum” OR antepartum OR “ante-partum” OR prenatal OR “pre-natal” OR matern* OR "after birth" OR puerper* OR obstetric* OR mother* OR mums OR mum OR moms OR mom OR prepartal OR “pre-partal” OR childbearing ) OR AB ( pregnan* or perinatal OR “peri-natal” OR prepartum OR “pre-partum” OR antenatal OR “ante-natal” OR birth* OR childbirth OR “child-birth” OR “child birth” OR postnatal OR “post-natal” OR postpartum OR “post-partum” OR peripartum OR “peri-partum” OR antepartum OR “ante-partum” OR prenatal OR “pre-natal” OR matern* OR "after birth" OR puerper* OR obstetric* OR mother* OR mums OR mum OR moms OR mom OR prepartal OR “pre-partal” OR childbearing ) | 119,970 | Title and abstract search of pregnancy keywords |
| S2 | TI ( mental* OR depress* OR “well-being” OR “well being” OR wellbeing OR "baby blues" OR distress* OR stress* OR psychological* OR psychiatric ) OR AB ( mental* OR depress* OR “well-being” OR “well being” OR wellbeing OR "baby blues" OR distress* OR stress* OR psychological* OR psychiatric ) | 334,727 | Title and abstract search of depression keywords |
| S3 | S1 AND S2 | 5,695 | Combination of the two previous searches to form perinatal depression |
| S4 | TI ( digital OR “digital health” OR telehealth OR phone* OR “cell-phone” OR cellphone OR “cell phone” OR telephone OR “mobile-phone” OR smartphone* OR “smart-phone” OR telemedic* OR telecare OR telesupport OR “tele-health” OR “tele-medicine” OR “tele-care” OR “tele-support” OR wearable* OR mobile OR mhealth OR m-health OR web* OR “web-based” OR “web-guided” OR “web-supported” OR “web-delivered” OR “web-assisted” OR “web-aided” OR “web-facilitated” OR “web portal” OR app OR apps OR application* OR “mobile apps” OR “mobile app” OR “app-based” OR “web-app” OR “web-apps” OR “social media” OR facebook OR twitter OR ehealth OR “e-health” OR internet OR “internet-based” OR “internet-guided” OR “internet-supported” OR “internet-delivered” OR “internet-assisted” OR “internet-aided” OR “internet-facilitated” OR online OR “on-line” OR “online-based” OR “online-guided” OR “online-supported” OR “online-delivered” OR “online-assisted” OR “online-aided” OR “online-facilitated” OR computer* OR “computer-based” OR “computer-guided” OR “computer-supported” OR “computer-delivered” OR “computer-assisted” OR “computer-aided” OR “computer-facilitated or SMS OR “text-based” OR “text based” OR “self-help” OR “self help” OR “self-guided” OR “self guided” OR “self-directed” OR “self directed” OR “self direct” OR “self-direct” OR telecommunication* OR “e-therapy” OR “e-mental” OR teletherapy OR telemental OR technolog* OR “technology-assisted” OR virtual OR cyber OR cyberpsychology OR “cyber psychology” OR cybertherapy OR iCBT OR cCBT OR eCBT or mCBT OR electronic* OR software OR telepsychiatry OR “instant messaging” OR “e-learning” ) OR AB ( digital OR “digital health” OR telehealth OR phone* OR “cell-phone” OR cellphone OR “cell phone” OR telephone OR “mobile-phone” OR smartphone* OR “smart-phone” OR telemedic* OR telecare OR telesupport OR “tele-health” OR “tele-medicine” OR “tele-care” OR “tele-support” OR wearable* OR mobile OR mhealth OR m-health OR web* OR “web-based” OR “web-guided” OR “web-supported” OR “web-delivered” OR “web-assisted” OR “web-aided” OR “web-facilitated” OR “web portal” OR app OR apps OR application* OR “mobile apps” OR “mobile app” OR “app-based” OR “web-app” OR “web-apps” OR “social media” OR facebook OR twitter OR ehealth OR “e-health” OR internet OR “internet-based” OR “internet-guided” OR “internet-supported” OR “internet-delivered” OR “internet-assisted” OR “internet-aided” OR “internet-facilitated” OR online OR “on-line” OR “online-based” OR “online-guided” OR “online-supported” OR “online-delivered” OR “online-assisted” OR “online-aided” OR “online-facilitated” OR computer* OR “computer-based” OR “computer-guided” OR “computer-supported” OR “computer-delivered” OR “computer-assisted” OR “computer-aided” OR “computer-facilitated or SMS OR “text-based” OR “text based” OR “self-help” OR “self help” OR “self-guided” OR “self guided” OR “self-directed” OR “self directed” OR “self direct” OR “self-direct” OR telecommunication* OR “e-therapy” OR “e-mental” OR teletherapy OR telemental OR technolog* OR “technology-assisted” OR virtual OR cyber OR cyberpsychology OR “cyber psychology” OR cybertherapy OR iCBT OR cCBT OR eCBT or mCBT OR electronic* OR software OR telepsychiatry OR “instant messaging” OR “e-learning” ) | 2,950,487 | Title and abstract search of digital health keywords |
| S5 | TI ( trial* OR cohort OR prospective OR retrospective OR “case-control” OR “case control” OR “quasi-experimental” OR “quasi experimental” OR intervention OR “user experience” OR “user activity” OR “user log” OR longitudinal OR efficacy OR effectiveness OR evaluat* OR comparative OR “before and after” OR “pre-post” ) NOT TI ( systematic OR review OR scoping OR protocol OR qualitative OR "focus group" OR "thematic analysis" OR "meta-analysis" OR predict* ) | 174,146 | Title search of desired study types while excluding unwanted study types reflected in titles |
| S6 | AB ( trial* OR cohort OR prospective OR retrospective OR “case-control” OR “case control” OR “quasi-experimental” OR “quasi experimental” OR intervention OR “user experience” OR “user activity” OR “user log” OR longitudinal OR efficacy OR effectiveness OR evaluat* OR comparative OR “before and after” OR “pre-post” ) NOT TI ( systematic OR review OR scoping OR protocol OR qualitative OR "focus group" OR "thematic analysis" OR "meta-analysis" OR predict* ) | 1,087,490 | Abstract search of desired study types while excluding unwanted study types reflected in titles |
| S7 | S5 OR S6 | 1,151,259 | Combining search of desired study types |
| S8 | S3 AND S4 AND S7 | 140 | Combining all searches |
| S9 | S3 AND S4 AND S7  Limiters - Publication Date: 20140101-20241231 | 104 | Year limited to between 2014 to current |
| S10 | S3 AND S4 AND S7  Limiters - Publication Date: 20140101-20241231  Narrow by Language: - english | 98 | Language limited to English only |
|  | Title screened | 8 |  |

## EMBASE – 25^th^ January 2024

| **ID** | **DETAILS** | **NO.** | **COMMENTS** |
| --- | --- | --- | --- |
| #1 | pregnan*:ti,ab,kw OR perinatal:ti,ab,kw OR 'peri-natal':ti,ab,kw OR prepartum:ti,ab,kw OR 'pre-partum':ti,ab,kw OR antenatal:ti,ab,kw OR 'ante-natal':ti,ab,kw OR birth*:ti,ab,kw OR childbirth:ti,ab,kw OR 'child-birth':ti,ab,kw OR 'child birth':ti,ab,kw OR postnatal:ti,ab,kw OR 'post-natal':ti,ab,kw OR postpartum:ti,ab,kw OR 'post-partum':ti,ab,kw OR peripartum:ti,ab,kw OR 'peri-partum':ti,ab,kw OR antepartum:ti,ab,kw OR 'ante-partum':ti,ab,kw OR prenatal:ti,ab,kw OR 'pre-natal':ti,ab,kw OR matern*:ti,ab,kw OR 'after birth':ti,ab,kw OR puerper*:ti,ab,kw OR obstetric*:ti,ab,kw OR mother*:ti,ab,kw OR mums:ti,ab,kw OR mum:ti,ab,kw OR moms:ti,ab,kw OR mom:ti,ab,kw OR prepartal:ti,ab,kw OR 'pre-partal':ti,ab,kw OR childbearing:ti,ab,kw | 1,832,944 | Title, abstract, and keywords search of pregnancy keywords |
| #2 | mental*:ti,ab,kw OR depress*:ti,ab,kw OR 'well-being':ti,ab,kw OR 'well being':ti,ab,kw OR wellbeing:ti,ab,kw OR 'baby blues':ti,ab,kw OR distress*:ti,ab,kw OR stress*:ti,ab,kw OR psychological*:ti,ab,kw OR psychiatric:ti,ab,kw | 3,199,755 | Title, abstract, and keywords search of depression keywords |
| #3 | #1 AND #2 | 218,640 | Combination of previous searches for entries related to perinatal depression |
| #4 | 'perinatal depression'/exp | 18,000 | Emtree exploded search of perinatal depression |
| #5 | #3 OR #4 | 221,083 | Combining all sources for perinatal depression |
| #6 | digital:ti,ab,kw OR 'digital health':ti,ab,kw OR telehealth:ti,ab,kw OR phone*:ti,ab,kw OR 'cell-phone':ti,ab,kw OR cellphone:ti,ab,kw OR 'cell phone':ti,ab,kw OR telephone:ti,ab,kw OR 'mobile-phone':ti,ab,kw OR smartphone*:ti,ab,kw OR 'smart-phone':ti,ab,kw OR telemedic*:ti,ab,kw OR telecare:ti,ab,kw OR telesupport:ti,ab,kw OR 'tele-health':ti,ab,kw OR 'tele-medicine':ti,ab,kw OR 'tele-care':ti,ab,kw OR 'tele-support':ti,ab,kw OR wearable*:ti,ab,kw OR mobile:ti,ab,kw OR mhealth:ti,ab,kw OR 'm health':ti,ab,kw OR web*:ti,ab,kw OR 'web-based':ti,ab,kw OR 'web-guided':ti,ab,kw OR 'web-supported':ti,ab,kw OR 'web-delivered':ti,ab,kw OR 'web-assisted':ti,ab,kw OR 'web-aided':ti,ab,kw OR 'web-facilitated':ti,ab,kw OR 'web portal':ti,ab,kw OR app:ti,ab,kw OR apps:ti,ab,kw OR application*:ti,ab,kw OR 'mobile apps':ti,ab,kw OR 'mobile app':ti,ab,kw OR 'app-based':ti,ab,kw OR 'web-app':ti,ab,kw OR 'web-apps':ti,ab,kw OR 'social media':ti,ab,kw OR facebook:ti,ab,kw OR twitter:ti,ab,kw OR ehealth:ti,ab,kw OR 'e-health':ti,ab,kw OR internet:ti,ab,kw OR 'internet-based':ti,ab,kw OR 'internet-guided':ti,ab,kw OR 'internet-supported':ti,ab,kw OR 'internet-delivered':ti,ab,kw OR 'internet-assisted':ti,ab,kw OR 'internet-aided':ti,ab,kw OR 'internet-facilitated':ti,ab,kw OR online:ti,ab,kw OR 'on-line':ti,ab,kw OR 'online-based':ti,ab,kw OR 'online-guided':ti,ab,kw OR 'online-supported':ti,ab,kw OR 'online-delivered':ti,ab,kw OR 'online-assisted':ti,ab,kw OR 'online-aided':ti,ab,kw OR 'online-facilitated':ti,ab,kw OR computer*:ti,ab,kw OR 'computer-based':ti,ab,kw OR 'computer-guided':ti,ab,kw OR 'computer-supported':ti,ab,kw OR 'computer-delivered':ti,ab,kw OR 'computer-assisted':ti,ab,kw OR 'computer-aided':ti,ab,kw OR 'computer facilitated':ti,ab,kw OR sms:ti,ab,kw OR 'text-based':ti,ab,kw OR 'text based':ti,ab,kw OR 'self-help':ti,ab,kw OR 'self help':ti,ab,kw OR 'self-guided':ti,ab,kw OR 'self guided':ti,ab,kw OR 'self-directed':ti,ab,kw OR 'self directed':ti,ab,kw OR 'self direct':ti,ab,kw OR 'self-direct':ti,ab,kw OR telecommunication*:ti,ab,kw OR 'e-therapy':ti,ab,kw OR 'e-mental':ti,ab,kw OR teletherapy:ti,ab,kw OR telemental:ti,ab,kw OR technolog*:ti,ab,kw OR 'technology-assisted':ti,ab,kw OR virtual:ti,ab,kw OR cyber:ti,ab,kw OR cyberpsychology:ti,ab,kw OR 'cyber psychology':ti,ab,kw OR cybertherapy:ti,ab,kw OR icbt:ti,ab,kw OR ccbt:ti,ab,kw OR ecbt:ti,ab,kw OR mcbt:ti,ab,kw OR electronic*:ti,ab,kw OR software:ti,ab,kw OR telepsychiatry:ti,ab,kw OR 'instant messaging':ti,ab,kw OR 'e-learning':ti,ab,kw | 4,756,663 | Title, abstract, and keywords search of digital health keywords |
| #7 | 'digital health'/exp OR 'telemedicine'/de OR 'telemental health'/de OR 'teleconsultation'/de OR 'teletherapy'/de OR 'e counseling'/de | 61,064 | Emtree search of digital health keywords, exploding only digital health and keeping others unexploded. |
| #8 | #6 OR #7 | 4,768,350 | Combining all returns for digital health keywords |
| #9 | ((trial*:ab,ti OR cohort:ab,ti OR prospective:ab,ti OR retrospective:ab,ti OR 'case-control':ab,ti OR 'case control':ab,ti OR 'quasi-experimental':ab,ti OR 'quasi experimental':ab,ti OR intervention:ab,ti OR 'user experience':ab,ti OR 'user activity':ab,ti OR 'user log':ab,ti OR longitudinal:ab,ti OR efficacy:ab,ti OR effectiveness:ab,ti OR evaluat*:ab,ti OR comparative:ab,ti OR before:ab,ti) AND after:ab,ti OR 'pre-post':ab,ti) NOT (systematic:ti OR review:ti OR scoping:ti OR protocol:ti OR qualitative:ti OR 'focus group':ti OR 'thematic analysis':ti OR 'meta-analysis':ti OR predict*:ti) | 3,974,402 | Title and abstract search of desired study types while removing unwanted study types reflected in titles |
| #10 | #5 AND #8 AND #9 | 4,289 | Combining all search results |
| #11 | #10 AND [embase]/lim NOT ([embase]/lim AND [medline]/lim) | 2,395 | Limiting the search source to Embase only |
| #12 | #11 AND (2014:py OR 2015:py OR 2016:py OR 2017:py OR 2018:py OR 2019:py OR 2020:py OR 2021:py OR 2022:py OR 2023:py OR 2024:py) | 1,770 | Limiting to sources between 2014 to date |
|  | Title screened | 68 |  |

## APA PsychInfo – 26^th^ January 2024

| **ID** | **DETAILS** | **NO.** | **COMMENTS** |
| --- | --- | --- | --- |
| S1 | TI ( pregnan* or perinatal OR “peri-natal” OR prepartum OR “pre-partum” OR antenatal OR “ante-natal” OR birth* OR childbirth OR “child-birth” OR “child birth” OR postnatal OR “post-natal” OR postpartum OR “post-partum” OR peripartum OR “peri-partum” OR antepartum OR “ante-partum” OR prenatal OR “pre-natal” OR matern* OR "after birth" OR puerper* OR obstetric* OR mother* OR mums OR mum OR moms OR mom OR prepartal OR “pre-partal” OR childbearing ) OR AB ( pregnan* or perinatal OR “peri-natal” OR prepartum OR “pre-partum” OR antenatal OR “ante-natal” OR birth* OR childbirth OR “child-birth” OR “child birth” OR postnatal OR “post-natal” OR postpartum OR “post-partum” OR peripartum OR “peri-partum” OR antepartum OR “ante-partum” OR prenatal OR “pre-natal” OR matern* OR "after birth" OR puerper* OR obstetric* OR mother* OR mums OR mum OR moms OR mom OR prepartal OR “pre-partal” OR childbearing ) | 273,851 | Title and abstract search of pregnancy keywords |
| S2 | TI ( mental* OR depress* OR “well-being” OR “well being” OR wellbeing OR "baby blues" OR distress* OR stress* OR psychological* OR psychiatric ) OR AB ( mental* OR depress* OR “well-being” OR “well being” OR wellbeing OR "baby blues" OR distress* OR stress* OR psychological* OR psychiatric ) | 1,442,141 | Title and abstract search of depression keywords |
| S3 | S1 AND S2 | 91,207 | Combination of the previous searches to generate perinatal depression papers |
| S4 | DE "Postpartum Depression" | 6,273 | Exploded thesaurus search of postpartum depression |
| S5 | S3 OR S4 | 91,496 | Combination of all perinatal depression returns |
| S6 | TI ( digital OR “digital health” OR telehealth OR phone* OR “cell-phone” OR cellphone OR “cell phone” OR telephone OR “mobile-phone” OR smartphone* OR “smart-phone” OR telemedic* OR telecare OR telesupport OR “tele-health” OR “tele-medicine” OR “tele-care” OR “tele-support” OR wearable* OR mobile OR mhealth OR m-health OR web* OR “web-based” OR “web-guided” OR “web-supported” OR “web-delivered” OR “web-assisted” OR “web-aided” OR “web-facilitated” OR “web portal” OR app OR apps OR application* OR “mobile apps” OR “mobile app” OR “app-based” OR “web-app” OR “web-apps” OR “social media” OR facebook OR twitter OR ehealth OR “e-health” OR internet OR “internet-based” OR “internet-guided” OR “internet-supported” OR “internet-delivered” OR “internet-assisted” OR “internet-aided” OR “internet-facilitated” OR online OR “on-line” OR “online-based” OR “online-guided” OR “online-supported” OR “online-delivered” OR “online-assisted” OR “online-aided” OR “online-facilitated” OR computer* OR “computer-based” OR “computer-guided” OR “computer-supported” OR “computer-delivered” OR “computer-assisted” OR “computer-aided” OR “computer-facilitated or SMS OR “text-based” OR “text based” OR “self-help” OR “self help” OR “self-guided” OR “self guided” OR “self-directed” OR “self directed” OR “self direct” OR “self-direct” OR telecommunication* OR “e-therapy” OR “e-mental” OR teletherapy OR telemental OR technolog* OR “technology-assisted” OR virtual OR cyber OR cyberpsychology OR “cyber psychology” OR cybertherapy OR iCBT OR cCBT OR eCBT or mCBT OR electronic* OR software OR telepsychiatry OR “instant messaging” OR “e-learning” ) OR AB ( digital OR “digital health” OR telehealth OR phone* OR “cell-phone” OR cellphone OR “cell phone” OR telephone OR “mobile-phone” OR smartphone* OR “smart-phone” OR telemedic* OR telecare OR telesupport OR “tele-health” OR “tele-medicine” OR “tele-care” OR “tele-support” OR wearable* OR mobile OR mhealth OR m-health OR web* OR “web-based” OR “web-guided” OR “web-supported” OR “web-delivered” OR “web-assisted” OR “web-aided” OR “web-facilitated” OR “web portal” OR app OR apps OR application* OR “mobile apps” OR “mobile app” OR “app-based” OR “web-app” OR “web-apps” OR “social media” OR facebook OR twitter OR ehealth OR “e-health” OR internet OR “internet-based” OR “internet-guided” OR “internet-supported” OR “internet-delivered” OR “internet-assisted” OR “internet-aided” OR “internet-facilitated” OR online OR “on-line” OR “online-based” OR “online-guided” OR “online-supported” OR “online-delivered” OR “online-assisted” OR “online-aided” OR “online-facilitated” OR computer* OR “computer-based” OR “computer-guided” OR “computer-supported” OR “computer-delivered” OR “computer-assisted” OR “computer-aided” OR “computer-facilitated or SMS OR “text-based” OR “text based” OR “self-help” OR “self help” OR “self-guided” OR “self guided” OR “self-directed” OR “self directed” OR “self direct” OR “self-direct” OR telecommunication* OR “e-therapy” OR “e-mental” OR teletherapy OR telemental OR technolog* OR “technology-assisted” OR virtual OR cyber OR cyberpsychology OR “cyber psychology” OR cybertherapy OR iCBT OR cCBT OR eCBT or mCBT OR electronic* OR software OR telepsychiatry OR “instant messaging” OR “e-learning” ) | 644,100 | Title and abstract search of digital health keywords |
| S7 | (DE "Digital Mental Health Resources" OR DE "Digital Health Resources" OR DE "Digital Mental Health Resources") OR (DE "Electronic Health Services" OR DE "Digital Interventions" OR DE "Mobile Health" OR DE "Precision Medicine" OR DE "Telemedicine" OR DE "Wearable Devices") | 16,447 | Exploded thesaurus search of digital health related keywords |
| S8 | S6 OR S7 | 646,992 | Combination of all digital health returns |
| S9 | TI ( trial* OR cohort OR prospective OR retrospective OR “case-control” OR “case control” OR “quasi-experimental” OR “quasi experimental” OR intervention OR “user experience” OR “user activity” OR “user log” OR longitudinal OR efficacy OR effectiveness OR evaluat* OR comparative OR “before and after” OR “pre-post” ) NOT TI ( systematic OR review OR scoping OR protocol OR qualitative OR "focus group" OR "thematic analysis" OR "meta-analysis" OR predict* ) | 347,064 | Title search of desired study types while excluding unwanted study types reflected in titles |
| S10 | AB ( trial* OR cohort OR prospective OR retrospective OR “case-control” OR “case control” OR “quasi-experimental” OR “quasi experimental” OR intervention OR “user experience” OR “user activity” OR “user log” OR longitudinal OR efficacy OR effectiveness OR evaluat* OR comparative OR “before and after” OR “pre-post” ) NOT TI ( systematic OR review OR scoping OR protocol OR qualitative OR "focus group" OR "thematic analysis" OR "meta-analysis" OR predict* ) | 1,384,394 | Abstract search of desired study types while excluding unwanted study types reflected in titles |
| S11 | S9 OR S10 | 1,460,370 | Combination of returns for desired study types |
| S12 | S5 AND S8 AND S11 | 2,783 | Combination of all major searches |
| S13 | S5 AND S8 AND S11  Limiters - Publication Year: 2014-2024 | 1,865 | Limiting publication year to between 2014 and current |
| S14 | S5 AND S8 AND S11  Limiters - Publication Year: 2014-2024  Narrow by Language: - english | 1,806 | Narrowed by language to only English |
|  | Excluding book sources | 1,733 |  |
|  | Title screened | 209 |  |

## CINAHL 25^th^ January 2024

| **ID** | **DETAILS** | **NO.** | **Comment** |
| --- | --- | --- | --- |
| S1 | TI ( pregnan* or perinatal OR “peri-natal” OR prepartum OR “pre-partum” OR antenatal OR “ante-natal” OR birth* OR childbirth OR “child-birth” OR “child birth” OR postnatal OR “post-natal” OR postpartum OR “post-partum” OR peripartum OR “peri-partum” OR antepartum OR “ante-partum” OR prenatal OR “pre-natal” OR matern* OR "after birth" OR puerper* OR obstetric* OR mother* OR mums OR mum OR moms OR mom OR prepartal OR “pre-partal” OR childbearing ) OR AB ( pregnan* or perinatal OR “peri-natal” OR prepartum OR “pre-partum” OR antenatal OR “ante-natal” OR birth* OR childbirth OR “child-birth” OR “child birth” OR postnatal OR “post-natal” OR postpartum OR “post-partum” OR peripartum OR “peri-partum” OR antepartum OR “ante-partum” OR prenatal OR “pre-natal” OR matern* OR "after birth" OR puerper* OR obstetric* OR mother* OR mums OR mum OR moms OR mom OR prepartal OR “pre-partal” OR childbearing ) | 429,147 | Title and abstract search of pregnancy keywords |
| S2 | TI ( mental* OR depress* OR “well-being” OR “well being” OR wellbeing OR "baby blues" OR distress* OR stress* OR psychological* OR psychiatric ) OR AB ( mental* OR depress* OR “well-being” OR “well being” OR wellbeing OR "baby blues" OR distress* OR stress* OR psychological* OR psychiatric ) | 749,347 | Title and abstract search of depression keywords |
| S3 | S1 AND S2 | 60,242 | Combination of the previous searches to generate perinatal depression papers |
| S4 | (MH "Depression, Postpartum") |  | Unexploded subject heading search of postpartum depression |
| S5 | S3 OR S4 | 61,421 | Combination of all perinatal depression returns |
| S6 | TI ( digital OR “digital health” OR telehealth OR phone* OR “cell-phone” OR cellphone OR “cell phone” OR telephone OR “mobile-phone” OR smartphone* OR “smart-phone” OR telemedic* OR telecare OR telesupport OR “tele-health” OR “tele-medicine” OR “tele-care” OR “tele-support” OR wearable* OR mobile OR mhealth OR m-health OR web* OR “web-based” OR “web-guided” OR “web-supported” OR “web-delivered” OR “web-assisted” OR “web-aided” OR “web-facilitated” OR “web portal” OR app OR apps OR application* OR “mobile apps” OR “mobile app” OR “app-based” OR “web-app” OR “web-apps” OR “social media” OR facebook OR twitter OR ehealth OR “e-health” OR internet OR “internet-based” OR “internet-guided” OR “internet-supported” OR “internet-delivered” OR “internet-assisted” OR “internet-aided” OR “internet-facilitated” OR online OR “on-line” OR “online-based” OR “online-guided” OR “online-supported” OR “online-delivered” OR “online-assisted” OR “online-aided” OR “online-facilitated” OR computer* OR “computer-based” OR “computer-guided” OR “computer-supported” OR “computer-delivered” OR “computer-assisted” OR “computer-aided” OR “computer-facilitated or SMS OR “text-based” OR “text based” OR “self-help” OR “self help” OR “self-guided” OR “self guided” OR “self-directed” OR “self directed” OR “self direct” OR “self-direct” OR telecommunication* OR “e-therapy” OR “e-mental” OR teletherapy OR telemental OR technolog* OR “technology-assisted” OR virtual OR cyber OR cyberpsychology OR “cyber psychology” OR cybertherapy OR iCBT OR cCBT OR eCBT or mCBT OR electronic* OR software OR telepsychiatry OR “instant messaging” OR “e-learning” ) OR AB ( digital OR “digital health” OR telehealth OR phone* OR “cell-phone” OR cellphone OR “cell phone” OR telephone OR “mobile-phone” OR smartphone* OR “smart-phone” OR telemedic* OR telecare OR telesupport OR “tele-health” OR “tele-medicine” OR “tele-care” OR “tele-support” OR wearable* OR mobile OR mhealth OR m-health OR web* OR “web-based” OR “web-guided” OR “web-supported” OR “web-delivered” OR “web-assisted” OR “web-aided” OR “web-facilitated” OR “web portal” OR app OR apps OR application* OR “mobile apps” OR “mobile app” OR “app-based” OR “web-app” OR “web-apps” OR “social media” OR facebook OR twitter OR ehealth OR “e-health” OR internet OR “internet-based” OR “internet-guided” OR “internet-supported” OR “internet-delivered” OR “internet-assisted” OR “internet-aided” OR “internet-facilitated” OR online OR “on-line” OR “online-based” OR “online-guided” OR “online-supported” OR “online-delivered” OR “online-assisted” OR “online-aided” OR “online-facilitated” OR computer* OR “computer-based” OR “computer-guided” OR “computer-supported” OR “computer-delivered” OR “computer-assisted” OR “computer-aided” OR “computer-facilitated or SMS OR “text-based” OR “text based” OR “self-help” OR “self help” OR “self-guided” OR “self guided” OR “self-directed” OR “self directed” OR “self direct” OR “self-direct” OR telecommunication* OR “e-therapy” OR “e-mental” OR teletherapy OR telemental OR technolog* OR “technology-assisted” OR virtual OR cyber OR cyberpsychology OR “cyber psychology” OR cybertherapy OR iCBT OR cCBT OR eCBT or mCBT OR electronic* OR software OR telepsychiatry OR “instant messaging” OR “e-learning” ) | 686,694 | Title and abstract search of digital health keywords |
| S7 | (MH "Digital Health+") OR (MH "Telepsychiatry") OR (MH "Remote Consultation") OR (MH "Telenursing") OR (MH "Smartphone") OR (MH "Internet-Based Intervention") OR (MH "Social Media+") OR (MH "Teleconferencing") | 62,454 | Subject heading search of digital health related keywords (digital health and social media were exploded) |
| S8 | S6 OR S7 | 712,461 | Combination of all digital health returns |
| S9 | TI ( trial* OR cohort OR prospective OR retrospective OR “case-control” OR “case control” OR “quasi-experimental” OR “quasi experimental” OR intervention OR “user experience” OR “user activity” OR “user log” OR longitudinal OR efficacy OR effectiveness OR evaluat* OR comparative OR “before and after” OR “pre-post” ) NOT TI ( systematic OR review OR scoping OR protocol OR qualitative OR "focus group" OR "thematic analysis" OR "meta-analysis" OR predict* ) | 672,214 | Title search of desired study types while excluding unwanted study types reflected in titles |
| S10 | AB ( trial* OR cohort OR prospective OR retrospective OR “case-control” OR “case control” OR “quasi-experimental” OR “quasi experimental” OR intervention OR “user experience” OR “user activity” OR “user log” OR longitudinal OR efficacy OR effectiveness OR evaluat* OR comparative OR “before and after” OR “pre-post” ) NOT TI ( systematic OR review OR scoping OR protocol OR qualitative OR "focus group" OR "thematic analysis" OR "meta-analysis" OR predict* ) | 1,725,878 | Abstract search of desired study types while excluding unwanted study types reflected in titles |
| S11 | S9 OR S10 | 2,015,383 | Combination of returns for desired study types |
| S12 | S5 AND S8 AND S11 | 2,541 | Combination of all major searches |
| S13 | S5 AND S8 AND S11  Limiters - Publication Date: 20140101-20241231 | 1,891 | Limiting publication year to between 2014 and current |
| S14 | S5 AND S8 AND S11  Limiters - Publication Date: 20140101-20241231  Narrow by Language: - english | 1,835 | Narrowed by language to only English |
|  | Excluding magazines and continuing education units | 1,815 |  |
|  | Title screened | 194 |  |

## WEB OF SCIENCE 26^th^ January 2024

| **ID** | **DETAILS** | **NO.** | **Comment** |
| --- | --- | --- | --- |
| #1 | (TI=(pregnan* or perinatal OR “peri-natal” OR prepartum OR “pre-partum” OR antenatal OR “ante-natal” OR birth* OR childbirth OR “child-birth” OR “child birth” OR postnatal OR “post-natal” OR postpartum OR “post-partum” OR peripartum OR “peri-partum” OR antepartum OR “ante-partum” OR prenatal OR “pre-natal” OR matern* OR "after birth" OR puerper* OR obstetric* OR mother* OR mums OR mum OR moms OR mom OR prepartal OR “pre-partal” OR childbearing)) OR AB=(pregnan* or perinatal OR “peri-natal” OR prepartum OR “pre-partum” OR antenatal OR “ante-natal” OR birth* OR childbirth OR “child-birth” OR “child birth” OR postnatal OR “post-natal” OR postpartum OR “post-partum” OR peripartum OR “peri-partum” OR antepartum OR “ante-partum” OR prenatal OR “pre-natal” OR matern* OR "after birth" OR puerper* OR obstetric* OR mother* OR mums OR mum OR moms OR mom OR prepartal OR “pre-partal” OR childbearing) | 1,605,059 | Title and abstract search of pregnancy keywords |
| #2 | (TI=(mental* OR depress* OR “well-being” OR “well being” OR wellbeing OR "baby blues" OR distress* OR stress* OR psychological* OR psychiatric)) OR AB=(mental* OR depress* OR “well-being” OR “well being” OR wellbeing OR "baby blues" OR distress* OR stress* OR psychological* OR psychiatric) | 3,718,705 | Title and abstract search of depression keywords |
| #3 | #1 AND #2 | 162,151 | Combination of the previous searches to generate perinatal depression papers |
| #4 | (TI=(digital OR “digital health” OR telehealth OR phone* OR “cell-phone” OR cellphone OR “cell phone” OR telephone OR “mobile-phone” OR smartphone* OR “smart-phone” OR telemedic* OR telecare OR telesupport OR “tele-health” OR “tele-medicine” OR “tele-care” OR “tele-support” OR wearable* OR mobile OR mhealth OR m-health OR web* OR “web-based” OR “web-guided” OR “web-supported” OR “web-delivered” OR “web-assisted” OR “web-aided” OR “web-facilitated” OR “web portal” OR app OR apps OR application* OR “mobile apps” OR “mobile app” OR “app-based” OR “web-app” OR “web-apps” OR “social media” OR facebook OR twitter OR ehealth OR “e-health” OR internet OR “internet-based” OR “internet-guided” OR “internet-supported” OR “internet-delivered” OR “internet-assisted” OR “internet-aided” OR “internet-facilitated” OR online OR “on-line” OR “online-based” OR “online-guided” OR “online-supported” OR “online-delivered” OR “online-assisted” OR “online-aided” OR “online-facilitated” OR computer* OR “computer-based” OR “computer-guided” OR “computer-supported” OR “computer-delivered” OR “computer-assisted” OR “computer-aided” OR “computer-facilitated” OR SMS OR “text-based” OR “text based” OR “self-help” OR “self help” OR “self-guided” OR “self guided” OR “self-directed” OR “self directed” OR “self direct” OR “self-direct” OR telecommunication* OR “e-therapy” OR “e-mental” OR teletherapy OR telemental OR technolog* OR “technology-assisted” OR virtual OR cyber OR cyberpsychology OR “cyber psychology” OR cybertherapy OR iCBT OR cCBT OR eCBT or mCBT OR electronic* OR software OR telepsychiatry OR “instant messaging” OR “e-learning”)) OR AB=(digital OR “digital health” OR telehealth OR phone* OR “cell-phone” OR cellphone OR “cell phone” OR telephone OR “mobile-phone” OR smartphone* OR “smart-phone” OR telemedic* OR telecare OR telesupport OR “tele-health” OR “tele-medicine” OR “tele-care” OR “tele-support” OR wearable* OR mobile OR mhealth OR m-health OR web* OR “web-based” OR “web-guided” OR “web-supported” OR “web-delivered” OR “web-assisted” OR “web-aided” OR “web-facilitated” OR “web portal” OR app OR apps OR application* OR “mobile apps” OR “mobile app” OR “app-based” OR “web-app” OR “web-apps” OR “social media” OR facebook OR twitter OR ehealth OR “e-health” OR internet OR “internet-based” OR “internet-guided” OR “internet-supported” OR “internet-delivered” OR “internet-assisted” OR “internet-aided” OR “internet-facilitated” OR online OR “on-line” OR “online-based” OR “online-guided” OR “online-supported” OR “online-delivered” OR “online-assisted” OR “online-aided” OR “online-facilitated” OR computer* OR “computer-based” OR “computer-guided” OR “computer-supported” OR “computer-delivered” OR “computer-assisted” OR “computer-aided” OR “computer-facilitated” OR SMS OR “text-based” OR “text based” OR “self-help” OR “self help” OR “self-guided” OR “self guided” OR “self-directed” OR “self directed” OR “self direct” OR “self-direct” OR telecommunication* OR “e-therapy” OR “e-mental” OR teletherapy OR telemental OR technolog* OR “technology-assisted” OR virtual OR cyber OR cyberpsychology OR “cyber psychology” OR cybertherapy OR iCBT OR cCBT OR eCBT or mCBT OR electronic* OR software OR telepsychiatry OR “instant messaging” OR “e-learning”) | 11,525,591 | Title and abstract search of digital health keywords |
| #5 | (TI=(trial* OR cohort OR prospective OR retrospective OR “case-control” OR “case control” OR “quasi-experimental” OR “quasi experimental” OR intervention OR “user experience” OR “user activity” OR “user log” OR longitudinal OR efficacy OR effectiveness OR evaluat* OR comparative OR “before and after” OR “pre-post”)) NOT TI=(systematic OR review OR scoping OR protocol OR qualitative OR "focus group" OR "thematic analysis" OR "meta-analysis" OR predict* ) | 3,264,217 | Title search of desired study types while excluding unwanted study types reflected in titles |
| #6 | (AB=(trial* OR cohort OR prospective OR retrospective OR “case-control” OR “case control” OR “quasi-experimental” OR “quasi experimental” OR intervention OR “user experience” OR “user activity” OR “user log” OR longitudinal OR efficacy OR effectiveness OR evaluat* OR comparative OR “before and after” OR “pre-post”)) NOT TI=(systematic OR review OR scoping OR protocol OR qualitative OR "focus group" OR "thematic analysis" OR "meta-analysis" OR predict* ) | 10,455,979 | Abstract search of desired study types while excluding unwanted study types reflected in titles |
| #7 | #5 OR #6 | 12,120,029 | Combination of returns for desired study types |
| #8 | #7 AND #4 AND #3 | 6,203 | Combination of all major searches |
| #9 | #8  Timespan: 2014-01-01 to 2024-01-26 (Publication Date) | 4,452 | Limiting publication year to between 2014 and current |
| #10 | #8 and English (Languages)  Timespan: 2014-01-01 to 2024-01-26 (Publication Date) | 4,319 | Narrowed by language to only English |
|  | Title screening | 282 |  |

## CENTRAL 27^th^ January 2024

| **ID** | **DETAILS** | **NO.** | **Comment** |
| --- | --- | --- | --- |
| #1 | (pregnan* or perinatal OR “peri-natal” OR prepartum OR “pre-partum” OR antenatal OR “ante-natal” OR birth* OR childbirth OR “child-birth” OR “child birth” OR postnatal OR “post-natal” OR postpartum OR “post-partum” OR peripartum OR “peri-partum” OR antepartum OR “ante-partum” OR prenatal OR “pre-natal” OR matern* OR "after birth" OR puerper* OR obstetric* OR mother* OR mums OR mum OR moms OR mom OR prepartal OR “pre-partal” OR childbearing):ti OR (pregnan* or perinatal OR “peri-natal” OR prepartum OR “pre-partum” OR antenatal OR “ante-natal” OR birth* OR childbirth OR “child-birth” OR “child birth” OR postnatal OR “post-natal” OR postpartum OR “post-partum” OR peripartum OR “peri-partum” OR antepartum OR “ante-partum” OR prenatal OR “pre-natal” OR matern* OR "after birth" OR puerper* OR obstetric* OR mother* OR mums OR mum OR moms OR mom OR prepartal OR “pre-partal” OR childbearing):ab | 122,501 | Title and abstract search of pregnancy keywords |
| #2 | (mental* OR depress* OR “well-being” OR “well being” OR wellbeing OR "baby blues" OR distress* OR stress* OR psychological* OR psychiatric):ti OR (mental* OR depress* OR “well-being” OR “well being” OR wellbeing OR "baby blues" OR distress* OR stress* OR psychological* OR psychiatric):ab | 248,451 | Title and abstract search of depression keywords |
| #3 | #1 AND #2 | 20,177 | Combination of the previous searches to generate perinatal depression papers |
| #4 | MeSH descriptor: [Depression, Postpartum] 1 tree(s) exploded | 888 | Exploded subject heading search of postpartum depression |
| #5 | #3 OR #4 | 20,212 | Combination of all perinatal depression returns |
| #6 | (digital OR “digital health” OR telehealth OR phone* OR “cell-phone” OR cellphone OR “cell phone” OR telephone OR “mobile-phone” OR smartphone* OR “smart-phone” OR telemedic* OR telecare OR telesupport OR “tele-health” OR “tele-medicine” OR “tele-care” OR “tele-support” OR wearable* OR mobile OR mhealth OR m-health OR web* OR “web-based” OR “web-guided” OR “web-supported” OR “web-delivered” OR “web-assisted” OR “web-aided” OR “web-facilitated” OR “web portal” OR app OR apps OR application* OR “mobile apps” OR “mobile app” OR “app-based” OR “web-app” OR “web-apps” OR “social media” OR facebook OR twitter OR ehealth OR “e-health” OR internet OR “internet-based” OR “internet-guided” OR “internet-supported” OR “internet-delivered” OR “internet-assisted” OR “internet-aided” OR “internet-facilitated” OR online OR “on-line” OR “online-based” OR “online-guided” OR “online-supported” OR “online-delivered” OR “online-assisted” OR “online-aided” OR “online-facilitated” OR computer* OR “computer-based” OR “computer-guided” OR “computer-supported” OR “computer-delivered” OR “computer-assisted” OR “computer-aided” OR “computer-facilitated” OR SMS OR “text-based” OR “text based” OR “self-help” OR “self help” OR “self-guided” OR “self guided” OR “self-directed” OR “self directed” OR “self direct” OR “self-direct” OR telecommunication* OR “e-therapy” OR “e-mental” OR teletherapy OR telemental OR technolog* OR “technology-assisted” OR virtual OR cyber OR cyberpsychology OR “cyber psychology” OR cybertherapy OR iCBT OR cCBT OR eCBT or mCBT OR electronic* OR software OR telepsychiatry OR “instant messaging” OR “e-learning”):ti OR (digital OR “digital health” OR telehealth OR phone* OR “cell-phone” OR cellphone OR “cell phone” OR telephone OR “mobile-phone” OR smartphone* OR “smart-phone” OR telemedic* OR telecare OR telesupport OR “tele-health” OR “tele-medicine” OR “tele-care” OR “tele-support” OR wearable* OR mobile OR mhealth OR m-health OR web* OR “web-based” OR “web-guided” OR “web-supported” OR “web-delivered” OR “web-assisted” OR “web-aided” OR “web-facilitated” OR “web portal” OR app OR apps OR application* OR “mobile apps” OR “mobile app” OR “app-based” OR “web-app” OR “web-apps” OR “social media” OR facebook OR twitter OR ehealth OR “e-health” OR internet OR “internet-based” OR “internet-guided” OR “internet-supported” OR “internet-delivered” OR “internet-assisted” OR “internet-aided” OR “internet-facilitated” OR online OR “on-line” OR “online-based” OR “online-guided” OR “online-supported” OR “online-delivered” OR “online-assisted” OR “online-aided” OR “online-facilitated” OR computer* OR “computer-based” OR “computer-guided” OR “computer-supported” OR “computer-delivered” OR “computer-assisted” OR “computer-aided” OR “computer-facilitated” OR SMS OR “text-based” OR “text based” OR “self-help” OR “self help” OR “self-guided” OR “self guided” OR “self-directed” OR “self directed” OR “self direct” OR “self-direct” OR telecommunication* OR “e-therapy” OR “e-mental” OR teletherapy OR telemental OR technolog* OR “technology-assisted” OR virtual OR cyber OR cyberpsychology OR “cyber psychology” OR cybertherapy OR iCBT OR cCBT OR eCBT or mCBT OR electronic* OR software OR telepsychiatry OR “instant messaging” OR “e-learning”):ab | 291,292 | Title and abstract search of digital health keywords |
| #7 | MeSH descriptor: [Telemedicine] 2 tree(s) exploded | 4,346 | Subject heading search of digital health related keyword (telemedicine) |
| #8 | #6 OR #7 | 291,696 | Combination of all digital health returns |
| #9 | #5 AND #8 | 5,271 | Combination of all major searches |
|  | Navigating to only trials | 5,089 |  |
|  | Limiting to only ICTRP (International Clinical Trials Registry Platform) | 1,358 |  |
|  | Limiting publication year to between 2014 and current | 1,164 |  |
|  | Title screened | 103 |  |

# SUPPLEMENTARY FILE – ELIGIBILITY ASSESSMENT OUTCOME OF ALL SCREENED FULL TEXT PAPERS.

| **Authors** | **Year** | **Title** | **Population** | **Intervention** | **Comparator** | **Outcome** | **Study Type** | **Desired Results** | **Potential (SR or MA or Excluded)** | **Comments** | **Year eligibility** | **Final decision** | **Documented reason for exclusion from SR** | **Documented reason for excluding from MA** |
| --- | --- | --- | --- | --- | --- | --- | --- | --- | --- | --- | --- | --- | --- | --- |
| Abujilban S,Al-Omari H,Issa E,ALhamdan A,Al-Nabulsi L,Mrayan L,Mahmoud KF,Kernohan WG | 2023 | Effectiveness of Telephone-Based Interpersonal Psychotherapy on Antenatal Depressive Symptoms: A Prospective Randomized Controlled Trial in The Kingdom of Jordan | Yes | Yes | Yes | Yes | Yes | Yes | SR & MA | Double arm trial | Yes | Include |  |  |
| Adamo KB,Semeniuk K,da Silva DF,Souza SC,Baillargeon JP,Redman LM,Piccinini-Vallis H,Shen GX,Nerenberg K | 2023 | SmartMoms Canada: An evaluation of a mobile app intervention to support a healthy pregnancy | Yes | Yes | Yes | Yes | Yes | No | Exclude | Protocol | Yes | Exclude | Protocol |  |
| Adams AM,Wu H,Zhang FR,Wajsberg JR, Bruney TL | 2023 | Postpartum Care in the Time of COVID-19: The Use of Telemedicine for Postpartum Care | Yes | Yes | No | Yes | Yes | No | Exclude | Control group received digital intervention | Yes | Exclude | Control group received digital intervention |  |
| Albanese AM,Geller PA,Steinkamp JM,Bloch JR,Sikes C,Barkin JL | 2022 | Introducing the Postpartum Toolkit: An Examination of the Feasibility, Acceptability and Pilot Efficacy of an Online Clinical Tool to Enhance Postpartum Functioning and Emotional Wellbeing | Yes | Yes | N/A | Yes | Yes | Yes | SR only | single group study | Yes | Exclude | Poor study quality on appraisal |  |
| Ameme DK,Akweongo P,Afari EA,Noora CL,Anthony R,Kenu E | 2022 | Effectiveness of adjunct telephone-based postnatal care on maternal and infant illness in the Greater Accra Region, Ghana: a randomized controlled trial | Yes | Yes | Yes | No | Yes | No | Exclude | No report on depression measurements | Yes | Exclude | No reported depression measurements |  |
| Anis W,Amalia RB | 2021 | The effects of telehealth during pregnancy on maternal knowledge and postpartum mental health in the covid-19 pandemic | Yes | Yes | Yes | Yes | Yes | Yes | SR only | Case-control study | Yes | Exclude | Poor study quality on appraisal |  |
| Arakawa Y,Haseda M,Inoue K,Nishioka D,Kino S,Nishi D,Hashimoto H,Kondo N | 2023 | Effectiveness of mHealth consultation services for preventing postpartum depressive symptoms: a randomized clinical trial | Yes | Yes | Yes | Yes | Yes | Yes | SR only | Double arm trial | Yes | Include |  | Result presented as proportions instead of means |
| Asadzadeh L,Jafari E,Kharaghani R,Taremian F | 2020 | Effectiveness of midwife-led brief counseling intervention on post-traumatic stress disorder, depression, and anxiety symptoms of women experiencing a traumatic childbirth: a randomized controlled trial | Yes | Yes | Yes | Yes | Yes | Yes | SR & MA | Double arm trial | Yes | Include |  |  |
| Avalos LA,Aghaee S,Kurtovich E,Jr CQ,Nkemere L,McGinnis MK,Kubo A | 2020 | A Mobile Health Mindfulness Intervention for Women With Moderate to Moderately Severe Postpartum Depressive Symptoms: Feasibility Study | Yes | Yes | Yes | Yes | Yes | Yes | SR only | single group study | Yes | Include |  | Single arm studies |
| Barrera AZ,Wickham RE,Muñoz RF | 2015 | Online prevention of postpartum depression for Spanish- and English-speaking pregnant women: A pilot randomized controlled trial | Yes | Yes | Yes | Yes | Yes | Yes | SR & MA | Double arm trial | No | Exclude | Year (2014-2018) |  |
| Baumel A,Tinkelman A,Mathur N,Kane JM | 2018 | Digital peer-support platform (7Cups) as an adjunct treatment for women with postpartum depression: Feasibility, acceptability, and preliminary efficacy study | Yes | Yes | Yes | Yes | Yes | Yes | SR & MA | Double arm trial | No | Exclude | Year (2014-2018) |  |
| Bear KA,Barber CC,Medvedev ON | 2022 | The impact of a mindfulness app on postnatal distress | Yes | Yes | No | Yes | Yes | Yes | Exclude | Control received digital intervention | Yes | Exclude | Control group received digital intervention |  |
| Bhat A,Mao J,Unützer J,Reed S,Unger J | 2018 | Text messaging to support a perinatal collaborative care model for depression: A multi-methods inquiry | Yes | Yes | Yes | Yes | No | No | Exclude | Qualitative | No | Exclude | Qualitative study |  |
| Boran P,Dönmez M,Barış E,Us MC,Altaş ZM,Nisar A,Atif N,Sikander S,Hıdıroğlu S,Save D,Rahman A | 2023 | Delivering the Thinking Healthy Programme as a universal group intervention integrated into routine antenatal care: A randomized-controlled pilot study | Yes | No | Yes | Yes | Yes | Yes | Exclude | Intervention is not purely digital as it was designed to be delivered either ways and hence it cannot be treated as a purely digital health intervention | Yes | Exclude | Non-digital intervention |  |
| Bourke-Taylor HM,Joyce KS,Grzegorczyn S,Tirlea L | 2022 | Mental Health and Health Behaviour Changes for Mothers of Children with a Disability: Effectiveness of a Health and Wellbeing Workshop | No | Unclear | N/A | Yes | Yes | Yes | Exclude | Mothers of Children with a Disability | Yes | Exclude | Wrong population |  |
| Boyd RC,Price J,Mogul M,Yates T,Guevara JP | 2019 | Pilot RCT of a social media parenting intervention for postpartum mothers with depression symptoms | Yes | Yes | Yes | Yes | Yes | Yes | SR & MA | Double arm trial | Yes | Include |  |  |
| Brewington-Brown AR | 2020 | Development and Evaluation of a Nurse Practitioner-Directed Mindfulness and Cognitive-Behavioral Therapy Program for Women with Perinatal Depression in an Obstetrics and Gynecology Clinical Care Center | Yes | Yes | Yes | Yes | Yes | No | SR only | Result presented as proportions instead of means | Yes | Exclude | Poor study quality on appraisal |  |
| Broom MA,Ladley AS,Rhyne EA,Halloran DR | 2015 | Feasibility and perception of using text messages as an adjunct therapy for low-income, minority mothers with postpartum depression | Yes | Yes | N/A | Yes | Yes | No | SR only | Summary statistics on depression not reported in paper | No | Exclude | Year (2014-2018) |  |
| Brunelli L,Bussolaro S,Cin MD,Ronfani L,Zanchiello S,Cassone A,Verardi G,Dobrina R,Bava M,Stampalija T | 2022 | CARE 1000: randomized controlled trial for the evaluation of the effectiveness of a mHealth app for supporting the first 1000 days of life | Yes | Yes | Yes | No | Yes | No | Exclude | A protocol and no plan for depression measures | Yes | Exclude | Protocol |  |
| Bryant AS,Coleman J,Shi X,Rodriguez M,Papadopoulos AS,Merz K,Leonard J,Samia N,Marceau L | 2023 | The power and promise of postpartum self care: Evaluation of a web-based tool for underserved women | Yes | Yes | N/A | Yes | Yes | Yes | SR only | single group study | Yes | Include |  | Single arm studies |
| Buultjens M,Gill J,Fielding J,Lambert KA,Vondeling K,Mastwyk SE,Sloane S,Fedele W,Karimi L,Milgrom J,von Treuer K,Erbas B | 2023 | Maternity care during a pandemic: Can a hybrid telehealth model comprising group interdisciplinary education support maternal psychological health? | Yes | Yes | Yes | Yes | Yes | No | SR only | Summary statistics on depression not reported in paper | Yes | Include |  | Summary statistics on depression not reported in paper |
| Byatt N,Brenckle L,Sankaran P,Flahive J,Ko JY,Robbins CL,Zimmermann M,Allison J,Person S,Simas TA | 2024 | Effectiveness of two systems-level interventions to address perinatal depression in obstetric settings (PRISM): an active-controlled cluster-randomised trial | Yes | No | Yes | Yes | Yes | Yes | Exclude | Non-digital intervention | Yes | Exclude | Non-digital intervention |  |
| Canfield SM,Canada KE,Rolbiecki AJ,Petroski GF | 2023 | Feasibility and acceptability of an online mental health intervention for pregnant women and their partners: a mixed method study with a pilot randomized control trial | No | No | Yes | Yes | Yes | Yes | Exclude | Study (population and intervention) was for couples and not about the woman herself | Yes | Exclude | Wrong population |  |
| Carona C,Pereira M,Araújo-Pedrosa A,Canavarro MC,Fonseca A | 2023 | The Efficacy of Be a Mom, a Web-Based Intervention to Prevent Postpartum Depression: Examining Mechanisms of Change in a Randomized Controlled Trial | Yes | Yes | Yes | Yes | Yes | Yes | SR & MA | Double arm trial | Yes | Include |  |  |
| Chan KL,Leung WC,Tiwari A,Or KL,Ip P | 2019 | Using smartphone-based psychoeducation to reduce postnatal depression among first-time mothers: Randomized controlled trial | Yes | Yes | Yes | Yes | Yes | Yes | SR & MA | Double arm trial | Yes | Include |  |  |
| Cheung RY,Chan SK,Chui H,Chan WM,Ngai SY | 2022 | Enhancing parental well-being: Initial efficacy of a 21-day online self-help mindfulness-based intervention for parents | No | Yes | Yes | Yes | Yes | Yes | Exclude | Population is on parents and not related to the perinatal period | Yes | Exclude | Wrong population |  |
| Chrzan-Dętkoś M,Murawska N,Walczak-Kozłowska T | 2022 | Next Stop: Mum': Evaluation of a Postpartum Depression Prevention Strategy in Poland | Yes | No | N/A | Yes | Yes | No | Exclude | Study's scope is on screening | Yes | Exclude | Study is on screening |  |
| Chyzzy B,Nelson LE,Stinson J,Vigod S,Dennis CL | 2020 | Adolescent Mothers' Perceptions of a Mobile Phone-Based Peer Support Intervention | Yes | Yes | Yes | Yes | No | No | Exclude | Qualitative | Yes | Exclude | Qualitative study |  |
| Ciciolla L,Addante S,Shreffler KM,Croff JM | 2023 | Effects of a Mindfulness-Based Parental Reflection Intervention on Pregnancy-Related Distress: A Pilot Study | Yes | No | Yes | Yes | Yes | Yes | Exclude | Non-digital intervention | Yes | Exclude | Non-digital intervention |  |
| Cluxton-Keller F,Williams M,Buteau J,Donnelly CL,Stolte P,Monroe-Cassel M,Bruce ML | 2018 | Video-Delivered Family Therapy for Home Visited Young Mothers With Perinatal Depressive Symptoms: Quasi-Experimental Implementation-Effectiveness Hybrid Trial | No | No | Yes | Yes | Yes | Yes | Exclude | Study (population and intervention) was for family and not about the woman herself | No | Exclude | Wrong population |  |
| Coo S,García MI,Pérez JC,Aldoney D,Olhaberry M,Fernández O,Alamo N,Franco P,Pérez F,Fernández S,Fisher J,Rowe H | 2023 | Online Intervention Targeting Postnatal Depression and Anxiety in Chilean First-Time Mothers: Feasibility Trial | Yes | Yes | Yes | Yes | Yes | Yes | SR & MA | Double arm trial | Yes | Include |  | Insufficient reported data for meta-analysis |
| Corno G,Etchemendy E,Espinoza M,Herrero R,Molinari G,Carrillo A,Drossaert C,Baños RM | 2018 | Effect of a web-based positive psychology intervention on prenatal well-being: A case series study | Yes | Yes | Yes | Yes | No | Yes | Exclude | Case series study | No | Exclude | Case series/report |  |
| Cox JE,Harris SK,Conroy K,Engelhart T,Vyavaharkar A,Federico A,Woods ER | 2019 | A parenting and life skills intervention for teen mothers: A randomized controlled trial | Yes | No | Yes | Yes | Yes | Yes | Exclude | Non-digital intervention | Yes | Exclude | Non-digital intervention |  |
| Daehn D,Martens C,Loew V,Kemmler L,Rudolf S,Kochen E,Renneberg B,Pawils S | 2023 | SmartMoms - a web application to raise awareness and provide information on postpartum depression | Yes | Yes | N/A | No | Yes | No | Exclude | No documented depression measurements | Yes | Exclude | No reported depression measurements |  |
| Daley AJ,Blamey RV,Jolly K,Roalfe AK,Turner KM,Coleman S,McGuinness M,Jones I,Sharp DJ,MacArthur C | 2015 | A pragmatic randomized controlled trial to evaluate the effectiveness of a facilitated exercise intervention as a treatment for postnatal depression: the PAMPeRS trial | Yes | No | Yes | Yes | Yes | Yes | Exclude | Non-digital intervention | No | Exclude | Non-digital intervention |  |
| Danaher BG,Seeley JR,Silver RK,Tyler MS,Kim JJ,Porte LM,Cleveland E,Smith DR,Milgrom J,Gau JM | 2023 | Trial of a patient-directed eHealth program to ameliorate perinatal depression: the MomMoodBooster2 practical effectiveness study | Yes | Yes | Yes | Yes | Yes | Yes | SR & MA | Double arm trial | Yes | Include |  |  |
| Davis JA,Ohan JL,Gregory S,Kottampally K,Silva D,Prescott SL,Finlay-Jones AL | 2023 | Perinatal Women's Perspectives of, and Engagement in, Digital Emotional Well-Being Training: Mixed Methods Study | Yes | Yes | No | Yes | Yes | No | Exclude | Control received digital intervention and desired results not reported in paper (although available in a separately published paper) | Yes | Exclude | Control group received digital intervention |  |
| DeMairo J,Rimsky L,Moses A,Birndorf C,Bellenbaum P,Van Nortwick N,Osborne LM,Robakis TK | 2023 | Outcomes at the motherhood center: A comparison of virtual and on-site versions of a specialized perinatal partial hospitalization program | Yes | Yes | Yes | Yes | Yes | Yes | SR & MA | Comparative study (study type not stated but appeared to be quaasi-experimental) | Yes | Include |  | Non-randomised trials |
| Dennis CL | 2014 | The process of developing and implementing a telephone-based peer support program for postpartum depression: evidence from two randomized controlled trials | Yes | Yes | Yes | Yes | No | No | Exclude | Qualitative | No | Exclude | Qualitative study |  |
| Dennis CL,Grigoriadis S,Zupancic J,Kiss A,Ravitz P | 2020 | Telephone-based nurse-delivered interpersonal psychotherapy for postpartum depression: Nationwide randomised controlled trial | Yes | Yes | Yes | Yes | Yes | Yes | SR & MA | Double arm trial | Yes | Include |  |  |
| Dennis-Tiwary TA,Denefrio S,Gelber S | 2017 | Salutary effects of an attention bias modification mobile application on biobehavioral measures of stress and anxiety during pregnancy | Yes | Yes | No | Yes | Yes | Yes | Exclude | Control received digital intervention | No | Exclude | Control group received digital intervention |  |
| Derksen C,Dietl JE,Haeussler FE,Zazo MS,Schmiedhofer M,Lippke S | 2023 | Behavior change training for pregnant women's communication during birth: A randomized controlled trial | Yes | Yes | Yes | No | Yes | No | Exclude | No documented depression measurements | Yes | Exclude | No reported depression measurements |  |
| Doty MS,Chen HY,Ajishegiri O,Sibai BM,Blackwell SC,Chauhan SP | 2022 | Daily meditation program for anxiety in individuals admitted to the antepartum unit: a multicenter randomized controlled trial (MEDITATE) | Yes | No | Yes | Yes | Yes | No | Exclude | Intervention was for anxiety and not necessarily directed for depression; Summary statistics on depression was also not reported in paper | Yes | Exclude | Intervention is not for pregnancy-related depression |  |
| Duffecy J,Grekin R,Long JD,Mills JA,O'Hara M | 2022 | Randomized controlled trial of Sunnyside: Individual versus group-based online interventions to prevent postpartum depression | Yes | Yes | No | Yes | Yes | No | Exclude | Control received digital intervention and summary statistics on depression not reported in paper | Yes | Exclude | Control group received digital intervention |  |
| Dyurich A,Oliver M | 2020 | Use of the VeedaMom electronic app as a pregnancy treatment companion | Yes | Yes | N/A | No | No | No | Exclude | Qualitative | Yes | Exclude | Qualitative study |  |
| Eisner E,Lewis S,Stockton-Powdrell C,Agass R,Whelan P,Tower C | 2022 | Digital screening for postnatal depression: mixed methods proof-of-concept study | Yes | Yes | N/A | Yes | Yes | No | Exclude | Study is on screening | Yes | Exclude | Study is on screening |  |
| Evans EC,Deutsch NL,Drake E,Bullock L | 2017 | Nurse–patient interaction as a treatment for antepartum depression: A mixed-methods analysis | Yes | No | Yes | Yes | Yes | No | Exclude | Non-digital intervention | No | Exclude | Non-digital intervention |  |
| Fatori D,Zuccolo P,Xavier MO,Matijasevich A,Polanczyk GV | 2023 | Smartphone-assisted online brief cognitive behavioral therapy to treat maternal depression: findings of a randomized controlled trial | Yes | Yes | No | Yes | Yes | Yes | Exclude | Control received digital intervention | Yes | Exclude | Control group received digital intervention |  |
| Felder JN,Epel ES,Neuhaus J,Krystal AD,Prather AA | 2022 | Randomized controlled trial of digital cognitive behavior therapy for prenatal insomnia symptoms: Effects on postpartum insomnia and mental health | Yes | No | Yes | Yes | Yes | No | Exclude | Intervention component is not for depression | Yes | Exclude | Intervention is not for pregnancy-related depression |  |
| Felder JN,Segal Z,Beck A,Sherwood NE,Goodman SH,Boggs J,Lemon E,Dimidjian S | 2017 | An open trial of web-based mindfulness-based cognitive therapy for perinatal women at risk for depressive relapse | Yes | Yes | N/A | Yes | Yes | No | SR only | Summary statistics on depression not reported in paper | No | Exclude | Year (2014-2018) |  |
| Fernandes DV,Monteiro F,Canavarro MC,Moreira H | 2022 | A Web-Based, Mindful, and Compassionate Parenting Training for Mothers Experiencing Parenting Stress: Results from a Pilot Randomized Controlled Trial of the Mindful Moment Program | No | Yes | Yes | Yes | Yes | Yes | Exclude | Population includes women who are above 12 months postpartum | Yes | Exclude | Wrong population |  |
| Fisher J,Tran T,Wynter K,Hiscock H,Bayer J,Rowe H | 2018 | Gender-informed psycho-educational programme to promote respectful relationships and reduce postpartum common mental disorders among primiparous women: Long-term follow-up of participants in a community-based cluster randomised controlled trial | No | Yes | Yes | Yes | Yes | Yes | Exclude | Population is women who are above 12 months postpartum | No | Exclude | Wrong population |  |
| Fletcher R,Campbell L,Williams AS,Rawlinson C,Dye J,Baldwin A,May C,StGeorge J | 2019 | SMS4 perinatal parents: Designing parenting support via text messages for mothers with severe mental illness (SMI) and their partners | No | No | N/A | No | Yes | No | Exclude | Study (population and intervention) was for couple and not about the woman herself | Yes | Exclude | Wrong population |  |
| Forsell E,Bendix M,Holländare F,von Schultz BS,Nasiell J,Blomdahl-Wetterholm M,Eriksson C,Kvarned S,van der Linden JL,Söderberg E,Jokinen J,Wide K,Kaldo V | 2017 | Internet delivered cognitive behavior therapy for antenatal depression: A randomised controlled trial | Yes | Yes | Yes | Yes | Yes | Yes | SR & MA | Double arm trial | No | Exclude | Year (2014-2018) |  |
| Gammer I,Hartley-Jones C,Jones FW | 2020 | A randomized controlled trial of an online, compassion-based intervention for maternal psychological well-being in the first year postpartum | Yes | Yes | Yes | Yes | Yes | Yes | SR & MA | Double arm trial | Yes | Include |  |  |
| Gao LL,Xie W,Yang X,Chan SW | 2015 | Effects of an interpersonal-psychotherapy-oriented postnatal programme for Chinese first-time mothers: a randomized controlled trial | Yes | No | Yes | Yes | Yes | Yes | Exclude | Non-digital intervention | No | Exclude | Non-digital intervention |  |
| Gemmill AW,Oliva JL,Ericksen J,Holt C,Holt CJ,Milgrom J | 2022 | Web-based treatment for depression in pregnancy: A feasibility study of Mum2BMoodBooster | Yes | Yes | N/A | Yes | Yes | Yes | SR only | single group study | Yes | Include |  | Single arm studies |
| Giallo R,Cooklin A,Dunning M,Seymour M | 2014 | The efficacy of an intervention for the management of postpartum fatigue | Yes | No | Yes | Yes | Yes | Yes | Exclude | Intervention component was not for depression (but for fatigue) | No | Exclude | Intervention is not for pregnancy-related depression |  |
| Goetz M,Schiele C,Müller M,Matthies LM,Deutsch TM,Spano C,Graf J,Zipfel S,Bauer A,Brucker SY,Wallwiener M,Wallwiener S | 2020 | Effects of a brief electronic mindfulness-based intervention on relieving prenatal depression and anxiety in hospitalized high-risk pregnant women: Exploratory pilot study | Yes | Yes | Yes | Yes | Yes | Yes | SR only | single group study | Yes | Include |  | Single arm studies |
| Golshani F,Hasanpour S,Mirghafourvand M,Esmaeilpour K | 2021 | Effect of cognitive behavioral therapy-based counseling on perceived stress in pregnant women with history of primary infertility: A controlled randomized clinical trial | Yes | No | Yes | Yes | Yes | Yes | Exclude | Non-digital intervention | Yes | Exclude | Non-digital intervention |  |
| Gomà M,Arias-Pujol E,Prims E,Ferrer J,Lara S,Glover V,Martinez M,Llairó A,Nanzer N | 2023 | Internet-based interdisciplinary therapeutic group (Grupo Interdisciplinar Online, GIO) for perinatal anxiety and depression-a randomized pilot study during COVID-19 | Yes | Yes | Yes | Yes | Yes | Yes | SR & MA | Double arm trial | Yes | Exclude | Poor study quality on appraisal |  |
| Gong MQ,Zhang S,Xi CH,Luo M,Wang T,Wang YX,Wang SB,Guo L,Lu CY | 2021 | Comprehensive intervention during pregnancy based on short message service to prevent or alleviate depression in pregnant women: A quasi-experimental study | Yes | Yes | Yes | Yes | Yes | Yes | SR & MA | Double arm trial | Yes | Include |  | Non-randomised trials |
| Green EP,Lai Y,Pearson N,Rajasekharan S,Rauws M,Joerin A,Kwobah E,Musyimi C,Jones RM,Bhat C,Mulinge A,Puffer ES | 2020 | Expanding Access to Perinatal Depression Treatment in Kenya Through Automated Psychological Support: Development and Usability Study | Yes | Yes | N/A | Yes | No | No | Exclude | Summary statistics on depression not reported in paper and a single-case experimental design | Yes | Exclude | Case series/report |  |
| Grote NK,Katon WJ,Lohr MJ,Carson K,Curran M,Galvin E,Russo JE,Gregory M | 2014 | Culturally relevant treatment services for perinatal depression in socio-economically disadvantaged women: The design of the MOMCare study | Yes | No | Yes | Yes | Yes | No | Exclude | Non-digital intervention and only baseline depression results were reported | No | Exclude | Non-digital intervention |  |
| Guevara P,Morales K,Mandell D,Mogul M,Charidah T,Luethke M,Min J,Clark R,Betancourt L,Boyd R | 2023 | Social Media-based Parenting Program for Women With Postpartum Depressive Symptoms: An RCT | Yes | Yes | No | Yes | Yes | Yes | Exclude | Control group received digital intervention | Yes | Exclude | Control group received digital intervention |  |
| Haga SM,Drozd F,Lisoy C,Wentzel-Larsen T,Slinning K | 2019 | Mamma Mia - A randomized controlled trial of an internet-based intervention for perinatal depression | Yes | Yes | Yes | Yes | Yes | Yes | SR & MA | Double arm trial | Yes | Include |  |  |
| Hassdenteufel K,Müller M,Abele H,Brucker SY,Graf J,Zipfel S,Bauer A,Jakubowski P,Pauluschke-Fröhlich J,Wallwiener M,Wallwiener S | 2023 | Using an Electronic Mindfulness-based Intervention (eMBI) to improve maternal mental health during pregnancy: Results from a randomized controlled trial | Yes | Yes | Yes | Yes | Yes | Yes | SR & MA | Double arm trial | Yes | Include |  |  |
| Heller HM,Hoogendoorn AW,Honig A,Broekman BF,van Straten A | 2020 | The effectiveness of a guided internet-based tool for the treatment of depression and anxiety in pregnancy (MamaKits Online): Randomized controlled trial | Yes | Yes | Yes | Yes | Yes | Yes | SR & MA | Double arm trial | Yes | Include |  |  |
| Highet N,Gamble J,Creedy D | 2018 | Perinatal mental health and psychosocial risk screening in a community maternal and child health setting: Evaluation of a digital platform | Yes | No | N/A | Yes | Yes | No | Exclude | Study's scope is on screening | No | Exclude | Study is on screening |  |
| Huang L,Shen Q,Fang Q,Zheng X | 2021 | Effects of Internet-Based Support Program on Parenting Outcomes for Primiparous Women: A Pilot Study | Yes | Yes | Yes | Yes | Yes | Yes | SR & MA | Double arm trial | Yes | Include |  |  |
| Huh K,Layton H,Savoy C,Ferro M,Bieling P,Hicks A,Lieshout R | 2023 | Online Public Health Nurse–Delivered Group Cognitive Behavioral Therapy for Postpartum Depression | Yes | Yes | Yes | Yes | Yes | Yes | SR & MA | Double arm trial | Yes | Include |  |  |
| Hulsbosch LP,Potharst ES,Schwabe I,Boekhorst MG,Pop VJ,Nyklíček I | 2023 | Online mindfulness-based intervention for women with pregnancy distress: A randomized controlled trial | Yes | Yes | Yes | Yes | Yes | Yes | SR & MA | Double arm trial | Yes | Include |  |  |
| Iloma DO,Abikoye GE,Nnam MU,Ogbonnaya CE | 2023 | Why we’re distraught: A pre and post quasi-experimental approach and interventional strategy for tackling psychological distress among perinatal women | Yes | No | N/A | Yes | Yes | Yes | Exclude | Non-digital intervention | Yes | Exclude | Non-digital intervention |  |
| Jannati N,Mazhari S,Ahmadian L,Mirzaee M | 2020 | Effectiveness of an app-based cognitive behavioral therapy program for postpartum depression in primary care: A randomized controlled trial | Yes | Yes | Yes | Yes | Yes | Yes | SR & MA | Double arm trial | Yes | Include |  |  |
| Jester JM,Riggs JL,Menke RA,Alfafara E,Issa M,Muzik M,Rosenblum KL | 2023 | Randomized pilot trial of the "Mom Power" trauma- and attachment-informed multi-family group intervention in treating and preventing postpartum symptoms of depression among a health disparity sample | No | No | Yes | Yes | Yes | Yes | Exclude | Population is women who are above 12 months postpartum | Yes | Exclude | Wrong population |  |
| Jiang L,Wang ZZ,Qiu LR,Wan GB,Lin Y,Wei Z | 2014 | Psychological intervention for postpartum depression | Yes | No | Yes | Yes | Yes | Yes | Exclude | Non-digital intervention | No | Exclude | Non-digital intervention |  |
| Jiao N,Zhu L,Chong YS,Chan WC,Luo N,Wang W,Hu R,Chan YH,He HG | 2019 | Web-based versus home-based postnatal psychoeducational interventions for first-time mothers: A randomised controlled trial | Yes | Yes | Yes | Yes | Yes | Yes | SR & MA | Double arm trial | Yes | Include |  |  |
| Jidong DE,Ike JT,Husain N,Murshed M,Francis C,Mwankon BS,Jack BD,Jidong JE,Pwajok YJ,Nyam PP,Kiran T,Bassett P | 2023 | Culturally adapted psychological intervention for treating maternal depression in British mothers of African and Caribbean origin: A randomized controlled feasibility trial | No | Yes | Yes | Yes | Yes | No | Exclude | Population is women who are above 12 months postpartum | Yes | Exclude | Wrong population |  |
| Jimah T,Borg H,Kehoe P,Pimentel P,Turner A,Labbaf S,Mehrabadi MA,Rahmani AM,Dutt N,Guo Y | 2021 | A Technology-Based Pregnancy Health and Wellness Intervention (Two Happy Hearts): Case Study | No | Yes | N/A | Yes | No | Yes | Exclude | Single case study design | Yes | Exclude | Case series/report |  |
| Kavanagh DJ,Connolly J,Fisher J,Halford WK,Hamilton K,Hides L,Milgrom J,Rowe H,Scuffham PA,White KM,Wittkowski A,Appleton S,Sanders D | 2021 | The Baby Steps Web Program for the Well-Being of New Parents: Randomized Controlled Trial | No | Yes | Yes | Yes | Yes | No | Exclude | Study (population and intervention) was for couple and not about the woman herself and summary statistics on depression not reported in paper | Yes | Exclude | Wrong population |  |
| Kingston D,Austin MP,van Zanten SV,Harvalik P,Giallo R,McDonald SD,MacQueen G,Vermeyden L,Lasiuk G,Sword W,Biringer A | 2017 | Pregnant women’s views on the feasibility and acceptability of web-based mental health e-screening versus paper-based screening: A randomized controlled trial | Yes | No | Yes | No | Yes | No | Exclude | Study's scope is on screening | No | Exclude | Study is on screening |  |
| Koire A,Erdei C,Mittal L,Wiegartz P,Liu CH | 2024 | Virtual(ly) no support: Associations between virtual support group participation and peripartum mental health outcomes during the COVID-19 pandemic | Yes | No | Yes | Yes | Yes | No | Exclude | Intervention is digital but not uniform; different people within the tested cohort received different digital interventions | Yes | Exclude | Non-uniform digital intervention within the experiment group |  |
| Krusche A,Dymond M,Murphy SE,Crane C | 2018 | Mindfulness for pregnancy: A randomised controlled study of online mindfulness during pregnancy | Yes | Yes | Yes | Yes | Yes | No | SR only | Summary statistics on depression not reported in paper | No | Exclude | Year (2014-2018) |  |
| Kubo A,Aghaee S,Kurtovich EM,Nkemere L,Quesenberry CP,McGinnis MK,Avalos LA | 2021 | mHealth Mindfulness Intervention for Women with Moderate-to-Moderately-Severe Antenatal Depressive Symptoms: a Pilot Study Within an Integrated Health Care System | Yes | Yes | N/A | Yes | Yes | Yes | SR only | single group study | Yes | Include |  | Single arm studies |
| Kuipers YJ,Bleijenbergh R,Rimaux S,Mestdagh E | 2024 | Evaluation of a web-based intervention to optimize perinatal emotional wellbeing: A nested case-control study | Yes | Yes | Yes | Yes | Yes | No | SR only | Summary statistics on depression not reported in paper | Yes | Exclude | Poor study quality on appraisal |  |
| Kurumiya Y,Garcia Y,Griffith AK,Szabo TG | 2022 | Online ACT Matrix Parent Training for Japanese-Speaking Mothers with Distress in the United States | No | Yes | N/A | Yes | Yes | Yes | Exclude | Population is women who are above 12 months postpartum | Yes | Exclude | Wrong population |  |
| Latendresse G,Bailey E,Iacob E,Murphy H,Pentecost R,Thompson N,Hogue C | 2021 | A Group Videoconference Intervention for Reducing Perinatal Depressive Symptoms: A Telehealth Pilot Study | Yes | Yes | No | Yes | Yes | Yes | Exclude | Control received digital intervention | Yes | Exclude | Control group received digital intervention |  |
| Latendresse G,Pentecost R,Iacob E,Simonsen S,Williams M,Thompson N,Hogue C | 2023 | A group telehealth intervention for rural perinatal depression and anxiety: A pilot study | Yes | Yes | N/A | Yes | Yes | Yes | SR only | single group study | Yes | Include |  | Single arm studies |
| Lawson A,Dalfen A,Murphy KE,Milligan N,Lancee W | 2019 | Use of text messaging for postpartum depression screening and information provision | Yes | No | N/A | Yes | Yes | No | Exclude | Study's scope is on screening | Yes | Exclude | Study is on screening |  |
| Lee E,Kim M | 2021 | The effects of a group cognitive behavioral therapy program using video communication for pregnant women with depressed mood in Korea: a pilot study | Yes | Yes | N/A | Yes | Yes | Yes | SR only | single group study | Yes | Include |  | Single arm studies |
| Lefever JE,Bigelow KM,Carta JJ,Borkowski JG,Grandfield E,McCune L,Irvin DW,Warren SF | 2017 | Long-Term Impact of a Cell Phone–Enhanced Parenting Intervention | No | Yes | Yes | Yes | Yes | Yes | Exclude | Population is women who are above 12 months postpartum | No | Exclude | Wrong population |  |
| Leng LL,Yin XC,Chan CL,Ng SM | 2023 | Antenatal mobile-delivered mindfulness-based intervention to reduce perinatal depression risk and improve obstetric and neonatal outcomes: A randomized controlled trial | Yes | Yes | No | Yes | Yes | No | Exclude | Control received digital intervention | Yes | Exclude | Control group received digital intervention |  |
| Lennard GR,Mitchell AE,Whittingham K | 2021 | Randomized controlled trial of a brief online self‐compassion intervention for mothers of infants: Effects on mental health outcomes | No | Yes | Yes | Yes | Yes | Yes | Exclude | Population is women who are above 12 months postpartum | Yes | Exclude | Wrong population |  |
| Letourneau N,Secco L,Colpitts J,Aldous S,Stewart M,Dennis C | 2015 | Quasi‐experimental evaluation of a telephone‐based peer support intervention for maternal depression | No | Yes | N/A | Yes | Yes | Yes | Exclude | Population is women who are above 12 months postpartum | No | Exclude | Wrong population |  |
| Liu C,Chen H,Zhou F,Long Q,Wu K,Lo LM,Hung TH,Liu CY,Chiou WK | 2022 | Positive intervention effect of mobile health application based on mindfulness and social support theory on postpartum depression symptoms of puerperae | Yes | Yes | Yes | Yes | Yes | Yes | SR & MA | Double arm trial | Yes | Exclude | Poor study quality on appraisal |  |
| Loughnan SA,Butler C,Sie AA,Grierson AB,Chen AZ,Hobbs MJ,Joubert AE,Haskelberg H,Mahoney A,Holt C,Gemmill AW,Milgrom J,Austin MP,Andrews G,Newby JM | 2019 | A randomised controlled trial of ‘MUMentum postnatal’: Internet-delivered cognitive behavioural therapy for anxiety and depression in postpartum women | Yes | Yes | Yes | Yes | Yes | Yes | SR & MA | Double arm trial | Yes | Include |  |  |
| Loughnan SA,Sie A,Hobbs MJ,Joubert AE,Smith J,Haskelberg H,Mahoney AE,Kladnitski N,Holt CJ,Milgrom J,Austin MP,Andrews G,Newby JM | 2019 | A randomized controlled trial of ‘MUMentum Pregnancy’: Internet-delivered cognitive behavioral therapy program for antenatal anxiety and depression | Yes | Yes | Yes | Yes | Yes | Yes | SR & MA | Double arm trial | Yes | Include |  |  |
| MacKinnon AL,Madsen JW,Giesbrecht GF,Campbell T,Carlson LE,Dimidjian S,Letourneau N,Tough S,Tomfohr-Madsen L | 2021 | Effects of Mindfulness-Based Cognitive Therapy in Pregnancy on Psychological Distress and Gestational Age: Outcomes of a Randomized Controlled Trial | Yes | No | Yes | Yes | Yes | No | Exclude | Non-digital intervention and summary statistics on depression not reported in paper | Yes | Exclude | Non-digital intervention |  |
| Mahoney A,Shiner CT,Grierson AB,Sharrock MJ,Loughnan SA,Harrison V,Millard M | 2023 | Online cognitive behaviour therapy for maternal antenatal and postnatal anxiety and depression in routine care | Yes | No | Yes | Yes | Yes | Yes | Exclude | Intervention is digital but not uniform; different people within the tested cohort received different digital interventions | Yes | Exclude | Non-uniform digital intervention within the experiment group |  |
| Matvienko-Sikar K,Dockray S | 2017 | Effects of a novel positive psychological intervention on prenatal stress and well-being: A pilot randomised controlled trial | Yes | Yes | Yes | Yes | Yes | Yes | SR & MA | Triple arm trial | No | Exclude | Year (2014-2018) |  |
| McCarter DE,Demidenko E,Hegel MT | 2018 | Measuring outcomes of digital technology‐assisted nursing postpartum: A randomized controlled trial | Yes | Yes | No | Yes | Yes | No | Exclude | Control received digital intervention and desired results not reported in paper | No | Exclude | Control group received digital intervention |  |
| McPherson KE,Wiseman K,Jasilek A,McAloney-Kocaman K,Morawska A,Haig C | 2022 | Baby Triple P: A Randomized Controlled Trial Testing the Efficacy in First-Time Parent Couples | No | Yes | Yes | Yes | Yes | Yes | Exclude | Study (population and intervention) was for couple and not about the woman herself | Yes | Exclude | Wrong population |  |
| Merza D,Amani B,Savoy C,Babiy Z,Bieling PJ,Streiner DL,Ferro MA,Van Lieshout RJ | 2023 | Online peer‐delivered group cognitive‐behavioral therapy for postpartum depression: A randomized controlled trial | Yes | Yes | Yes | Yes | Yes | Yes | SR & MA | Double arm trial | Yes | Include |  |  |
| Milani HS,Azargashb E,Beyraghi N,Defaie S,Asbaghi T | 2015 | Effect of telephone-based support on postpartum depression: A randomized controlled trial | Yes | Yes | Yes | Yes | Yes | Yes | SR & MA | Double arm trial | No | Exclude | Year (2014-2018) |  |
| Miles A,Lovell M,Ibrahim R,Dagli TE,Dagli FS,Sethna DV | 2023 | A feasibility study of Online Mellow Bumps: A Turkish pilot study of an online group-based antenatal parenting intervention | Yes | Yes | N/A | Yes | Yes | Yes | SR only | single group study | Yes | Include |  | Single arm studies |
| Milgrom J,Danaher BG,Gemmill AW,Holt C,Holt CJ,Seeley JR,Tyler MS,Ross J,Ericksen J | 2016 | Internet Cognitive Behavioral Therapy for Women With Postnatal Depression: A Randomized Controlled Trial of MumMoodBooster | Yes | Yes | Yes | Yes | Yes | Yes | SR & MA | Double arm trial | No | Exclude | Year (2014-2018) |  |
| Milgrom J,Danaher BG,Seeley JR,Holt CJ,Holt C,Ericksen J,Tyler MS,Gau JM,Gemmill AW | 2021 | Internet and Face-to-face Cognitive Behavioral Therapy for Postnatal Depression Compared With Treatment as Usual: Randomized Controlled Trial of MumMoodBooster | Yes | Yes | Yes | Yes | Yes | Yes | SR & MA | Triple arm trial | Yes | Include |  |  |
| Missler M,van Straten A,Denissen J,Donker T,Beijers R | 2020 | Effectiveness of a psycho-educational intervention for expecting parents to prevent postpartum parenting stress, depression and anxiety: a randomized controlled trial | No | Yes | Yes | Yes | Yes | Yes | Exclude | Study (population and intervention) was for couple and not about the woman herself | Yes | Exclude | Wrong population |  |
| Monteiro F,Pereira M,Canavarro MC,Fonseca A | 2020 | Be a Mom’s Efficacy in Enhancing Positive Mental Health among Postpartum Women Presenting Low Risk for Postpartum Depression: Results from a Pilot Randomized Trial | Yes | Yes | Yes | Yes | Yes | Yes | SR & MA | Double arm trial | Yes | Include |  |  |
| Mundorf C,Shankar A,Moran T,Heller S,Hassan A,Harville E,Lichtveld M | 2018 | Reducing the Risk of Postpartum Depression in a Low-Income Community Through a Community Health Worker Intervention | Yes | No | Yes | Yes | Yes | Yes | Exclude | Non-digital intervention | No | Exclude | Non-digital intervention |  |
| Naja S,Elyamani R,Chehab M,Ahmed MA,Babeker G,Lawand G,Singh R,Adli N,Mohamad T,Bougmiza I | 2023 | The impact of telemental health interventions on maternal mental health outcomes: a pilot randomized controlled trial during the COVID-19 pandemic | Yes | Yes | Yes | Yes | Yes | Yes | SR & MA | Double arm trial | Yes | Include |  | Insufficient reported data for meta-analysis |
| Nakku JE,Nalwadda O,Garman E,Honikman S,Hanlon C,Kigozi F,Lund C | 2021 | Group problem solving therapy for perinatal depression in primary health care settings in rural Uganda: an intervention cohort study | Yes | No | N/A | Yes | Yes | Yes | Exclude | Non-digital intervention | Yes | Exclude | Non-digital intervention |  |
| Nejad FK,Shahraki KA,Nejad PS,Moghaddam NK,Jahani Y,Divsalar P | 2021 | The influence of mindfulness-based stress reduction (MBSR) on stress, anxiety and depression due to unwanted pregnancy: a randomized clinical trial | Yes | No | Yes | Yes | Yes | Yes | Exclude | Non-digital intervention | Yes | Exclude | Non-digital intervention |  |
| Nieminen K,Berg I,Frankenstein K,Viita L,Larsson K,Persson U,Spånberger L,Wretman A,Silfvernagel K,Andersson G,Wijma K | 2016 | Internet-provided cognitive behaviour therapy of posttraumatic stress symptoms following childbirth—a randomized controlled trial | Yes | No | Yes | Yes | Yes | Yes | Exclude | Intervention component was not for depression (but for trauma) | No | Exclude | Intervention is not for pregnancy-related depression |  |
| Niksalehi S,Taghadosi M,Mazhariazad F,Tashk M | 2018 | The effectiveness of mobile phone text massaging support for mothers with postpartum depression: A clinical before and after study | Yes | Yes | N/A | Yes | Yes | Yes | SR only | single group study | No | Exclude | Year (2014-2018) |  |
| Nishi D,Imamura K,Watanabe K,Obikane E,Sasaki N,Yasuma N,Sekiya Y,Matsuyama Y,Kawakami N | 2022 | The preventive effect of internet‐based cognitive behavioral therapy for prevention of depression during pregnancy and in the postpartum period ( iPDP ): a large scale randomized controlled trial | Yes | Yes | Yes | Yes | Yes | Yes | SR & MA | Double arm trial | Yes | Include |  |  |
| Obichili MI,Ogwo CA,Udeh K,Obiechina CK,Kakwagh VV,Eze CC,Gever VC | 2023 | Effect of social media-based psychodrama therapy on reduction in symptoms of postpartum depression in women with first birth experience: The contributing role of spousal support | No | Yes | N/A | Yes | Yes | No | Exclude | Study (population and intervention) was for couples and not about the woman herself and no summary statistics on depression | Yes | Exclude | Wrong population |  |
| O'Mahen HA,Richards DA,Woodford J,Wilkinson E,McGinley J,Taylor RS,Warren FC | 2014 | Netmums: a phase II randomized controlled trial of a guided Internet behavioural activation treatment for postpartum depression | Yes | Yes | Yes | Yes | Yes | Yes | SR & MA | Double arm trial | No | Exclude | Year (2014-2018) |  |
| Ortiz Collado MA,Saez M,Favrod J,Hatem M | 2014 | Antenatal psychosomatic programming to reduce postpartum depression risk and improve childbirth outcomes: a randomized controlled trial in Spain and France | No | No | Yes | Yes | Yes | Yes | Exclude | Study (population and intervention) was for couples and not about the woman herself | No | Exclude | Wrong population |  |
| O'Shea A,Kaplan K,Solomon P,Salzer MS | 2019 | Randomized Controlled Trial of an Internet-Based Educational Intervention for Mothers With Mental Illnesses: An 18-Month Follow-Up | No | Yes | Yes | No | Yes | No | Exclude | Population is women who are above 12 months postpartum and included women with vast range of mental illnesses | Yes | Exclude | Wrong population |  |
| Pan Q,Huang Y,Duan B | 2021 | EFFECTS OF HEALTH EDUCATION BASED ON OMAHA SYSTEM ON ANXIETY, DEPRESSION AND SELF-MANAGEMENT ABILITY OF PRIMIPARA | Yes | No | Yes | Yes | Yes | Yes | Exclude | Non-digital intervention | Yes | Exclude | Non-digital intervention |  |
| Patil M,Malhotra J | 2021 | Mindful Digital Program–based Interventions and their Role in Pregnancy and Fetal Outcomes | Yes | No | Yes | No | Yes | No | Exclude | Non-digital intervention and no report on depression measurements | Yes | Exclude | Non-digital intervention |  |
| Peifer JS,Bradley E,Taasoobshirazi G | 2022 | Pilot Testing a Brief Partner-Inclusive Hybrid Intervention for Perinatal Mood and Anxiety Disorders | No | No | N/A | Yes | Yes | Yes | Exclude | Study (population and intervention) was for couples and not about the woman herself and the intervention was non-digital | Yes | Exclude | Wrong population |  |
| Perkins R,Spiro N,Waddell G | 2023 | Online songwriting reduces loneliness and postnatal depression and enhances social connectedness in women with young babies: randomised controlled trial | Yes | No | Yes | Yes | Yes | Yes | Exclude | Component of the intervention is not a validated management option for perinatal depression | Yes | Exclude | Component of the intervention is not a validated management option for perinatal depression |  |
| Platt R,Martin CP,Perry O,Cooper L,Tandon D,Richman R,Bettencourt AF,Polk S | 2023 | A Mixed-Methods Evaluation of Virtually Delivered Group-Based Mothers and Babies for Latina Immigrant Mothers | No | Yes | N/A | Yes | Yes | No | Exclude | Population is women who are above 12 months postpartum and no reported summary statistics on depression | Yes | Exclude | Wrong population |  |
| Porte LM,Kim JJ,Adams MG,Zagorsky BM,Gibbons R,Silver RK | 2020 | Feasibility of perinatal mood screening and text messaging on patients’ personal smartphones | Yes | No | N/A | Yes | Yes | No | Exclude | Study's scope is on screening | Yes | Exclude | Study is on screening |  |
| Porter AC,Hunter S,Noonan K,Hoffman MC | 2022 | A Mindfulness Application for Reducing Prenatal Stress | Yes | Yes | Yes | No | Yes | No | Exclude | No report on depression measurements | Yes | Exclude | No reported depression measurements |  |
| Posmontier B,Neugebauer R,Stuart S,Chittams J,Shaughnessy R | 2016 | Telephone‐Administered Interpersonal Psychotherapy by Nurse‐Midwives for Postpartum Depression | Yes | Yes | Yes | Yes | Yes | Yes | SR & MA | Double arm trial | No | Exclude | Year (2014-2018) |  |
| Potharst ES,Boekhorst MG,Cuijlits I,van Broekhoven KE,Jacobs A,Spek V,Nyklíček I,Bögels SM,Pop VJ | 2019 | A Randomized Control Trial Evaluating an Online Mindful Parenting Training for Mothers With Elevated Parental Stress | No | No | Yes | Yes | Yes | No | Exclude | Population is women who are above 12 months postpartum and intervention was designed for mothers with toddlers. No summary statistic for depression | Yes | Exclude | Wrong population |  |
| Potharst ES,Schaeffer MA,Gunning C,de Lara MC,Boekhorst MG,Hulsbosch LP,Pop VJ,Duijff SN | 2022 | Implementing “Online Communities” for pregnant women in times of COVID-19 for the promotion of maternal well-being and mother-to-infant bonding: a pretest–posttest study | Yes | No | N/A | Yes | Yes | Yes | Exclude | Intervention is not primarily for perinatal depression but rather COVID-19 induced depressive symptoms in pregnant women | Yes | Exclude | Intervention is not for pregnancy-related depression |  |
| Powell C,Bamber D,Long J,Garratt R,Brown J,Rudge S,Morris T,Jaicim NB,Plachcinski R,Dyson S,Boyle EM,James‐Roberts IS | 2018 | Mental health and well‐being in parents of excessively crying infants: Prospective evaluation of a support package | Yes | No | Yes | Yes | Yes | Yes | Exclude | Non-digital intervention and intervention component was designed for mothers with babies who cry excessively. | No | Exclude | Non-digital intervention |  |
| Puertas-Gonzalez JA,Mariño-Narvaez C,Romero-Gonzalez B,Sanchez-Perez GM,Peralta-Ramirez MI | 2022 | Online cognitive behavioural therapy as a psychological vaccine against stress during the COVID-19 pandemic in pregnant women: A randomised controlled trial | Yes | Yes | Yes | No | Yes | No | Exclude | No report on depression measurements | Yes | Exclude | No reported depression measurements |  |
| Pugh NE,Hadjistavropoulos HD,Dirkse D | 2016 | A Randomised Controlled Trial of Therapist-Assisted, Internet-Delivered Cognitive Behavior Therapy for Women with Maternal Depression | Yes | Yes | Yes | Yes | Yes | Yes | SR & MA | Double arm trial | No | Exclude | Year (2014-2018) |  |
| Qin X,Liu C,Zhu W,Chen Y,Wang Y | 2022 | Preventing Postpartum Depression in the Early Postpartum Period Using an App-Based Cognitive Behavioral Therapy Program: A Pilot Randomized Controlled Study | Yes | Yes | Yes | Yes | Yes | Yes | SR & MA | Double arm trial | Yes | Include |  | Insufficient reported data for meta-analysis |
| Salonen AH,Pridham KF,Brown RL,Kaunonen M | 2014 | Impact of an internet-based intervention on Finnish mothers' perceptions of parenting satisfaction, infant centrality and depressive symptoms during the postpartum year | Yes | Yes | Yes | Yes | Yes | Yes | SR & MA | Double arm trial | No | Exclude | Year (2014-2018) |  |
| Sapkota D,Baird K,Saito A,Rijal P,Anderson D | 2022 | Antenatal-Based Pilot Psychosocial Intervention to Enhance Mental Health of Pregnant Women Experiencing Domestic and Family Violence in Nepal | Yes | No | Yes | Yes | Yes | Yes | Exclude | Non-digital intervention | Yes | Exclude | Non-digital intervention |  |
| Sawyer A,Kaim A,Le HN,McDonald D,Mittinty M,Lynch J,Sawyer M | 2019 | The Effectiveness of an App-Based Nurse-Moderated Program for New Mothers With Depression and Parenting Problems (eMums Plus): Pragmatic Randomized Controlled Trial | Yes | Yes | Yes | Yes | Yes | Yes | SR & MA | Double arm trial | Yes | Include |  |  |
| Sawyer MG,Reece CE,Bowering K,Jeffs D,Sawyer AC,Mittinty M,Lynch JW | 2017 | Nurse-Moderated Internet-Based Support for New Mothers: Non-Inferiority, Randomized Controlled Trial | Yes | Yes | Yes | No | Yes | No | Exclude | No report on depression measurements | No | Exclude | No reported depression measurements |  |
| Schwartz H,McCusker J,Costa D,Singh S,Baskaran S,Belzile E,Van Roost K | 2023 | A pilot randomized controlled trial of a lay telephone coaching and web-based intervention for postpartum depression and anxiety: The MPOWER study | Yes | Yes | No | Yes | Yes | Yes | Exclude | Control group received digital intervention | Yes | Exclude | Control group received digital intervention |  |
| Seo JM,Kim SJ,Na H,Kim JH,Lee H | 2022 | Effectiveness of a mobile application for postpartum depression self-management: Evidence from a randomised controlled trial in South Korea | Yes | Yes | Yes | Yes | Yes | Yes | SR & MA | Double arm trial | Yes | Include |  |  |
| Shahsavan F,Akbari N,Gharraee B,Abolghasemi J,Khedmat L | 2021 | The effect of internet‐based guided self‐help cognitive‐behavioral therapies on Iranian women's psychological symptoms and preferred method of childbirth | Yes | Yes | Yes | Yes | Yes | Yes | SR only | Quasi-experimental study | Yes | Include |  | Non-randomised trials |
| Shariatpanahi G,Effatpanah M,Moienafshar A,Shariati M,Kheiltash A,Ahadpourkhanghah E,Khaneghah AA | 2023 | Comparing the Effectiveness of Internet-Based Cognitive Behavioral Therapy and Drug Therapy for Treating Postpartum Depression and Children Weight Gain: A Randomized Clinical Trial | Yes | Yes | Yes | Yes | Yes | Yes | SR & MA | Double arm trial | Yes | Exclude | Poor study quality on appraisal |  |
| Sheeber LB,Feil EG,Seeley JR,Leve C,Gau JM,Davis B,Sorensen E,Allan S | 2017 | Mom-net: Evaluation of an internet-facilitated cognitive behavioral intervention for low-income depressed mothers | No | Yes | Yes | Yes | Yes | Yes | Exclude | Population is women who are above 12 months postpartum | No | Exclude | Wrong population |  |
| Shorey S,Chee CY,Ng ED,Lau Y,Dennis CL,Chan YH | 2019 | Evaluation of a Technology-Based Peer-Support Intervention Program for Preventing Postnatal Depression (Part 1): Randomized Controlled Trial | Yes | Yes | Yes | Yes | Yes | Yes | SR only | Baseline depression results not reported | Yes | Include |  | Baseline depression results not reported |
| Shorey S,Lau Y,Dennis C,Chan YS,Tam WW,Chan YH | 2017 | A randomized‐controlled trial to examine the effectiveness of the ‘Home‐but not Alone’ mobile‐health application educational programme on parental outcomes | No | Yes | Yes | Yes | Yes | Yes | Exclude | Study (population and intervention) was for couples and not about the woman herself | No | Exclude | Wrong population |  |
| Shorey S,Ng YP,Ng ED,Siew AL,Mörelius E,Yoong J,Gandhi M | 2019 | Effectiveness of a Technology-Based Supportive Educational Parenting Program on Parental Outcomes (Part 1): Randomized Controlled Trial | No | Yes | Yes | Yes | Yes | Yes | Exclude | Study (population and intervention) was for couples and not about the woman herself | Yes | Exclude | Wrong population |  |
| Shulman B,Dueck R,Ryan D,Breau G,Sadowski I,Misri S | 2018 | Feasibility of a mindfulness-based cognitive therapy group intervention as an adjunctive treatment for postpartum depression and anxiety | Yes | No | Yes | Yes | Yes | Yes | Exclude | Non-digital intervention | No | Exclude | Non-digital intervention |  |
| Sjömark J,Svanberg AS,Larsson M,Viirman F,Poromaa IS,Skalkidou A,Jonsson M,Parling T | 2022 | Effect of internet-based cognitive behaviour therapy among women with negative birth experiences on mental health and quality of life - a randomized controlled trial | No | Yes | Yes | Yes | Yes | Yes | Exclude | Study (population and intervention) was for couples and not about the woman herself | Yes | Exclude | Wrong population |  |
| Solness CL,Kroska EB,Holdefer PJ,O'Hara MW | 2021 | Treating postpartum depression in rural veterans using internet delivered CBT: program evaluation of MomMoodBooster | No | Yes | N/A | Yes | Yes | Yes | Exclude | Population is women who are above 12 months postpartum | Yes | Exclude | Wrong population |  |
| Suchan V,Peynenburg V,Thiessen D,Nugent M,Dear B,Titov N,Hadjistavropoulos H | 2022 | Transdiagnostic Internet-Delivered Cognitive Behavioral Therapy for Symptoms of Postpartum Anxiety and Depression: Feasibility Randomized Controlled Trial | Yes | Yes | Yes | Yes | Yes | Yes | SR & MA | Double arm trial | Yes | Include |  |  |
| Suharwardy S,Ramachandran M,Leonard SA,Gunaseelan A,Lyell DJ,Darcy A,Robinson A,Judy A | 2023 | Feasibility and impact of a mental health chatbot on postpartum mental health: a randomized controlled trial | Yes | Yes | Yes | Yes | Yes | Yes | SR & MA | Double arm trial | Yes | Include |  |  |
| Sun Y,Li Y,Wang J,Chen Q,Bazzano AN,Cao F | 2021 | Effectiveness of Smartphone-Based Mindfulness Training on Maternal Perinatal Depression: Randomized Controlled Trial | Yes | Yes | No | Yes | Yes | Yes | Exclude | Control group received digital intervention | Yes | Exclude | Control group received digital intervention |  |
| Thomas N,Komiti A,Judd F | 2014 | Pilot early intervention antenatal group program for pregnant women with anxiety and depression | Yes | No | N/A | Yes | Yes | Yes | Exclude | Non-digital intervention | No | Exclude | Non-digital intervention |  |
| Trevillion K,Ryan EG,Pickles A,Heslin M,Byford S,Nath S,Bick D,Milgrom J,Mycroft R,Domoney J,Pariante C,Hunter MS,Howard LM | 2020 | An exploratory parallel-group randomised controlled trial of antenatal Guided Self-Help (plus usual care) versus usual care alone for pregnant women with depression: DAWN trial | Yes | No | Yes | Yes | Yes | Yes | Exclude | Non-digital intervention | Yes | Exclude | Non-digital intervention |  |
| Tsai Y,Hsu Y,Hou T,Chang C | 2018 | Effects of a Web‐Based Antenatal Care System on Maternal Stress and Self‐Efficacy During Pregnancy: A Study in Taiwan | Yes | No | Yes | No | Yes | No | Exclude | Intervention component was not on depression (but on antenatal) and hence, no report on depression measurements | No | Exclude | Intervention is not for pregnancy-related depression |  |
| Van Lieshout RJ,Layton H,Savoy CD,Brown JS,Ferro MA,Streiner DL,Bieling PJ,Feller A,Hanna S | 2021 | Effect of Online 1-Day Cognitive Behavioral Therapy–Based Workshops Plus Usual Care vs Usual Care Alone for Postpartum Depression | Yes | Yes | Yes | Yes | Yes | Yes | SR & MA | Double arm trial | Yes | Include |  |  |
| Vigod SN,Cook GS,Macdonald K,Hussain‐Shamsy N,Brown HK,Oliveira C,Torshizi K,Benipal PK,Grigoriadis S,Classen CC,Dennis C | 2021 | Mother Matters: Pilot randomized wait‐list controlled trial of an online therapist‐facilitated discussion board and support group for postpartum depression symptoms | Yes | Yes | Yes | Yes | Yes | Yes | SR & MA | Double arm trial | Yes | Include |  | Insufficient reported data for meta-analysis |
| Wozney L,Olthuis J,Lingley-Pottie P,McGrath PJ,Chaplin W,Elgar F,Cheney B,Huguet A,Turner K,Kennedy J | 2017 | Strongest Families™ Managing Our Mood (MOM): A randomized controlled trial of a distance intervention for women with postpartum depression | Yes | Yes | Yes | Yes | Yes | No | SR only | Summary statistics on depression not reported | No | Exclude | Year (2014-2018) |  |
| Wu W,Hung C | 2019 | Impact of a peer virtual community on pregnant women's well‐being: A repeated‐measure and quasi‐experimental study | Yes | Yes | Yes | Yes | Yes | No | SR only | Summary statistics on depression not reported | Yes | Include |  | Summary statistics on depression not reported in paper |
| Xie EB,Freeman M,Penner-Goeke L,Reynolds K,Lebel C,Giesbrecht GF,Rioux C,MacKinnon A,Sauer-Zavala S,Roos LE,Tomfohr-Madsen L | 2023 | Building Emotional Awareness and Mental Health (BEAM): an open-pilot and feasibility study of a digital mental health and parenting intervention for mothers of infants | No | Yes | N/A | Yes | Yes | Yes | Exclude | Population is women who are above 12 months postpartum | Yes | Exclude | Wrong population |  |
| Yang M,Jia G,Sun S,Ye C,Zhang R,Yu X | 2019 | Effects of an Online Mindfulness Intervention Focusing on Attention Monitoring and Acceptance in Pregnant Women: A Randomized Controlled Trial | Yes | Yes | Yes | Yes | Yes | Yes | SR & MA | Double arm trial | Yes | Include |  | Insufficient reported data for meta-analysis |
| Yang R,Vigod SN,Hensel JM | 2019 | Optional Web-Based Videoconferencing Added to Office-Based Care for Women Receiving Psychotherapy During the Postpartum Period: Pilot Randomized Controlled Trial | Yes | Yes | Yes | Yes | Yes | Yes | SR & MA | Double arm trial | Yes | Exclude | Poor study quality on appraisal |  |
| Yang X,Li L,Zhou R,Xia J,Li M,Zhang C,Guo H | 2023 | Effects of the online and offline hybrid continuous group care on maternal and infant health: a randomized controlled trial | Yes | Yes | Yes | Yes | Yes | No | SR only | Summary statistics on depression not reported and study is a double arm quasi-experimental although claimed to be an RCT | Yes | Exclude | Poor study quality on appraisal |  |
| Zhang X,Li Y,Wang J,Mao F,Wu L,Huang Y,Sun J,Cao F | 2023 | Effectiveness of Digital Guided Self-help Mindfulness Training During Pregnancy on Maternal Psychological Distress and Infant Neuropsychological Development: Randomized Controlled Trial | Yes | Yes | Yes | Yes | Yes | Yes | SR & MA | Double arm trial | Yes | Include |  |  |
| Zhang X,Lin P,Sun J,Sun Y,Shao D,Cao D,Cao F | 2023 | Prenatal stress self-help mindfulness intervention via social media: a randomized controlled trial | Yes | Yes | No | Yes | Yes | Yes | Exclude | Control group received digital intervention | Yes | Exclude | Control group received digital intervention |  |
| Zhang Y,Zhu J,Li S,Huang L,Fang Q,Zheng X | 2023 | The effectiveness of an internet-based support program on maternal self-efficacy, postpartum depression and social support for primiparous women during the COVID-19 pandemic: Randomized controlled trial | Yes | Yes | No | Yes | Yes | Yes | Exclude | Control group received digital intervention | Yes | Exclude | Control group received digital intervention |  |

# SUPPLEMENTARY FILE – DETAILED STEP BY STEP SCREENING OF PAPERS FOR ELIGIBLE PAPERS

# SUPPLEMENTARY FILE – POPULATED RISK OF BIAS ASSESSMENT FOR STUDIES INCLUDED IN THE META-ANALYSES

| **Unique ID** | Abujilban 2023 | **Study ID** |  | **Assessor** | I. S. Anyanwu |
| --- | --- | --- | --- | --- | --- |
| **Ref or Label** |  | **Aim** | assignment to intervention (the 'intention-to-treat' effect) |  |  |
| **Experimental** |  | **Comparator** |  | **Source** | Journal article(s) |
| **Outcome** |  | **Results** |  | **Weight** | 1 |
| **Domain** | **Signalling question** | | | **Response** | **Comments** |
| **Bias arising from the randomization process** | 1.1 Was the allocation sequence random? | | | Y |  |
|  | 1.2 Was the allocation sequence concealed until participants were enrolled and assigned to interventions? | | | Y |  |
|  | 1.3 Did baseline differences between intervention groups suggest a problem with the randomization process? | | | N |  |
|  | **Risk of bias judgement** | | | **Low** |  |
| **Bias due to deviations from intended interventions** | 2.1.Were participants aware of their assigned intervention during the trial? | | | NI |  |
|  | 2.2.Were carers and people delivering the interventions aware of participants' assigned intervention during the trial? | | | Y |  |
|  | 2.3. If Y/PY/NI to 2.1 or 2.2: Were there deviations from the intended intervention that arose because of the experimental context? | | | N |  |
|  | 2.4 If Y/PY to 2.3: Were these deviations likely to have affected the outcome? | | | NA |  |
|  | 2.5. If Y/PY/NI to 2.4: Were these deviations from intended intervention balanced between groups? | | | NA |  |
|  | 2.6 Was an appropriate analysis used to estimate the effect of assignment to intervention? | | | Y |  |
|  | 2.7 If N/PN/NI to 2.6: Was there potential for a substantial impact (on the result) of the failure to analyse participants in the group to which they were randomized? | | | NA |  |
|  | **Risk of bias judgement** | | | **Low** |  |
| **Bias due to missing outcome data** | 3.1 Were data for this outcome available for all, or nearly all, participants randomized? | | | Y |  |
|  | 3.2 If N/PN/NI to 3.1: Is there evidence that result was not biased by missing outcome data? | | | NA |  |
|  | 3.3 If N/PN to 3.2: Could missingness in the outcome depend on its true value? | | | NA |  |
|  | 3.4 If Y/PY/NI to 3.3: Is it likely that missingness in the outcome depended on its true value? | | | NA |  |
|  | **Risk of bias judgement** | | | **Low** |  |
| **Bias in measurement of the outcome** | 4.1 Was the method of measuring the outcome inappropriate? | | | N |  |
|  | 4.2 Could measurement or ascertainment of the outcome have differed between intervention groups? | | | N |  |
|  | 4.3 Were outcome assessors aware of the intervention received by study participants? | | | NI |  |
|  | 4.4 If Y/PY/NI to 4.3: Could assessment of the outcome have been influenced by knowledge of intervention received? | | | Y |  |
|  | 4.5 If Y/PY/NI to 4.4: Is it likely that assessment of the outcome was influenced by knowledge of intervention received? | | | N |  |
|  | **Risk of bias judgement** | | | **Some concerns** |  |
| **Bias in selection of the reported result** | 5.1 Were the data that produced this result analysed in accordance with a pre-specified analysis plan that was finalized before unblinded outcome data were available for analysis? | | | Y |  |
|  | 5.2 ... multiple eligible outcome measurements (e.g. scales, definitions, time points) within the outcome domain? | | | N |  |
|  | 5.3 ... multiple eligible analyses of the data? | | | N |  |
|  | **Risk of bias judgement** | | | **Low** |  |
| **Overall bias** | **Risk of bias judgement** | | | **Some concerns** |  |
|  |  |  |  |  |  |
|  |  |  |  |  |  |
| **Unique ID** | Asadzadeh 2020 | **Study ID** |  | **Assessor** | I. S. Anyanwu |
| **Ref or Label** |  | **Aim** | assignment to intervention (the 'intention-to-treat' effect) |  |  |
| **Experimental** |  | **Comparator** |  | **Source** | Journal article(s); Non-commercial trial registry record (e.g. ClinicalTrials.gov record) |
| **Outcome** |  | **Results** |  | **Weight** | 1 |
| **Domain** | **Signalling question** | | | **Response** | **Comments** |
| **Bias arising from the randomization process** | 1.1 Was the allocation sequence random? | | | Y |  |
|  | 1.2 Was the allocation sequence concealed until participants were enrolled and assigned to interventions? | | | NI |  |
|  | 1.3 Did baseline differences between intervention groups suggest a problem with the randomization process? | | | N |  |
|  | **Risk of bias judgement** | | | **Low** |  |
| **Bias due to deviations from intended interventions** | 2.1.Were participants aware of their assigned intervention during the trial? | | | Y |  |
|  | 2.2.Were carers and people delivering the interventions aware of participants' assigned intervention during the trial? | | | Y |  |
|  | 2.3. If Y/PY/NI to 2.1 or 2.2: Were there deviations from the intended intervention that arose because of the experimental context? | | | N |  |
|  | 2.4 If Y/PY to 2.3: Were these deviations likely to have affected the outcome? | | | NA |  |
|  | 2.5. If Y/PY/NI to 2.4: Were these deviations from intended intervention balanced between groups? | | | NA |  |
|  | 2.6 Was an appropriate analysis used to estimate the effect of assignment to intervention? | | | Y |  |
|  | 2.7 If N/PN/NI to 2.6: Was there potential for a substantial impact (on the result) of the failure to analyse participants in the group to which they were randomized? | | | NA |  |
|  | **Risk of bias judgement** | | | **Low** |  |
| **Bias due to missing outcome data** | 3.1 Were data for this outcome available for all, or nearly all, participants randomized? | | | Y |  |
|  | 3.2 If N/PN/NI to 3.1: Is there evidence that result was not biased by missing outcome data? | | | NA |  |
|  | 3.3 If N/PN to 3.2: Could missingness in the outcome depend on its true value? | | | NA |  |
|  | 3.4 If Y/PY/NI to 3.3: Is it likely that missingness in the outcome depended on its true value? | | | NA |  |
|  | **Risk of bias judgement** | | | **Low** |  |
| **Bias in measurement of the outcome** | 4.1 Was the method of measuring the outcome inappropriate? | | | N |  |
|  | 4.2 Could measurement or ascertainment of the outcome have differed between intervention groups? | | | N |  |
|  | 4.3 Were outcome assessors aware of the intervention received by study participants? | | | N |  |
|  | 4.4 If Y/PY/NI to 4.3: Could assessment of the outcome have been influenced by knowledge of intervention received? | | | NA |  |
|  | 4.5 If Y/PY/NI to 4.4: Is it likely that assessment of the outcome was influenced by knowledge of intervention received? | | | NA |  |
|  | **Risk of bias judgement** | | | **Low** |  |
| **Bias in selection of the reported result** | 5.1 Were the data that produced this result analysed in accordance with a pre-specified analysis plan that was finalized before unblinded outcome data were available for analysis? | | | Y |  |
|  | 5.2 ... multiple eligible outcome measurements (e.g. scales, definitions, time points) within the outcome domain? | | | N |  |
|  | 5.3 ... multiple eligible analyses of the data? | | | N |  |
|  | **Risk of bias judgement** | | | **Low** |  |
| **Overall bias** | **Risk of bias judgement** | | | **Low** |  |
|  |  |  |  |  |  |
|  |  |  |  |  |  |
| **Unique ID** | Boyd 2019 | **Study ID** |  | **Assessor** | I. S. Anyanwu |
| **Ref or Label** |  | **Aim** | assignment to intervention (the 'intention-to-treat' effect) |  |  |
| **Experimental** |  | **Comparator** |  | **Source** | Journal article(s); Non-commercial trial registry record (e.g. ClinicalTrials.gov record) |
| **Outcome** |  | **Results** |  | **Weight** | 1 |
| **Domain** | **Signalling question** | | | **Response** | **Comments** |
| **Bias arising from the randomization process** | 1.1 Was the allocation sequence random? | | | Y |  |
|  | 1.2 Was the allocation sequence concealed until participants were enrolled and assigned to interventions? | | | Y |  |
|  | 1.3 Did baseline differences between intervention groups suggest a problem with the randomization process? | | | N |  |
|  | **Risk of bias judgement** | | | **Low** |  |
| **Bias due to deviations from intended interventions** | 2.1.Were participants aware of their assigned intervention during the trial? | | | PY |  |
|  | 2.2.Were carers and people delivering the interventions aware of participants' assigned intervention during the trial? | | | PY |  |
|  | 2.3. If Y/PY/NI to 2.1 or 2.2: Were there deviations from the intended intervention that arose because of the experimental context? | | | N |  |
|  | 2.4 If Y/PY to 2.3: Were these deviations likely to have affected the outcome? | | | NA |  |
|  | 2.5. If Y/PY/NI to 2.4: Were these deviations from intended intervention balanced between groups? | | | NA |  |
|  | 2.6 Was an appropriate analysis used to estimate the effect of assignment to intervention? | | | Y |  |
|  | 2.7 If N/PN/NI to 2.6: Was there potential for a substantial impact (on the result) of the failure to analyse participants in the group to which they were randomized? | | | NA |  |
|  | **Risk of bias judgement** | | | **Low** |  |
| **Bias due to missing outcome data** | 3.1 Were data for this outcome available for all, or nearly all, participants randomized? | | | N |  |
|  | 3.2 If N/PN/NI to 3.1: Is there evidence that result was not biased by missing outcome data? | | | PN |  |
|  | 3.3 If N/PN to 3.2: Could missingness in the outcome depend on its true value? | | | N |  |
|  | 3.4 If Y/PY/NI to 3.3: Is it likely that missingness in the outcome depended on its true value? | | | NA |  |
|  | **Risk of bias judgement** | | | **Low** |  |
| **Bias in measurement of the outcome** | 4.1 Was the method of measuring the outcome inappropriate? | | | N |  |
|  | 4.2 Could measurement or ascertainment of the outcome have differed between intervention groups? | | | N |  |
|  | 4.3 Were outcome assessors aware of the intervention received by study participants? | | | PY |  |
|  | 4.4 If Y/PY/NI to 4.3: Could assessment of the outcome have been influenced by knowledge of intervention received? | | | PY |  |
|  | 4.5 If Y/PY/NI to 4.4: Is it likely that assessment of the outcome was influenced by knowledge of intervention received? | | | PN |  |
|  | **Risk of bias judgement** | | | **Some concerns** |  |
| **Bias in selection of the reported result** | 5.1 Were the data that produced this result analysed in accordance with a pre-specified analysis plan that was finalized before unblinded outcome data were available for analysis? | | | Y |  |
|  | 5.2 ... multiple eligible outcome measurements (e.g. scales, definitions, time points) within the outcome domain? | | | N |  |
|  | 5.3 ... multiple eligible analyses of the data? | | | N |  |
|  | **Risk of bias judgement** | | | **Low** |  |
| **Overall bias** | **Risk of bias judgement** | | | **Some concerns** |  |
|  |  |  |  |  |  |
|  |  |  |  |  |  |
| **Unique ID** | Carona 2023 | **Study ID** |  | **Assessor** | I. S. Anyanwu |
| **Ref or Label** |  | **Aim** | assignment to intervention (the 'intention-to-treat' effect) |  |  |
| **Experimental** |  | **Comparator** |  | **Source** | Journal article(s); Non-commercial trial registry record (e.g. ClinicalTrials.gov record) |
| **Outcome** |  | **Results** |  | **Weight** | 1 |
| **Domain** | **Signalling question** | | | **Response** | **Comments** |
| **Bias arising from the randomization process** | 1.1 Was the allocation sequence random? | | | Y |  |
|  | 1.2 Was the allocation sequence concealed until participants were enrolled and assigned to interventions? | | | Y |  |
|  | 1.3 Did baseline differences between intervention groups suggest a problem with the randomization process? | | | N |  |
|  | **Risk of bias judgement** | | | **Low** |  |
| **Bias due to deviations from intended interventions** | 2.1.Were participants aware of their assigned intervention during the trial? | | | Y |  |
|  | 2.2.Were carers and people delivering the interventions aware of participants' assigned intervention during the trial? | | | N |  |
|  | 2.3. If Y/PY/NI to 2.1 or 2.2: Were there deviations from the intended intervention that arose because of the experimental context? | | | N |  |
|  | 2.4 If Y/PY to 2.3: Were these deviations likely to have affected the outcome? | | | NA |  |
|  | 2.5. If Y/PY/NI to 2.4: Were these deviations from intended intervention balanced between groups? | | | NA |  |
|  | 2.6 Was an appropriate analysis used to estimate the effect of assignment to intervention? | | | Y |  |
|  | 2.7 If N/PN/NI to 2.6: Was there potential for a substantial impact (on the result) of the failure to analyse participants in the group to which they were randomized? | | | NA |  |
|  | **Risk of bias judgement** | | | **Low** |  |
| **Bias due to missing outcome data** | 3.1 Were data for this outcome available for all, or nearly all, participants randomized? | | | N |  |
|  | 3.2 If N/PN/NI to 3.1: Is there evidence that result was not biased by missing outcome data? | | | Y |  |
|  | 3.3 If N/PN to 3.2: Could missingness in the outcome depend on its true value? | | | NA |  |
|  | 3.4 If Y/PY/NI to 3.3: Is it likely that missingness in the outcome depended on its true value? | | | NA |  |
|  | **Risk of bias judgement** | | | **Low** |  |
| **Bias in measurement of the outcome** | 4.1 Was the method of measuring the outcome inappropriate? | | | N |  |
|  | 4.2 Could measurement or ascertainment of the outcome have differed between intervention groups? | | | N |  |
|  | 4.3 Were outcome assessors aware of the intervention received by study participants? | | | Y |  |
|  | 4.4 If Y/PY/NI to 4.3: Could assessment of the outcome have been influenced by knowledge of intervention received? | | | N |  |
|  | 4.5 If Y/PY/NI to 4.4: Is it likely that assessment of the outcome was influenced by knowledge of intervention received? | | | NA |  |
|  | **Risk of bias judgement** | | | **Low** |  |
| **Bias in selection of the reported result** | 5.1 Were the data that produced this result analysed in accordance with a pre-specified analysis plan that was finalized before unblinded outcome data were available for analysis? | | | Y |  |
|  | 5.2 ... multiple eligible outcome measurements (e.g. scales, definitions, time points) within the outcome domain? | | | N |  |
|  | 5.3 ... multiple eligible analyses of the data? | | | N |  |
|  | **Risk of bias judgement** | | | **Low** |  |
| **Overall bias** | **Risk of bias judgement** | | | **Low** |  |
|  |  |  |  |  |  |
|  |  |  |  |  |  |
| **Unique ID** | Chan 2019 | **Study ID** |  | **Assessor** | I. S. Anyanwu |
| **Ref or Label** |  | **Aim** | assignment to intervention (the 'intention-to-treat' effect) |  |  |
| **Experimental** |  | **Comparator** |  | **Source** | Journal article(s) |
| **Outcome** |  | **Results** |  | **Weight** | 1 |
| **Domain** | **Signalling question** | | | **Response** | **Comments** |
| **Bias arising from the randomization process** | 1.1 Was the allocation sequence random? | | | Y |  |
|  | 1.2 Was the allocation sequence concealed until participants were enrolled and assigned to interventions? | | | Y |  |
|  | 1.3 Did baseline differences between intervention groups suggest a problem with the randomization process? | | | N |  |
|  | **Risk of bias judgement** | | | **Low** |  |
| **Bias due to deviations from intended interventions** | 2.1.Were participants aware of their assigned intervention during the trial? | | | Y |  |
|  | 2.2.Were carers and people delivering the interventions aware of participants' assigned intervention during the trial? | | | N |  |
|  | 2.3. If Y/PY/NI to 2.1 or 2.2: Were there deviations from the intended intervention that arose because of the experimental context? | | | N |  |
|  | 2.4 If Y/PY to 2.3: Were these deviations likely to have affected the outcome? | | | NA |  |
|  | 2.5. If Y/PY/NI to 2.4: Were these deviations from intended intervention balanced between groups? | | | NA |  |
|  | 2.6 Was an appropriate analysis used to estimate the effect of assignment to intervention? | | | Y |  |
|  | 2.7 If N/PN/NI to 2.6: Was there potential for a substantial impact (on the result) of the failure to analyse participants in the group to which they were randomized? | | | NA |  |
|  | **Risk of bias judgement** | | | **Low** |  |
| **Bias due to missing outcome data** | 3.1 Were data for this outcome available for all, or nearly all, participants randomized? | | | N |  |
|  | 3.2 If N/PN/NI to 3.1: Is there evidence that result was not biased by missing outcome data? | | | Y |  |
|  | 3.3 If N/PN to 3.2: Could missingness in the outcome depend on its true value? | | | NA |  |
|  | 3.4 If Y/PY/NI to 3.3: Is it likely that missingness in the outcome depended on its true value? | | | NA |  |
|  | **Risk of bias judgement** | | | **Low** |  |
| **Bias in measurement of the outcome** | 4.1 Was the method of measuring the outcome inappropriate? | | | N |  |
|  | 4.2 Could measurement or ascertainment of the outcome have differed between intervention groups? | | | N |  |
|  | 4.3 Were outcome assessors aware of the intervention received by study participants? | | | N |  |
|  | 4.4 If Y/PY/NI to 4.3: Could assessment of the outcome have been influenced by knowledge of intervention received? | | | NA |  |
|  | 4.5 If Y/PY/NI to 4.4: Is it likely that assessment of the outcome was influenced by knowledge of intervention received? | | | NA |  |
|  | **Risk of bias judgement** | | | **Low** |  |
| **Bias in selection of the reported result** | 5.1 Were the data that produced this result analysed in accordance with a pre-specified analysis plan that was finalized before unblinded outcome data were available for analysis? | | | Y |  |
|  | 5.2 ... multiple eligible outcome measurements (e.g. scales, definitions, time points) within the outcome domain? | | | N |  |
|  | 5.3 ... multiple eligible analyses of the data? | | | N |  |
|  | **Risk of bias judgement** | | | **Low** |  |
| **Overall bias** | **Risk of bias judgement** | | | **Low** |  |
|  |  |  |  |  |  |
|  |  |  |  |  |  |
| **Unique ID** | Danaher 2023 | **Study ID** |  | **Assessor** | I. S. Anyanwu |
| **Ref or Label** |  | **Aim** | assignment to intervention (the 'intention-to-treat' effect) |  |  |
| **Experimental** | MomMoodBooster2 + Perinatal Depression Program (TAU) | **Comparator** | TAU | **Source** | Journal article(s) |
| **Outcome** |  | **Results** |  | **Weight** | 1 |
| **Domain** | **Signalling question** | | | **Response** | **Comments** |
| **Bias arising from the randomization process** | 1.1 Was the allocation sequence random? | | | PY | Exact information on randomisation and allocation concealment were not provided, however, this was said to be done by a popular research management software - REDCap - hence, the assumption of "probably yes", for the two questions. |
|  | 1.2 Was the allocation sequence concealed until participants were enrolled and assigned to interventions? | | | PY |  |
|  | 1.3 Did baseline differences between intervention groups suggest a problem with the randomization process? | | | N |  |
|  | **Risk of bias judgement** | | | **Low** |  |
| **Bias due to deviations from intended interventions** | 2.1.Were participants aware of their assigned intervention during the trial? | | | PY | Although not stated explicitly, since the intervention is the addition of a web-app to routine care and all participants would have expectedingly signed an informed consent before participating, those using the web-app will likely know that they are in the interevention group. |
|  | 2.2.Were carers and people delivering the interventions aware of participants' assigned intervention during the trial? | | | NI |  |
|  | 2.3. If Y/PY/NI to 2.1 or 2.2: Were there deviations from the intended intervention that arose because of the experimental context? | | | N |  |
|  | 2.4 If Y/PY to 2.3: Were these deviations likely to have affected the outcome? | | | NA |  |
|  | 2.5. If Y/PY/NI to 2.4: Were these deviations from intended intervention balanced between groups? | | | NA |  |
|  | 2.6 Was an appropriate analysis used to estimate the effect of assignment to intervention? | | | Y |  |
|  | 2.7 If N/PN/NI to 2.6: Was there potential for a substantial impact (on the result) of the failure to analyse participants in the group to which they were randomized? | | | NA |  |
|  | **Risk of bias judgement** | | | **Low** |  |
| **Bias due to missing outcome data** | 3.1 Were data for this outcome available for all, or nearly all, participants randomized? | | | Y | <20% attrition rate per group |
|  | 3.2 If N/PN/NI to 3.1: Is there evidence that result was not biased by missing outcome data? | | | NA |  |
|  | 3.3 If N/PN to 3.2: Could missingness in the outcome depend on its true value? | | | NA |  |
|  | 3.4 If Y/PY/NI to 3.3: Is it likely that missingness in the outcome depended on its true value? | | | NA |  |
|  | **Risk of bias judgement** | | | **Low** |  |
| **Bias in measurement of the outcome** | 4.1 Was the method of measuring the outcome inappropriate? | | | N |  |
|  | 4.2 Could measurement or ascertainment of the outcome have differed between intervention groups? | | | N |  |
|  | 4.3 Were outcome assessors aware of the intervention received by study participants? | | | PY | The participants were the outcome assessors themselves and as previously noted, they probably knew their intervention groups, hence the answer "probably yes". |
|  | 4.4 If Y/PY/NI to 4.3: Could assessment of the outcome have been influenced by knowledge of intervention received? | | | PY |  |
|  | 4.5 If Y/PY/NI to 4.4: Is it likely that assessment of the outcome was influenced by knowledge of intervention received? | | | N |  |
|  | **Risk of bias judgement** | | | **Some concerns** |  |
| **Bias in selection of the reported result** | 5.1 Were the data that produced this result analysed in accordance with a pre-specified analysis plan that was finalized before unblinded outcome data were available for analysis? | | | Y |  |
|  | 5.2 ... multiple eligible outcome measurements (e.g. scales, definitions, time points) within the outcome domain? | | | N |  |
|  | 5.3 ... multiple eligible analyses of the data? | | | N |  |
|  | **Risk of bias judgement** | | | **Low** |  |
| **Overall bias** | **Risk of bias judgement** | | | **Some concerns** |  |
|  |  |  |  |  |  |
|  |  |  |  |  |  |
| **Unique ID** | Dennis 2020 | **Study ID** |  | **Assessor** | I. S. Anyanwu |
| **Ref or Label** |  | **Aim** | assignment to intervention (the 'intention-to-treat' effect) |  |  |
| **Experimental** | Telephone-IPT | **Comparator** | TAU | **Source** | Journal article(s); Trial protocol; Non-commercial trial registry record (e.g. ClinicalTrials.gov record) |
| **Outcome** |  | **Results** |  | **Weight** | 1 |
| **Domain** | **Signalling question** | | | **Response** | **Comments** |
| **Bias arising from the randomization process** | 1.1 Was the allocation sequence random? | | | Y | Web-based randomisation (www.randomize.net) and stratification based on province was used |
|  | 1.2 Was the allocation sequence concealed until participants were enrolled and assigned to interventions? | | | Y |  |
|  | 1.3 Did baseline differences between intervention groups suggest a problem with the randomization process? | | | N |  |
|  | **Risk of bias judgement** | | | **Low** |  |
| **Bias due to deviations from intended interventions** | 2.1.Were participants aware of their assigned intervention during the trial? | | | Y |  |
|  | 2.2.Were carers and people delivering the interventions aware of participants' assigned intervention during the trial? | | | Y |  |
|  | 2.3. If Y/PY/NI to 2.1 or 2.2: Were there deviations from the intended intervention that arose because of the experimental context? | | | N |  |
|  | 2.4 If Y/PY to 2.3: Were these deviations likely to have affected the outcome? | | | NA |  |
|  | 2.5. If Y/PY/NI to 2.4: Were these deviations from intended intervention balanced between groups? | | | NA |  |
|  | 2.6 Was an appropriate analysis used to estimate the effect of assignment to intervention? | | | Y |  |
|  | 2.7 If N/PN/NI to 2.6: Was there potential for a substantial impact (on the result) of the failure to analyse participants in the group to which they were randomized? | | | NA |  |
|  | **Risk of bias judgement** | | | **Low** |  |
| **Bias due to missing outcome data** | 3.1 Were data for this outcome available for all, or nearly all, participants randomized? | | | Y |  |
|  | 3.2 If N/PN/NI to 3.1: Is there evidence that result was not biased by missing outcome data? | | | NA |  |
|  | 3.3 If N/PN to 3.2: Could missingness in the outcome depend on its true value? | | | NA |  |
|  | 3.4 If Y/PY/NI to 3.3: Is it likely that missingness in the outcome depended on its true value? | | | NA |  |
|  | **Risk of bias judgement** | | | **Low** |  |
| **Bias in measurement of the outcome** | 4.1 Was the method of measuring the outcome inappropriate? | | | N |  |
|  | 4.2 Could measurement or ascertainment of the outcome have differed between intervention groups? | | | N |  |
|  | 4.3 Were outcome assessors aware of the intervention received by study participants? | | | N |  |
|  | 4.4 If Y/PY/NI to 4.3: Could assessment of the outcome have been influenced by knowledge of intervention received? | | | NA |  |
|  | 4.5 If Y/PY/NI to 4.4: Is it likely that assessment of the outcome was influenced by knowledge of intervention received? | | | NA |  |
|  | **Risk of bias judgement** | | | **Low** |  |
| **Bias in selection of the reported result** | 5.1 Were the data that produced this result analysed in accordance with a pre-specified analysis plan that was finalized before unblinded outcome data were available for analysis? | | | Y |  |
|  | 5.2 ... multiple eligible outcome measurements (e.g. scales, definitions, time points) within the outcome domain? | | | N |  |
|  | 5.3 ... multiple eligible analyses of the data? | | | N |  |
|  | **Risk of bias judgement** | | | **Low** |  |
| **Overall bias** | **Risk of bias judgement** | | | **Low** |  |
|  |  |  |  |  |  |
|  |  |  |  |  |  |
| **Unique ID** | Gammer 2020 | **Study ID** |  | **Assessor** | I. S. Anyanwu |
| **Ref or Label** |  | **Aim** | assignment to intervention (the 'intention-to-treat' effect) |  |  |
| **Experimental** | Web-based mindfulness and self-compassion tool | **Comparator** | TAU | **Source** | Journal article(s); Non-commercial trial registry record (e.g. ClinicalTrials.gov record) |
| **Outcome** |  | **Results** |  | **Weight** | 1 |
| **Domain** | **Signalling question** | | | **Response** | **Comments** |
| **Bias arising from the randomization process** | 1.1 Was the allocation sequence random? | | | Y | Randomization was automated and conducted by a computerized random number generator built into the delivery website and programmed to perform block randomization (in blocks of six) to ensure equal group sizes.  The researchers did not know in advance which condition a participant would be allocated to. |
|  | 1.2 Was the allocation sequence concealed until participants were enrolled and assigned to interventions? | | | Y |  |
|  | 1.3 Did baseline differences between intervention groups suggest a problem with the randomization process? | | | N |  |
|  | **Risk of bias judgement** | | | **Low** |  |
| **Bias due to deviations from intended interventions** | 2.1.Were participants aware of their assigned intervention during the trial? | | | PY | The intervention was self-guided and by the nature of the study, since ethical consent will be given following informed consent, the participants in the experiment group would probably know they were in that group. |
|  | 2.2.Were carers and people delivering the interventions aware of participants' assigned intervention during the trial? | | | PY |  |
|  | 2.3. If Y/PY/NI to 2.1 or 2.2: Were there deviations from the intended intervention that arose because of the experimental context? | | | N |  |
|  | 2.4 If Y/PY to 2.3: Were these deviations likely to have affected the outcome? | | | NA |  |
|  | 2.5. If Y/PY/NI to 2.4: Were these deviations from intended intervention balanced between groups? | | | NA |  |
|  | 2.6 Was an appropriate analysis used to estimate the effect of assignment to intervention? | | | Y |  |
|  | 2.7 If N/PN/NI to 2.6: Was there potential for a substantial impact (on the result) of the failure to analyse participants in the group to which they were randomized? | | | NA |  |
|  | **Risk of bias judgement** | | | **Low** |  |
| **Bias due to missing outcome data** | 3.1 Were data for this outcome available for all, or nearly all, participants randomized? | | | N | There was >20% attrition rate at post-intervention and 12 weeks follow-up |
|  | 3.2 If N/PN/NI to 3.1: Is there evidence that result was not biased by missing outcome data? | | | Y | No significant difference was noted in the characteristics of those missing and completers. Also ITT analyses were used to assess the outcome data. |
|  | 3.3 If N/PN to 3.2: Could missingness in the outcome depend on its true value? | | | NA |  |
|  | 3.4 If Y/PY/NI to 3.3: Is it likely that missingness in the outcome depended on its true value? | | | NA |  |
|  | **Risk of bias judgement** | | | **Low** |  |
| **Bias in measurement of the outcome** | 4.1 Was the method of measuring the outcome inappropriate? | | | N |  |
|  | 4.2 Could measurement or ascertainment of the outcome have differed between intervention groups? | | | N |  |
|  | 4.3 Were outcome assessors aware of the intervention received by study participants? | | | PY | The participants were the outcome assesors themselves and as previously stated, they probably knew their intervention groups in the study. |
|  | 4.4 If Y/PY/NI to 4.3: Could assessment of the outcome have been influenced by knowledge of intervention received? | | | PY |  |
|  | 4.5 If Y/PY/NI to 4.4: Is it likely that assessment of the outcome was influenced by knowledge of intervention received? | | | N |  |
|  | **Risk of bias judgement** | | | **Some concerns** |  |
| **Bias in selection of the reported result** | 5.1 Were the data that produced this result analysed in accordance with a pre-specified analysis plan that was finalized before unblinded outcome data were available for analysis? | | | Y |  |
|  | 5.2 ... multiple eligible outcome measurements (e.g. scales, definitions, time points) within the outcome domain? | | | N |  |
|  | 5.3 ... multiple eligible analyses of the data? | | | N |  |
|  | **Risk of bias judgement** | | | **Low** |  |
| **Overall bias** | **Risk of bias judgement** | | | **Some concerns** |  |
|  |  |  |  |  |  |
|  |  |  |  |  |  |
| **Unique ID** | Haga 2019 | **Study ID** |  | **Assessor** | I. S. Anyanwu |
| **Ref or Label** |  | **Aim** | assignment to intervention (the 'intention-to-treat' effect) |  |  |
| **Experimental** | Online mindfulness | **Comparator** | TAU | **Source** | Journal article(s); Non-commercial trial registry record (e.g. ClinicalTrials.gov record) |
| **Outcome** |  | **Results** |  | **Weight** | 1 |
| **Domain** | **Signalling question** | | | **Response** | **Comments** |
| **Bias arising from the randomization process** | 1.1 Was the allocation sequence random? | | | Y | Automated, unrestricted randomization procedure was used |
|  | 1.2 Was the allocation sequence concealed until participants were enrolled and assigned to interventions? | | | Y |  |
|  | 1.3 Did baseline differences between intervention groups suggest a problem with the randomization process? | | | N |  |
|  | **Risk of bias judgement** | | | **Low** |  |
| **Bias due to deviations from intended interventions** | 2.1.Were participants aware of their assigned intervention during the trial? | | | PY | Although not explicitly stated, the nature of the intervention and the compulsory expectation of acquiring informed consent for ethical reasons, there is a high probability that the participants knew they were in the experimental group. |
|  | 2.2.Were carers and people delivering the interventions aware of participants' assigned intervention during the trial? | | | PY |  |
|  | 2.3. If Y/PY/NI to 2.1 or 2.2: Were there deviations from the intended intervention that arose because of the experimental context? | | | N |  |
|  | 2.4 If Y/PY to 2.3: Were these deviations likely to have affected the outcome? | | | NA |  |
|  | 2.5. If Y/PY/NI to 2.4: Were these deviations from intended intervention balanced between groups? | | | NA |  |
|  | 2.6 Was an appropriate analysis used to estimate the effect of assignment to intervention? | | | Y |  |
|  | 2.7 If N/PN/NI to 2.6: Was there potential for a substantial impact (on the result) of the failure to analyse participants in the group to which they were randomized? | | | NA |  |
|  | **Risk of bias judgement** | | | **Low** |  |
| **Bias due to missing outcome data** | 3.1 Were data for this outcome available for all, or nearly all, participants randomized? | | | N |  |
|  | 3.2 If N/PN/NI to 3.1: Is there evidence that result was not biased by missing outcome data? | | | PY |  |
|  | 3.3 If N/PN to 3.2: Could missingness in the outcome depend on its true value? | | | NA |  |
|  | 3.4 If Y/PY/NI to 3.3: Is it likely that missingness in the outcome depended on its true value? | | | NA |  |
|  | **Risk of bias judgement** | | | **Low** |  |
| **Bias in measurement of the outcome** | 4.1 Was the method of measuring the outcome inappropriate? | | | N |  |
|  | 4.2 Could measurement or ascertainment of the outcome have differed between intervention groups? | | | N |  |
|  | 4.3 Were outcome assessors aware of the intervention received by study participants? | | | NI |  |
|  | 4.4 If Y/PY/NI to 4.3: Could assessment of the outcome have been influenced by knowledge of intervention received? | | | PN |  |
|  | 4.5 If Y/PY/NI to 4.4: Is it likely that assessment of the outcome was influenced by knowledge of intervention received? | | | NA |  |
|  | **Risk of bias judgement** | | | **Low** |  |
| **Bias in selection of the reported result** | 5.1 Were the data that produced this result analysed in accordance with a pre-specified analysis plan that was finalized before unblinded outcome data were available for analysis? | | | Y |  |
|  | 5.2 ... multiple eligible outcome measurements (e.g. scales, definitions, time points) within the outcome domain? | | | N |  |
|  | 5.3 ... multiple eligible analyses of the data? | | | N |  |
|  | **Risk of bias judgement** | | | **Low** |  |
| **Overall bias** | **Risk of bias judgement** | | | **Low** |  |
|  |  |  |  |  |  |
|  |  |  |  |  |  |
| **Unique ID** | Hassdenteufel 2023 | **Study ID** |  | **Assessor** | I. S. Anyanwu |
| **Ref or Label** |  | **Aim** | assignment to intervention (the 'intention-to-treat' effect) |  |  |
| **Experimental** | eMBI | **Comparator** | TAU | **Source** | Journal article(s); Trial protocol; Non-commercial trial registry record (e.g. ClinicalTrials.gov record) |
| **Outcome** |  | **Results** |  | **Weight** | 1 |
| **Domain** | **Signalling question** | | | **Response** | **Comments** |
| **Bias arising from the randomization process** | 1.1 Was the allocation sequence random? | | | Y |  |
|  | 1.2 Was the allocation sequence concealed until participants were enrolled and assigned to interventions? | | | Y |  |
|  | 1.3 Did baseline differences between intervention groups suggest a problem with the randomization process? | | | N |  |
|  | **Risk of bias judgement** | | | **Low** |  |
| **Bias due to deviations from intended interventions** | 2.1.Were participants aware of their assigned intervention during the trial? | | | PY |  |
|  | 2.2.Were carers and people delivering the interventions aware of participants' assigned intervention during the trial? | | | Y |  |
|  | 2.3. If Y/PY/NI to 2.1 or 2.2: Were there deviations from the intended intervention that arose because of the experimental context? | | | N |  |
|  | 2.4 If Y/PY to 2.3: Were these deviations likely to have affected the outcome? | | | NA |  |
|  | 2.5. If Y/PY/NI to 2.4: Were these deviations from intended intervention balanced between groups? | | | NA |  |
|  | 2.6 Was an appropriate analysis used to estimate the effect of assignment to intervention? | | | Y |  |
|  | 2.7 If N/PN/NI to 2.6: Was there potential for a substantial impact (on the result) of the failure to analyse participants in the group to which they were randomized? | | | NA |  |
|  | **Risk of bias judgement** | | | **Low** |  |
| **Bias due to missing outcome data** | 3.1 Were data for this outcome available for all, or nearly all, participants randomized? | | | N | >20% attrition rate |
|  | 3.2 If N/PN/NI to 3.1: Is there evidence that result was not biased by missing outcome data? | | | Y |  |
|  | 3.3 If N/PN to 3.2: Could missingness in the outcome depend on its true value? | | | NA |  |
|  | 3.4 If Y/PY/NI to 3.3: Is it likely that missingness in the outcome depended on its true value? | | | NA |  |
|  | **Risk of bias judgement** | | | **Low** |  |
| **Bias in measurement of the outcome** | 4.1 Was the method of measuring the outcome inappropriate? | | | N |  |
|  | 4.2 Could measurement or ascertainment of the outcome have differed between intervention groups? | | | N |  |
|  | 4.3 Were outcome assessors aware of the intervention received by study participants? | | | PY | The participants self-reported the outcomes and they probably knew their intervention groups. |
|  | 4.4 If Y/PY/NI to 4.3: Could assessment of the outcome have been influenced by knowledge of intervention received? | | | PY |  |
|  | 4.5 If Y/PY/NI to 4.4: Is it likely that assessment of the outcome was influenced by knowledge of intervention received? | | | N |  |
|  | **Risk of bias judgement** | | | **Some concerns** |  |
| **Bias in selection of the reported result** | 5.1 Were the data that produced this result analysed in accordance with a pre-specified analysis plan that was finalized before unblinded outcome data were available for analysis? | | | Y |  |
|  | 5.2 ... multiple eligible outcome measurements (e.g. scales, definitions, time points) within the outcome domain? | | | N |  |
|  | 5.3 ... multiple eligible analyses of the data? | | | N |  |
|  | **Risk of bias judgement** | | | **Low** |  |
| **Overall bias** | **Risk of bias judgement** | | | **Some concerns** |  |
|  |  |  |  |  |  |
|  |  |  |  |  |  |
| **Unique ID** | Heller 2020 | **Study ID** |  | **Assessor** | I. S. Anyanwu |
| **Ref or Label** |  | **Aim** | assignment to intervention (the 'intention-to-treat' effect) |  |  |
| **Experimental** | Online (web-based) PST | **Comparator** | TAU | **Source** | Journal article(s); Trial protocol |
| **Outcome** |  | **Results** |  | **Weight** | 1 |
| **Domain** | **Signalling question** | | | **Response** | **Comments** |
| **Bias arising from the randomization process** | 1.1 Was the allocation sequence random? | | | Y |  |
|  | 1.2 Was the allocation sequence concealed until participants were enrolled and assigned to interventions? | | | Y |  |
|  | 1.3 Did baseline differences between intervention groups suggest a problem with the randomization process? | | | N |  |
|  | **Risk of bias judgement** | | | **Low** |  |
| **Bias due to deviations from intended interventions** | 2.1.Were participants aware of their assigned intervention during the trial? | | | PY | 1. As an online intervention, participants probably knew if they were in the experiment group or not. 2. The interventon contained a thearpist-assited component delivered by trained coaches and this was not available for the TAU group, so the people delivering this part of the intervention knew the participants' groups. |
|  | 2.2.Were carers and people delivering the interventions aware of participants' assigned intervention during the trial? | | | Y |  |
|  | 2.3. If Y/PY/NI to 2.1 or 2.2: Were there deviations from the intended intervention that arose because of the experimental context? | | | PY | The protocol originally planned for the course to be completed in 6 weeks but the final published work implies a minimum completion time of 5 weeks. |
|  | 2.4 If Y/PY to 2.3: Were these deviations likely to have affected the outcome? | | | N |  |
|  | 2.5. If Y/PY/NI to 2.4: Were these deviations from intended intervention balanced between groups? | | | NA |  |
|  | 2.6 Was an appropriate analysis used to estimate the effect of assignment to intervention? | | | Y |  |
|  | 2.7 If N/PN/NI to 2.6: Was there potential for a substantial impact (on the result) of the failure to analyse participants in the group to which they were randomized? | | | NA |  |
|  | **Risk of bias judgement** | | | **Some concerns** |  |
| **Bias due to missing outcome data** | 3.1 Were data for this outcome available for all, or nearly all, participants randomized? | | | N | >20% attrition rate |
|  | 3.2 If N/PN/NI to 3.1: Is there evidence that result was not biased by missing outcome data? | | | Y |  |
|  | 3.3 If N/PN to 3.2: Could missingness in the outcome depend on its true value? | | | NA |  |
|  | 3.4 If Y/PY/NI to 3.3: Is it likely that missingness in the outcome depended on its true value? | | | NA |  |
|  | **Risk of bias judgement** | | | **Low** |  |
| **Bias in measurement of the outcome** | 4.1 Was the method of measuring the outcome inappropriate? | | | N |  |
|  | 4.2 Could measurement or ascertainment of the outcome have differed between intervention groups? | | | N |  |
|  | 4.3 Were outcome assessors aware of the intervention received by study participants? | | | PY | The participants were the assesors themselves and probably knew that they were in the experiment group or not. |
|  | 4.4 If Y/PY/NI to 4.3: Could assessment of the outcome have been influenced by knowledge of intervention received? | | | PY |  |
|  | 4.5 If Y/PY/NI to 4.4: Is it likely that assessment of the outcome was influenced by knowledge of intervention received? | | | N |  |
|  | **Risk of bias judgement** | | | **Some concerns** |  |
| **Bias in selection of the reported result** | 5.1 Were the data that produced this result analysed in accordance with a pre-specified analysis plan that was finalized before unblinded outcome data were available for analysis? | | | Y |  |
|  | 5.2 ... multiple eligible outcome measurements (e.g. scales, definitions, time points) within the outcome domain? | | | N |  |
|  | 5.3 ... multiple eligible analyses of the data? | | | N |  |
|  | **Risk of bias judgement** | | | **Low** |  |
| **Overall bias** | **Risk of bias judgement** | | | **Some concerns** |  |
|  |  |  |  |  |  |
|  |  |  |  |  |  |
| **Unique ID** | Huang 2021 | **Study ID** |  | **Assessor** | I. S. Anyanwu |
| **Ref or Label** |  | **Aim** | assignment to intervention (the 'intention-to-treat' effect) |  |  |
| **Experimental** | Internet based support program | **Comparator** | TAU | **Source** | Journal article(s) |
| **Outcome** |  | **Results** |  | **Weight** | 1 |
| **Domain** | **Signalling question** | | | **Response** | **Comments** |
| **Bias arising from the randomization process** | 1.1 Was the allocation sequence random? | | | Y |  |
|  | 1.2 Was the allocation sequence concealed until participants were enrolled and assigned to interventions? | | | Y |  |
|  | 1.3 Did baseline differences between intervention groups suggest a problem with the randomization process? | | | N |  |
|  | **Risk of bias judgement** | | | **Low** |  |
| **Bias due to deviations from intended interventions** | 2.1.Were participants aware of their assigned intervention during the trial? | | | N |  |
|  | 2.2.Were carers and people delivering the interventions aware of participants' assigned intervention during the trial? | | | Y |  |
|  | 2.3. If Y/PY/NI to 2.1 or 2.2: Were there deviations from the intended intervention that arose because of the experimental context? | | | N |  |
|  | 2.4 If Y/PY to 2.3: Were these deviations likely to have affected the outcome? | | | NA |  |
|  | 2.5. If Y/PY/NI to 2.4: Were these deviations from intended intervention balanced between groups? | | | NA |  |
|  | 2.6 Was an appropriate analysis used to estimate the effect of assignment to intervention? | | | Y |  |
|  | 2.7 If N/PN/NI to 2.6: Was there potential for a substantial impact (on the result) of the failure to analyse participants in the group to which they were randomized? | | | NA |  |
|  | **Risk of bias judgement** | | | **Low** |  |
| **Bias due to missing outcome data** | 3.1 Were data for this outcome available for all, or nearly all, participants randomized? | | | Y |  |
|  | 3.2 If N/PN/NI to 3.1: Is there evidence that result was not biased by missing outcome data? | | | NA |  |
|  | 3.3 If N/PN to 3.2: Could missingness in the outcome depend on its true value? | | | NA |  |
|  | 3.4 If Y/PY/NI to 3.3: Is it likely that missingness in the outcome depended on its true value? | | | NA |  |
|  | **Risk of bias judgement** | | | **Low** |  |
| **Bias in measurement of the outcome** | 4.1 Was the method of measuring the outcome inappropriate? | | | N |  |
|  | 4.2 Could measurement or ascertainment of the outcome have differed between intervention groups? | | | N |  |
|  | 4.3 Were outcome assessors aware of the intervention received by study participants? | | | N |  |
|  | 4.4 If Y/PY/NI to 4.3: Could assessment of the outcome have been influenced by knowledge of intervention received? | | | NA |  |
|  | 4.5 If Y/PY/NI to 4.4: Is it likely that assessment of the outcome was influenced by knowledge of intervention received? | | | NA |  |
|  | **Risk of bias judgement** | | | **Low** |  |
| **Bias in selection of the reported result** | 5.1 Were the data that produced this result analysed in accordance with a pre-specified analysis plan that was finalized before unblinded outcome data were available for analysis? | | | Y |  |
|  | 5.2 ... multiple eligible outcome measurements (e.g. scales, definitions, time points) within the outcome domain? | | | N |  |
|  | 5.3 ... multiple eligible analyses of the data? | | | N |  |
|  | **Risk of bias judgement** | | | **Low** |  |
| **Overall bias** | **Risk of bias judgement** | | | **Low** |  |
|  |  |  |  |  |  |
|  |  |  |  |  |  |
| **Unique ID** | Huh 2023 | **Study ID** |  | **Assessor** | I. S. Anyanwu |
| **Ref or Label** |  | **Aim** | assignment to intervention (the 'intention-to-treat' effect) |  |  |
| **Experimental** | Online group CBT | **Comparator** | TAU | **Source** | Journal article(s); Non-commercial trial registry record (e.g. ClinicalTrials.gov record) |
| **Outcome** |  | **Results** |  | **Weight** | 1 |
| **Domain** | **Signalling question** | | | **Response** | **Comments** |
| **Bias arising from the randomization process** | 1.1 Was the allocation sequence random? | | | Y |  |
|  | 1.2 Was the allocation sequence concealed until participants were enrolled and assigned to interventions? | | | Y |  |
|  | 1.3 Did baseline differences between intervention groups suggest a problem with the randomization process? | | | N |  |
|  | **Risk of bias judgement** | | | **Low** |  |
| **Bias due to deviations from intended interventions** | 2.1.Were participants aware of their assigned intervention during the trial? | | | Y |  |
|  | 2.2.Were carers and people delivering the interventions aware of participants' assigned intervention during the trial? | | | Y |  |
|  | 2.3. If Y/PY/NI to 2.1 or 2.2: Were there deviations from the intended intervention that arose because of the experimental context? | | | N |  |
|  | 2.4 If Y/PY to 2.3: Were these deviations likely to have affected the outcome? | | | NA |  |
|  | 2.5. If Y/PY/NI to 2.4: Were these deviations from intended intervention balanced between groups? | | | NA |  |
|  | 2.6 Was an appropriate analysis used to estimate the effect of assignment to intervention? | | | Y |  |
|  | 2.7 If N/PN/NI to 2.6: Was there potential for a substantial impact (on the result) of the failure to analyse participants in the group to which they were randomized? | | | NA |  |
|  | **Risk of bias judgement** | | | **Low** |  |
| **Bias due to missing outcome data** | 3.1 Were data for this outcome available for all, or nearly all, participants randomized? | | | N | >20% attrition rate |
|  | 3.2 If N/PN/NI to 3.1: Is there evidence that result was not biased by missing outcome data? | | | N | THere were some significant differences between those lost to attrition and those who completed the intervention at the two times of follow-up. |
|  | 3.3 If N/PN to 3.2: Could missingness in the outcome depend on its true value? | | | N |  |
|  | 3.4 If Y/PY/NI to 3.3: Is it likely that missingness in the outcome depended on its true value? | | | NA |  |
|  | **Risk of bias judgement** | | | **Low** |  |
| **Bias in measurement of the outcome** | 4.1 Was the method of measuring the outcome inappropriate? | | | N |  |
|  | 4.2 Could measurement or ascertainment of the outcome have differed between intervention groups? | | | N |  |
|  | 4.3 Were outcome assessors aware of the intervention received by study participants? | | | N | Data was collected by research assistants who were blinded to the participants groups. |
|  | 4.4 If Y/PY/NI to 4.3: Could assessment of the outcome have been influenced by knowledge of intervention received? | | | NA |  |
|  | 4.5 If Y/PY/NI to 4.4: Is it likely that assessment of the outcome was influenced by knowledge of intervention received? | | | NA |  |
|  | **Risk of bias judgement** | | | **Low** |  |
| **Bias in selection of the reported result** | 5.1 Were the data that produced this result analysed in accordance with a pre-specified analysis plan that was finalized before unblinded outcome data were available for analysis? | | | Y |  |
|  | 5.2 ... multiple eligible outcome measurements (e.g. scales, definitions, time points) within the outcome domain? | | | N |  |
|  | 5.3 ... multiple eligible analyses of the data? | | | N |  |
|  | **Risk of bias judgement** | | | **Low** |  |
| **Overall bias** | **Risk of bias judgement** | | | **Low** |  |
|  |  |  |  |  |  |
|  |  |  |  |  |  |
| **Unique ID** | Hulsbosch 2023 | **Study ID** |  | **Assessor** | I. S. Anyanwu |
| **Ref or Label** |  | **Aim** | assignment to intervention (the 'intention-to-treat' effect) |  |  |
| **Experimental** | Online MBI | **Comparator** | TAU | **Source** | Journal article(s); Trial protocol |
| **Outcome** |  | **Results** |  | **Weight** | 1 |
| **Domain** | **Signalling question** | | | **Response** | **Comments** |
| **Bias arising from the randomization process** | 1.1 Was the allocation sequence random? | | | Y |  |
|  | 1.2 Was the allocation sequence concealed until participants were enrolled and assigned to interventions? | | | Y |  |
|  | 1.3 Did baseline differences between intervention groups suggest a problem with the randomization process? | | | N |  |
|  | **Risk of bias judgement** | | | **Low** |  |
| **Bias due to deviations from intended interventions** | 2.1.Were participants aware of their assigned intervention during the trial? | | | Y |  |
|  | 2.2.Were carers and people delivering the interventions aware of participants' assigned intervention during the trial? | | | Y |  |
|  | 2.3. If Y/PY/NI to 2.1 or 2.2: Were there deviations from the intended intervention that arose because of the experimental context? | | | N |  |
|  | 2.4 If Y/PY to 2.3: Were these deviations likely to have affected the outcome? | | | NA |  |
|  | 2.5. If Y/PY/NI to 2.4: Were these deviations from intended intervention balanced between groups? | | | NA |  |
|  | 2.6 Was an appropriate analysis used to estimate the effect of assignment to intervention? | | | Y |  |
|  | 2.7 If N/PN/NI to 2.6: Was there potential for a substantial impact (on the result) of the failure to analyse participants in the group to which they were randomized? | | | NA |  |
|  | **Risk of bias judgement** | | | **Low** |  |
| **Bias due to missing outcome data** | 3.1 Were data for this outcome available for all, or nearly all, participants randomized? | | | Y |  |
|  | 3.2 If N/PN/NI to 3.1: Is there evidence that result was not biased by missing outcome data? | | | NA |  |
|  | 3.3 If N/PN to 3.2: Could missingness in the outcome depend on its true value? | | | NA |  |
|  | 3.4 If Y/PY/NI to 3.3: Is it likely that missingness in the outcome depended on its true value? | | | NA |  |
|  | **Risk of bias judgement** | | | **Low** |  |
| **Bias in measurement of the outcome** | 4.1 Was the method of measuring the outcome inappropriate? | | | N |  |
|  | 4.2 Could measurement or ascertainment of the outcome have differed between intervention groups? | | | N |  |
|  | 4.3 Were outcome assessors aware of the intervention received by study participants? | | | Y | Participants were the assessors and they knew their intervention groups. |
|  | 4.4 If Y/PY/NI to 4.3: Could assessment of the outcome have been influenced by knowledge of intervention received? | | | PY | Since the participants knew if they were in the experiment group, they may have responded in a way that they feel that the intervention was helpful whether it was so or not. |
|  | 4.5 If Y/PY/NI to 4.4: Is it likely that assessment of the outcome was influenced by knowledge of intervention received? | | | N |  |
|  | **Risk of bias judgement** | | | **Some concerns** |  |
| **Bias in selection of the reported result** | 5.1 Were the data that produced this result analysed in accordance with a pre-specified analysis plan that was finalized before unblinded outcome data were available for analysis? | | | Y |  |
|  | 5.2 ... multiple eligible outcome measurements (e.g. scales, definitions, time points) within the outcome domain? | | | N |  |
|  | 5.3 ... multiple eligible analyses of the data? | | | N |  |
|  | **Risk of bias judgement** | | | **Low** |  |
| **Overall bias** | **Risk of bias judgement** | | | **Some concerns** |  |
|  |  |  |  |  |  |
|  |  |  |  |  |  |
| **Unique ID** | Jannati 2020 | **Study ID** |  | **Assessor** | I. S. Anyanwu |
| **Ref or Label** |  | **Aim** | assignment to intervention (the 'intention-to-treat' effect) |  |  |
| **Experimental** | Mobile app CBT | **Comparator** | TAU | **Source** | Journal article(s) |
| **Outcome** |  | **Results** |  | **Weight** | 1 |
| **Domain** | **Signalling question** | | | **Response** | **Comments** |
| **Bias arising from the randomization process** | 1.1 Was the allocation sequence random? | | | Y |  |
|  | 1.2 Was the allocation sequence concealed until participants were enrolled and assigned to interventions? | | | Y |  |
|  | 1.3 Did baseline differences between intervention groups suggest a problem with the randomization process? | | | PN |  |
|  | **Risk of bias judgement** | | | **Low** |  |
| **Bias due to deviations from intended interventions** | 2.1.Were participants aware of their assigned intervention during the trial? | | | Y |  |
|  | 2.2.Were carers and people delivering the interventions aware of participants' assigned intervention during the trial? | | | Y |  |
|  | 2.3. If Y/PY/NI to 2.1 or 2.2: Were there deviations from the intended intervention that arose because of the experimental context? | | | N |  |
|  | 2.4 If Y/PY to 2.3: Were these deviations likely to have affected the outcome? | | | NA |  |
|  | 2.5. If Y/PY/NI to 2.4: Were these deviations from intended intervention balanced between groups? | | | NA |  |
|  | 2.6 Was an appropriate analysis used to estimate the effect of assignment to intervention? | | | Y |  |
|  | 2.7 If N/PN/NI to 2.6: Was there potential for a substantial impact (on the result) of the failure to analyse participants in the group to which they were randomized? | | | NA |  |
|  | **Risk of bias judgement** | | | **Low** |  |
| **Bias due to missing outcome data** | 3.1 Were data for this outcome available for all, or nearly all, participants randomized? | | | Y |  |
|  | 3.2 If N/PN/NI to 3.1: Is there evidence that result was not biased by missing outcome data? | | | NA |  |
|  | 3.3 If N/PN to 3.2: Could missingness in the outcome depend on its true value? | | | NA |  |
|  | 3.4 If Y/PY/NI to 3.3: Is it likely that missingness in the outcome depended on its true value? | | | NA |  |
|  | **Risk of bias judgement** | | | **Low** |  |
| **Bias in measurement of the outcome** | 4.1 Was the method of measuring the outcome inappropriate? | | | N |  |
|  | 4.2 Could measurement or ascertainment of the outcome have differed between intervention groups? | | | N |  |
|  | 4.3 Were outcome assessors aware of the intervention received by study participants? | | | Y |  |
|  | 4.4 If Y/PY/NI to 4.3: Could assessment of the outcome have been influenced by knowledge of intervention received? | | | Y |  |
|  | 4.5 If Y/PY/NI to 4.4: Is it likely that assessment of the outcome was influenced by knowledge of intervention received? | | | N |  |
|  | **Risk of bias judgement** | | | **Some concerns** |  |
| **Bias in selection of the reported result** | 5.1 Were the data that produced this result analysed in accordance with a pre-specified analysis plan that was finalized before unblinded outcome data were available for analysis? | | | Y |  |
|  | 5.2 ... multiple eligible outcome measurements (e.g. scales, definitions, time points) within the outcome domain? | | | N |  |
|  | 5.3 ... multiple eligible analyses of the data? | | | N |  |
|  | **Risk of bias judgement** | | | **Low** |  |
| **Overall bias** | **Risk of bias judgement** | | | **Some concerns** |  |
|  |  |  |  |  |  |
|  |  |  |  |  |  |
| **Unique ID** | Jiao 2019 | **Study ID** |  | **Assessor** | I. S. Anyanwu |
| **Ref or Label** |  | **Aim** | assignment to intervention (the 'intention-to-treat' effect) |  |  |
| **Experimental** | Web-based psychoeducation | **Comparator** | TAU | **Source** | Journal article(s); Trial protocol |
| **Outcome** |  | **Results** |  | **Weight** | 1 |
| **Domain** | **Signalling question** | | | **Response** | **Comments** |
| **Bias arising from the randomization process** | 1.1 Was the allocation sequence random? | | | Y |  |
|  | 1.2 Was the allocation sequence concealed until participants were enrolled and assigned to interventions? | | | Y |  |
|  | 1.3 Did baseline differences between intervention groups suggest a problem with the randomization process? | | | N |  |
|  | **Risk of bias judgement** | | | **Low** |  |
| **Bias due to deviations from intended interventions** | 2.1.Were participants aware of their assigned intervention during the trial? | | | Y |  |
|  | 2.2.Were carers and people delivering the interventions aware of participants' assigned intervention during the trial? | | | Y |  |
|  | 2.3. If Y/PY/NI to 2.1 or 2.2: Were there deviations from the intended intervention that arose because of the experimental context? | | | N |  |
|  | 2.4 If Y/PY to 2.3: Were these deviations likely to have affected the outcome? | | | NA |  |
|  | 2.5. If Y/PY/NI to 2.4: Were these deviations from intended intervention balanced between groups? | | | NA |  |
|  | 2.6 Was an appropriate analysis used to estimate the effect of assignment to intervention? | | | Y |  |
|  | 2.7 If N/PN/NI to 2.6: Was there potential for a substantial impact (on the result) of the failure to analyse participants in the group to which they were randomized? | | | NA |  |
|  | **Risk of bias judgement** | | | **Low** |  |
| **Bias due to missing outcome data** | 3.1 Were data for this outcome available for all, or nearly all, participants randomized? | | | Y |  |
|  | 3.2 If N/PN/NI to 3.1: Is there evidence that result was not biased by missing outcome data? | | | NA |  |
|  | 3.3 If N/PN to 3.2: Could missingness in the outcome depend on its true value? | | | NA |  |
|  | 3.4 If Y/PY/NI to 3.3: Is it likely that missingness in the outcome depended on its true value? | | | NA |  |
|  | **Risk of bias judgement** | | | **Low** |  |
| **Bias in measurement of the outcome** | 4.1 Was the method of measuring the outcome inappropriate? | | | N |  |
|  | 4.2 Could measurement or ascertainment of the outcome have differed between intervention groups? | | | N |  |
|  | 4.3 Were outcome assessors aware of the intervention received by study participants? | | | N |  |
|  | 4.4 If Y/PY/NI to 4.3: Could assessment of the outcome have been influenced by knowledge of intervention received? | | | NA |  |
|  | 4.5 If Y/PY/NI to 4.4: Is it likely that assessment of the outcome was influenced by knowledge of intervention received? | | | NA |  |
|  | **Risk of bias judgement** | | | **Low** |  |
| **Bias in selection of the reported result** | 5.1 Were the data that produced this result analysed in accordance with a pre-specified analysis plan that was finalized before unblinded outcome data were available for analysis? | | | Y |  |
|  | 5.2 ... multiple eligible outcome measurements (e.g. scales, definitions, time points) within the outcome domain? | | | N |  |
|  | 5.3 ... multiple eligible analyses of the data? | | | N |  |
|  | **Risk of bias judgement** | | | **Low** |  |
| **Overall bias** | **Risk of bias judgement** | | | **Low** |  |
|  |  |  |  |  |  |
|  |  |  |  |  |  |
| **Unique ID** | Loughnan, Butler 2019 | **Study ID** |  | **Assessor** | I. S. Anyanwu |
| **Ref or Label** |  | **Aim** | assignment to intervention (the 'intention-to-treat' effect) |  |  |
| **Experimental** | iCBT | **Comparator** | TAU | **Source** | Journal article(s); Trial protocol |
| **Outcome** |  | **Results** |  | **Weight** | 1 |
| **Domain** | **Signalling question** | | | **Response** | **Comments** |
| **Bias arising from the randomization process** | 1.1 Was the allocation sequence random? | | | Y | Randomisation sequence (i.e., 1:1 ratio within blocks of 20) was uploaded to the server by a personnel not involved in the study. |
|  | 1.2 Was the allocation sequence concealed until participants were enrolled and assigned to interventions? | | | Y |  |
|  | 1.3 Did baseline differences between intervention groups suggest a problem with the randomization process? | | | N |  |
|  | **Risk of bias judgement** | | | **Low** |  |
| **Bias due to deviations from intended interventions** | 2.1.Were participants aware of their assigned intervention during the trial? | | | PY | The participants delivered the intervention themselves, and since they have to give their informed consent prior to participation, there is a high probability that they knew they were the experiment groups. |
|  | 2.2.Were carers and people delivering the interventions aware of participants' assigned intervention during the trial? | | | PY |  |
|  | 2.3. If Y/PY/NI to 2.1 or 2.2: Were there deviations from the intended intervention that arose because of the experimental context? | | | N |  |
|  | 2.4 If Y/PY to 2.3: Were these deviations likely to have affected the outcome? | | | NA |  |
|  | 2.5. If Y/PY/NI to 2.4: Were these deviations from intended intervention balanced between groups? | | | NA |  |
|  | 2.6 Was an appropriate analysis used to estimate the effect of assignment to intervention? | | | Y |  |
|  | 2.7 If N/PN/NI to 2.6: Was there potential for a substantial impact (on the result) of the failure to analyse participants in the group to which they were randomized? | | | NA |  |
|  | **Risk of bias judgement** | | | **Low** |  |
| **Bias due to missing outcome data** | 3.1 Were data for this outcome available for all, or nearly all, participants randomized? | | | N |  |
|  | 3.2 If N/PN/NI to 3.1: Is there evidence that result was not biased by missing outcome data? | | | PY |  |
|  | 3.3 If N/PN to 3.2: Could missingness in the outcome depend on its true value? | | | NA |  |
|  | 3.4 If Y/PY/NI to 3.3: Is it likely that missingness in the outcome depended on its true value? | | | NA |  |
|  | **Risk of bias judgement** | | | **Low** |  |
| **Bias in measurement of the outcome** | 4.1 Was the method of measuring the outcome inappropriate? | | | N |  |
|  | 4.2 Could measurement or ascertainment of the outcome have differed between intervention groups? | | | N |  |
|  | 4.3 Were outcome assessors aware of the intervention received by study participants? | | | NI |  |
|  | 4.4 If Y/PY/NI to 4.3: Could assessment of the outcome have been influenced by knowledge of intervention received? | | | PY |  |
|  | 4.5 If Y/PY/NI to 4.4: Is it likely that assessment of the outcome was influenced by knowledge of intervention received? | | | N |  |
|  | **Risk of bias judgement** | | | **Some concerns** |  |
| **Bias in selection of the reported result** | 5.1 Were the data that produced this result analysed in accordance with a pre-specified analysis plan that was finalized before unblinded outcome data were available for analysis? | | | Y |  |
|  | 5.2 ... multiple eligible outcome measurements (e.g. scales, definitions, time points) within the outcome domain? | | | N |  |
|  | 5.3 ... multiple eligible analyses of the data? | | | N |  |
|  | **Risk of bias judgement** | | | **Low** |  |
| **Overall bias** | **Risk of bias judgement** | | | **Some concerns** |  |
|  |  |  |  |  |  |
|  |  |  |  |  |  |
| **Unique ID** | Loughnan, Sie 2019 | **Study ID** |  | **Assessor** | I. S. Anyanwu |
| **Ref or Label** |  | **Aim** | assignment to intervention (the 'intention-to-treat' effect) |  |  |
| **Experimental** | iCBT | **Comparator** | TAU | **Source** | Journal article(s); Trial protocol |
| **Outcome** |  | **Results** |  | **Weight** | 1 |
| **Domain** | **Signalling question** | | | **Response** | **Comments** |
| **Bias arising from the randomization process** | 1.1 Was the allocation sequence random? | | | Y | Randomisation sequence (i.e., 1:1 ratio within blocks of 20) was uploaded to the server by a personnel not involved in the study. |
|  | 1.2 Was the allocation sequence concealed until participants were enrolled and assigned to interventions? | | | Y |  |
|  | 1.3 Did baseline differences between intervention groups suggest a problem with the randomization process? | | | N |  |
|  | **Risk of bias judgement** | | | **Low** |  |
| **Bias due to deviations from intended interventions** | 2.1.Were participants aware of their assigned intervention during the trial? | | | PY | The participants delivered the intervention themselves, and since they must give their informed consent prior to participation, there is a high probability that they knew they were the experiment groups. |
|  | 2.2.Were carers and people delivering the interventions aware of participants' assigned intervention during the trial? | | | PY |  |
|  | 2.3. If Y/PY/NI to 2.1 or 2.2: Were there deviations from the intended intervention that arose because of the experimental context? | | | N |  |
|  | 2.4 If Y/PY to 2.3: Were these deviations likely to have affected the outcome? | | | NA |  |
|  | 2.5. If Y/PY/NI to 2.4: Were these deviations from intended intervention balanced between groups? | | | NA |  |
|  | 2.6 Was an appropriate analysis used to estimate the effect of assignment to intervention? | | | Y |  |
|  | 2.7 If N/PN/NI to 2.6: Was there potential for a substantial impact (on the result) of the failure to analyse participants in the group to which they were randomized? | | | NA |  |
|  | **Risk of bias judgement** | | | **Low** |  |
| **Bias due to missing outcome data** | 3.1 Were data for this outcome available for all, or nearly all, participants randomized? | | | N |  |
|  | 3.2 If N/PN/NI to 3.1: Is there evidence that result was not biased by missing outcome data? | | | PY |  |
|  | 3.3 If N/PN to 3.2: Could missingness in the outcome depend on its true value? | | | NA |  |
|  | 3.4 If Y/PY/NI to 3.3: Is it likely that missingness in the outcome depended on its true value? | | | NA |  |
|  | **Risk of bias judgement** | | | **Low** |  |
| **Bias in measurement of the outcome** | 4.1 Was the method of measuring the outcome inappropriate? | | | N |  |
|  | 4.2 Could measurement or ascertainment of the outcome have differed between intervention groups? | | | N |  |
|  | 4.3 Were outcome assessors aware of the intervention received by study participants? | | | NI |  |
|  | 4.4 If Y/PY/NI to 4.3: Could assessment of the outcome have been influenced by knowledge of intervention received? | | | PY |  |
|  | 4.5 If Y/PY/NI to 4.4: Is it likely that assessment of the outcome was influenced by knowledge of intervention received? | | | N |  |
|  | **Risk of bias judgement** | | | **Some concerns** |  |
| **Bias in selection of the reported result** | 5.1 Were the data that produced this result analysed in accordance with a pre-specified analysis plan that was finalized before unblinded outcome data were available for analysis? | | | Y |  |
|  | 5.2 ... multiple eligible outcome measurements (e.g. scales, definitions, time points) within the outcome domain? | | | N |  |
|  | 5.3 ... multiple eligible analyses of the data? | | | N |  |
|  | **Risk of bias judgement** | | | **Low** |  |
| **Overall bias** | **Risk of bias judgement** | | | **Some concerns** |  |
|  |  |  |  |  |  |
|  |  |  |  |  |  |
| **Unique ID** | Merza 2023 | **Study ID** |  | **Assessor** | I. S. Anyanwu |
| **Ref or Label** |  | **Aim** | assignment to intervention (the 'intention-to-treat' effect) |  |  |
| **Experimental** | Internet group CBT | **Comparator** | TAU | **Source** | Journal article(s); Non-commercial trial registry record (e.g. ClinicalTrials.gov record) |
| **Outcome** |  | **Results** |  | **Weight** | 1 |
| **Domain** | **Signalling question** | | | **Response** | **Comments** |
| **Bias arising from the randomization process** | 1.1 Was the allocation sequence random? | | | Y |  |
|  | 1.2 Was the allocation sequence concealed until participants were enrolled and assigned to interventions? | | | Y |  |
|  | 1.3 Did baseline differences between intervention groups suggest a problem with the randomization process? | | | N |  |
|  | **Risk of bias judgement** | | | **Low** |  |
| **Bias due to deviations from intended interventions** | 2.1.Were participants aware of their assigned intervention during the trial? | | | PY | 1. As an online intervention, participants probably knew if they were in the experiment group or not.  2. The interventon was delivered by peers and this was not available for the TAU group, so the peers likely knew the participants' groups. |
|  | 2.2.Were carers and people delivering the interventions aware of participants' assigned intervention during the trial? | | | PY |  |
|  | 2.3. If Y/PY/NI to 2.1 or 2.2: Were there deviations from the intended intervention that arose because of the experimental context? | | | N |  |
|  | 2.4 If Y/PY to 2.3: Were these deviations likely to have affected the outcome? | | | NA |  |
|  | 2.5. If Y/PY/NI to 2.4: Were these deviations from intended intervention balanced between groups? | | | NA |  |
|  | 2.6 Was an appropriate analysis used to estimate the effect of assignment to intervention? | | | Y |  |
|  | 2.7 If N/PN/NI to 2.6: Was there potential for a substantial impact (on the result) of the failure to analyse participants in the group to which they were randomized? | | | NA |  |
|  | **Risk of bias judgement** | | | **Low** |  |
| **Bias due to missing outcome data** | 3.1 Were data for this outcome available for all, or nearly all, participants randomized? | | | Y |  |
|  | 3.2 If N/PN/NI to 3.1: Is there evidence that result was not biased by missing outcome data? | | | NA |  |
|  | 3.3 If N/PN to 3.2: Could missingness in the outcome depend on its true value? | | | NA |  |
|  | 3.4 If Y/PY/NI to 3.3: Is it likely that missingness in the outcome depended on its true value? | | | NA |  |
|  | **Risk of bias judgement** | | | **Low** |  |
| **Bias in measurement of the outcome** | 4.1 Was the method of measuring the outcome inappropriate? | | | N |  |
|  | 4.2 Could measurement or ascertainment of the outcome have differed between intervention groups? | | | N |  |
|  | 4.3 Were outcome assessors aware of the intervention received by study participants? | | | PY |  |
|  | 4.4 If Y/PY/NI to 4.3: Could assessment of the outcome have been influenced by knowledge of intervention received? | | | PY |  |
|  | 4.5 If Y/PY/NI to 4.4: Is it likely that assessment of the outcome was influenced by knowledge of intervention received? | | | N |  |
|  | **Risk of bias judgement** | | | **Some concerns** |  |
| **Bias in selection of the reported result** | 5.1 Were the data that produced this result analysed in accordance with a pre-specified analysis plan that was finalized before unblinded outcome data were available for analysis? | | | Y |  |
|  | 5.2 ... multiple eligible outcome measurements (e.g. scales, definitions, time points) within the outcome domain? | | | N |  |
|  | 5.3 ... multiple eligible analyses of the data? | | | N |  |
|  | **Risk of bias judgement** | | | **Low** |  |
| **Overall bias** | **Risk of bias judgement** | | | **Some concerns** |  |
|  |  |  |  |  |  |
|  |  |  |  |  |  |
| **Unique ID** | Milgrom 2021 | **Study ID** |  | **Assessor** | I. S. Anyanwu |
| **Ref or Label** |  | **Aim** | assignment to intervention (the 'intention-to-treat' effect) |  |  |
| **Experimental** | Internet CBT | **Comparator** | TAU | **Source** | Journal article(s) |
| **Outcome** |  | **Results** |  | **Weight** | 1 |
| **Domain** | **Signalling question** | | | **Response** | **Comments** |
| **Bias arising from the randomization process** | 1.1 Was the allocation sequence random? | | | Y |  |
|  | 1.2 Was the allocation sequence concealed until participants were enrolled and assigned to interventions? | | | Y |  |
|  | 1.3 Did baseline differences between intervention groups suggest a problem with the randomization process? | | | N |  |
|  | **Risk of bias judgement** | | | **Low** |  |
| **Bias due to deviations from intended interventions** | 2.1.Were participants aware of their assigned intervention during the trial? | | | Y |  |
|  | 2.2.Were carers and people delivering the interventions aware of participants' assigned intervention during the trial? | | | Y |  |
|  | 2.3. If Y/PY/NI to 2.1 or 2.2: Were there deviations from the intended intervention that arose because of the experimental context? | | | N |  |
|  | 2.4 If Y/PY to 2.3: Were these deviations likely to have affected the outcome? | | | NA |  |
|  | 2.5. If Y/PY/NI to 2.4: Were these deviations from intended intervention balanced between groups? | | | NA |  |
|  | 2.6 Was an appropriate analysis used to estimate the effect of assignment to intervention? | | | Y |  |
|  | 2.7 If N/PN/NI to 2.6: Was there potential for a substantial impact (on the result) of the failure to analyse participants in the group to which they were randomized? | | | NA |  |
|  | **Risk of bias judgement** | | | **Low** |  |
| **Bias due to missing outcome data** | 3.1 Were data for this outcome available for all, or nearly all, participants randomized? | | | Y |  |
|  | 3.2 If N/PN/NI to 3.1: Is there evidence that result was not biased by missing outcome data? | | | NA |  |
|  | 3.3 If N/PN to 3.2: Could missingness in the outcome depend on its true value? | | | NA |  |
|  | 3.4 If Y/PY/NI to 3.3: Is it likely that missingness in the outcome depended on its true value? | | | NA |  |
|  | **Risk of bias judgement** | | | **Low** |  |
| **Bias in measurement of the outcome** | 4.1 Was the method of measuring the outcome inappropriate? | | | N |  |
|  | 4.2 Could measurement or ascertainment of the outcome have differed between intervention groups? | | | N |  |
|  | 4.3 Were outcome assessors aware of the intervention received by study participants? | | | Y | Participants were the outcome assessors and they knew their respective intervention groups |
|  | 4.4 If Y/PY/NI to 4.3: Could assessment of the outcome have been influenced by knowledge of intervention received? | | | PY |  |
|  | 4.5 If Y/PY/NI to 4.4: Is it likely that assessment of the outcome was influenced by knowledge of intervention received? | | | N |  |
|  | **Risk of bias judgement** | | | **Some concerns** |  |
| **Bias in selection of the reported result** | 5.1 Were the data that produced this result analysed in accordance with a pre-specified analysis plan that was finalized before unblinded outcome data were available for analysis? | | | Y |  |
|  | 5.2 ... multiple eligible outcome measurements (e.g. scales, definitions, time points) within the outcome domain? | | | N |  |
|  | 5.3 ... multiple eligible analyses of the data? | | | N |  |
|  | **Risk of bias judgement** | | | **Low** |  |
| **Overall bias** | **Risk of bias judgement** | | | **Some concerns** |  |
|  |  |  |  |  |  |
|  |  |  |  |  |  |
| **Unique ID** | Monteiro 2020 | **Study ID** |  | **Assessor** | I. S. Anyanwu |
| **Ref or Label** |  | **Aim** | assignment to intervention (the 'intention-to-treat' effect) |  |  |
| **Experimental** | Web-based CBT | **Comparator** | TAU | **Source** | Journal article(s); Non-commercial trial registry record (e.g. ClinicalTrials.gov record) |
| **Outcome** |  | **Results** |  | **Weight** | 1 |
| **Domain** | **Signalling question** | | | **Response** | **Comments** |
| **Bias arising from the randomization process** | 1.1 Was the allocation sequence random? | | | Y |  |
|  | 1.2 Was the allocation sequence concealed until participants were enrolled and assigned to interventions? | | | Y |  |
|  | 1.3 Did baseline differences between intervention groups suggest a problem with the randomization process? | | | N |  |
|  | **Risk of bias judgement** | | | **Low** |  |
| **Bias due to deviations from intended interventions** | 2.1.Were participants aware of their assigned intervention during the trial? | | | Y |  |
|  | 2.2.Were carers and people delivering the interventions aware of participants' assigned intervention during the trial? | | | Y |  |
|  | 2.3. If Y/PY/NI to 2.1 or 2.2: Were there deviations from the intended intervention that arose because of the experimental context? | | | N |  |
|  | 2.4 If Y/PY to 2.3: Were these deviations likely to have affected the outcome? | | | NA |  |
|  | 2.5. If Y/PY/NI to 2.4: Were these deviations from intended intervention balanced between groups? | | | NA |  |
|  | 2.6 Was an appropriate analysis used to estimate the effect of assignment to intervention? | | | Y |  |
|  | 2.7 If N/PN/NI to 2.6: Was there potential for a substantial impact (on the result) of the failure to analyse participants in the group to which they were randomized? | | | NA |  |
|  | **Risk of bias judgement** | | | **Low** |  |
| **Bias due to missing outcome data** | 3.1 Were data for this outcome available for all, or nearly all, participants randomized? | | | N |  |
|  | 3.2 If N/PN/NI to 3.1: Is there evidence that result was not biased by missing outcome data? | | | Y |  |
|  | 3.3 If N/PN to 3.2: Could missingness in the outcome depend on its true value? | | | NA |  |
|  | 3.4 If Y/PY/NI to 3.3: Is it likely that missingness in the outcome depended on its true value? | | | NA |  |
|  | **Risk of bias judgement** | | | **Low** |  |
| **Bias in measurement of the outcome** | 4.1 Was the method of measuring the outcome inappropriate? | | | N |  |
|  | 4.2 Could measurement or ascertainment of the outcome have differed between intervention groups? | | | N |  |
|  | 4.3 Were outcome assessors aware of the intervention received by study participants? | | | Y |  |
|  | 4.4 If Y/PY/NI to 4.3: Could assessment of the outcome have been influenced by knowledge of intervention received? | | | PY |  |
|  | 4.5 If Y/PY/NI to 4.4: Is it likely that assessment of the outcome was influenced by knowledge of intervention received? | | | N |  |
|  | **Risk of bias judgement** | | | **Some concerns** |  |
| **Bias in selection of the reported result** | 5.1 Were the data that produced this result analysed in accordance with a pre-specified analysis plan that was finalized before unblinded outcome data were available for analysis? | | | Y |  |
|  | 5.2 ... multiple eligible outcome measurements (e.g. scales, definitions, time points) within the outcome domain? | | | N |  |
|  | 5.3 ... multiple eligible analyses of the data? | | | N |  |
|  | **Risk of bias judgement** | | | **Low** |  |
| **Overall bias** | **Risk of bias judgement** | | | **Some concerns** |  |
|  |  |  |  |  |  |
|  |  |  |  |  |  |
| **Unique ID** | Nishi 2022 | **Study ID** |  | **Assessor** | I. S. Anyanwu |
| **Ref or Label** |  | **Aim** | assignment to intervention (the 'intention-to-treat' effect) |  |  |
| **Experimental** | iCBT | **Comparator** | TAU | **Source** | Journal article(s); Trial protocol |
| **Outcome** |  | **Results** |  | **Weight** | 1 |
| **Domain** | **Signalling question** | | | **Response** | **Comments** |
| **Bias arising from the randomization process** | 1.1 Was the allocation sequence random? | | | Y |  |
|  | 1.2 Was the allocation sequence concealed until participants were enrolled and assigned to interventions? | | | Y |  |
|  | 1.3 Did baseline differences between intervention groups suggest a problem with the randomization process? | | | N |  |
|  | **Risk of bias judgement** | | | **Low** |  |
| **Bias due to deviations from intended interventions** | 2.1.Were participants aware of their assigned intervention during the trial? | | | PY |  |
|  | 2.2.Were carers and people delivering the interventions aware of participants' assigned intervention during the trial? | | | PY |  |
|  | 2.3. If Y/PY/NI to 2.1 or 2.2: Were there deviations from the intended intervention that arose because of the experimental context? | | | PN |  |
|  | 2.4 If Y/PY to 2.3: Were these deviations likely to have affected the outcome? | | | NA |  |
|  | 2.5. If Y/PY/NI to 2.4: Were these deviations from intended intervention balanced between groups? | | | NA |  |
|  | 2.6 Was an appropriate analysis used to estimate the effect of assignment to intervention? | | | Y |  |
|  | 2.7 If N/PN/NI to 2.6: Was there potential for a substantial impact (on the result) of the failure to analyse participants in the group to which they were randomized? | | | NA |  |
|  | **Risk of bias judgement** | | | **Low** |  |
| **Bias due to missing outcome data** | 3.1 Were data for this outcome available for all, or nearly all, participants randomized? | | | N | >20% attrition rate |
|  | 3.2 If N/PN/NI to 3.1: Is there evidence that result was not biased by missing outcome data? | | | Y |  |
|  | 3.3 If N/PN to 3.2: Could missingness in the outcome depend on its true value? | | | NA |  |
|  | 3.4 If Y/PY/NI to 3.3: Is it likely that missingness in the outcome depended on its true value? | | | NA |  |
|  | **Risk of bias judgement** | | | **Low** |  |
| **Bias in measurement of the outcome** | 4.1 Was the method of measuring the outcome inappropriate? | | | N |  |
|  | 4.2 Could measurement or ascertainment of the outcome have differed between intervention groups? | | | N |  |
|  | 4.3 Were outcome assessors aware of the intervention received by study participants? | | | PY |  |
|  | 4.4 If Y/PY/NI to 4.3: Could assessment of the outcome have been influenced by knowledge of intervention received? | | | PY |  |
|  | 4.5 If Y/PY/NI to 4.4: Is it likely that assessment of the outcome was influenced by knowledge of intervention received? | | | PN |  |
|  | **Risk of bias judgement** | | | **Some concerns** |  |
| **Bias in selection of the reported result** | 5.1 Were the data that produced this result analysed in accordance with a pre-specified analysis plan that was finalized before unblinded outcome data were available for analysis? | | | Y |  |
|  | 5.2 ... multiple eligible outcome measurements (e.g. scales, definitions, time points) within the outcome domain? | | | N |  |
|  | 5.3 ... multiple eligible analyses of the data? | | | N |  |
|  | **Risk of bias judgement** | | | **Low** |  |
| **Overall bias** | **Risk of bias judgement** | | | **Some concerns** |  |
|  |  |  |  |  |  |
|  |  |  |  |  |  |
| **Unique ID** | Sawyer 2019 | **Study ID** |  | **Assessor** | I. S. Anyanwu |
| **Ref or Label** |  | **Aim** | assignment to intervention (the 'intention-to-treat' effect) |  |  |
| **Experimental** | Mobile app CBT & parenting education | **Comparator** | TAU | **Source** | Journal article(s); Trial protocol |
| **Outcome** |  | **Results** |  | **Weight** | 1 |
| **Domain** | **Signalling question** | | | **Response** | **Comments** |
| **Bias arising from the randomization process** | 1.1 Was the allocation sequence random? | | | Y |  |
|  | 1.2 Was the allocation sequence concealed until participants were enrolled and assigned to interventions? | | | Y |  |
|  | 1.3 Did baseline differences between intervention groups suggest a problem with the randomization process? | | | PY | There were many variables that were significantly different between the two experiment groups |
|  | **Risk of bias judgement** | | | **Some concerns** |  |
| **Bias due to deviations from intended interventions** | 2.1.Were participants aware of their assigned intervention during the trial? | | | PY |  |
|  | 2.2.Were carers and people delivering the interventions aware of participants' assigned intervention during the trial? | | | Y |  |
|  | 2.3. If Y/PY/NI to 2.1 or 2.2: Were there deviations from the intended intervention that arose because of the experimental context? | | | N |  |
|  | 2.4 If Y/PY to 2.3: Were these deviations likely to have affected the outcome? | | | NA |  |
|  | 2.5. If Y/PY/NI to 2.4: Were these deviations from intended intervention balanced between groups? | | | NA |  |
|  | 2.6 Was an appropriate analysis used to estimate the effect of assignment to intervention? | | | Y |  |
|  | 2.7 If N/PN/NI to 2.6: Was there potential for a substantial impact (on the result) of the failure to analyse participants in the group to which they were randomized? | | | NA |  |
|  | **Risk of bias judgement** | | | **Low** |  |
| **Bias due to missing outcome data** | 3.1 Were data for this outcome available for all, or nearly all, participants randomized? | | | Y |  |
|  | 3.2 If N/PN/NI to 3.1: Is there evidence that result was not biased by missing outcome data? | | | NA |  |
|  | 3.3 If N/PN to 3.2: Could missingness in the outcome depend on its true value? | | | NA |  |
|  | 3.4 If Y/PY/NI to 3.3: Is it likely that missingness in the outcome depended on its true value? | | | NA |  |
|  | **Risk of bias judgement** | | | **Low** |  |
| **Bias in measurement of the outcome** | 4.1 Was the method of measuring the outcome inappropriate? | | | N |  |
|  | 4.2 Could measurement or ascertainment of the outcome have differed between intervention groups? | | | N |  |
|  | 4.3 Were outcome assessors aware of the intervention received by study participants? | | | Y |  |
|  | 4.4 If Y/PY/NI to 4.3: Could assessment of the outcome have been influenced by knowledge of intervention received? | | | PY |  |
|  | 4.5 If Y/PY/NI to 4.4: Is it likely that assessment of the outcome was influenced by knowledge of intervention received? | | | N |  |
|  | **Risk of bias judgement** | | | **Some concerns** |  |
| **Bias in selection of the reported result** | 5.1 Were the data that produced this result analysed in accordance with a pre-specified analysis plan that was finalized before unblinded outcome data were available for analysis? | | | Y |  |
|  | 5.2 ... multiple eligible outcome measurements (e.g. scales, definitions, time points) within the outcome domain? | | | N |  |
|  | 5.3 ... multiple eligible analyses of the data? | | | N |  |
|  | **Risk of bias judgement** | | | **Low** |  |
| **Overall bias** | **Risk of bias judgement** | | | **Some concerns** |  |
|  |  |  |  |  |  |
|  |  |  |  |  |  |
| **Unique ID** | Seo 2022 | **Study ID** |  | **Assessor** | I. S. Anyanwu |
| **Ref or Label** |  | **Aim** | assignment to intervention (the 'intention-to-treat' effect) |  |  |
| **Experimental** | Mobile app CBT and psychoeducation | **Comparator** | TAU | **Source** | Journal article(s) |
| **Outcome** |  | **Results** |  | **Weight** | 1 |
| **Domain** | **Signalling question** | | | **Response** | **Comments** |
| **Bias arising from the randomization process** | 1.1 Was the allocation sequence random? | | | Y |  |
|  | 1.2 Was the allocation sequence concealed until participants were enrolled and assigned to interventions? | | | Y |  |
|  | 1.3 Did baseline differences between intervention groups suggest a problem with the randomization process? | | | N |  |
|  | **Risk of bias judgement** | | | **Low** |  |
| **Bias due to deviations from intended interventions** | 2.1.Were participants aware of their assigned intervention during the trial? | | | Y |  |
|  | 2.2.Were carers and people delivering the interventions aware of participants' assigned intervention during the trial? | | | Y |  |
|  | 2.3. If Y/PY/NI to 2.1 or 2.2: Were there deviations from the intended intervention that arose because of the experimental context? | | | N |  |
|  | 2.4 If Y/PY to 2.3: Were these deviations likely to have affected the outcome? | | | NA |  |
|  | 2.5. If Y/PY/NI to 2.4: Were these deviations from intended intervention balanced between groups? | | | NA |  |
|  | 2.6 Was an appropriate analysis used to estimate the effect of assignment to intervention? | | | Y |  |
|  | 2.7 If N/PN/NI to 2.6: Was there potential for a substantial impact (on the result) of the failure to analyse participants in the group to which they were randomized? | | | NA |  |
|  | **Risk of bias judgement** | | | **Low** |  |
| **Bias due to missing outcome data** | 3.1 Were data for this outcome available for all, or nearly all, participants randomized? | | | N |  |
|  | 3.2 If N/PN/NI to 3.1: Is there evidence that result was not biased by missing outcome data? | | | PN |  |
|  | 3.3 If N/PN to 3.2: Could missingness in the outcome depend on its true value? | | | N |  |
|  | 3.4 If Y/PY/NI to 3.3: Is it likely that missingness in the outcome depended on its true value? | | | NA |  |
|  | **Risk of bias judgement** | | | **Low** |  |
| **Bias in measurement of the outcome** | 4.1 Was the method of measuring the outcome inappropriate? | | | N |  |
|  | 4.2 Could measurement or ascertainment of the outcome have differed between intervention groups? | | | N |  |
|  | 4.3 Were outcome assessors aware of the intervention received by study participants? | | | N |  |
|  | 4.4 If Y/PY/NI to 4.3: Could assessment of the outcome have been influenced by knowledge of intervention received? | | | NA |  |
|  | 4.5 If Y/PY/NI to 4.4: Is it likely that assessment of the outcome was influenced by knowledge of intervention received? | | | NA |  |
|  | **Risk of bias judgement** | | | **Low** |  |
| **Bias in selection of the reported result** | 5.1 Were the data that produced this result analysed in accordance with a pre-specified analysis plan that was finalized before unblinded outcome data were available for analysis? | | | Y |  |
|  | 5.2 ... multiple eligible outcome measurements (e.g. scales, definitions, time points) within the outcome domain? | | | N |  |
|  | 5.3 ... multiple eligible analyses of the data? | | | N |  |
|  | **Risk of bias judgement** | | | **Low** |  |
| **Overall bias** | **Risk of bias judgement** | | | **Low** |  |
|  |  |  |  |  |  |
|  |  |  |  |  |  |
| **Unique ID** | Suchan 2022 | **Study ID** |  | **Assessor** | I. S. Anyanwu |
| **Ref or Label** |  | **Aim** | assignment to intervention (the 'intention-to-treat' effect) |  |  |
| **Experimental** |  | **Comparator** |  | **Source** | Journal article(s); Non-commercial trial registry record (e.g. ClinicalTrials.gov record) |
| **Outcome** |  | **Results** |  | **Weight** | 1 |
| **Domain** | **Signalling question** | | | **Response** | **Comments** |
| **Bias arising from the randomization process** | 1.1 Was the allocation sequence random? | | | Y |  |
|  | 1.2 Was the allocation sequence concealed until participants were enrolled and assigned to interventions? | | | Y |  |
|  | 1.3 Did baseline differences between intervention groups suggest a problem with the randomization process? | | | N |  |
|  | **Risk of bias judgement** | | | **Low** |  |
| **Bias due to deviations from intended interventions** | 2.1.Were participants aware of their assigned intervention during the trial? | | | Y |  |
|  | 2.2.Were carers and people delivering the interventions aware of participants' assigned intervention during the trial? | | | Y |  |
|  | 2.3. If Y/PY/NI to 2.1 or 2.2: Were there deviations from the intended intervention that arose because of the experimental context? | | | N |  |
|  | 2.4 If Y/PY to 2.3: Were these deviations likely to have affected the outcome? | | | NA |  |
|  | 2.5. If Y/PY/NI to 2.4: Were these deviations from intended intervention balanced between groups? | | | NA |  |
|  | 2.6 Was an appropriate analysis used to estimate the effect of assignment to intervention? | | | Y |  |
|  | 2.7 If N/PN/NI to 2.6: Was there potential for a substantial impact (on the result) of the failure to analyse participants in the group to which they were randomized? | | | NA |  |
|  | **Risk of bias judgement** | | | **Low** |  |
| **Bias due to missing outcome data** | 3.1 Were data for this outcome available for all, or nearly all, participants randomized? | | | Y |  |
|  | 3.2 If N/PN/NI to 3.1: Is there evidence that result was not biased by missing outcome data? | | | NA |  |
|  | 3.3 If N/PN to 3.2: Could missingness in the outcome depend on its true value? | | | NA |  |
|  | 3.4 If Y/PY/NI to 3.3: Is it likely that missingness in the outcome depended on its true value? | | | NA |  |
|  | **Risk of bias judgement** | | | **Low** |  |
| **Bias in measurement of the outcome** | 4.1 Was the method of measuring the outcome inappropriate? | | | N |  |
|  | 4.2 Could measurement or ascertainment of the outcome have differed between intervention groups? | | | N |  |
|  | 4.3 Were outcome assessors aware of the intervention received by study participants? | | | NI |  |
|  | 4.4 If Y/PY/NI to 4.3: Could assessment of the outcome have been influenced by knowledge of intervention received? | | | PY |  |
|  | 4.5 If Y/PY/NI to 4.4: Is it likely that assessment of the outcome was influenced by knowledge of intervention received? | | | N |  |
|  | **Risk of bias judgement** | | | **Some concerns** |  |
| **Bias in selection of the reported result** | 5.1 Were the data that produced this result analysed in accordance with a pre-specified analysis plan that was finalized before unblinded outcome data were available for analysis? | | | Y |  |
|  | 5.2 ... multiple eligible outcome measurements (e.g. scales, definitions, time points) within the outcome domain? | | | N |  |
|  | 5.3 ... multiple eligible analyses of the data? | | | N |  |
|  | **Risk of bias judgement** | | | **Low** |  |
| **Overall bias** | **Risk of bias judgement** | | | **Some concerns** |  |
|  |  |  |  |  |  |
|  |  |  |  |  |  |
| **Unique ID** | Suharwardy 2023 | **Study ID** |  | **Assessor** | I. S. Anyanwu |
| **Ref or Label** |  | **Aim** | assignment to intervention (the 'intention-to-treat' effect) |  |  |
| **Experimental** | App-based CBT | **Comparator** | TAU | **Source** | Journal article(s); Non-commercial trial registry record (e.g. ClinicalTrials.gov record) |
| **Outcome** |  | **Results** |  | **Weight** | 1 |
| **Domain** | **Signalling question** | | | **Response** | **Comments** |
| **Bias arising from the randomization process** | 1.1 Was the allocation sequence random? | | | Y |  |
|  | 1.2 Was the allocation sequence concealed until participants were enrolled and assigned to interventions? | | | Y |  |
|  | 1.3 Did baseline differences between intervention groups suggest a problem with the randomization process? | | | N |  |
|  | **Risk of bias judgement** | | | **Low** |  |
| **Bias due to deviations from intended interventions** | 2.1.Were participants aware of their assigned intervention during the trial? | | | PY |  |
|  | 2.2.Were carers and people delivering the interventions aware of participants' assigned intervention during the trial? | | | PY |  |
|  | 2.3. If Y/PY/NI to 2.1 or 2.2: Were there deviations from the intended intervention that arose because of the experimental context? | | | N |  |
|  | 2.4 If Y/PY to 2.3: Were these deviations likely to have affected the outcome? | | | NA |  |
|  | 2.5. If Y/PY/NI to 2.4: Were these deviations from intended intervention balanced between groups? | | | NA |  |
|  | 2.6 Was an appropriate analysis used to estimate the effect of assignment to intervention? | | | Y |  |
|  | 2.7 If N/PN/NI to 2.6: Was there potential for a substantial impact (on the result) of the failure to analyse participants in the group to which they were randomized? | | | NA |  |
|  | **Risk of bias judgement** | | | **Low** |  |
| **Bias due to missing outcome data** | 3.1 Were data for this outcome available for all, or nearly all, participants randomized? | | | N |  |
|  | 3.2 If N/PN/NI to 3.1: Is there evidence that result was not biased by missing outcome data? | | | N |  |
|  | 3.3 If N/PN to 3.2: Could missingness in the outcome depend on its true value? | | | N |  |
|  | 3.4 If Y/PY/NI to 3.3: Is it likely that missingness in the outcome depended on its true value? | | | NA |  |
|  | **Risk of bias judgement** | | | **Low** |  |
| **Bias in measurement of the outcome** | 4.1 Was the method of measuring the outcome inappropriate? | | | N |  |
|  | 4.2 Could measurement or ascertainment of the outcome have differed between intervention groups? | | | N |  |
|  | 4.3 Were outcome assessors aware of the intervention received by study participants? | | | Y |  |
|  | 4.4 If Y/PY/NI to 4.3: Could assessment of the outcome have been influenced by knowledge of intervention received? | | | PY |  |
|  | 4.5 If Y/PY/NI to 4.4: Is it likely that assessment of the outcome was influenced by knowledge of intervention received? | | | N |  |
|  | **Risk of bias judgement** | | | **Some concerns** |  |
| **Bias in selection of the reported result** | 5.1 Were the data that produced this result analysed in accordance with a pre-specified analysis plan that was finalized before unblinded outcome data were available for analysis? | | | Y |  |
|  | 5.2 ... multiple eligible outcome measurements (e.g. scales, definitions, time points) within the outcome domain? | | | N |  |
|  | 5.3 ... multiple eligible analyses of the data? | | | N |  |
|  | **Risk of bias judgement** | | | **Low** |  |
| **Overall bias** | **Risk of bias judgement** | | | **Some concerns** |  |
|  |  |  |  |  |  |
|  |  |  |  |  |  |
| **Unique ID** | Van 2021 | **Study ID** |  | **Assessor** | I. S. Anyanwu |
| **Ref or Label** |  | **Aim** | assignment to intervention (the 'intention-to-treat' effect) |  |  |
| **Experimental** | Online 1-day CBT | **Comparator** | TAU | **Source** | Journal article(s); Trial protocol; Non-commercial trial registry record (e.g. ClinicalTrials.gov record) |
| **Outcome** |  | **Results** |  | **Weight** | 1 |
| **Domain** | **Signalling question** | | | **Response** | **Comments** |
| **Bias arising from the randomization process** | 1.1 Was the allocation sequence random? | | | Y |  |
|  | 1.2 Was the allocation sequence concealed until participants were enrolled and assigned to interventions? | | | Y |  |
|  | 1.3 Did baseline differences between intervention groups suggest a problem with the randomization process? | | | N |  |
|  | **Risk of bias judgement** | | | **Low** |  |
| **Bias due to deviations from intended interventions** | 2.1.Were participants aware of their assigned intervention during the trial? | | | PY |  |
|  | 2.2.Were carers and people delivering the interventions aware of participants' assigned intervention during the trial? | | | PY |  |
|  | 2.3. If Y/PY/NI to 2.1 or 2.2: Were there deviations from the intended intervention that arose because of the experimental context? | | | N |  |
|  | 2.4 If Y/PY to 2.3: Were these deviations likely to have affected the outcome? | | | NA |  |
|  | 2.5. If Y/PY/NI to 2.4: Were these deviations from intended intervention balanced between groups? | | | NA |  |
|  | 2.6 Was an appropriate analysis used to estimate the effect of assignment to intervention? | | | Y |  |
|  | 2.7 If N/PN/NI to 2.6: Was there potential for a substantial impact (on the result) of the failure to analyse participants in the group to which they were randomized? | | | NA |  |
|  | **Risk of bias judgement** | | | **Low** |  |
| **Bias due to missing outcome data** | 3.1 Were data for this outcome available for all, or nearly all, participants randomized? | | | N |  |
|  | 3.2 If N/PN/NI to 3.1: Is there evidence that result was not biased by missing outcome data? | | | PY |  |
|  | 3.3 If N/PN to 3.2: Could missingness in the outcome depend on its true value? | | | NA |  |
|  | 3.4 If Y/PY/NI to 3.3: Is it likely that missingness in the outcome depended on its true value? | | | NA |  |
|  | **Risk of bias judgement** | | | **Low** |  |
| **Bias in measurement of the outcome** | 4.1 Was the method of measuring the outcome inappropriate? | | | N |  |
|  | 4.2 Could measurement or ascertainment of the outcome have differed between intervention groups? | | | N |  |
|  | 4.3 Were outcome assessors aware of the intervention received by study participants? | | | NI |  |
|  | 4.4 If Y/PY/NI to 4.3: Could assessment of the outcome have been influenced by knowledge of intervention received? | | | NI |  |
|  | 4.5 If Y/PY/NI to 4.4: Is it likely that assessment of the outcome was influenced by knowledge of intervention received? | | | N |  |
|  | **Risk of bias judgement** | | | **Some concerns** |  |
| **Bias in selection of the reported result** | 5.1 Were the data that produced this result analysed in accordance with a pre-specified analysis plan that was finalized before unblinded outcome data were available for analysis? | | | Y |  |
|  | 5.2 ... multiple eligible outcome measurements (e.g. scales, definitions, time points) within the outcome domain? | | | N |  |
|  | 5.3 ... multiple eligible analyses of the data? | | | N |  |
|  | **Risk of bias judgement** | | | **Low** |  |
| **Overall bias** | **Risk of bias judgement** | | | **Some concerns** |  |
|  |  |  |  |  |  |
|  |  |  |  |  |  |
| **Unique ID** | Zhang, Li 2023 | **Study ID** |  | **Assessor** | I. S. Anyanwu |
| **Ref or Label** |  | **Aim** | assignment to intervention (the 'intention-to-treat' effect) |  |  |
| **Experimental** | Wechat Mindfulness | **Comparator** | TAU | **Source** | Journal article(s) |
| **Outcome** |  | **Results** |  | **Weight** | 1 |
| **Domain** | **Signalling question** | | | **Response** | **Comments** |
| **Bias arising from the randomization process** | 1.1 Was the allocation sequence random? | | | Y |  |
|  | 1.2 Was the allocation sequence concealed until participants were enrolled and assigned to interventions? | | | Y |  |
|  | 1.3 Did baseline differences between intervention groups suggest a problem with the randomization process? | | | N |  |
|  | **Risk of bias judgement** | | | **Low** |  |
| **Bias due to deviations from intended interventions** | 2.1.Were participants aware of their assigned intervention during the trial? | | | Y |  |
|  | 2.2.Were carers and people delivering the interventions aware of participants' assigned intervention during the trial? | | | Y |  |
|  | 2.3. If Y/PY/NI to 2.1 or 2.2: Were there deviations from the intended intervention that arose because of the experimental context? | | | N |  |
|  | 2.4 If Y/PY to 2.3: Were these deviations likely to have affected the outcome? | | | NA |  |
|  | 2.5. If Y/PY/NI to 2.4: Were these deviations from intended intervention balanced between groups? | | | NA |  |
|  | 2.6 Was an appropriate analysis used to estimate the effect of assignment to intervention? | | | Y |  |
|  | 2.7 If N/PN/NI to 2.6: Was there potential for a substantial impact (on the result) of the failure to analyse participants in the group to which they were randomized? | | | NA |  |
|  | **Risk of bias judgement** | | | **Low** |  |
| **Bias due to missing outcome data** | 3.1 Were data for this outcome available for all, or nearly all, participants randomized? | | | Y |  |
|  | 3.2 If N/PN/NI to 3.1: Is there evidence that result was not biased by missing outcome data? | | | NA |  |
|  | 3.3 If N/PN to 3.2: Could missingness in the outcome depend on its true value? | | | NA |  |
|  | 3.4 If Y/PY/NI to 3.3: Is it likely that missingness in the outcome depended on its true value? | | | NA |  |
|  | **Risk of bias judgement** | | | **Low** |  |
| **Bias in measurement of the outcome** | 4.1 Was the method of measuring the outcome inappropriate? | | | N |  |
|  | 4.2 Could measurement or ascertainment of the outcome have differed between intervention groups? | | | N |  |
|  | 4.3 Were outcome assessors aware of the intervention received by study participants? | | | N |  |
|  | 4.4 If Y/PY/NI to 4.3: Could assessment of the outcome have been influenced by knowledge of intervention received? | | | NA |  |
|  | 4.5 If Y/PY/NI to 4.4: Is it likely that assessment of the outcome was influenced by knowledge of intervention received? | | | NA |  |
|  | **Risk of bias judgement** | | | **Low** |  |
| **Bias in selection of the reported result** | 5.1 Were the data that produced this result analysed in accordance with a pre-specified analysis plan that was finalized before unblinded outcome data were available for analysis? | | | Y |  |
|  | 5.2 ... multiple eligible outcome measurements (e.g. scales, definitions, time points) within the outcome domain? | | | N |  |
|  | 5.3 ... multiple eligible analyses of the data? | | | N |  |
|  | **Risk of bias judgement** | | | **Low** |  |
| **Overall bias** | **Risk of bias judgement** | | | **Low** |  |

# SUPPLEMENTARY FILE - EXTRACTED DATA FROM STUDIES INCLUDED IN THIS REVIEW

| **First author and year^a^** | **Aim, Country  (Economic region), Study Design** | **Role in review** | **Age, Sample size, and Pregnancy status** | **Eligibility criteria** | **Platform/Channel and Component** | **Purpose and Guiding theory for the DHI** | **Delivery, Delivery mode, Frequency, Facilitation mode, and How integrity/fidelity was maintained** | **Timing** | **Comparators' intervention** | **Relevant Outcome Measurement Tools** | **Depression** |
| --- | --- | --- | --- | --- | --- | --- | --- | --- | --- | --- | --- |
| Abujilban 2023 [64] | **Aim:** To compare the depressive symptom level(s) among Jordanian pregnant women who received IPT treatment with those who received routine antenatal care.  **Country:** Jordan (LMIC)  **Study Design:** RCT | SR & MA | **Age:** 24-37  **Sample size:** Total = 104 Trial = 53 Control = 51  **Pregnancy status:** Pregnant (Mean gestation age =31.3 weeks) | **Inclusion:** (a) Pregnant women in their 24th to 37th gestational week;  (b) currently experiencing depressive symptoms;  (c) between the age of 18 and 42 years old; and  (d) married with singleton and multiparous pregnancy  **Exclusion:** (a) women with any previously diagnosed mental health problems (including anxiety); (b) who were receiving antidepressant medication;  (c) who were receiving psychotherapy or supportive therapy for their depressive symptoms; or  (d) currently not living with their husbands. | **Channel:** Telephone (calls)  **Component:** IPT | **Purpose:** Treatment delivery option  **Guiding Theory:** N/S | **Delivery:** Therapy consisted of three phases: initial/orientation (1 session), intermediate (4 sessions), and closing (2 sessions). Each session = 30 minutes  **Delivery mode:** Individual  **Frequency:** 2 sessions per week  **Facilitation mode:** Therapist-facilitated  **How Integrity was maintained:** The primary therapist trained and supervised the other two psychotherapists on how to apply the program throughout the treatment period. | **Time of initiation:** Antepartum  **Duration:** 1 month (4 weeks)  **Period covered by intervention:** Antenatal only | TAU | **Depression:** EPDS (possible range: 0-30; cut-off score = 12) | Mean ± SD  DHI: Pre-test EPDS = 19.34 ± 3.59 Post-test EPDS = 8.88 ± 5.82  TAU Pre-test = 17.82 ± 2.79 Post-test = 16.42 ± 4.35 |
| Arakawa 2023 [65] | **Aim:** To examine the effectiveness of mHealth professional consultation services in preventing postpartum depressive symptoms.  **Country:** Japan (HIC)  **Study Type:** RCT | SR only | **Age:** 32.9 ± 4.1  **Sample size:** Total randomised = 734 DHI (enrolled & received intervention) = 338 Control (enrolled & received TAU) = 354 DHI (completed) = 310 Control (completed) = 329  **Pregnancy status:** Pregnant (all gestational age) | **Inclusion:** (a) Self-reported pregnancy whose expected date of delivery was until October 31, 2021 (b) Living in Yokohama city (c) Able to communicate in Japanese  **Exclusion:** None | **Channel:** Multi-options, via either voice calling, text messaging/chat, or video calling  **Component:** Teleconsultation | **Purpose:** To prevent depression  **Guiding theory:** N/S | **Delivery:** The teleconsultation was general consultation and emotional support related to pregnancy and childcare but excluding formal diagnosis or prescription.  Participants could book available 10-min consultation times and select their preferred available consultants and methods between 6 p.m. and 10 p.m. on weekdays.  After childbirth, they could also have chat consultations with midwives without booking or time restrictions between 1 p.m. and 5 p.m. on Mondays, Wednesdays, and Fridays.  The service provider regularly sent magazines to all users with helpful information on maternity and childcare as a push-type service, with advice to promote service use for women who needed help.  **Delivery mode:** Individual  **Frequency:** Unlimited  **Facilitation mode:** Therapist-facilitated  **How Integrity was maintained:** The service provider had a quality control team and conducted quality assessments for consultants to improve their services. | **Time of initiation:** Antepartum  **Duration:** Unlimited  **Period covered by intervention:** Antenatal and Postnatal | TAU | **Depression:** EPDS (possible range: 0-30; cut-off score = ≥9) | DHI: Baseline = 20.7% proportion 3 months follow-up = 4.7 ±3.9 (15.2% proportion)  TAU: Baseline = 21.9% proportion 3 months follow-up = 5.6 ± 4.5 (22.8% proportion) |
| Asadzadeh 2020 [66] | **Aim:** To investigate the effectiveness of Gamble and colleagues’ midwife-led brief counseling intervention on reducing PTSD, depression, and anxiety symptoms  **Country:** Iran (LMIC)  **Study Type:** RCT | SR & MA | **Age:** DHI = 25.38 ± 4.56 Control = 25.62 ± 4.48  **Sample size:** Total randomised = 90 DHI (enrolled) = 45 Control (enrolled) = 45 DHI (completed) = 44 Control (completed) = 43  **Pregnancy status:** Pregnant women in their third trimester | **Inclusion:** (a) 18 to 35 years old (b) Able to speak and read Persian (c) In the last pregnancy trimester, and  (d) Having a single embryo.  **Exclusion:** (a) Score ≥ 10 on the Edinburgh postnatal depression scale (b) History of abortion and infertility (c) Mental or physical chronic diseases (d) Taking medicine that causes symptoms of depression (e) History of postpartum depression in the first-degree relatives, and  (f) Experience of a major stressful event during the past year. | **Channel:** Hybrid: Physical & Telephone (calls)  **Component:** Counselling | **Purpose:** Postpartum support **Guiding theory:** N/S | **Delivery:** First, a face-to-face counselling session by the first author within 72 h after their childbirth.  Then, a telephone counselling session at four to 6 weeks after giving birth.  Each session lasted 40 to 60 min.  In addition, the intervention group could contact their midwife between the two sessions by telephone.   **Delivery mode:** Individual  **Frequency:** Minimum of 2 sessions  **Facilitation mode:** Therapist-facilitated  **How Integrity was maintained:** Sessions were randomly recorded and listened by the fourth author to make sure that the intervention is in accordance with the principles of Gamble and colleagues’ protocol. | **Time of initiation:** 72 hours postpartum  **Duration:** 4-6 weeks  **Period covered by intervention:** Postnatal only | TAU | **Depression:** EPDS (possible range: 0-30; cut-off score = 14 for antenatal and 12 for postnatal) | Mean ± SD  DHI: Baseline = 7.52 ± 2.79 Post-intervention = 3.43 ± 1.93 Follow-up = 1.25 ± 0.88  TAU Baseline = 7.30 ± 4.2 Post-intervention = 7 ± 3.25 Follow-up = 4 ± 1.91 |
| Avalos 2020 [99] | **Aim:** To assess the feasibility, acceptability, and preliminary efficacy of an mHealth mindfulness intervention for postpartum women with moderate to moderately severe depressive symptoms.  **Country:** United States (HIC)  **Study Type:** Mixed-methods; single-arm trial | SR only | **Age:** 30.9 ± 5.2  **Sample size:** Enrolled = 27 Completed = 19  **Pregnancy status:** Postpartum | **Inclusion:** (a) Aged at least 18 years (b) Within 6 months of giving birth (c) With a PHQ-9 score of 10 to 19 (indicating moderate to moderately severe depressive symptoms) (d) English-speaking (e) With access to a smartphone, tablet, or computer with internet access.  **Exclusion:** (a) Engaged in regular mindfulness, meditation, or yoga practice 3 or more times per week (b) Enrolled in a mindfulness program | **Channel:** Mobile application  **Component:** Mindfulness | **Purpose:** Treatment delivery option **Guiding Theory:** N/S | **Delivery:** The women were asked to use the app daily during the study period. Each participant was given a study-specific log-in ID and encouraged to complete the 30-day Basics course first and then choose from the other themed sessions (e.g., anxiety, relationships) for the remainder of the study period.  **Delivery mode:** Individual  **Frequency:** 10-20 minutes per day  **Facilitation mode:** Self-guided  **How Integrity was maintained:** N/A | **Time of initiation:** Postpartum  **Duration:** 6 weeks  **Period covered by intervention:** Postnatal only | N/A | **Depression:** PHQ-8 (possible range: 0-24; 1-4 = minimal depression; 5-9 = mild depression; 10-14 = moderate depression; 15-19 = moderately severe depression, and 20-24 = severe depression) | Baseline = 15.2 6 weeks follow-up = 11.4 |
| Boyd 2019 [67] | **Aim:** To describe the adaptation of a parenting group intervention for social media, and examine the feasibility, acceptability and initial outcomes of the adapted intervention for mothers with postpartum depression symptoms.  **Country:** United States (HIC)  **Study Type:** RCT | SR & MA | **Age:** DHI group: 26.4 ± 1.9 Control: 26.3 ± 1.8  **Sample size:** Total randomised = 24 DHI (enrolled) = 12 Control (enrolled) = 12 DHI (completed) = 12 Control (completed) = 8  **Pregnancy status:** Postpartum | **Inclusion:** (a) Be at least 15 years old (b) Have a child between 1 and 3 months old (c) Have internet access on a computer or a smart phone, and (d) screen positive for depressive symptoms.  **Exclusion:** (a) expressed suicidal intent | **Channel:** Social media (Facebook group)  **Component:** Psychoeducation & Behavioural activation | **Purpose:** Treatment delivery option  **Guiding theory:** N/S | **Delivery:** Included eight weekly sessions on depression psychoeducation and behavioural activation, alongside parenting knowledge. Each weekly session was organised into a narrated PowerPoint presentation, a video clip if appropriate, questions inviting participants to share their prior experiences, an exercise to promote understanding of the topic, follow-up questions on what they learned from the exercise and summary content to review lessons learned. Each week, the content is uploaded in three instalments to allow participants the time to read the material, practise the exercises and post comments/answers to the questions posed by the facilitator.  **Delivery mode:** Group  **Frequency:** One session weekly but posted in 3 instalments  **Facilitation mode:** Therapist-assisted  **How Integrity was maintained:** N/S | **Time of initiation:** Postpartum  **Duration:** 8 weeks  **Period covered by intervention:** Postnatal only | In-person group delivery of the same component | **Depression:** BDI-II (possible range: 0-63; higher scores representing more severe symptoms). | Mean ± SD  DHI: Baseline = 29.5 ± 2.3 Post-intervention = 20.2 ± 2.2  TAU Baseline = 23.4 ± 3.3 Post-intervention = 23.3 ± 4.9 |
| Bryant 2023 [100] | **Aim:** To assess the effects of a prototype of the Joyuus tool in a limited sample to assess the key domains (social support, resilience, depression, anxiety, and COVID-19 mental health impacts) and trends related to treatment effect.  **Country:** United States (HIC)  **Study Type:** Mixed-methods; single-arm trial | SR only | **Age:** Mean = 30  **Sample size:** Enrolled = 87 Completed = 79  **Pregnancy status:** Mixed (pregnant and postpartum | **Inclusion:** (a) An expectant or new mother (at least 28 weeks/6 months pregnant up to 1-year post-birth) (b) 18 years or older (c) Identified as non-white.  (d) Able to read, write, and speak English  (e) Have a smartphone with internet  **Exclusion:** N/S | **Channel:** Mobile web application  **Component:** (a) Mixed educational resources (b) Self-guided interventions, and (c) Referral links | **Purpose:** Postpartum support  **Guiding theory:** N/S | **Delivery:** Nothing specific; participants were simply assigned the Joyuus prototype to use for one month and completed a post-test after one month of accessing the tool.  **Delivery mode:** Individual  **Frequency:** N/S  **Facilitation mode:** Self-guided  **How Integrity was maintained:** N/S | **Time of initiation:** Antepartum & Postpartum (depending on participant's stage)  **Duration:** 1 month  **Period covered by intervention:** Both antenatal & postnatal | N/A | **Depression:** EPDS (Possible score range: 0-10, cut-off score = ≥10 | Pre-test = 8.94 ± 5.47 Post-test = 8.70 ± 5.07 p-value = 0.624 |
| Buultjens 2023 [101] | **Aim:** To explore a hybrid model of pregnancy care, including telehealth and fewer in-person health assessments, coupled with concurrent small group interdisciplinary education delivered via video conferencing, extending into the postnatal period.  **Country:** Australia (HIC)   **Study Type:** Quasi-experimental; double arm | SR only | **Age:** 18-40  **Sample size:** Total enrolled = 69 DHI (baseline) = 43 Control (baseline) = 26 DHI (completed) = 31 Control (completed) = 22  **Pregnancy status:** Pregnant | **Inclusion:** (a) First pregnancy (b) Aged ≥ 18 years (c) ≥ 12-weeks of gestation at time of recruitment (d) No history of mental illness (as declared by woman at booking-in appointment with midwife) (e) Deemed medically low risk by obstetrician by 28-weeks’ gestation (f) Must be able to read, understand and speak English  **Exclusion:** N/S | **Channel:** Hybrid: Physical & Online (video conferencing)  **Component:** (a) Interdisciplinary education (b) Peer support, and (c) Referral and access to women’s health interdisciplinary professionals | **Purpose:** Pregnancy care  **Guiding theory:** N/S | **Delivery:** The perinatal care, education and support (PECS) intervention consisted of four group education sessions antenatally, and four group education sessions postnatally, with six to eight women in each group. The women were grouped based on their estimated delivery date. The four antenatal sessions were delivered at gestation ages of 30–32, 33–34, 35–37, and 38–40 weeks, respectively. The respective postnatal sessions were delivered at postpartum period of 2-4, 5-7, 8-10, and 12-14 weeks. The last postnatal session was delivered in-person. The interdisciplinary team comprised midwife, dietitian, psychologist, physiotherapist, yoga/occupational therapist, and lactation consultant. The sessions delivered by the lactation consultant were only present in the postnatal sessions.  **Delivery mode:** Hybrid: one-on-one physical clinical care and group online education   **Frequency:** N/S  **Facilitation mode:** Therapist-assisted  **How Integrity was maintained:** N/S | **Time of initiation:** Antepartum  **Duration:** 24 weeks (deducted)  **Period covered by intervention:** Antenatal & postnatal | TAU | **Depression:** EPDS (possible score range: 0-30, cut-off score = ≥13) | N/S |
| Carona 2023 [68] | **Aim:** To assess the efficacy of Be a Mom in reducing depressive and anxiety symptoms of women at high risk for PPD and to examine mechanisms of change linking modifiable self-regulatory skills to improved perinatal mental health outcomes.  **Country:** Portugal (HIC)  **Study Type:** RCT | SR & MA | **Age:** DHI = 32.91 ± 4.40 Control = 32.81 ± 4.66  **Sample size:** Total randomised = 1,053 DHI (enrolled) = 542 Control (enrolled) = 511 DHI (completed) = 267 Control (completed) = 445  **Pregnancy status:** Postpartum | **Inclusion:** (a) Being an adult woman (aged ≥18 years) (b) In the early postpartum period (up to 3 months postpartum) (c) Presenting high risk for PPD (a score of ≥5.5 on the PDPI-R scale (d) Having a computer or tablet device or smartphone and internet access at home (e) Having the ability to read and speak Portuguese (f) being a resident of Portugal.  **Exclusion:** (a) The presence of a severe medically diagnosed health condition in the infant or in the mother as self-reported by mothers. | **Channel:** Web-based  **Component:** CBT | **Purpose:** Prevention of PPD in women at risk or present early-onset PPD symptoms  **Guiding theory:** N/S | **Delivery:** The women had access to the 5 modules (changes and emotional reactions; cognitions; values and social support; couple relationship (presented only presented to women in a relationship); and PPD alert signs and professional help seeking). Each module was approximately 45 minutes long.  Web link: <https://beamom.pt/>  Email reminders were sent automatically to the participants if they went 3, 7, and 13 days without accessing the program. Asynchronous communication channels were provided for program-related support only.  **Delivery mode:** Individual  **Frequency:** 1 module per week (or slower, but with a maximum allowance of completion of all 5 modules within 8 weeks)  **Facilitation mode:** Hybrid (predominantly self-guided and supported with a weekly coach call)  **How Integrity was maintained:** N/S | **Time of initiation:** Postpartum  **Duration:** 5-8 weeks  **Period covered by intervention:** Postnatal only | TAU | **Depression:** EPDS (possible range: 0-30, higher scores indicative of higher depressive symptoms) | Mean ± SD  DHI: Baseline = 10.99 ± 5.14 Post-intervention = 8.75 ± 4.53  TAU Baseline = 11.73 ± 4.81 Post-intervention = 10.78 ± 5.06 |
| Chan 2019 [69] | **Aim:** To examine whether a smartphone-based psychoeducation would lead to reduced postnatal depression, anxiety, or stress and result in a better health-related quality of life.  **Country:** Hong Kong (HIC)  **Study Type:** RCT | SR & MA | **Age:** DHI = 31.3 ± 4.6 Control = 31.2 ± 4.5  **Sample size:** Total randomised = 660 DHI (enrolled and completed baseline assessment) = 330 Control (enrolled and completed baseline assessment) = 330 DHI (completed) = 218 Control (completed) = 225  **Pregnancy status:** Pregnant | **Inclusion:** (a) First-time expectant mothers receiving regular antenatal care services at the hospital (b) Able to read and understand Chinese or English (c) Willing to consent to the terms of the study.  **Exclusion:** (a) Unable to give informed written consent or communicate with the interviewers. | **Channel:** Mobile application  **Component:** Regular antenatal education | **Purpose:** Preventing PPD  **Guiding theory:** N/S | **Delivery:** App provided between the first antenatal visit and childbirth. All materials presented in the app were equivalent to those offered in the face-to-face nurse-led antenatal TAU classes and includes articles about nutrition, infant caring, and vaccine injections for infants, as well as videos demonstrating what expectant mothers might face when delivering their baby. Furthermore, a platform within the app allowed users to ask questions related to pregnancy, childbirth, and infant health and care. All questions were answered by obstetricians via private, direct messages within the app and then shared in the Frequently Asked Questions module of the app, if permitted by the user and after personal identifiable information was removed. This function dealt only with regular and nonemergent consultations.  **Delivery mode:** Individual  **Frequency:** N/S  **Facilitation mode:** Self-guided  **How Integrity was maintained:** N/S | **Time of initiation:** Antenatal  **Duration:** Non-specific  **Period covered by intervention:** Antenatal only | TAU | **Depression:** EPDS (possible range: 0-30, higher scores indicative of higher depressive symptoms) | Mean ± SD  DHI: Baseline = 7.3 ± 4.6 Post-intervention = 5.3 ± 4.4  TAU Baseline = 7.2 ± 4.6 Post-intervention = 5.9 ± 4.7  p = 0.049 |
| Coo 2023 [70] | **Aim:** To assess the feasibility, acceptability and preliminary effectiveness of m-WWWT in a community sample of Chilean first-time mothers.  **Country:** Chile (HIC)   **Study Type:** Mixed-methods, RCT | SR only | **Age:** DHI = 25.56 ± 4.1 Control = 24.98 ± 5.2  **Sample size:** Total randomised = 128 DHI (enrolled) = 65 Control (enrolled) = 63 DHI (completed baseline assessment) = 59 Control (completed baseline assessment) = 57 DHI (completed) = 54 Control (completed) = 51  **Pregnancy status:** Postpartum | **Inclusion:** (a) Adult (>18 years) (b) First-time mothers to a full-term infant between 4 and 10 weeks old. (c) No medical condition(s) that required treatment and/or hospitalization in both infant and mothers (d) Received health care in the primary public health system. (e) Access to a mobile phone with an instant messaging service (i.e., WhatsApp) (f) Fluent in Spanish.  **Exclusion:** (a) Presenting severe mental health problems (b) Current psychiatric diagnosis of a mental health disorder (c) Psychosocial problems (i.e., domestic violence problems, cognitive disability), as reported by the psychologists of the involved health centres at the moment of recruitment. | **Channel:** Instant messaging platform (WhatsApp)  **Component:** (a) Psychoeducational information (b) Professional support (c) Referral links | **Purpose:** Preventing PPD  **Guiding theory:** N/S | **Delivery:** Includes three components: psychoeducational information, individual contact with the program facilitator to ask questions that arose from the psychoeducational modules; and a group, 1-hr virtual meeting with the program facilitator to offer participants and their partners (or support person) the opportunity to share their experiences about parenthood with other mothers.  The psychoeducational information was delivered in 14 modules with a 3- to 5-min video, and an invitation to read and discuss some questions to promote self-reflection. Women initiated contact voluntarily and the facilitator tailored the answers according to women’s individual needs. The group session was offered three times to the women, who were grouped in cohorts of 15–20 participants.  **Delivery mode:** Hybrid: individual and group components  **Frequency:** 3 times a week for a 5-week for the psychoeducational information  **Facilitation mode:** Therapist-facilitated  **How Integrity was maintained:**  N/S | **Time of initiation:** Postpartum  **Duration:** 5 weeks  **Period covered by intervention:** Postnatal only | TAU | **Depression:** EPDS (possible score range: 0-30, cut-off score = ≥11) | Mean ± SD  DHI: Baseline = 8.2 ± 4.93 Post-intervention = 8.84 ± 5.76  TAU Baseline = 8.67 ± 5.2 Post-intervention = 8.63 ± 5.72 |
| Danaher 2023 [71] | **Aim:** To evaluate the effectiveness of a perinatal version of MomMoodBooster encompassing both prenatal and postpartum content in a healthcare delivery setting already providing universal screening and referral of at-risk patients as part of routine care.  **Country:** United States (HIC)  **Study Type:** RCT | SR & MA | **Age:** 32  **Sample size:** Total randomised = 191 DHI (enrolled) = 96 Control (enrolled) = 95 DHI (completed) = 86 Control (completed) = 92  **Pregnancy status:** Both pregnant and postpartum | **Inclusion:** (a) Pregnant or <1 year postpartum (b) ≥18 years of age (c) No active suicidal ideation (d) Access to broadband internet via desktop/laptop, tablet, or smartphone (e) Proficient use of English language (f) Women with affirmative answers to the EPDS self-harm item were included in the study if social work assessment deemed them low-risk for suicide.  **Exclusion:** Patients with active suicidal ideation | **Channel:** Web application  **Component:** CBT | **Purpose:** Treatment delivery option  **Guiding theory:** N/S | **Delivery:** During the 12-week active treatment phase, each of the 6 MMB2 sessions became available sequentially according to a weekly schedule. Thereafter, users could continue visiting MMB2 for 7 additional months.  **Delivery mode:** Individual  **Frequency:** N/S  **Facilitation mode:** Self-guided  **How Integrity was maintained:** N/S | **Time of initiation:** Any (both antepartum & postpartum)  **Duration:** 12 weeks  **Period covered by intervention:** Any (both antenatal and/or postnatal) | TAU | **Depression:** PHQ-9 (minimal clinically important difference (MCID) was used to evaluate the clinical significance of the intervention effects, and this was noted as a baseline to post-test PHQ-9 reduction of at least 5 points) | Mean ± SD  DHI: Baseline = 10.68 ± 4.96 Post-intervention = 5.78 ± 4.42  TAU Baseline = 10.24 ± 5.57 Post-intervention = 7.48 ± 5.67 |
| DeMairo 2023 [102] | **Aim:** To compare outcomes obtained for women admitted to the original "The Motherhood Center of New York partial hospitalization program", who were discharged prior to March 16, 2020, with those obtained for women who were admitted on or after that date and completed the program virtually.  **Country:** United States (HIC)  **Study Type:** Retrospective cohort | SR only | **Age:** DHI = 35.8 ± 4.5 Control = 35.0 ± 4.1  **Sample size:** DHI = 60 Control = 81  **Pregnancy status:** Pregnant & postpartum | Inclusion:   Exclusion: | **Channel:** Video conferencing (Zoom)  **Component:** (a) Professional support (b) Health education (c) Psychotherapy, including mindfulness | **Purpose:** Perinatal support  **Guiding theory:** N/S | **Delivery:** Combination of therapeutic support, medication management, interpersonal process and education groups, along with alternative interventions including yoga, mindfulness and meditation, and art therapy. Patients are followed by an individual therapist and a reproductive psychiatrist and nursery care and mother-infant dyadic psychotherapy are also available. Intensive medical interventions were not available, as they would require an inpatient level of monitoring.  **Delivery mode:** Hybrid: individual and group  **Frequency:** N/S  **Facilitation mode:** Therapist-facilitated  **How Integrity was maintained:** N/S | **Time of initiation:** Any (both antepartum & postpartum)  **Duration:** N/S  **Period covered by intervention:** Any (both antenatal and/or postnatal) | On-site delivery of same intervention component | **Depression:** EPDS (possible score range: 0-30, cut-off score = ≥12.5) | Mean ± SD  DHI: Baseline = 18.90 ± 5.63 Post-intervention = 6.86 ± 3.47  TAU Baseline = 17.43 ± 5.37 Post-intervention = 8.57 ± 4.63 |
| Dennis 2020 [72] | **Aim:** To examine the effectiveness of nurse-delivered telephone interpersonal psychotherapy (IPT) for postpartum depression.  **Country:** Canada (HIC)  **Study Type:** RCT | SR & MA | **Age:** Range and mean not specified  **Sample size:** Total randomised = 241 DHI (enrolled) = 120 Control (enrolled) = 121 DHI (received intervention and completed baseline) = 113 Control (completed baseline) = 121 DHI (completed) = 104 Control (completed) = 100  **Pregnancy status:** Postpartum | **Inclusion:** (a) English-speaking (b) Clinically depressed as per a SCID-I interview (c) >18 years old (d) Between 2 and 24 weeks postpartum, and (e) Discharged home from hospital with their infant.  **Exclusion:** (a) Current antidepressant or antipsychotic medication (b) Receiving psychotherapy from a trained professional (c) Active suicidal, self-harm or infanticidal thoughts (d) Psychosis, and (e) Chronic depression (episode length >2 years). | **Channel:** Telephone (calls)  **Component:** IPT | **Purpose:** Treatment delivery option  **Guiding Theory:** N/S | **Delivery:** In addition to TAU, the women received 12 IPT sessions delivered by a trained nurse, with the first contact to initiate treatment occurring within 72 h of trial enrolment. Treatment occurred in three phases. The first phases included an establishment of therapeutic alliance, provision of psychoeducation about depression and IPT, placed the depression in an interpersonal context, reviewed the mother's current and past interpersonal relationships, identified the interpersonal problem area(s) most related to the current depressive episode and set treatment goals. During the middle phase, treatment focused on resolving interpersonal difficulties, such as conflicts with a partner or extended family (interpersonal disputes) and changes in social roles associated with the challenges of new parenthood and needed support (role transitions). The concluding phase reinforced the mother's efforts, gains and competence in working through the interpersonal problems and overcoming depression, with contingency planning in the event of depression recurrence. Each session was about 60 minutes long.  **Delivery mode:** Individual  **Frequency:** 1 session per week  **Facilitation mode:** Therapist-facilitated  **How Integrity was maintained:** The facilitating nurses were continuously monitored via a checklist and they used treatment-tracking forms with IPT phase- and focus-specific practice activity logs. Telephone-IPT sessions were digitally audio recorded to guide supervision and ensure adherence and in-person or teleconferenced group supervision occurred weekly. A subset of 25% of randomly selected IPT session recordings from each of the three treatment phases and nurses was reviewed by an independent IPT-trained rater for treatment fidelity using an IPT adherence checklist; the rater was trained to achieve over 90% agreement with an IPT expert trainer on the adherence scale used for the trial. | **Time of initiation:** Postpartum  **Duration:** 12 weeks  **Period covered by intervention:** Postnatal only | TAU | **Depression:** SCID-I major depression module.  EPDS (possible range: 0-30, cut-off score = >12) | SCID-1 (reported as proportions) [n(%)] DHI: Baseline = 120 (100) Post-intervention (12 weeks) = 11 (10.5) 24 weeks follow-up= 11 (10.9) 36 weeks follow-up = 11 (10.9)  TAU Baseline = 121 (100) Post-intervention (12 weeks) = 35 (35) 24 weeks follow-up= 34 (33.7) 36 weeks follow-up = 15 (14.9)  .................................  EPDS (reported as Mean ± SD) DHI: Baseline = 17.57 ± 2.85 Post-intervention (12 weeks) = 7.27 ± 5.14 24 weeks follow-up = 6.54 ± 4.89 36 weeks follow-up = 6.79 ± 5.40  Control: Baseline = 17.47 ± 2.93 Post-intervention (12 weeks) = 12.40 ± 4.36 24 weeks follow-up = 11.79 ± 4.74 36 weeks follow-up = 9.77 ± 4.69  p<0.001 |
| Gammer 2020 [73] | **Aim:** To evaluate Kindness for Mums Online (KFMO) for maternal psychological well-being in the first year postpartum.  **Country:** United Kingdom (HIC)  **Study Type:** RCT | SR & MA | **Age:** 35.19 ± 4.04  **Sample size:** Total randomised = 209 DHI (allocated) = 105 Control (allocated) = 101 DHI (started intervention) = 57 DHI (completed) = 54 Control (completed) = 80  **Pregnancy status:** Postpartum | **Inclusion:** (a) Aged 18 years or over (b) Identified as the mother (biological, adoptive, or full-time foster carer) of a child aged under 1 year at baseline (c) Lived in the UK, and  (d) Comfortable reading in English.  **Exclusion:** (a) Reported thoughts about self-harm or suicide in the 2 weeks preceding enrolment. | **Channel:** Web-based  **Component:** Mindfulness and self-compassion | **Purpose:** Prevent PPD and improve maternal psychological wellbeing  **Guiding theory:** N/S | **Delivery:** In addition to TAU, mothers in the experimental group were given access to the web-based self-help tool. The content was tailored to mothers by applying examples and techniques to common tasks, experiences, and difficulties that they would likely encounter. Illustrative quotes from other mothers formed a significant part of the content. Exercises were designed to be brief and fit around parenting tasks and activities. The program was designed to be followed over 5 to 6 weeks, with one session becoming available for each of the first 5 weeks. The time requirement was estimated at 10–15 min per week for reading the content, plus a few minutes each day to try an exercise.  **Delivery mode:** Individual  **Frequency:** 1 session per week  **Facilitation mode:** Self-guided  **How Integrity was maintained:** N/S | **Time of initiation:** Postpartum  **Duration:** 5-6 weeks  **Period covered by intervention:** Postnatal only | TAU | **Depression:** DASS-21-depression (possible score range: 0-21; higher score indicate higher symptoms). | Mean ± SD  DHI: Baseline = 4.77 ± 4.66 Post-intervention (6 weeks) = 3.11 ± 2.81 12 weeks follow-up = 2.84 ± 3.05  Control: Baseline = 4.55 ± 3.46 Post-intervention (6 weeks) = 4.09 ± 3.70 12 weeks follow-up = 3.49 ± 3.67 |
| Gemmill 2022 [103] | **Aim:** To evaluate the new Mum2BMoodBooster program in a feasibility trial.  **Country:** Australia (HIC)  **Study Type:** Pre-post single group test | SR only | **Age:** 28-38  **Sample size:** Baseline = 27 Completed = N/S  **Pregnancy status:** Pregnant (between 6 and 35 weeks) | **Inclusion:** (a) Pregnant and  (b) EPDS score of 11–22 (inclusive).  **Exclusion:** (a) Living outside Australia (b) < 18 years old (c) Current diagnosis of substance abuse, bipolar disorder, post-traumatic stress disorder (PTSD) or depression with psychotic features meeting DSM-IV criteria (d) Moderate to high risk of suicide (e) Current depression treatment (psychotherapy or antidepressant treatment). | **Channel:** Multi-options: Online (web-based) & mobile application  **Component:** (a) CBT (b) Educational resources on depression, lifestyle and pregnancy (c) Partner support website | **Purpose:** Treatment delivery option **Guiding Theory:** N/S | **Delivery:** The postnatal MumMoodBooster program comprises six interactive sessions that are sequentially accessed weekly, designed to parallel face-to-face treatment. Each session begins with a video introducing content and includes text, animations, videos, tutorials, and case vignettes relevant to life in the antepartum period. Personalised content such as lists of pleasant activities and goals were created within the program. Self-monitoring tools, such as daily mood and activity tracking, were depicted in charts. Homework activities are to be completed between sessions.  Users have access to library articles on topics including depression, lifestyle and pregnancy, and a partner support website that mothers can invite their partner to access. Low intensity (< 30 min) telephone coaching once a week encourages participants to use the program.  **Delivery mode:** Individual  **Frequency:** 1 session and 1 telephone coaching per week  **Facilitation mode:** Hybrid: Predominantly self-guided and therapist-assisted  **How Integrity was maintained:** N/S | **Time of initiation:** Antepartum  **Duration:** 8 weeks  **Period covered by intervention:** Antenatal only | N/A | **Depression:** DASS-21-depression (Normal 0–9, Mild 10–13, Moderate 14–20, Severe 21–27, Extremely Severe 28 +) | Mean ± SD  DHI: Baseline = 15.05 ± 6.05 Post-intervention (8 weeks) = 7.38 ± 6.99 |
| Goetz 2020 [104] | **Aim:** To explore the clinical effectiveness of a 1-week electronic course of mindfulness on prenatal depression and anxiety in hospitalized, high-risk pregnant women.  **Country:** Germany (HIC)  **Study Type:** Pre-post single group test | SR only | **Age:** 22-41  **Sample size:** Baseline = 68 Completed = 39  **Pregnancy status:** Pregnant | **Inclusion:** (a) Age of 18 years or older (b) Fluency in the German language (c) A gestational age of ≥24 and ≤34 weeks, and (d) Ability to access to the internet.  **Exclusion:** (a) Multiple pregnancy | **Channel:** Mobile application **Component:** (a) Mindfulness (b) Psychoeducation | **Purpose:** Treatment delivery option **Guiding Theory:** N/S | **Delivery:** The app contains instructional videos and audio files, interactive worksheets, and a personal “skills box” to collect exercises, videos, and texts. It comprised three 45-min modules on mindfulness. After the 1-week course, participants had the opportunity to continue accessing the exercises. All the participants received scientifically validated information about pregnancy and birth via a pregnancy counsellor. The main topics were the physical changes in pregnancy, the birth process, pain relief during birth, bonding between parents and child, breastfeeding, and tips on the formalities related to birth.  **Delivery mode:** Individual  **Frequency:** 1 module every 2 days  **Facilitation mode:** Hybrid: Predominantly self-guided and therapist-assisted  **How Integrity was maintained:** N/S | **Time of initiation:** Antepartum  **Duration:** 1 week  **Period covered by intervention:** Antenatal only | N/A | **Depression:** EPDS (range: 0-30, cut-off score = >9) | Mean ± SD  DHI: Baseline = 8.41 ± 4.77 Post-intervention = 8.62 ± 4.13 |
| Gong 2021 [105] | **Aim:** To explore the effectiveness and feasibility of a comprehensive intervention based on using a short message service (SMS) to reduce depressive symptoms and prevent depression during pregnancy.  **Country:** China (UMIC)  **Study Type:** Quasi-experimental; double arm | SR only | **Age:** DHI = 28.7 ± 4.3 Control = 29.0 ± 4.7  **Sample size:** Total randomised = 4,501 DHI (allocated) = 1,739 Control (allocated) = 2,762 DHI (completed) = 1,481 Control (completed) = 1,855  **Pregnancy status:** Pregnant | **Inclusion:** (a) First birth examination was at less than 14 weeks of gestation (b) Had regular expression and understanding ability (c) Had no history or current symptoms of severe or life-threatening mental illness, and  (d) Not participating in psychotherapy at recruitment.  **Exclusion:** (a) Termination of pregnancy for various reasons (b) No medical follow-up during the entire pregnancy (c) Requirement of immediate medical or psychiatric inpatient care. | **Channel:** Telephone (SMS)  **Component:** (a) Health education (b) Behavioural intervention (c) Antenatal schedule reminders | **Purpose:** Prevention and treatment delivery option  **Guiding theory:** N/S | **Delivery:** There were two types of SMS content, one for health education (alongside behavioural intervention) and another for reminders. A one-way message with health education information about how to reduce the risk of depression during pregnancy was sent via SMS every Monday from 9:00 am to 10:00 am. Other interventions included reminding pregnant women of their check-up time (sent on the day before the scheduled check-up time at 9:00 am to 10:00 am), providing maternity school courses (courses included pregnancy psychology, pregnancy exercise guidance, pregnancy diet and weight control guidance. Each course is 1 hour and runs every Saturday, a complete maternity course takes 4 weeks. All the pregnant women in the intervention group were required to participate.  For participants who were screened positive for depression at baseline, the health manager scheduled an appointment with the psychiatrist for a one-on-one consultation and text appointment details to the patient.  **Delivery mode:** Individual  **Frequency:** N/S  **Facilitation mode:** Therapist-facilitated  **How Integrity was maintained:** N/S | **Time of initiation:** Antepartum  **Duration:** N/S  **Period covered by intervention:** Antenatal only | TAU | **Depression:** EPDS (range: 0-30, cut-off score = >9) | Mean ± SD  DHI: Baseline = 6.2 ± 4.3 Post-intervention = 3.9 ± 3.9  Control: Baseline = 6.0 ± 4.4 Post-intervention = 5.2 ± 4.3 |
| Haga 2019 [74] | **Aim:** To assess the effectiveness of an automated internet intervention (‘Mamma Mia’) on perinatal depressive symptoms.  **Country:** Norway (HIC)  **Study Type:** RCT | SR & MA | **Age:** DHI = 31.0 ± 4.6 Control = 31.1 ± 4.5  **Sample size:** Total randomised = 1,342 DHI (allocated) = 678 Control (allocated) = 664 DHI (completed) = 381 Control (completed) = 466  **Pregnancy status:** Pregnant | **Inclusion:** (a) Pregnant (up until 25 weeks) (b) At least 18 years  (c) Able to read and write Norwegian (d) Have access to the internet  (e) Have an electronic mailing account.  **Exclusion:** N/S | **Channel:** Online (web-based & email)  **Component:** Mindfulness | **Purpose:** Prevention and treatment delivery option  **Guiding theory:** N/S | **Delivery:** The intervention comprises three phases. The first phase consists of 11 sessions beginning in the second trimester in gw 21–25 and ends in gw37. The second phase starts when the infant is 2–3-weeks-old, and lasts for 6 weeks, with three sessions per week. The final phase consists of 10 sessions over an 18-week period. In total, the intervention consists of 44 sessions over a period of 11.5 months.  All sessions include themes specific to the perinatal period. The intervention combines text, pictures, prerecorded audio files, and user input. Each session is designed to take about 10 min and must be completed before users can access the next session. This is done to ensure that relevant information has been reviewed and to create continuity and a narrative in the program  **Delivery mode:** Individual  **Frequency:** N/S  **Facilitation mode:** Self-guided  **How Integrity was maintained:** N/S | **Time of initiation:** Antepartum  **Duration:** 11.5 months  **Period covered by intervention:** Antenatal & postnatal | TAU | **Depression:** EPDS (range: 0-30, cut-off score = ≥10) | Mean ± SD  DHI: Baseline = 6.5 ± 4.5 Post-intervention = 4.0 ± 4.0  Control: Baseline = 6.2 ± 4.4 Post-intervention = 4.4 ± 4.3 |
| Hassdenteufel 2023 [75] | **Aim:** To investigate the clinical effectiveness of an eMBI in a sample of pregnant women who screened positive for emotional distress.  **Country:** Germany (HIC)  **Study Type:** RCT | SR & MA | **Age:** 32.6 ± 4.3  **Sample size:** Total randomised = 460 DHI (allocated) = 230 Control (allocated) = 230 DHI (completed) = 142 Control (completed) = 174  **Pregnancy status:** Pregnant | **Inclusion:** (a) Age 18 years or older (b) Adequate proficiency in German (c) Singleton pregnancy (d) No known neonatal malformations, anomalies or underlying risk factors for preterm birth (d) <29 weeks of gestation at screening (e) Health insurance coverage by one of the participating statutory health insurance providers, and (f) Residency within the state of Baden-Württemberg.  **Exclusion:** (a) Expecting multiples and have an otherwise increased risk of preterm delivery (b) Acute psychotic episodes or diagnosed schizophrenic disorders, suicidality, substance abuse disorders, borderline personality disorder, bipolar disorders, traumatic experiences without reference to the current pregnancy, or the need for an acute psychiatric treatment. (c) Not have participated in any mindfulness-based intervention during the current pregnancy. | **Channel:** Mobile application  **Component:** Mindfulness | **Purpose:** Treatment delivery option  **Guiding theory:** N/S | **Delivery:** The intervention consisted of eight weekly sessions lasting 45 min involving psychoeducational and obstetrical content, mindfulness exercises, and cognitive behavioural approaches.  Mediated skills comprised how to exit from the vicious circle of fear and the use of mindful breathing and mindful body scans.  Content was delivered in the form of audio files, videos, written content, a personal skills box, and interactive worksheets.  **Delivery mode:** Individual  **Frequency:** 1 session per week  **Facilitation mode:** Self-guided  **How Integrity was maintained:** N/S | **Time of initiation:** Antepartum  **Duration:** 8 weeks  **Period covered by intervention:** Antenatal and postnatal | TAU | **Depression:** EPDS (range: 0-30, cut-off score = >9) | Mean  DHI: Baseline = 11.8 Post-intervention = 9.3  Control: Baseline = 11.7 Post-intervention = 10.8 |
| Heller 2020 [76] | **Aim:** To examine the effectiveness of a guided internet intervention (MamaKits online) for pregnant women with moderate to severe symptoms of anxiety or depression.  **Country:** Netherlands (HIC)  **Study Type:** RCT | SR & MA | **Age:** DHI = 32.08 ± 4.61 Control = 31.94 ± 4.83  **Sample size:** Total randomised = 159 DHI (allocated) = 79 Control (allocated) = 80 DHI (completed) = 54 Control (completed) = 65  **Pregnancy status:** Pregnant | **Inclusion:** (a) Aged 18 years and older (b) Pregnant for less than 30 weeks (c) Showed symptoms of depression or anxiety or both (d) Had sufficient access to the internet.  **Exclusion:** (a) Reported intentions to harm themselves or to attempt suicide. | **Channel:** Online (web-based)  **Component:** PST | **Purpose:** Treatment delivery option  **Guiding theory:** N/S | **Delivery:** The course consists of 5 modules comprising educating information, examples of other pregnant women with depressive or anxiety symptoms, and homework assignments.  The intervention consists of three steps: (1) participants describe what really matters to them (2) participants write down their current worries and problems and categorize them into three types: (a) unimportant problems (problems unrelated to the things that matter to them), (b) problems which can be solved, and (c) problems which cannot be solved (e.g. the loss of a loved one), (3) participants make a plan for the future in which they describe how they will try to accomplish those things that matter most to them. The core of the intervention consists of a structured approach to solve the potentially solvable problems. This approach consists of 6 steps: (1) write down a clear definition of the problem, (2) generate multiple solutions to the problem, (3) select the best solution, (4) work out a systematic plan for this solution, (5) carry out the solution, and (6) evaluate whether the solution has resolved the problem. After each module, trained coaches (students pursuing Master’s in Psychology) provided feedback on the assignments via secured email. On average, the coaches gave 20 min of feedback per patient per module. The feedback was directed to helping the patient work through the intervention; the coaches answered questions if something was not clear and provided feedback on homework assignments. If a participant was delayed in submitting the homework, the coach sent a reminder by email, with a maximum of three emails and one phone call after that.  **Delivery mode:** Individual  **Frequency:** One module per week  **Facilitation mode:** Hybrid (self-guided and therapist-assisted)  **How Integrity was maintained:** All coaches were trained for 4 hours in PST and providing feedback via secured email. They were trained by an experienced psychotherapist, who also provided the coaches with regular supervision. | **Time of initiation:** Antepartum  **Duration:** 5 weeks  **Period covered by intervention:** Antenatal only | TAU | **Depression:** CES-D (Range: 0-60; the higher the value, the higher the symptom, cut-off score = ≥16)  EPDS (Range: 0-30) | Mean ± SD  CES-D DHI: Baseline = 28.8 ± 7.5 Post-intervention = 19.5 ± 10.2  Control: Baseline = 27.9 ± 9.0 Post-intervention = 18.6 ± 9.4   EPDS DHI: Baseline = 14.3 ± 4.9 Post-intervention = 9.5 ± 5.6  Control: Baseline = 14.0 ± 4.9 Post-intervention = 8.9 ± 5.5 |
| Huang 2021 [77] | **Aim:** To assess the effects of internet-based support program for Chinese first-time mothers during the early stage of motherhood, in regarding of the improvements of maternal self-efficacy, and social support; and the alleviation of postpartum depression symptoms.  **Country:** China (UMIC)  **Study Type:** RCT | SR & MA | **Age:** 27.25 ± 3.04  **Sample size:** Total randomised = 40 DHI (allocated) = 20 Control (allocated) = 20 DHI (completed) = 18 Control (completed) = 18  **Pregnancy status:** Postpartum | **Inclusion:** (a) 18 years old or above (b) Being first-time mothers with healthy babies (c) Ability to response the questionnaires, and  (d) Having internet connection by mobile phone or computer.  **Exclusion:** (a) Mother or their infants had serious diseases | **Channel:** Online (web-based)  **Component:** (a) Educational resources (b) Professional support | **Purpose:** Postpartum support **Guiding theory:** N/S | **Delivery:** In addition to the routine care, women in the intervention group had access to the website which has five components including learning forum with educational resources, communication forum for social function, ask-the-expert forum for professional support, baby home forum for recording baby's milestones, and the reminder function.  **Delivery mode:** Individual  **Frequency:** Reminder telephones per week, reminding them to log in the ISP at least twice a week, and no less than total 1 hr per week  **Facilitation mode:** Self-guided  **How Integrity was maintained:** N/S | **Time of initiation:** Postpartum **Duration:** 12 weeks **Period covered by intervention:** Postnatal only | TAU | **Depression:** EPDS (range: 0-30) | Mean ± SD  DHI: Baseline = 5.05 ± 2.54 Post-intervention = 6.11 ± 2.54  Control: Baseline = 5.25 ± 2.15 Post-intervention = 9.22 ± 3.30 |
| Huh 2023 [78] | **Aim:** To determine if online public health nurses-delivered group CBT for PPD added to TAU can improve the mother-infant relationship, social support, and infant temperament more than TAU alone.  **Country:** Canada (HIC)  **Study Type:** RCT | SR & MA | **Age:** DHI = 31.6 ± 4.8 Control = 31.3 ± 4.9  **Sample size:** Total randomised = 159 DHI (enrolled and completed baseline assessment) = 71 Control (enrolled and completed baseline assessment) = 65 DHI (completed) = 62 Control (completed) = 60  **Pregnancy status:** Postpartum | **Inclusion:** (a) ≥ 18 years old (b) had an infant < 12 months old (c) EPDS score ≥ 10 (d) living in the Niagara region  **Exclusion:** (a) Bipolar, psychotic, substance use, and/or borderline personality disorders | **Channel:** Videoconferencing (Zoom)  **Component:** (a) CBT (b) Psychoeducation | **Purpose:** Treatment delivery option **Guiding Theory:** N/S | **Delivery:** The first half of each session is CBT-focused while the second half is on psychoeducation. Each session lasted 2 hours and was delivered by 2 public health nurses.  **Delivery mode:** Group  **Frequency:** 1 session per week  **Facilitation mode:** Therapist-facilitated  **How Integrity was maintained:** None | **Time of initiation:** Postpartum  **Duration:** 9 weeks  **Period covered by intervention:** Postnatal only | TAU | **Depression:** EPDS (range: 0-30) | Mean  DHI: Baseline = 14.60 Post-intervention = 10.80  Control: Baseline = 14.92 Post-intervention = 12.86 |
| Hulsbosch 2023 [79] | **Aim:** To examine the effectiveness of an online self-guided MBI in pregnant women with pregnancy distress.  **Country:** Netherlands (HIC)   **Study Type:** RCT | SR & MA | **Age:** 22-42 years  **Sample size:** Total randomised = 360 DHI (enrolled and completed baseline assessment) = 109 Control (enrolled and completed baseline assessment) = 112 DHI (completed) = 104 Control (completed) = 95  **Pregnancy status:** Pregnant | **Inclusion:** (a) 18+ years (b) A sufficient understanding of the Dutch language (c) Had their antenatal visit before 12 weeks of gestation.  **Exclusion:** (a) Multiple pregnancy (b) Known endocrine disorder before pregnancy (other than thyroid function problems), diabetes type I, rheumatoid arthritis, severe psychiatric disease (schizophrenia, borderline personality disorder, or bipolar disorder), HIV, drug or alcohol addiction problems, or any other disease resulting in treatment with drugs that are potentially adverse for the foetus and need careful follow-up during the pregnancy, and (b) No access to the internet | **Channel:** Online (web-based)  **Component:** Mindfulness | **Purpose:** Treatment delivery option **Guiding Theory:** N/S | **Delivery:** The intervention included eight weekly online one-hour sessions with in between sessions invitation to practice a mindfulness meditation daily (e.g., body scan, 3 min breathing space, routine activity). A certified mindfulness trainer, reviewed questions of participants and sent weekly reminders to positively encourage the participating women to follow the course and to ask for help when they need it. Content include stress and mindfulness, dealing with obstacles, the body and the senses, thoughts, emotions, communication and awareness, take good care of yourself and your baby, and the beginning of a new way of life.  **Delivery mode:** Individual  **Frequency:** 1 session per week  **Facilitation mode:** Hybrid: self-guided and some coach input  **How Integrity was maintained:** N/S | **Time of initiation:** Antepartum  **Duration:** 8 weeks  **Period covered by intervention:** Antenatal only | TAU | **Depression:** EPDS (range: 0-30, cut-off score = ≥8) | Mean ± SD  DHI: Baseline = 9.5 ± 4.2 Post-intervention = 8.6 ± 4.8  Control: Baseline = 9.2 ± 3.7 Post-intervention = 8.1 ± 4.7 |
| Jannati 2020 [80] | **Aim:** To examine the effect of mobile phone applications-based CBT on postpartum depression.  **Country:** Iran (LMIC)  **Study Type:** RCT | SR & MA | **Age:** DHI = 27.65 ± 4.2 Control = 27.39 ± 4.8  **Sample size:** Total randomised = 78 DHI (allocated) = 39 Control (allocated) = 39 DHI (completed) = 38 Control (completed) = 37  **Pregnancy status:** Postpartum | **Inclusion:** (a) Aged 18 or above (b) At least weekly access to the Internet and mobile phone (c) Giving birth in the last six months, and (d) Having sufficient Persian language skills to complete self-administered surveys. (e) A score of 13 or higher on EPDS  **Exclusion:** N/S | **Channel:** Mobile application  **Component:** CBT | **Purpose:** Treatment delivery option **Guiding Theory:** N/S | **Delivery:** The mobile application consisted of eight sequential lessons which read like a storybook, and participants follow the story of women who had the same situation and suffered from PPD, and through their story, learn how they come to manage their symptoms, and these principles can then be applied to the participants' own life.  Each lesson is about 45−60 minutes long.  At the end of each lesson, the assignments associated with that lesson were given to the participants.  The sessions contents include introduction to the program and goal setting, getting activated, emotional recognition, noticing thoughts, thought challenging, problem-solving, improving social skills, and relapse prevention.  **Delivery mode:** Individual  **Frequency:** N/S  **Facilitation mode:** Self-guided  **How Integrity was maintained:** N/S | **Time of initiation:** Postpartum **Duration:** 8 weeks  **Period covered by intervention:** Postnatal only | TAU | **Depression:** EPDS (range: 0-30, cut-off score = ≥13) | Mean ± SD  DHI: Baseline = 17.42 ± 2.8 Post-intervention = 8.18 ± 1.5  Control: Baseline = 17.39 ± 2.2 Post-intervention = 15.05 ± 2.9 |
| Jiao 2019 [81] | **Aim:** To examine the effectiveness of web-based and home-based psychoeducational programs on self-efficacy in newborn care, as well as social support, psychological well-being, and maternal satisfaction with postnatal support.  **Country:** Singapore (HIC)  **Study Type:** RCT | SR & MA | **Age:** DHI = 31.1 ± 3.8 Third-arm (home-based) = 30.5 ± 3.9 Control = 30.3 ± 3.7  **Sample size:** Total randomised = 205 DHI (allocated) = 68 Home-based (allocation) = 68 Control = 68 DHI (completed) = 64 Home-based (completed) = 62 Control (completed) = 64  **Pregnancy status:** Postpartum | **Inclusion:** (a) Had full-term pregnancy (b) 21 years old and older (c) Able to read and speak in English (d) Had Internet access through a computer or a smartphone, and (e) Planned to reside in Singapore for the 6 months post-delivery.  **Exclusion:** (a) Had identified physical or mental disorders before and during pregnancy that would hinder ability to participate in the study (b) Had complicated assisted delivery with 4th degree perineal tear,  (c) Gave birth to a stillborn child or a child with congenital anomalies or medical complications (e.g., pathological jaundice), which required specialized attention in the hospital. | **Channel:** Online (web-based)  **Component:** Psychoeducation | **Purpose:** Postpartum support **Guiding theory:** N/S | **Delivery:** Content included materials on postnatal experiences, maternal self-care, newborn care, and social support. There was a peer discussion forum where the participant could communicate with other participants, or a confidential corner for participants to ask personal questions. Expert advice was also provided when needed. There were also thrice-weekly telephone reminders, which lasted for about 3 min each, solely as a reminder for participants to access the website without any additional education provided.  **Delivery mode:** Hybrid (Individual learning and peer discussions)  **Frequency:** N/A  **Facilitation mode:** Hybrid (Self-guided and therapist-facilitated)  **How Integrity was maintained:** N/S | **Time of initiation:** Postpartum  **Duration:** 1 month  **Period covered by intervention:** Postnatal only | 1. TAU 2. Home-based intervention of the same web-based component | **Depression:** EPDS (range: 0-30) | Mean ± SD  DHI: Baseline = 7.4 ± 4.5 Post-intervention = 4.73 ± 9.94  Home-based: Baseline = 7.7 ± 4.8 Post-intervention = 5.63 ± 9.70  Control: Baseline = 9.0 ± 4.0 Post-intervention = 5.14 ± 10.55 |
| Kubo 2021 [106] | **Aim:** To test the feasibility and acceptability of offering mindfulness-based interventions for pregnant women with moderate-to-moderately-severe depression symptoms.  **Country:** United States (HIC)  **Study Type:** Pre-post single group test | SR only | **Age:** 19-39  **Sample size:** Enrolled = 27 Completed = 20  **Pregnancy status:** Pregnant | **Inclusion:** (a) age ≥18 years (b) Patient Health Questionnare-9 (PHQ-9) score 10–19 (indicating moderate-to-moderately-severe depression symptoms) (c) <28 weeks of gestation (d) English-speaking, and (e) access to a smartphone, tablet, and/or computer with Internet connection.  **Exclusion:** (a) Engaged in a regular mindfulness or meditation practice three or more times per week  (b) Enrolled in a mindfulness program. | **Channel:** Mobile application or website  **Component:** Mindfulness | **Purpose:** Treatment delivery option **Guiding Theory:** N/S | **Delivery:** The intervention used a commercially available mindfulness program, Headspace™. Each participant was given a study-specific log-in ID and instructed to first complete the 30-day “Basics” course after which they could choose other 10- to 30-day courses that are condition- or situation-specific, such as “Pregnancy,” “Anxiety,” “Relationships,” or “Sleep,” or single meditation sessions for the remainder of the 6-week study period.   **Delivery mode:** Individual  **Frequency:** 10-20 mins per day  **Facilitation mode:** Self-guided  **How Integrity was maintained:** N/S | **Time of initiation:** Antepartum  **Duration:** 6 weeks  **Period covered by intervention:** Antenatal only | N/A | **Depression:** PHQ-8 (possible range: 0-24; 1-4 = minimal depression; 5-9 = mild depression; 10-14 = moderate depression; 15-19 = moderately severe depression, and 20-24 = severe depression) | Baseline = 13.5 ± 3.5 Post-intervention = 7.5 ± 4.4 |
| Latendresse 2023 [107] | **Aim:** To explore the feasibility, acceptability, and preliminary assessment of effectiveness of a group telehealth intervention for PD in rural women.  **Country:** United States (HIC)  **Study Type:** Pre-post single group test | SR only | **Age:** Median = 26  **Sample size:** Baseline = 28 Completed = 22  **Pregnancy status:** Pregnant and Postpartum | **Inclusion:** (a) English-speaking (b) EPDS scores 9–20 (corresponding to mild to moderate symptoms of depression) (c) 18 years of age or older  **Exclusion:** (a) EPDS scores < 9 (no depressive symptoms) or > 20 (likely severe depression) (b) self-reporting other severe mental illnesses (schizophrenia, bipolar mood disorder)  (c) already taking a psychotropic medication | **Channel:** Videoconferencing  **Component:** MBCT | **Purpose:** Treatment delivery option **Guiding Theory:** N/S | **Delivery:** Women joined a live videoconference group of 4–6 women for 1-hr weekly sessions for 8 weeks, facilitated by a licensed psychiatric-mental health nurse practitioner and trained in MBCT principles and techniques. Each session included presentation of MBCT content, facilitator-led engagement in MBCT activities, and participant-directed discussion of their experiences in applying MBCT practices. Prior to joining the sessions, participants met with study personnel on two occasions: (a) to discuss technology literacy, training, privacy protection issues, and to demonstrate how to access the videoconference platform, and (b) further assessment by an MHP to determine appropriateness for the study (no evidence of severe depression or other serious or persistent mental illness) and referral to mental health resources, if needed.  **Delivery mode:** Group  **Frequency:** 1 session per week  **Facilitation mode:** Therapist-facilitated  **How Integrity was maintained:** N/S | **Time of initiation:** Any (both antepartum & postpartum)  **Duration:** 8 weeks  **Period covered by intervention:** Any (antenatal or postnatal) | N/A | **Depression:** EPDS (range: 0-30; < 9 indicate little to no depressive symptoms; 9–13 indicates mild depressive symptoms; 14–19 suggests moderate symptoms; and ≥20 indicates a high probability of a severe depressive disorder) | Baseline = 14.0 ± 4.8 Post-intervention = 9.5 ± 5.0 |
| Lee 2021 [108] | **Aim:** To develop a video communication-based group CBT program for pregnant women with depressed mood, investigate its effectiveness, and identify the correlations between automatic thoughts, dysfunctional attitudes, and depression in depressed pregnant women.  **Country:** Korea (HIC)  **Study Type:** Quasi-experimental; single arm | SR only | **Age:** 30-37 years  **Sample size:** Enrolled = 13 Completed = 13  **Pregnancy status:** Pregnant | **Inclusion:** (a) 20 years of age or older (b) Between 14 and 32 weeks of pregnancy (c) A PPD test (Edinburgh Postnatal Depression Scale, EPDS) score of 9 or higher (d) Ability to understand and respond to the questionnaire (e) Owning computers or smartphones capable of video communication, and  (f) voluntary participation in this study  **Exclusion:** (a) Diagnosed with a mental illness and taking drugs or receiving psychiatric treatment (b) Having serious pregnancy complications (c) non-Korean citizenship, and (d) illiteracy. | **Channel:** Videoconferencing  **Component:** CBT | **Purpose:** Treatment delivery option **Guiding Theory:** N/S | **Delivery:** Participants were divided into four experimental groups by assigning three to four participants to each group on a first-come, first-served basis. Each group participated in eight sessions of the CBT program for 80 minutes (with CBT content consisting of 50 minutes and 30 minutes for introduction, overview, and checking on assignments). A research assistant contacted the participants in advance before the start of each session to check whether they were connected for video communication and to help them connect smoothly. Each session was conducted in the following sequence: sharing life experiences after the last session, sharing the main learning content of each session and feelings about the activities, and instructions on the assignment. At the end of the session, the assignment was explained and participants were asked to send their finished assignment before the next session via email or social media. The submitted assignments were shared with other members of the group during group activities in the next session. Assuming that self-disclosure might be difficult due to prejudice against people with depression, the participants were asked to select an alias during the first self-introduction session, and it was used until the end of the program.  **Delivery mode:** Group  **Frequency:** 2 sessions per week  **Facilitation mode:** Therapist-facilitated  **How Integrity was maintained:** N/S | **Time of initiation:** Antepartum  **Duration:** 4 weeks  **Period covered by intervention:** Antenatal only | N/A | **Depression:** EPDS (range: 0-30) | Baseline = 14.38 ± 5.10 Post-intervention = 6.30 ± 3.79 |
| Loughnan, Butler 2019 [82] | **Aim:** To evaluate the efficacy and acceptability of the three-lesson unguided MUMentum postnatal program in postpartum women with elevated symptoms of depression and/or generalized anxiety compared to TAU  **Country:** Australia (HIC)  **Study Type:** RCT | SR & MA | **Age:** 32.56 ± 4.53  **Sample size:** Total randomised = 131 DHI (enrolled and completed baseline assessment) = 65 Control (enrolled and completed baseline assessment) = 55 DHI (completed) = 50 Control (completed) = 47  **Pregnancy status:** Postpartum | **Inclusion:** (a) within 12 months postpartum (b) aged over 18 years (c) fluent in written and spoken English (d) Australian resident (e) computer and internet access (f) self-report symptoms of anxiety and/or depression above clinical threshold (e.g., GAD-7 and/or PHQ-9 total score ≥ 10) (g) and willing to provide personal contact details and details of their general practitioner (GP).  **Exclusion:** (a) current substance abuse or dependence (b) current use of benzodiazepines (c) self-reported diagnosis of schizophrenia or bipolar disorder (d) started psychological therapy <4 weeks ago or medication <8 weeks ago for anxiety/depression. (e) Severe depression (PHQ-9 total score ≥ 23) or current suicidality at screening | **Channel:** Online (web-based)  **Component:** (a) CBT (b) Psychoeducation | **Purpose:** Treatment delivery option **Guiding Theory:** N/S | **Delivery:** The program was delivered in an illustrated comic-style story, with two fictional women experiencing postpartum anxiety and depression symptoms. Participants followed the characters' experiences of learning how to self-manage their symptoms during the postpartum period using CBT skills. Each lesson consisted of a set of lesson slides showing the characters’ stories and describing specific CBT skills; a lesson summary and action plan to revise and implement skills (i.e. homework); and a range of additional postpartum-relevant resources. All lessons were accessed sequentially with an automated 5-day lockout period between lessons. Assistance was only available for technical issues.  **Delivery mode:** Individual  **Frequency:** 1 lesson every one-to-two weeks  **Facilitation mode:** Self-guided  **How Integrity was maintained:** N/S | **Time of initiation:** Postpartum **Duration:** 6 weeks **Period covered by intervention:** Postnatal only | TAU | **Depression:** PHQ-9 (possible range: 0-27; cut-off score ≥ 9)  EPDS (range: 0-30, cut-off score = ≥12) | Mean ± SD  PHQ-9 DHI: Baseline = 11.81 ± 3.22 Post-intervention = 6.11 ± 4.34  Control: Baseline = 12.26 ± 3.11 Post-intervention = 10.44 ± 4.35   EPDS DHI: Baseline = 14.91 ± 3.15 Post-intervention = 8.82 ± 4.96  Control: Baseline = 15.04 ± 3.04 Post-intervention = 13.34 ± 4.96 |
| Loughnan, Sie 2019 [83] | **Aim:** To test the efficacy and acceptability of the MUMentum Pregnancy program compared to TAU control group in reducing symptoms of anxiety, depression, and general psychological distress.  **Country:** Australia (HIC)  **Study Type:** RCT | SR & MA | **Age:** 31.61 ± 4.0  **Sample size:** Total randomised = 87 DHI (enrolled and completed baseline assessment) = 36 Control (enrolled and completed baseline assessment) = 41 DHI (completed) = 23 Control (completed) = 36  **Pregnancy status:** Pregnant | **Inclusion:** (a) aged over 18 years (b) fluent in written and spoken English (c) Australian resident (d) had computer and internet access (e) met criteria for a probable diagnosis of GAD and/or MDD  (f) willing to provide their name, telephone number, address, email address, and the name and contact details of their general practitioner, and (g) between 13 and 30 weeks pregnant.  **Exclusion:** (a) current substance abuse or dependence (b) current use of benzodiazepines (c) diagnosis of schizophrenia or bipolar disorder (d) they had commenced psychological therapy less than four weeks before intake assessment or had commenced medication for anxiety/depression less than eight weeks before intake assessment. (e) Applicants reporting severe depression or current suicidality | **Channel:** Online (web-based)  **Component:** (a) CBT (b) Psychoeducation | **Purpose:** Treatment delivery option **Guiding Theory:** N/S | **Delivery:** The program was delivered in an illustrated comic-style story, with two fictional women experiencing postpartum anxiety and depression symptoms. Participants followed the characters' experiences of learning how to self-manage their symptoms during the postpartum period using CBT skills. Each lesson consisted of a set of lesson slides showing the characters’ stories and describing specific CBT skills; a lesson summary and action plan to revise and implement skills (i.e. homework); and a range of additional postpartum-relevant resources. All lessons were accessed sequentially with an automated 7-day lockout period between lessons. Assistance was only available for technical issues.  **Delivery mode:** Individual  **Frequency:** 1 lesson per week  **Facilitation mode:** Self-guided  **How Integrity was maintained:** N/S | **Time of initiation:** Antepartum **Duration:** 4 weeks **Period covered by intervention:** Antenatal only | TAU | **Depression:** PHQ-9 (possible range: 0-27; cut-off score ≥ 9)  EPDS (range: 0-30, cut-off score = ≥13) | Mean ± SD  PHQ-9 DHI: Baseline = 11.69 ± 4.56 Post-intervention = 11.05 ± 4.48  Control: Baseline = 11.05 ± 4.48 Post-intervention = 8.99 ± 4.56   EPDS DHI: Baseline = 13.41 ± 4.31 Post-intervention = 10.01 ± 4.64  Control: Baseline = 14.50 ± 4.23 Post-intervention = 10.97 ± 4.78 |
| Merza 2023 [84] | **Aim:** To determine if a synchronous online 9-week group CBT delivered by mothers who have recovered from postpartum depression could effectively improve PPD and its comorbidities.  **Country:** Canada  **Study Type:** RCT | SR & MA | **Age:** DHI = 31.7 ± 4.7 Control = 31.6 ± 4.9  **Sample size:** Total randomised = 183 DHI (enrolled and completed baseline assessment) = 77 Control (enrolled and completed baseline assessment) = 67 DHI (completed) = 71 Control (completed) = 66  **Pregnancy status:** Postpartum | **Inclusion:** (a) Identified as mothers or birthing parents (b) Were ≥18 years old (c) Had an infant <12 months at recruitment (d) Fluent in written and spoken English (e) Lived in Ontario, Canada, and (f) Had an Edinburgh Postnatal Depression Scale (EPDS) score of ≥10  **Exclusion:** (a) Had bipolar, psychotic, current substance misuse disorders or borderline personality disorder | **Channel:** Videoconferencing  **Component:** CBT | **Purpose:** Treatment delivery option **Guiding Theory:** N/S | **Delivery:** Participants completed one 2-h session each week, which were led by two randomly selected peer facilitators via ZOOM®. The first half of each session involved teaching and practice of core CBT skills, followed by 1-h of unstructured discussion on topics relevant to those with PPD. Core cognitive skills (e.g., identifying depressogenic thoughts, completing thought records, engaging in cognitive restructuring) were introduced from week one. Behavioural techniques (i.e., behavioural activation, relaxation techniques, problem solving, and goal setting) were introduced in week two and continued throughout the program. The research coordinator oversaw and recorded the sessions. Peer facilitators were recruited through advertisements and community partners and were eligible if they scored below clinical cut-offs on BDI-II (<14) and the GAD-7 (<10) at the time of recruitment. Five peers who had recovered from PPD were selected after completing a written application and telephone interview. They underwent a 3-day training program for individuals with no prior formal psychiatric training and then observed the 9-week intervention delivered by experts in the hospital setting which it was developed. Finally, they delivered the intervention online in pairs and were randomly assigned to lead groups.  **Delivery mode:** Group  **Frequency:** 1 session per week  **Facilitation mode:** Peer facilitated  **How Integrity was maintained:** An experienced perinatal psychiatrist (RJV) listened to session recordings and provided 1-h of supervision to peer facilitators weekly. | **Time of initiation:** Postpartum **Duration:** 9 weeks **Period covered by intervention:** Postnatal only | TAU | **Depression:** EPDS (range: 0-30, cut-off score = ≥10) | Mean ± SD  DHI: Baseline = 16.19 ± 4.71 Post-intervention = 10.20 ± 4.32  Control: Baseline = 15.79 ± 4.51 Post-intervention = 13.96 ± 4.90 |
| Miles 2023 [109] | **Aim:** To evaluate the implementation of antenatal parenting intervention Mellow Bumps in an online format and determine if this can be done safely and without detriment to pregnant women in Turkey.  **Country:** Turkey (UMIC)  **Study Type:** Pre-post single group test | SR only | **Age:** 22-48  **Sample size:** Enrolled = 128 Baseline completed = 101 Completed = 59  **Pregnancy status:** Pregnant | **Inclusion:** (a) in third trimester of pregnancy (b) willingness to participate and (c) able to provide a tablet or computer with internet access.  **Exclusion:** | **Channel:** Videoconferencing  **Component:** Health education | **Purpose:** Pregnancy care and PPD prevention  **Guiding theory:** N/S | **Delivery:** All practitioners completed full training in Online Mellow Bumps as a prerequisite to any group delivery. Groups were delivered online using Zoom and would consist of 2 practitioners and between 6 and 8 mums-to-be. Participants were allocated in the order that they were recruited. Each group would run over 7 weekly sessions and last approximately 90 minutes each. There was an additional eighth ‘Partners Session’ that groups had the option to run.  **Delivery mode:** Group  **Frequency:** 1 session per week  **Facilitation mode:** Therapist-facilitated  **How Integrity was maintained:** Mandatory reflective consultation sessions between group practitioners and senior Mellow Parenting trainers | **Time of initiation:** Antepartum  **Duration:** 7-8 weeks  **Period covered by intervention:** Antenatal only | N/A | **Depression:** DASS-42-depression (Normal 0–9, Mild 10–13, Moderate 14–20, Severe 21–27, Extremely Severe 28+) | Mean ± SD  Baseline = 7.00 ± 5.64 Post-intervention = 5.78 ± 5.09 |
| Milgrom 2021 [85] | **Aim:** To compare the efficacy of a web-based CBT interventions (internet CBT+coach calls) for postnatal depression (MumMoodBooster [MMB]) with face-to-face CBT  **Country:** Australia (HIC)  **Study Type:** RCT | SR & MA | **Age:** DHI = 30.8 ± 4.3 Third-arm (face-fo-face) = 32.2 ± 5.3 TAU = 31.9 ± 4.2  **Sample size:** Total randomised = 116 DHI (allocated) = 39 Face-to-face (allocation) = 39 TAU = 38 DHI (completed) = 32 Face-to-face (completed) = 36 TAU (completed) = 33  **Pregnancy status:** Postpartum | **Inclusion:** (a) EPDS scores of 11-25 (b) aged ≥18 years (c) 6 weeks to 1 year postpartum (d) home internet access (e) familiarity with internet and email, and (f) able and willing to give informed consent that included agreeing to be assigned to any of the 3 experimental conditions.  **Exclusion:** (a) Women deemed to be at risk of suicide  (b) Current substance abuse, manic or hypomanic symptoms or depression with psychotic features, posttraumatic stress disorder, and under current treatment for depression (medication or psychotherapy). | **Channel:** Online (web-based)  **Component:** (a) CBT (b) Psychoeducation | **Purpose:** Treatment delivery option **Guiding Theory:** N/S | **Delivery:** The initial steps of the program provide explicit direction, whereas the latter steps encourage participants to assume increasingly greater responsibility for managing their own plan for change. The intervention uses step-by-step guidance through 6 sessions, with a new session becoming available for use every week. Although the intervention can be accessed and used on smartphones, it was designed and optimized for use on desktop computers, laptops, and tablets and designed to function on popular browsers for both Windows PCs and Mac computers. Its charting function was designed to help participants see the functional relationship between mood and activity levels. A printable summary describes the key content covered in each session and a tailored list of recommended home practice activities. An article in the library covers articles on relaxation, problem solving, and getting support for parenting and an article relevant to the women's partners. In addition, it also enables participants to choose whether to invite their partner to use a free-standing partner support website with a separate user log-in.  Weekly low-intensity telephone coaching support (30 minutes maximum per week) was provided to reinforce participant progress, encourage program use, and introduce the themes of upcoming sessions.  **Delivery mode:** Individual  **Frequency:** 1 session and 1 coaching call per week, with allowance for the rescheduling of up to 3 missed coach calls.  **Facilitation mode:** Hybrid (predominantly self-guided and supported with a weekly coach call)  **How Integrity was maintained:** Using a manualized script and a session-by-session checklist | Time of initiation: Postpartum  **Duration:** 6 weeks **Period covered by intervention:** Postnatal only | 1. TAU 2. Face-to-face intervention of the same web-based component | **Depression:** BDI-II (possible range: 0-63; minimal depression = 0-13, mild depression = 14-19, moderate depression = 20-28, and severe depression = 29-63). | Mean ± SD  DHI: Baseline = 28.10 ± 7.91 Post-intervention = 11.63 ± 8.96  Control: Baseline = 29.97 ± 8.76 Post-intervention = 18.85 ± 10.16  Face-to-face: Baseline = 27.18 ± 9.95 Post-intervention = 21.36 ± 12.15 |
| Monteiro 2020 [86] | **Aim:** To explore the efficacy of Be a Mom in enhancing positive mental health among low-risk postpartum women and to test its acceptability, adherence and pattern of usage.  **Country:** Portugal (HIC)  **Study Type:** RCT | SR & MA | **Age:** DHI = 33 ± 4.04 Control = 33 ± 4.43  **Sample size:** Total randomised = 367 DHI (allocated) = 191 Control (allocated) = 176 DHI (completed) = 104 Control (completed) = 145  **Pregnancy status:** Postpartum | **Inclusion:** (a) being in the early postpartum period (up to 3 months postpartum) (b) being 18 years or older (c) presenting low risk for PPD (having a score lower than 5.5 on the Postpartum Depression Predictors Inventory-Revised (d) having internet access at home (e) being a resident of Portugal, and (f) understanding Portuguese  **Exclusion:** (a) The presence of a serious medical condition (physical or psychiatric) in the mother or in the infant (self-reported) | **Channel:** Online (web-based)  **Component:** CBT | **Purpose:** Treatment delivery option **Guiding Theory:** N/S | **Delivery:** The women had access to the 5 modules (changes and emotional reactions; cognitions; values and social support; couple relationship (presented only presented to women in a relationship); and PPD alert signs and professional help seeking). Each module is approximately 30-45 minutes long and women can interrupt it whenever they need to and resume when they are available. Web link: https://beamom.pt/ Email reminders were sent automatically to the participants if they went 3, 7, and 13 days without accessing the program. Asynchronous communication channels were provided for program-related support only.  **Delivery mode:** Individual  **Frequency:** 1 module per week (or slower, but with a maximum allowance of completion of all 5 modules within 8 weeks)  **Facilitation mode:** Hybrid (predominantly self-guided and supported with a weekly coach call)  **How Integrity was maintained:** N/S | **Time of initiation:** Postpartum  **Duration:** 5-8 weeks  **Period covered by intervention:** Postnatal only | TAU | **Depression:** EPDS (possible range: 0-30, higher scores indicative of higher depressive symptoms) | Mean ± SD  DHI: Baseline = 6.38 ± 0.26 Post-intervention = 5.26 ± 0.33  TAU Baseline = 6.72 ± 0.27 Post-intervention = 6.19 ± 0.29 |
| Naja 2023 [87] | **Aim:** To measure the efficacy of low-intensity psychosocial video-based consultations on antenatal mental health outcomes.  **Country:** Qatar (HIC)  **Study Type:** RCT | SR only | **Age:** 25-43  **Sample size:** Total randomised = 58 DHI (allocated and received intervention) = 26 Control (allocated and received intervention) = 21 DHI (completed) = 22 Control (completed) = 11  **Pregnancy status:** Pregnant | **Inclusion:** (a) pregnant women above 18 years old (b) in their second trimester  (c) accepted video consultation, and (d) verbally consented to participate in the study.   **Exclusion:** (a) diagnosed with psychiatric illness  (b) had follow-up appointments with public mental health services  (c) receiving mood stabilizers or antidepressants. (d) not speaking or understanding English and Arabic | **Channel:** Video consultation (via a computer software - Vsee)  **Component:** Psychosocial education | **Purpose:** Preventing antenatal & postnatal depression  **Guiding theory:** N/S | **Delivery:** Delivered through video consultation as two one-on-one sessions of low-intensity psychosocial intervention with each session lasting up to 45 min and scheduled four weeks apar. A screen-sharing software/application tool VSee was used for the consultation   **Delivery mode:** Individual  **Frequency:** 2 sessions, 4 weeks apart  **Facilitation mode:** Therapist-facilitated  **How Integrity was maintained:** N/S | **Time of initiation:** Antepartum  **Duration:** 4 weeks **Period covered by intervention:** Antenatal only | TAU | **Depression:** EPDS (range: 0-30, cut-off score = ≥13) | Mean ± SD  DHI: Baseline = 8.7 ± 5.3 Post-intervention = 8.3 ± 5.0  TAU Baseline = 11.0 ± 4.8 Post-intervention = 9.4 ± 4.1 |
| Nishi 2022 [88] | **Aim:** To investigate the effectiveness of recently developed internet-delivered CBT for preventing the onset of a major depressive episode (MDE) in the third trimester and at 3 months postpartum.  **Country:** Japan (HIC)  **Study Type:** RCT | SR & MA | **Age:** 30.44 ± 4.6  **Sample size:** Total randomised = 5,017 DHI (allocated) = 2,509 Control (allocated) = 2,508 DHI (completed) = 1,705 Control (completed) = 1,804  **Pregnancy status:** Pregnant | **Inclusion:** (a) 20 years of age or older (b) 16–20 weeks of gestation (c) no diagnosis of a major depressive episode in the past month, and (d) no diagnosis of lifetime bipolar disorder  **Exclusion:** N/S | **Channel:** Mobile application  **Component:** (a) Mindfulness (b) Psychoeducation (c) CBT (d) Behavioural activation | **Purpose:** Prevention of pregnancy-related major depressive episode  **Guiding theory:** N/S | **Delivery:** Has six sequential modules including psychoeducation, case formulation based on a cognitive–behavioural model, behavioural activation, self-compassion, mindfulness and problem solving. Each takes about 5 minutes to complete.  **Delivery mode:** Individual  **Frequency:** 1 module per week  **Facilitation mode:** Self-guided  **How Integrity was maintained:** N/S | **Time of initiation:** Antepartum  **Duration:** 6 weeks  **Period covered by intervention:** Antenatal only | TAU | **Depression:** EPDS (range: 0-30) | Mean ± SD  DHI: Baseline = 5.26 Post-intervention = 5.55  TAU Baseline = 5.07 Post-intervention = 5.41 |
| Qin 2022 [89] | **Aim:** To explore the preliminary effectiveness of the CareMom program on the universal prevention of postpartum depression, and to evaluate its acceptability.  **Country:** China (UMIC)  **Study Type:** RCT | SR only | **Age:** DHI = 31.6 ± 3.35 Control = 32.2 ± 3.89  **Sample size:** Total randomised = 112 DHI (allocated) = 57 Control (allocated) = 55 DHI (completed) = 52 Control (completed) = 53  **Pregnancy status:** Postpartum | **Inclusion:** (a) 0 days to 3 days after delivery (b) not diagnosed with any mental disorders at any time or experiencing severe depressive symptoms during the recruitment  (c) mother or baby not diagnosed with or experiencing any severe illnesses during the recruitment (d) no drug or alcohol issues over the past 12 months (e) not undergoing any kinds of psychological services or treatments (f) owned a smartphone, and (g) available to independently engage with the program for 4 weeks.  **Exclusion:** N/S | **Channel:** Instant messaging (WeChat)  **Component:** (a) Psychoeducation (b) CBT | **Purpose:** Prevention of PPD  **Guiding theory:** N/S | **Delivery:** The CareMom program is a WeChat mini program in Chinese language, and users can open the program by scanning a QR code in the WeChat application. It comprises two main components including daily challenges and mood management. The daily challenge component includes 28 challenges which users can complete each day. The first 14 challenges includes a video (about 2-4 minutes long) and a few quiz questions. The videos cover CBT topics related to postpartum depression and quizzes were designed to test users’ understanding of the video content. The last 14 challenges contain only the quiz questions. The program automatically releases one challenge each day as the user activates her account. If the user misses a daily challenge, she can complete that challenge in the later days. The mood management component is designed for users to record their daily moods and reflect on the events and thoughts related to the mood. When the user logs into the program for the first time in a day, she will be asked to rate her overall daily mood from five options: very good, good, neutral, bad, and very bad. If the user selects negative emotions (bad and very bad), the program will navigate her to reflect on the events and thoughts related to her negative emotions, and it will guide her to challenge and reconstruct her negative thoughts. When users select positive (good and very good) or neutral emotions, the program encourages them to record their positive events on the current day. Users can review their mood records via the mood calendar functionality. To motivate users to complete daily challenges and actively track their mood, gamification factors were incorporated into the design of the program.  **Delivery mode:** Individual  **Frequency:** Daily  **Facilitation mode:** Self-guided  **How Integrity was maintained:** N/S | **Time of initiation:** Postpartum  **Duration:** 4 weeks  **Period covered by intervention:** Postnatal only | TAU | **Depression:** EPDS (possible range: 0-30, higher scores indicative of higher depressive symptoms) | Mean ± SD  DHI: Baseline = 4.58 ± 3.37 Post-intervention = 2.71 ± 2.75  TAU Baseline = 5.42 ± 3.59 Post-intervention = 4.55 ± 3.87 |
| Sawyer 2019 [90] | **Aim:** To test the effectiveness of a 4-month online group–based nurse-led intervention delivered when infants were aged 2 to 6 months as compared with standard care outcomes.   **Country:** Australia (HIC)  **Study Type:** RCT | SR & MA | **Age:** DHI = 31.1 ± 5 Control = 32.2 ± 4  **Sample size:** Total randomised = 133 DHI (allocated & received intervention) = 70 Control (allocated & received intervention) = 61 DHI (completed) = 60 Control (completed) = 58  **Pregnancy status:** Postpartum | **Inclusion:** (a) EPDS score ≥7 (b) at least 1 self-reported parenting problem (c) literacy in English, and  (d) access to a smartphone.  **Exclusion:** (a) with an EPDS score >13 and who were judged by their screening nurse to have a level of depressive symptoms (b) experiencing domestic violence, illicit drug use, or other major distress  (3) lacked sufficient English skills to complete the self-report questionnaires. | **Channel:** Mobile application  **Component:** (a) CBT (b) Parenting education | **Purpose:** Treatment delivery option and postpartum support  **Guiding theory:** N/S | **Delivery:** A nurse-led, online group consisting of approximately 20 mothers of similarly aged infants, delivered when infants were aged approximately 2 to 6 months and was accessed by mothers via a mobile phone app. Nursing staff delivering the intervention were trained in the use and management of the app and in the mental health components of the intervention. The intervention was designed to reduce maternal depressive symptoms, support mothers to gain competence and self-efficacy in caring for their infants and solving caregiving difficulties, and support mothers to achieve healthy lifestyles for themselves and their infants. The “mother’s view” of the app comprises 4 component including a chat room where mothers post questions and nurses or other mothers can reply with posts and comments visible to all group members in a similar format to Facebook; a timeline which provides a list of child development milestones and health reminders that provides guidance to mothers appropriate to their baby’s age during the intervention; short articles and activities on parenting and emotional health; and useful contact numbers and a portal through which mothers can privately message their group’s nurse.  **Delivery mode:** Hybrid (contains both individual and group components)  **Frequency:** N/S  **Facilitation mode:** Hybrid (self-guided & therapist-facilitated)  **How Integrity was maintained:** N/S | **Time of initiation:** Postpartum  **Duration:** 4 months  **Period covered by intervention:** Postnatal only | TAU | **Depression:** EPDS (range: 0-30) | Mean ± SD  DHI: Baseline = 8.6 Post-intervention = 7.8  TAU Baseline = 9.6 Post-intervention = 8.8 |
| Seo 2022 [91] | **Aim:** To examine the effectiveness of the Happy Mother mobile app developed for self-management of postpartum depression, based on cognitive behavioural therapy.  **Country:** Korea (HIC)  **Study Type:** RCT | SR & MA | **Age:** DHI = 33.54 ± 3.30 Control = 33.36 ± 4.47  **Sample size:** Total randomised = 100 DHI (allocated & received intervention) = 50 Control (allocated & received intervention) = 50 DHI (completed) = 37 Control (completed) = 36  **Pregnancy status:** Postpartum | **Inclusion:** (a) belonging to a risk or high-risk group, with a score of 9 or higher on EPDS (b) within a year of childbirth, and (c) used Android-based smartphones.  **Exclusion:** (a) receiving psychiatric treatment | **Channel:** Mobile application  **Component:** (a) CBT (b) Psychoeducation | **Purpose:** Treatment delivery option  **Guiding theory:** N/S | **Delivery:** The Happy Mother app’s framework consists of psychoeducation, managing mood and negative thoughts, increasing pleasant activity, and facilitating help-seeking behaviour.  Psychoeducation provides information on self-diagnosing and understanding, treating, and overcoming postpartum depression; learning the role of a mother; and becoming a healthy mother. The management of mood begins with identifying the mother’s mood and sleep conditions and incorporating a diary where mood and sleep are captured. The management of negative thoughts involves identifying and countering them and has resources that encourages users to think differently, have a daily motto, and write in one’s diary. Furthermore, to increase pleasant activities, a happiness diary was added to the app.  **Delivery mode:** Individual  **Frequency:** N/S  **Facilitation mode:** Self-guided  **How Integrity was maintained:** N/S | **Time of initiation:** Postpartum  **Duration:** 8 weeks  **Period covered by intervention:** Postnatal only | TAU | **Depression:** EPDS (range: 0-30) | Mean ± SD  DHI: Baseline = 13.95 ± 4.56 Post-intervention = 10.70 ± 4.64  TAU Baseline = 15.03 ± 5.35 Post-intervention = 13.03 ± 6.19 |
| Shahsavan 2021 [110] | **Aim:** To implement I-GSH-CBT program among IPW during the first delivery and to determine its efficiency using the standard questionnaire-based scales.  **Country:** Iran (LMIC)  **Study Type:** RCT | SR only | **Age:** DHI = 28.10 ± 5.20 Control = 28.69 ± 5.31  **Sample size:** DHI (allocated) = 51 Control (allocated) = 51 DHI (completed) = 37 Control (completed) = 36  **Pregnancy status:** Pregnant | **Inclusion:** (a) Having an Iranian nationality (b) people without labour history or with history of less than three abortions (c) an age range of 18 to 35 years (d) a 30-week gestation, (e) not having high-risk pregnancies (f) no caesarean section indications (g) no mental and physical disabilities (h) having complete alertness (i) listening and speaking ability to answer questions (j) having access to the software program via the smartphone or tablet computer under the Android operating system (k) high levels of childbirth fear (score ≥ 85) based on the Wijma Delivery Expectancy/Experience Questionnaire, version A (W-DEQ-A) (l) a medium score range for depression (10-13), anxiety (10-14), and stress (14-20) according to the Depression, Anxiety, and Stress Scale 42-item questionnaire.  **Exclusion:** (a) unwillingness to cooperate in the plan. | **Channel:** Mobile application  **Component:** CBT | **Purpose:** Treatment delivery option  **Guiding theory:** N/S | **Delivery:** The program included information, skills, and exercises encouraging users to challenge thought patterns causing fear and anxiety. It also allowed users to counteract and reduce childbirth anxiety by managing their thoughts and emotions logically and mindfully. In general, the components of the program included self-monitoring, cognitive restructuring, relaxation, assertiveness, and problem-solving. At the end of each stage, individuals were asked to submit their homework to receive feedback from trained clinical psychologists.  **Delivery mode:** Individual  **Frequency:** N/S  **Facilitation mode:** Hybrid (predominantly self-guided and supported with feedback from therapists)  **How Integrity was maintained:** N/S | **Time of initiation:** Antepartum **Duration:** 8 weeks **Period covered by intervention:** Antenatal only | TAU | **Depression:** DASS-42-depression (Normal 0–9, Mild 10–13, Moderate 14–20, Severe 21–27, Extremely Severe 28+) | Mean ± SD  DHI: Baseline = 11.43 ± 0.20 Post-intervention = 10.40 ± 0.24  TAU Baseline = 15.53 ± 0.19  Post-intervention = 11.53 ± 0.36 |
| Shorey 2019 [92] | **Aim:** To evaluate the effectiveness of a technology-based peer-support intervention program (PIP) on maternal outcomes during the early postpartum period.  **Country:** Singapore (HIC)  **Study Type:** RCT | SR only | **Age:** 23-43  **Sample size:** Total randomised = 138 DHI (allocated and received intervention) = 69 Control (allocated and received intervention) = 69 DHI (completed) = 55 Control (completed) = 58  **Pregnancy status:** Postpartum | **Inclusion:** (a) aged at least 21 years (b) could read and speak English (c) owned a mobile phone and willing to share their number (d) planned to stay in Singapore for 3 months post birth (e) delivered a healthy baby without birth defects and/or medical complications, and (f) had a baseline Edinburgh Postnatal Depression Scale (EPDS) score of more than or equal to 9  **Exclusion:** (a) had a history of existing psychiatric illness, cognitive impairment, and/or major medical conditions  (b) had a vacuum- or forceps-assisted delivery with a fourth-degree perineal tear. | **Channel:** Multi: phone calls, emails, or mobile communication applications  **Component:** Peer support | **Purpose:** Postpartum support  **Guiding theory:** N/S | **Delivery:** The intervention involved correspondence with a trained peer volunteer via phone calls, emails, or mobile communication applications (e.g., WhatsApp), depending on each mother’s preference and convenience. The peer volunteer will initiate contact with the participant within 2 to 3 days post childbirth to discuss suitable timings for future correspondence. During the introductory phone session, both sides share their experience regarding emotional distress during the early postpartum period and extra efforts were made by the peer volunteer to build a strong relationship with the mother. Mothers were also informed that health care professionals would be notified if the mothers became too stressed during the correspondence. Subsequent sessions were individualized based on the unique needs of the mothers. Peer volunteers were encouraged to keep a free text journal of their conversations, and the intensity and duration of each correspondence were recorded in an activity log. Before the recruitment of postnatal mothers, the peer volunteers underwent a half-a-day training session by a psychiatrist. The training session inculcated roleplaying and strategizing to hone skills required in administering successful technology-based peer support. Volunteers were also taught to conduct appropriate referrals to health care professionals, should the need arise. A training booklet was prepared and given to each peer volunteer for future references.  **Delivery mode:** Individual  **Frequency:** at least once a week   **Facilitation mode:** Peer-facilitated  **How Integrity was maintained:** N/S | **Time of initiation:** Postpartum  **Duration:** 4 weeks  **Period covered by intervention:** Postnatal only | TAU | **Depression:** EPDS (range: 0-30)  PHQ-9 (possible range: 0-27; higher score indicating a higher severity of PND) | Mean ± SD  PHQ-9 DHI: Baseline = - Post-intervention = 4.6 ± 2.0  Control: Baseline = - Post-intervention = 6.2 ± 2.1   EPDS DHI: Baseline = - Post-intervention = 11.4 ± 2.0  Control: Baseline = - Post-intervention = 12.4 ± 2.1 |
| Suchan 2022 [93] | **Aim:** To examine the impact of a therapist-assisted, transdiagnostic ICBT program on symptoms of PPD and PPA.  **Country:** Canada (HIC)  **Study Type:** RCT | SR & MA | **Age:** 30.83 ± 4.29  **Sample size:** Total randomised = 63 DHI (allocated & completed baseline) = 28 Control (allocated & completed baseline) = 32 DHI (completed) = 25 Control (completed) = 29  **Pregnancy status:** Postpartum | **Inclusion:** (a) aged ≥18 years (b) be female (c) have given birth and have a child aged <1 year (d) have a score ≥10 on EPDS or score ≥9 on the 7-item GAD questionnaire (e) be a resident of Saskatchewan (f) be comfortable using technology (g) have access to a secure computer and the internet, and (h) be willing to provide a medical contact as an emergency contact.  **Exclusion:** (a) were hospitalized in the prior year for mental health concerns or suicidality (b) had unmanaged alcohol or drug use, mania, or psychosis, or (c) started a new psychotropic medication within the past month. | **Channel:** Online (web-based)  **Component:** CBT | **Purpose:** Treatment delivery option  **Guiding theory:** N/S | **Delivery:** The intervention has 5 lessons. Lesson 1 (1 week) provides psychoeducation about anxiety and depression in general and in postpartum population. Lesson 2 (2 weeks) provides information on unhelpful thoughts. Lesson 3 (1 week) comprises psychoeducation on physical symptoms in relation to the CBT model (e.g., controlled breathing). Lesson 4 (2 weeks) focuses on information related to unhelpful behaviours. The fifth and final lesson (2 weeks) includes information about relapse prevention, normalization, and creation of relapse prevention plans. Each lesson includes case stories and do-it-yourself guides, as well as additional resources that could be accessed at any point throughout the course. Participants received asynchronous weekly therapist support from a CBT-trained social support worker. The therapist contacted clients on the same day each week using secure emails on the Online Therapy Unit’s platform. Each therapist message was personalized but included several important elements. Phone calls were made to clients in specific sensitive cases. The clients also received automated messages as reminders of new lessons or questionnaires to complete.  **Delivery mode:** Individual  **Frequency:** Therapist support is weekly  **Facilitation mode:** Hybrid (predominantly self-guided with accompanying therapist support)  **How Integrity was maintained:** N/S | **Time of initiation:** Postpartum  **Duration:** 8 weeks  **Period covered by intervention:** Postnatal only | TAU | **Depression:** EPDS (possible range: 0-30; cutoff score = ≥10)  DASS-21-depression (possible score range: 0-21; higher score indicates higher symptoms). | Mean ± SD  EPDS DHI: Baseline = 14.47 ± 4.27 Post-intervention = 9.54 ± 5.59  Control: Baseline = 15.44 ± 4.79 Post-intervention = 12.35 ± 5.04   DASS-21-Depression DHI: Baseline = 6.83 ± 5.11 Post-intervention = 4.08 ± 3.59  Control: Baseline = 7.57 ± 5.22 Post-intervention = 5.62 ± 4.48 |
| Suharwardy 2023 [94] | **Aim:** To evaluate the acceptability and preliminary efficacy of a mental health chatbot for mood management in a general postpartum population.  **Country:** United States (HIC)  **Study Type:** RCT | SR & MA | **Age:** Mean = 34  **Sample size:** Total randomised = 192 DHI (allocated) = 96 Control (allocated) = 96 DHI (completed) = 84 Control (completed) = 68  **Pregnancy status:** Postpartum | **Inclusion:** (a) English-speaking (b) being 18 years or older, and (c) access to a smartphone.  **Exclusion:** (a) a foetal or neonatal demise | **Channel:** Mobile application  **Component:** (a) CBT (b) IPT | **Purpose:** Treatment delivery option  **Guiding theory:** N/S | **Delivery:** The program is a chatbot embedded in a mobile application. It invites users to track and discover patterns in their mood through text-based conversations in the smartphone application. Its language detection protocol (LDP) uses natural language processing algorithms to detect and flag potential self-harm phrases. The purpose of the LDP is to detect concerning topics within patient-input free-text. Upon detection and confirmation of any concerning topics, LDP initiates a conversation between the chatbot and the participant to remind them of the application's limitations of services and offer a resource list which includes readily accessible support channels.  **Delivery mode:** Individual  **Frequency:** N/A  **Facilitation mode:** Self-guided  **How Integrity was maintained:** N/S | **Time of initiation:** Postpartum  **Duration:** 6 weeks  **Period covered by intervention:** Postnatal only | TAU | **Depression:** EPDS (possible range: 0-30)  PHQ-9 | Mean ± SD  EPDS DHI: Baseline = 5.51 ± 4.70 Post-intervention = 4.88 ± 5.26  Control: Baseline = 5.37 ± 4.20 Post-intervention = 4.61 ± 5.20   PHQ-9 DHI: Baseline = 4.41 ± 4.29 Post-intervention = 3.09 ± 3.02  Control: Baseline = 3.36 ± 3.05 Post-intervention = 3.23 ± 3.84 |
| Van 2021 [95] | **Aim:** To determine whether an online 1-day cognitive behavioural therapy (CBT)–based workshop added to treatment as usual improves PPD, anxiety, social support, mother-infant relationship quality, and infant temperament more than treatment as usual alone.  **Country:** Canada  **Study Type:** RCT | SR & MA | **Age:** 31.8 ± 4.4  **Sample size:** Total randomised = 403 DHI (allocated and received intervention) = 161 Control (allocated) = 201 DHI (completed) = 124 Control (completed) = 192  **Pregnancy status:** Postpartum | **Inclusion:** (a) 18 years or older (b) had an infant younger than 12 months (c) lived in Ontario, and (d) had an EPDS score of at least 10.  **Exclusion:** N/S | **Channel:** Videoconferencing (Zoom)  **Component:** CBT | **Purpose:** Treatment delivery option  **Guiding theory:** N/S | **Delivery:** The workshops were delivered by a registered psychotherapist, a clinical psychology graduate student, or a psychiatrist. Therapists completed 1 day of in-classroom training and had 1 workshop observed before delivering the workshops. They were randomly assigned to deliver workshops.  The intervention was from 9 am to 4 pm and an interactive workshop consisting of didactic teaching, group exercises/discussion, and role playing in 4 modules. The first module reviewed PPD aetiology with a focus on modifiable cognitive risk factors (negative thoughts, maladaptive core beliefs). The second module focused on cognitive skills, including cognitive restructuring. The third module built behavioural skills such as problem solving, behavioural activation, and assertiveness. The fourth module provided an opportunity for goal setting and action planning. Participants received a workshop manual before their workshop took place   **Delivery mode:** Group  **Frequency:** N/A  **Facilitation mode:** Therapist-facilitated  **How Integrity was maintained:** N/S | **Time of initiation:** Postpartum  **Duration:** 1 day  **Period covered by intervention:** Postnatal only | TAU | **Depression:** EPDS (possible range: 0-30; cutoff score = ≥10) | Mean ± SD  DHI: Baseline = 16.47 ± 4.41 Post-intervention = 11.65 ± 4.83  TAU Baseline = 15.92 ± 4.54 Post-intervention = 14.04 ± 4.54 |
| Vigod 2021 [96] | **Aim:** To evaluate the feasibility, acceptability, participant adherence, and preliminary efficacy estimates in relation to postpartum depressive symptomatology.  **Country:** Canada (HIC)  **Study Type:** RCT | SR only | **Age:** DHI = 33.4 ± 4.49 Control = 32.6 ± 5.51  **Sample size:** Total randomised = 98 DHI (allocated & completed baseline) = 49 Control (allocated & completed baseline) = 47 DHI (completed) = 37 Control (completed) = 40  **Pregnancy status:** Postpartum | **Inclusion:** (a) identified as a mother (inclusive of all genders, adoptive and birth parents) (b) 18 years or older  (c) with an infant between 0 and 12 months old living with them (d) resided in Ontario, and (e) had an EPDS score of 10 or above.  **Exclusion:** (a) active suicidal ideation (b) mania, psychosis, or a substance or alcohol use disorder (c) without internet access, or (d) unable to read or write in English | **Channel:** Online (web-based)  **Component:** IPT | **Purpose:** Treatment delivery option  **Guiding theory:** N/S | **Delivery:** The intervention comprises 10 weekly topics covering: psychoeducation (Weeks 1 and 2), issues related to obtaining adequate social support (Week 3), and interpersonal problem areas (Weeks 4–9), including challenges related to baby's sleep and feeding, maternal identity, and interpersonal relationships with partners. The final week (Week 10) was for consolidation and saying goodbye to the group. Two mental health therapists facilitated the intervention.  There was also a weekly optional “live chat” hour where the therapists attended, and participants could join in to have further discussion about the weekly topic and strategies associated with it.  **Delivery mode:** Group  **Frequency:** Weekly  **Facilitation mode:** Therapist-facilitated  **How Integrity was maintained:** N/S | **Time of initiation:** Postpartum  **Duration:** 10 weeks  **Period covered by intervention:** Postnatal only | TAU | **Depression:** EPDS (possible range: 0-30; cutoff score = ≥10) | Mean ± SD  DHI: Baseline = 14.5 ± 4.07 Post-intervention = 11.3 ± 4.54  TAU Baseline = 15.0 ± 3.56 Post-intervention = 12.0 ± 4.79 |
| Wu 2019 [111] | **Aim:** To investigate the effects of a virtual community on pregnant women's well-being.  **Country:** Taiwan (HIC)  **Study Type:** Quasi-experimental; double arm | SR only | **Age:** DHI = 32.79 ± 3.87 Control = 32.78 ± 3.82  **Sample size:** DHI (allocated) = 66 Control (allocated) = 55 DHI (completed) = 59 Control (completed) = 50  **Pregnancy status:** Pregnant | **Inclusion:** (a) Pregnant women (b) over 20 years old (c) had at least 9 years of education (d) in less than 12 weeks of gestation (e) did not have complications or underlying medical problems in their pregnancy, and (f) had convenient access to the Internet.  **Exclusion:** N/S | **Channel:** Social media (Facebook)  **Component:** (a) Pregnancy health education (b) Peer-to-peer support | **Purpose:** Pregnancy support  **Guiding theory:** N/S | **Delivery:** The intervention comprised two main components, a discussion forum and a library area for pregnancy-related information. For the discussion forum, according to members’ willingness, writing posts, offering comments, proposing questions, and writing feedback or sharing personal experiences were encouraged. The library area contained pre-uploaded pregnancy information compiled by the researchers, including information on foetal growth, week-by-week pregnancy progress, a prenatal check-up schedule and contents and pregnancy nutrition.  **Delivery mode:** Group  **Frequency:** N/A  **Facilitation mode:** Peer-to-peer  **How Integrity was maintained:** N/S | **Time of initiation:** Antepartum  **Duration:** 24 weeks+  **Period covered by intervention:** Antenatl only | TAU | **Depression:** EPDS (possible range: 0-30; cutoff score = ≥12) | - |
| Yang 2019 [97] | **Aim:** To test the feasibility and acceptability of an online mindfulness intervention for pregnant women as an approach to reduce depressive and anxious symptoms.  **Country:** China (UMIC)  **Study Type:** RCT | SR only | **Age:** DHI = 31.31 ± 4.97 Control = 30.38 ± 3.91  **Sample size:** Total randomised = 123 DHI (allocated & completed baseline) = 62 Control (allocated & completed baseline) = 61 DHI (completed) = 52 Control (completed) = 50  **Pregnancy status:** Pregnant | **Inclusion:** (a) women aged more than 18 years (b) 24 to 30 weeks’ gestation (c) low-risk pregnancy at the start of the intervention (d) internet access (e) fluent in Chinese and able to complete the questionnaires, and (f) elevated depressive or anxious symptoms  **Exclusion:** (a) history or current diagnosis of a psychosomatic disease  (b) current substance abuse (c) previous participation in psychological therapy or a stress reduction program (d) history of suicide attempts (e) current use of any psychoactive drug (f) a high level of depression or anxiety  (g) Women who had regular mind-body practice | **Channel:** Instant messaging (WeChat)  **Component:** Mindfulness | **Purpose:** Treatment delivery option  **Guiding theory:** N/S | **Delivery:** The intervention included 4 sessions which were recorded and uploaded to the WeChat platform for participants to access. Additional text, pictures, and audios related to the course were available for participants to review.  Each session started with a 15-minute review of the practice that addressed the questions and difficulties that women found in mindfulness practice and guiding the brief mindfulness practice. The new content in each session took 25 to 30 minutes. Each session ended with a 5-minute assignment of homework.  All questions and consultations related to the intervention or pregnancy were addressed within 48 hours.  **Delivery mode:** Group  **Frequency:** N/S  **Facilitation mode:** Therapist-facilitated  **How Integrity was maintained:** N/S | **Time of initiation:** Antepartum **Duration:** 8 weeks **Period covered by intervention:** Antenatal only | TAU | **Depression:** PHQ-9 (possible range: 0-27) | Mean ± SD  DHI: Baseline = 5.98 ± 2.24 Post-intervention = 3.58 ± 2.32  TAU Baseline = 5.72 ± 2.65 Post-intervention = 6.26 ± 3.31 |
| Zhang 2023 [98] | **Aim:** To investigate the effectiveness of a digital guided self-help mindfulness-based intervention in reducing maternal psychological distress and improving infant neuropsychological performance.  **Country:** China (UMIC)  **Study Type:** RCT | SR & MA | **Age:** 30.29 ± 4.29  **Sample size:** Total randomised = 160 DHI (allocated & completed baseline) = 80 Control (allocated & completed baseline) = 80 DHI (completed) = 78 Control (completed) = 79  **Pregnancy status:** Pregnant | **Inclusion:** (a) aged ≥18 years (b) have single pregnancy (c) be at 12 to 20 weeks of gestation (d) have EPDS score of ≥9 or GAD-7 scale score of ≥5 (e) be fluent in reading and writing Chinese (f) not participated in any other psychological intervention, and (g) be able to access the WeChat mini program.  **Exclusion:** (a) had suicidal ideation (b) serious mental disorders (e.g., schizophrenia and bipolar disorder) or physical illnesses (c) drug abuse or dependence, or (d)prior experience in mindfulness exercises. | **Channel:** Instant messaging (WeChat)  **Component:** Mindfulness | **Purpose:** Treatment delivery option  **Guiding theory:** N/S | **Delivery:** The intervention included 6 modules, each of which lasted for 1 week. Each module consisted of thematic video lessons and homework. Each video was 10- to 20-minute long and included a variety of cartoon images and mindfulness practice demonstrations. The formal practice involved daily audio-based practices, such as mindful breathing and body scan. They were sent standardized practice reminders every day by the research assistant via WeChat.   **Delivery mode:** Individual  **Frequency:** 1 module per week  **Facilitation mode:** Self-guided  **How Integrity was maintained:** N/S | **Time of initiation:** Antepartum **Duration:** 6 weeks **Period covered by intervention:** Antenatal only | TAU | **Depression:** EPDS (possible range: 0-30) | Mean ± SD  DHI: Baseline = 8.91 ± 3.54 Post-intervention = 5.21 ± 4.46  TAU Baseline = 9.43 ± 3.26 Post-intervention = 7.86 ±  5.07 |

# SUPPLEMENTARY FILE – FOREST PLOTS OF SUBGROUP ANALYSES

## Subgroup analysis of economic regions


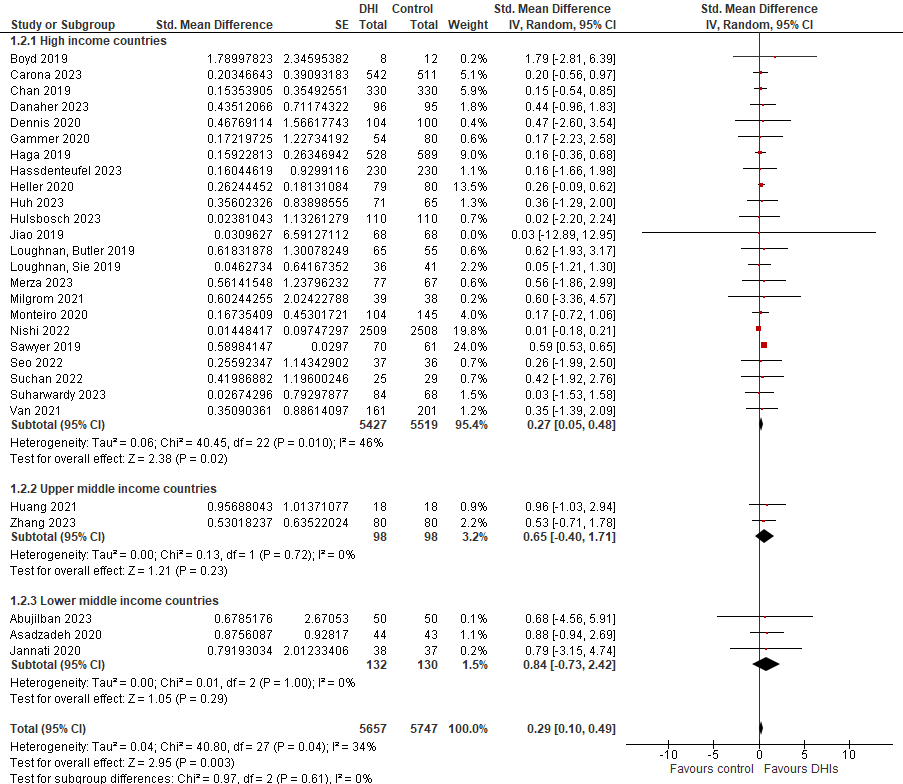


## Subgroup analysis of intervention platforms


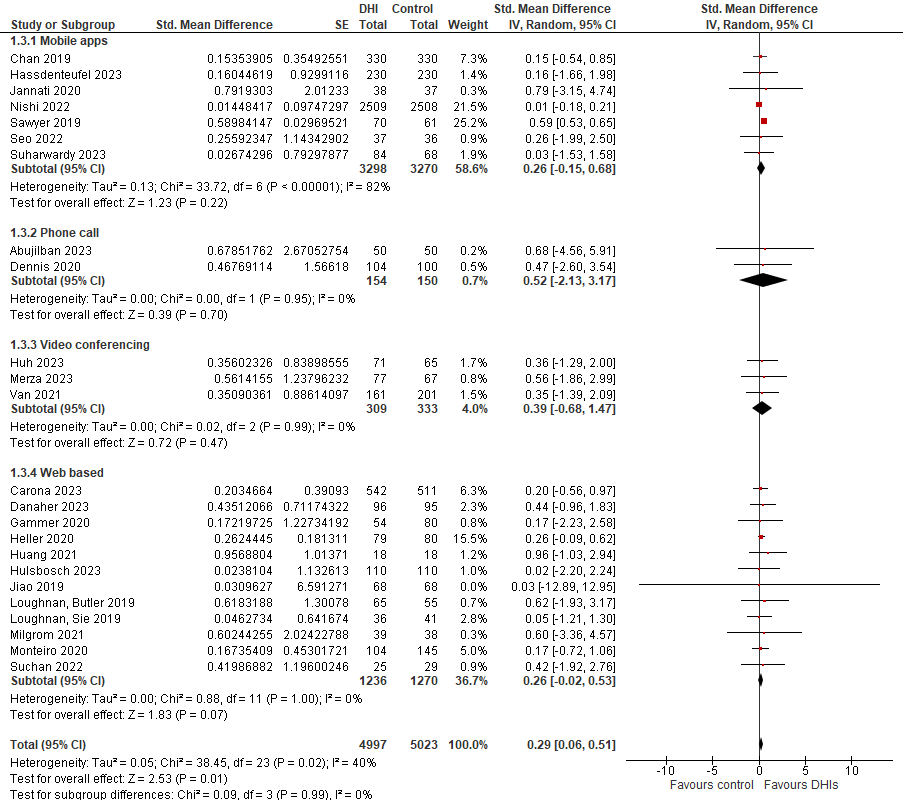


## Subgroup analysis of intervention delivery mode


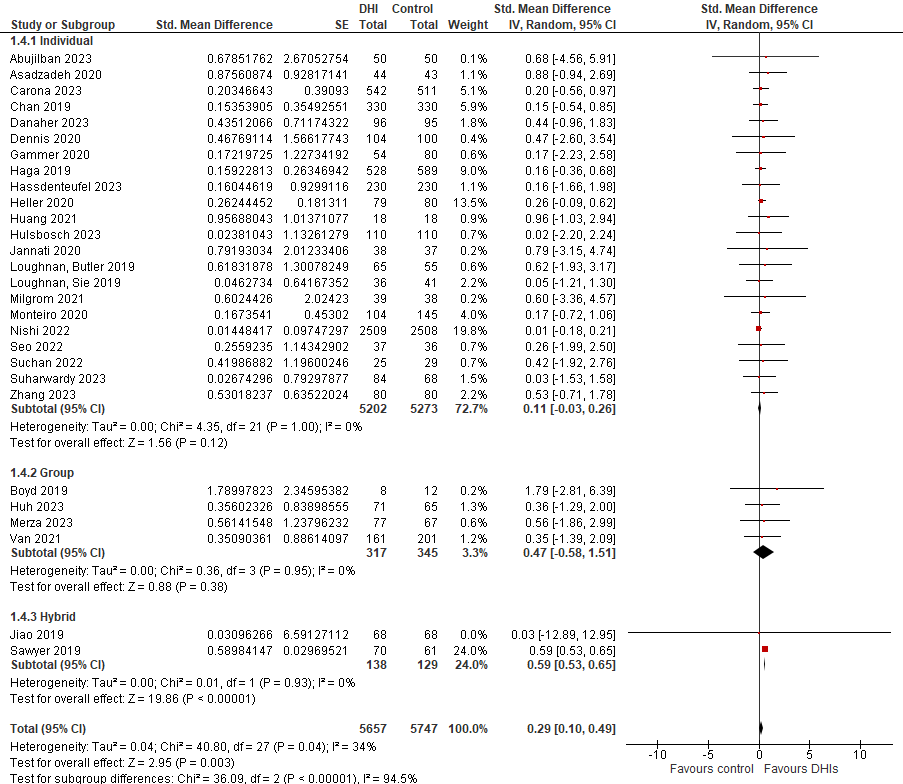


## Subgroup analysis of intervention facilitation mode


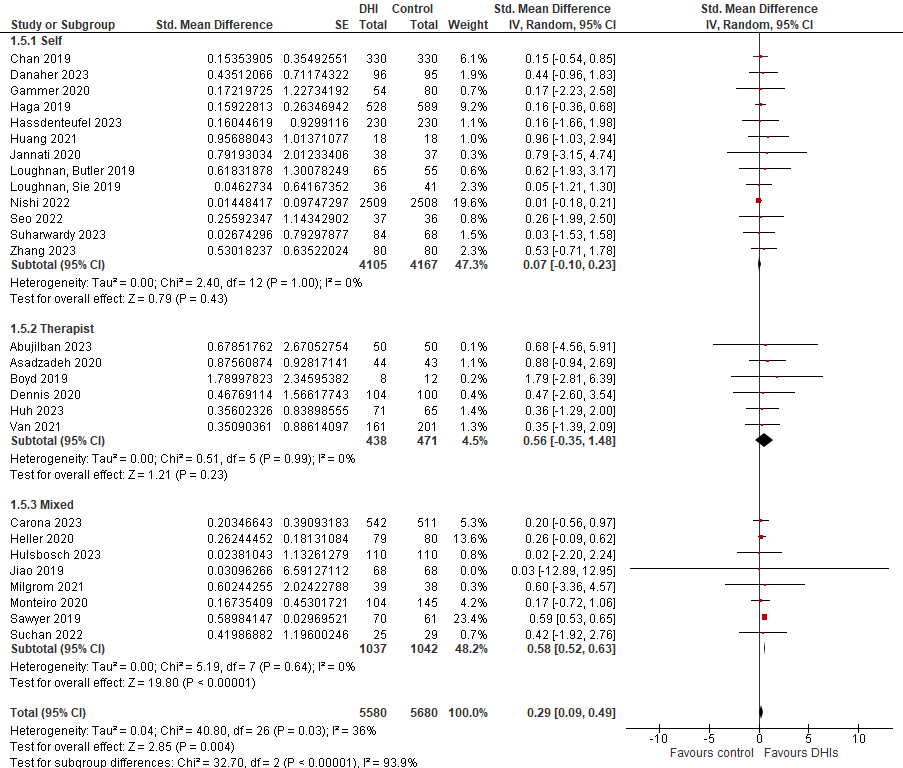


## Subgroup analysis of time of intervention initiation


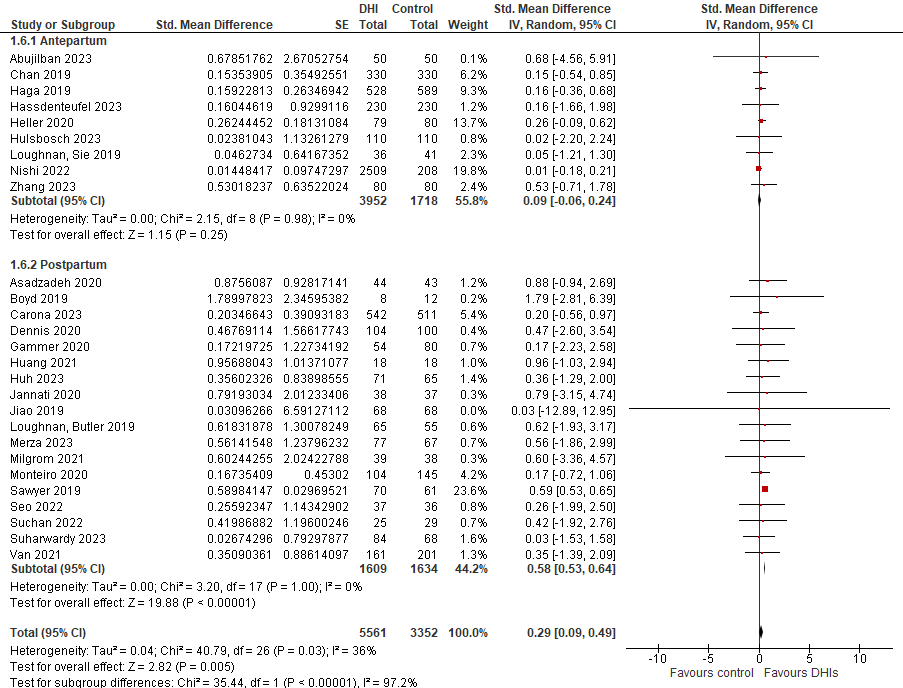


## Subgroup analysis of period covered by intervention.


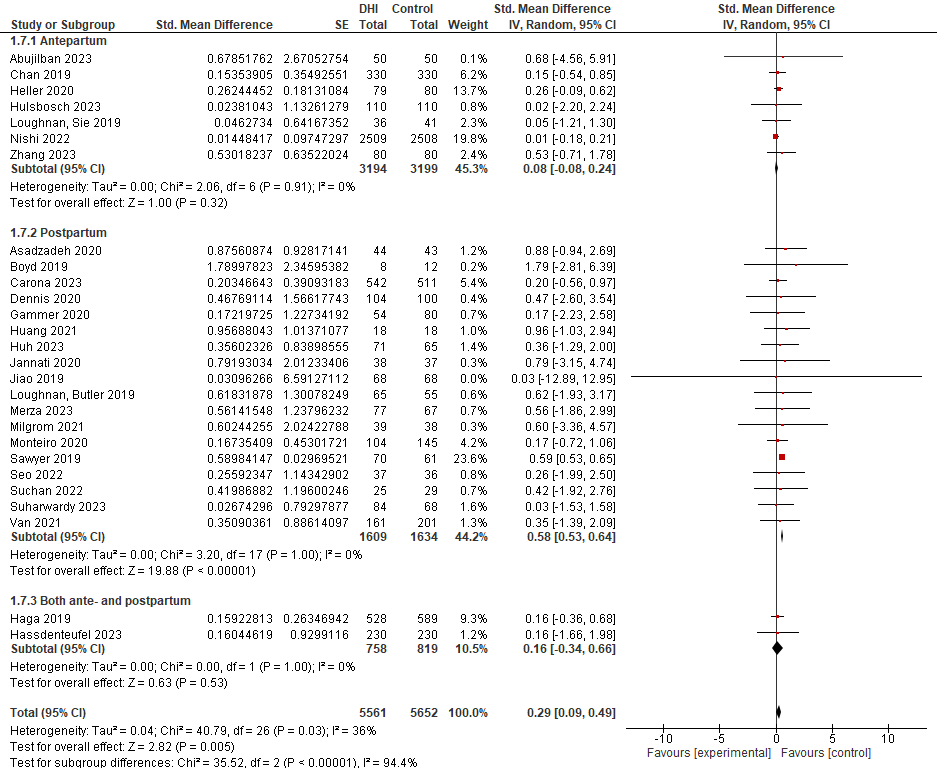


## Subgroup analysis of duration of intervention


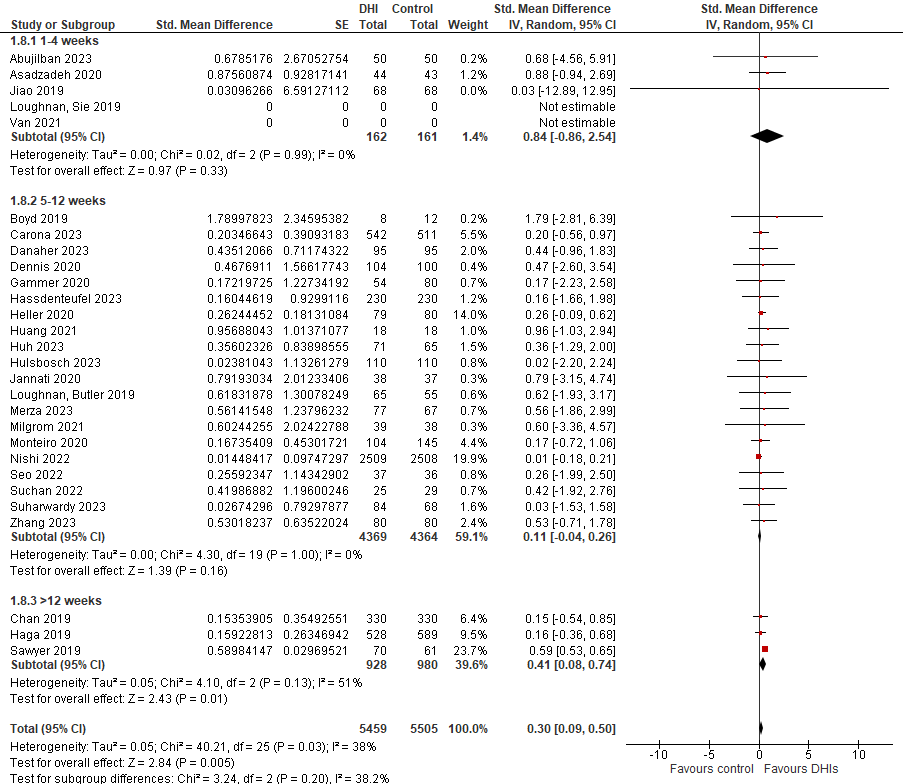

Supplement: Supplementary_file_oqae026 [file Supplementary_file_oqae026.docx]
